# Direct C–H difluoromethylation of heterocycles via organic photoredox catalysis

**DOI:** 10.1038/s41467-020-14494-8

**Published:** 2020-01-31

**Authors:** Wei Zhang, Xin-Xin Xiang, Junyi Chen, Chen Yang, Yu-Liang Pan, Jin-Pei Cheng, Qingbin Meng, Xin Li

**Affiliations:** 1 State Key Laboratory of Elemento-Organic Chemistry, College of Chemistry, Nankai University, Tianjin, 300071 China; 2 State Key Laboratory of Toxicology and Medical Countermeasures, Beijing Institute of Pharmacology and Toxicology, Beijing, 100850 China

**Keywords:** Photocatalysis, Synthetic chemistry methodology, Organic chemistry

## Abstract

The discovery of modern medicine relies on the sustainable development of synthetic methodologies to meet the needs associated with drug molecular design. Heterocycles containing difluoromethyl groups are an emerging but scarcely investigated class of organofluoro molecules with potential applications in pharmaceutical, agricultural and material science. Herein, we developed an organophotocatalytic direct difluoromethylation of heterocycles using O_2_ as a green oxidant. The C–H oxidative difluoromethylation obviates the need for pre-functionalization of the substrates, metals and additives. The operationally straightforward method enriches the efficient synthesis of many difluoromethylated heterocycles in moderate to excellent yields. The direct difluoromethylation of pharmaceutical moleculars demonstrates the practicability of this methodology to late-stage drug development. Moreover, 2′-deoxy-5-difluoromethyluridine (F_2_TDR) exhibits promising activity against some cancer cell lines, indicating that the difluoromethylation methodology might provide assistance for drug discovery.

## Abstract (web summary)

Heterocycles containing difluoromethyl groups are molecules with potential application in pharmaceutical, agricultural and materials science. Here, the authors show an organophotocatalytic difluoromethylation of heterocycles using O_2_ as green oxidant and preliminarily study the products’ bioactivity.

## Introduction

**Fig. 1:**
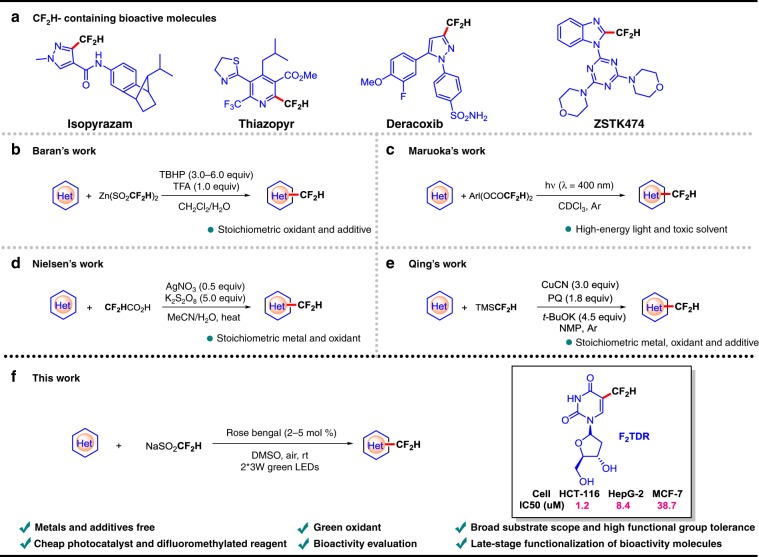
Construction of CF_2_H-containing heterocycles. **a** Examples of CF_2_H- bioactive molecules. **b** TBHP promoted direct difluoromethylation of heterocycles. **c** High-energy light promoted direct difluoromethylation of heterocycles. **d** Ag and K_2_S_2_O_8_ co-catalyzed direct difluoromethylation of heterocycles. **e** Cu and PQ co-catalyzed direct difluoromethylation of heterocycles. **f** Organic photoredox catalysis triggered direct difluoromethylation of heterocycles.

Organofluoro compounds are widely used in the fields of pharmaceutical, agricultural, and material science^1–3^. The introduction of fluorine atoms or fluorine-containing groups into the framework of organic molecules often changes the physicochemical properties or biological activities of compounds^4–8^, and has become an essential topic for chemists. During the past few decades, much attention has been focused on the synthesis of fluorinated^9–12^ and trifluoromethylated molecules^13–19^. On the other hand, the difluoromethyl is also a critical fluorinated functional group due to its use as a lipophilic hydrogen bond donor. In addition, CF_2_H group can be considered as the isostere of a thiol, a hydroxyl, and an amide, which brings out its potential value in drug development with novel scaffold^20,21^. However, unlike well-developed synthesis of fluorinated and trifluoromethylated compounds, the construction of difluoromethylated substances^22–27^, especially for difluoromethylated heterocycles, which widely exist in bioactive molecules (Fig. 1a), has been less explored. The traditional strategies for the synthesis of difluoromethylated heterocycles include deoxyfluorination of aldehydes^28^, difluorination of benzylic C−H bonds^29,30^, decarbonylation/decarboxylation difluoromethylation^31,32^, cycloaddition reactions^24,33,34^, and conversion of CF_2_R containing heterocycles precursors^35,36^. However, these above-mentioned methods need expensive and toxic fluorinating agents, harsh reaction conditions, and are limited in functional group compatibility. Recently, difluoromethylation of heteroaromatic compounds catalyzed by transition metals^37^, such as copper, palladium, nickel, has been developed. However, most of these approaches depend on preactivation of substrates (i.e., aryl halides^38–42^, aryl boronic acids^43–45^, arylzincs^46^, arenediazonium salts^47^).

The radical-based difluoromethylation method has been developed as an advantageous synthetic strategy for the construction of difluoromethylated heterocycles. The pioneering work was reported by Baran and co-workers (Fig. 1b), in which they developed a new difluoromethylated reagent Zn(SO_2_CF_2_H)_2_ (DFMS) that can effectively release CF_2_H radical in the presence of *tert*-butyl hydroperoxide (TBHP) as a stoichiometric oxidant^48^. Sakamoto et al. presented a photolytic direct C–H difluoromethylation of heterocycles using hypervalent iodine(III) reagents that contain difluoroacetoxy ligands (Fig. 1c)^49^. Shortly afterward, Tung et al. developed direct difluoromethylation of heterocycles using difluoroacetic acid as CF_2_H radical precursors via transition metal catalysis (Fig. 1d)^50^. In 2018, Zhu et al. reported a new strategy through the copper-mediated C−H oxidative difluoromethylation of heterocycles with TMSCF_2_H (Fig. 1e)^51^. It should be noted that these protocols of direct difluoromethylation of heterocycles need either expensive/toxic metal catalysts or external oxidants and strictly inert conditions, which narrow the functional group tolerance and limit the substrate scope. During the submission of this manuscript, Duan and Xia reported an efficient photocatalytic strategy for C–H perfluoroalkylation of quinoxalinones under aerobic oxidation conditions, however, the corresponding difluoromethylation has not been reported^52^. Therefore, it is highly desirable to develop new approaches to achieve direct C–H difluoromethylation of heterocycles, which can overcome the above-mentioned defects and avoid the safety problems with stoichiometric oxidant at a larger scale.

In the past decade, visible-light catalysis has attracted extensive attention, by which highly active radical species generated under mild conditions can be involved in various chemical bond formation^53–59^. Notably, organic dyes are cheaper and more reliable than expensive metal photoredox catalysts in many valuable reactions^55,56,59^. In this study, we focus our attention on developing an straightforward and practical method for difluoromethylation reactions using the inexpensive, commercially available, and user friendly Hu’s reagent sodium difluoromethane sulfonate (CF_2_HSO_2_Na) as the difluoromethyl radical precursor^60^ under mild conditions. This protocol combines organic photocatalysis with green air oxidant for the difluoromethylation of many categories of heterocycles. Notably, the difluoromethylation product 2′-deoxy-5-difluoromethyluridine (F_2_TDR) commendably inhibits the proliferation of cancer cells, such as HCT116, HepG-2, and MCF-7 (Fig. 1f).

## Results

### Investigation of the reaction conditions

We started our investigation by the model reaction of 1-methyl quinoxolin-2-one **1a** with CF_2_HSO_2_Na **2** as a fluorine source and eosin Y as a photocatalyst in DMSO at room temperature under green LEDs irradiation. To our delight, the difluoromethylation product **3a** was obtained in 64% yield (Table 1, entry 1). Then, different photocatalysts were examined (Table 1, entries 2–8), in which rose bengal (RB) was the best one, providing **3a** in 72% yield in 12 h. After identifying the optimal photocatalyst, we found that DMSO was the best reaction media through solvent screening (Table 1, entries 2 and 9–16). The yield of product reduced obviously when the loading of the photocatalyst was further decreased to 1 mol% (Table 1, entry 17). Control experiments indicated that photocatalyst and green light source were both essential for the reaction efficiency (Table 1, entries 18 and 19).

**Table 1:** Reaction optimization^a,b^.


Entry	Photocatalysts	Solvent	Time (h)	Yield^b^/%
1	Eosin Y	DMSO	15	64
2	Rose bengal	DMSO	12	72
3	Acridinium salt	DMSO	36	64
4	DCA	DMSO	15	NR
5	MB	DMSO	15	Trace
6	Ensin B	DMSO	24	61
7	Ru(bpy)_3_Cl_2_·6H_2_O	DMSO	36	62
8	*fac*-Ir(ppy)_3_	DMSO	36	56
9	Rose bengal	DMF	12	10
10	Rose bengal	MeCN	12	20
11	Rose bengal	MeOH	12	51
12	Rose bengal	CHCl_3_	12	Trace
13	Rose bengal	Acetone	12	55
14	Rose bengal	Toluene	12	NR
15	Rose bengal	EtOAc	12	15
16	Rose bengal	1,4-dioxane	12	22
17^c^	Rose bengal	DMSO	12	51
18	–	DMSO	12	NR
19^d^	Rose bengal	DMSO	12	NR


*NR* no reaction.

^a^The reactions were carried out with **1a** (0.2 mmol), CF_2_HSO_2_Na **2** (0.4 mmol), photocatalyst (2 mol%) in 1 mL solvent under two 3 W green LEDs irradiation at room temperature.

^b^Isolated yield.

^c^The photocatalyst loading was decreased to 1 mol%.

^d^In the dark.

### Scope of quinoxalin-2(*1H*)-ones on the phenyl ring

**Fig. 2:**
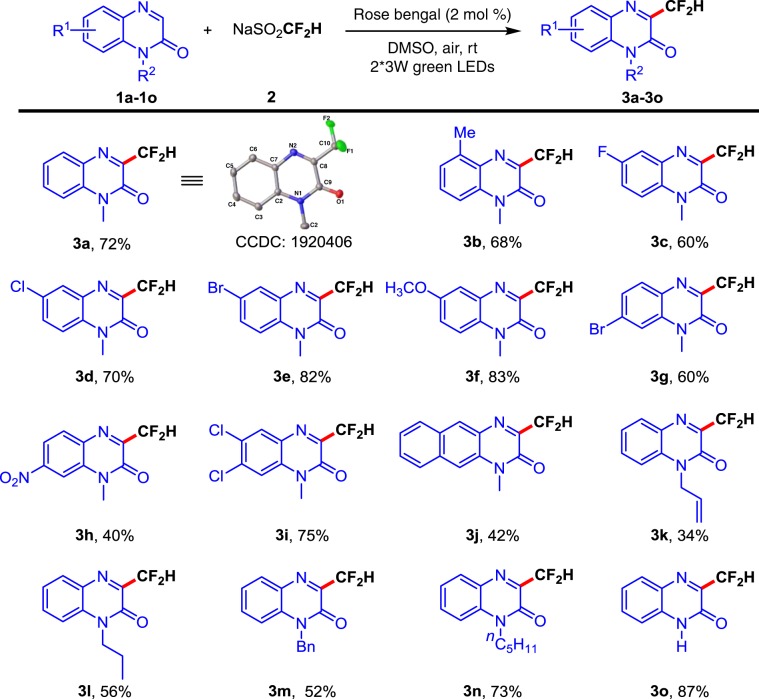
Substrate scope of quinoxalin-2(1*H*)-ones. Reaction conditions: **1** (0.2 mmol), CF_2_HSO_2_Na **2** (0.4 mmol), rose bengal (2 mol%) in 1 mL DMSO under two 3 W green LEDs irradiation at room temperature. Isolated yields based on **1**.

With the optimal reaction conditions in hand, the substrate scope of various quinoxalin-2(1*H*)-ones was then investigated. As exhibited in Fig. 2, the reactions worked well with a range of substituted quinoxalin-2(1*H*)-ones bearing either electron-donating or -withdrawing substituents (methyl, fluoro, chloro, bromo, methoxy, nitro, and naphthyl), giving the desired difluoromethylation products **3a**–**3j** in moderate to good yields. To highlight the utility of this transformation, a variety of *N*-substituted quinoxalin-2(1*H*)-ones (**1k−1n**) were also tested. As a result, all the *N*-substituted quinoxalin-2(1*H*)-ones are compatible with the reaction, furnishing the expected products **3k–3n** in 34–73% yields. Notably, *N*-unsubstituted quinoxalin-2(1*H*)-one also proceeded smoothly, delivering the desired product **3o** in 87% yield.

### Scope of heteroaromatics

**Fig. 3:**
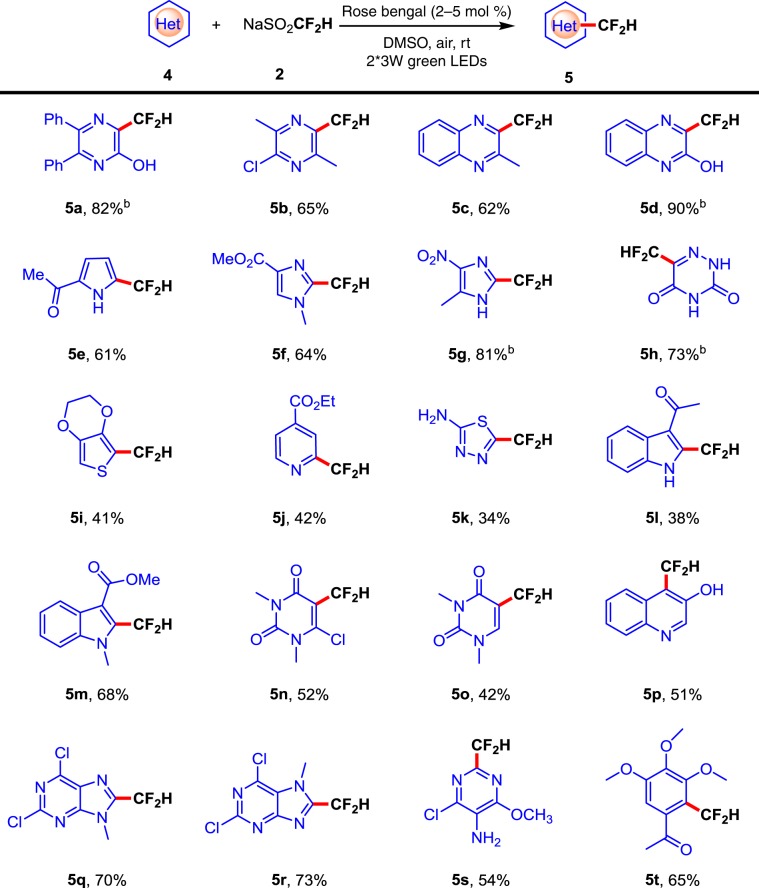
Substrate scope of heteroaromatics. Reaction conditions: **4** (0.1 mmol), CF_2_HSO_2_Na **2** (0.4 mmol), rose bengal (5 mol.%) in 1 mL DMSO under two 3 W green LEDs irradiation at room temperature. Isolated yields based on **4**. ^b^With CF_2_HSO_2_Na **2** (0.4 mmol), rose bengal (2 mol%) in 1 mL DMSO.

To further extend the scope of this methodology, some other heteroaromatic substrates were investigated (Fig. 3). A wide range of five- and six-membered difluoromethylated heteroarenes, such as pyrazines (**5a** and **5b**), quinoxalines (**5c** and **5d)**, pyrrole (**5e**), imidazoles (**5f** and **5g**), thiophene (**5i**), pyridines (**5j**), thiadiazole (**5k**), indoles (**5l** and **5m**), RNA-based dimethyluracils (**5n** and **5o**), quinoline (**5p**), purine derivatives (**5q** and **5r**), and pyrimidine (**5 s**), provided final products with good yields. Several heteroarenes with potentially sensitive functional groups (OH, NH_2_, or CHO) (**5a**, **5k**, and **6j**) that are barely reported in the previous study^48–51^ are also compatible with this difluoromethylation strategy. Moreover, this direct C–H difluoromethylation method also suitable for some arenes (**5t** and **6j**).

### Scope of bioactive molecules

**Fig. 4:**
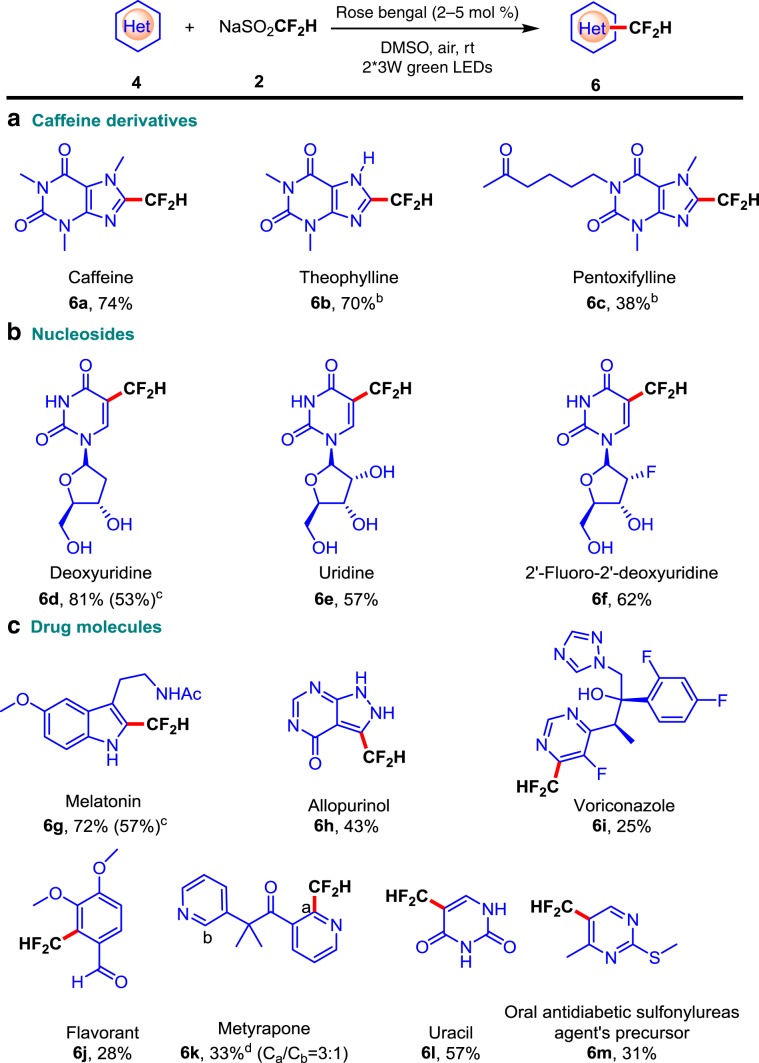
Substrate scope of bioactive molecules. Reaction conditions: **4** (0.1 mmol), CF_2_HSO_2_Na **2** (0.4 mmol), rose bengal (5 mol%) in 1 mL DMSO under two 3 W green LEDs irradiation at room temperature. Isolated yields based on **4**. ^b^With CF_2_HSO_2_Na **2** (0.4 mmol), rose bengal (2 mol%) in 1 mL DMSO. ^c^Large scale with 2 mmol heteroarenes. ^d^The minor regioisomeric position is labeled with the respective carbon atom number.

The methodology can also be applied to late-stage functionalization of complex nitrogen-containing bioactive molecules (Fig. 4). For example, the modification of caffeine and its derivatives delivers the desired difluoromethylated products **6a**–**6c** in 38–74% yields. In addition, deoxyuridine, uridine, and 2′-fluoro-2′-deoxyuridine, which have free OH group as well as amide group, could tolerate this difluoromethylation reaction, leading to **6d**–**6f** in 57–81% yields. Furthermore, melatonin, allopurinol, and uracil, which bear free secondary N–H groups, furnished the difluoromethylation reaction with moderate to good yields (**6g**, **6h**, and **6l**). Moreover, some other bioactive heteroarene substrates, such as voriconazole, flavorant, metyrapone, and sulfonylureas, can also proceed well with the reaction, giving the difluoromethylated products **6i**, **6j**, **6k**, and **6m** in acceptable yields.

### Site-selectivity study

**Fig. 5:**
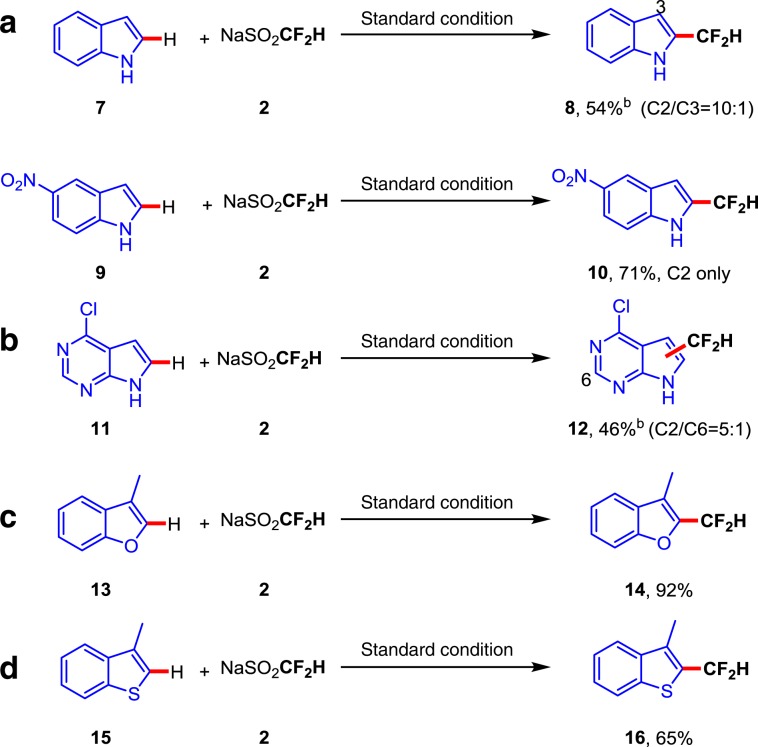
Site-selectivity study. **a** Regioselectivity for indoles. **b** Regioselectivity for 4-chloro-7H-pyrrolo[2,3-d] pyrimidine. **c** Regioselectivity for benzofuran. **d** Regioselectivity for thianaphthene. Reaction conditions: heterocycles (0.1 mmol), CF_2_HSO_2_Na **2** (0.4 mmol), rose bengal (5 mol%) in 1 mL DMSO under two 3 W green LEDs irradiation at room temperature. Isolated yields based on heterocycles. ^b^The minor regioisomeric position is labeled with the respective carbon atom number.

The substrate scope results of Figs. 2–4 show that most of the difluoromethylation occurs on the C2–H bond adjacent to the heteroatom. Some heterocycle substrates bearing more than one radical attacked carbon center were also examined (Fig. 5). As a result, the unsubstituted indole substrate **7** gave the major product at the C2 position in 54% yield with 10:1 regioselectivity. The 5-nitro-substituted indole substrate **9** can obtain a single regioselective difluoromethylation product **10** with 71% yield. When 4-chloro-7H-pyrrolo[2,3-d] pyrimidine was used as substrate, the reaction furnish the mixture of product **12** (C2/C6 = 5:1) in 46% yield. Notably, other electron-rich heteroarenes (benzofuran **13** and thianaphthene **15**), which have not been investigated as substrates in previous reported radical difluoromethylation reactions^48–50^, also exhibited good reaction efficiency, producing the difluoromethylation products **14** and **16** in 92% and 65% yield, respectively. Unfortunately, other types of heteroarenes, including phenanthroline, 1,3,5-triazine, and thiazone, are not ideal substrates in this difluoromethylation reaction (Supplementary Fig. 2).

### Synthetic applications

**Fig. 6:**
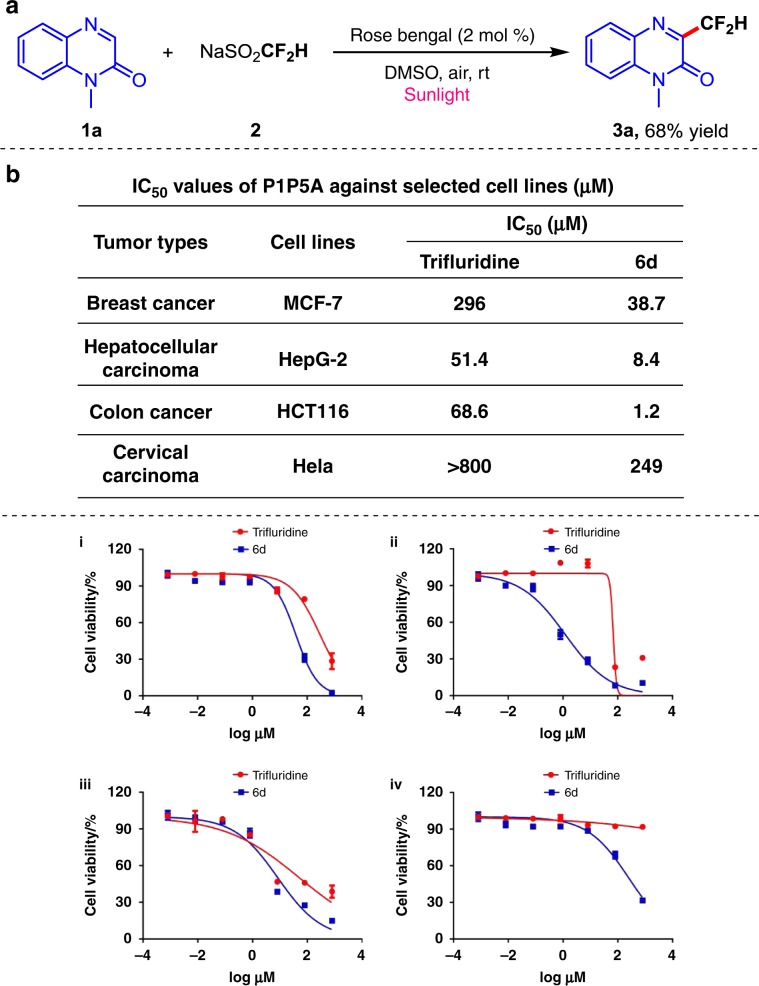
Synthetic applications. **a** The reactions were carried out with **4** (0.1 mmol), CF_2_HSO_2_Na **2** (0.2 mmol), rose bengal (2 mol%) in 1 mL DMSO under sunlight irradiation at room temperature. **b** Cytotoxicity of compounds. i MCF-7 (human breast adenocarcinoma cells), ii HepG-2 (human liver hepatocellular carcinoma cells), iii HCT116 (human colorectal cancer cells), and iv Hela (human cervical carcinoma cells) were treated with various concentrations of each compound for 72 h. Cell death was then measured by using a CCK-8 assays (*n* = 5, mean ± SD).

To evaluate the synthetic potential of this methodology, sunlight-driven experiment was performed. When the reaction was conducted under sunlight irradiation, the desired product **3a** was obtained in 68% yield (Fig. 6a). Furthermore, a slight reduction of yields with large scale preparation of **6d** and **6g** also prove the practicability of this difluoromethylation strategy (Fig. 4, yields in parentheses for **6d** and **6g**). We further explored the potential application of the synthesized difluoromethylated product in medicinal chemistry. The F_2_TDR **6d** has a similar structure to the trifluridine, which has been approved by FDA for the treatment of adult patients with metastatic colorectal cancer (for details, see https://www.drugbank.ca/drugs/DB00432). Therefore, we selected four tumor cell lines to evaluate the inhibitory activities of **6d**, and made the comparison of the result with trifluridine. As shown in Fig. 6b, **6d** higher tumor cell inhibitory capability than the trifluridine with relatively low IC_50_ values. Notably, the IC_50_ values of **6d** against HCT116 and HepG-2 cells reach low micromolar level, which are about 57- and 6-fold lower than that of trifluridine, respectively. The improvement of antitumor activity indicates the practicality of this difluoromethylation methodology and the potential in the field of discovery of active drug molecules.

### Mechanistic investigations

**Fig. 7:**
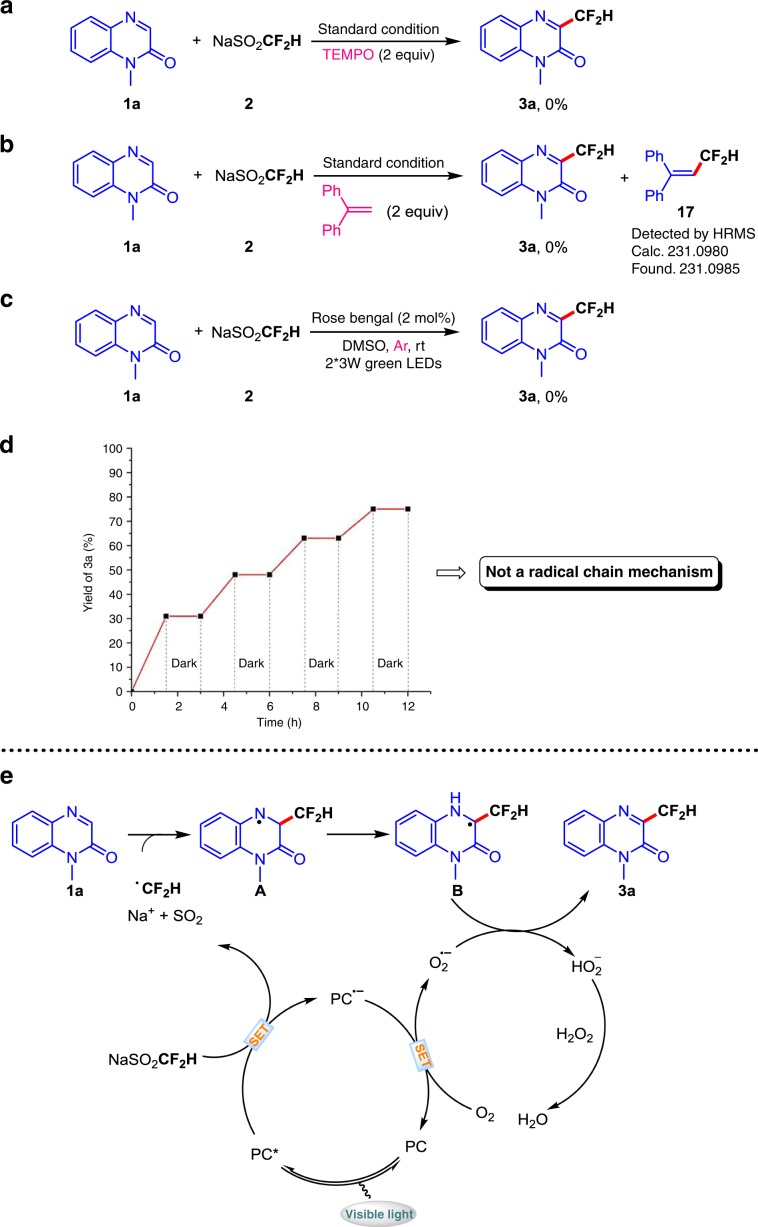
Mechanistic investigations. **a** Investigation on the effect of TEMPO. **b** Radical trapping with 1,1-diphenylethylene. **c** Investigation on the effect of oxygen. **d** Light/dark experiment. **e** Proposed reaction mechanism.

To gain insights into the current studied reaction, control experiments were conducted. When a radical scavenger, 2,2,6,6-tetramethyl-1-piperidinyloxy (TEMPO) or 1,1-diphenylethylene (Fig. 7a, b) was existing in the mixture containing **1a** and CF_2_HSO_2_Na, the reaction was completely suppressed, while the radical intermediate was detected by ESI-HRMS (Supplementary Fig. 5), indicating the existence of CF_2_H radical. When the reaction was carried out under inert atmosphere (Fig. 7c), the formation of **3a** was completely inhibited, revealing that oxygen is crucial for the reaction. In situ ^1^H NMR experiment demonstrated that hydrogen peroxide (H_2_O_2_) does not form after irradiation of the reaction mixture in DMSO-*d*_6_ for 12 h under the optimized reaction condition. In addition, the observed water peak (H_2_O) growth indicating that the oxygen was eventually converted to H_2_O rather than H_2_O_2_ (Supplementary Figs. 6 and 7). The generated H_2_O_2_ could participate in the catalytic cycle and ultimately converted to H_2_O as the byproduct^61^. These experimental results illustrated an available radical pathway. Moreover, we also conducted the light/dark experiment. As shown in Fig. 7, the desired product **3a** formed only under continuous irradiation, which ruled out the possibility of a radical chain propagation. On the basis of our experimental observations and previous studies^59^, a possible mechanism was proposed (Fig. 7e). Upon absorption of visible light, the photocatalyst RB is excited into RB* (*E*_red_ = 0.99 V vs SCE) and a single electron is transferred from CF_2_HSO_2_Na^62^ (*E* = 0.59 V vs SCE) to RB*, which affords CF_2_H radical and generates an RB^•−^ radical anion. The photoredox cycle is completed by the molecular oxygen oxidation of RB^•−^, giving RB and O_2_^•−^. After that, addition of CF_2_H radical to **1a** occurs, leading to intermediate **A**, which undergoes a 1,2-H shift to generate carbon radical intermediate **B**. The intermediate **B** loses a hydrogen atom to O_2_^•−^ to furnish the desired product **3a**.

## Discussion

In summary, we have achieved a visible-light triggered direct C–H difluoromethylation of heterocycles by using commercially available and inexpensive sodium difluoromethane sulfonate as CF_2_H radical source. The process is  under mild conditions using O_2_ as a green oxidant and without using  metal additive. Furthermore, we also use this highly efficient methodology for direct difluoromethylation of some nitrogen-containing biological and pharmaceutical active molecules. In addition, the bioactivity evaluation of a representative difluoromethylation product 2′-deoxy-5-difluoromethyluridine (**6d**) exhibited promising activity against cancer cell lines. We expect this simple protocol to be of broad utility for the development of new drugs. Further synthetic applications and bioactivity tests are ongoing.

## Methods

### Procedure for difluoromethylation of quinoxalin-2(1*H*)-ones

To a 10 mL Schlenk tube equipped with a magnetic stir bar added quinoxalin-2(1*H*)-ones **1** (0.2 mmol), CF_2_HSO_2_Na **2** (0.4 mmol), and RB (0.004 mmol, 2 mol.%) in DMSO (1.0 mL). Then the mixture was stirred and irradiated by two 3 W green LEDs at room temperature for 12 h. The residue was added water (10 mL) and extracted with ethyl acetate (5 mL × 3). The combined organic phase was dried over Na_2_SO_4_. The resulting crude residue was purified via column chromatography on silica gel to afford desired products.

### Procedure for difluoromethylation of other heterocycles

To a 10 mL Schlenk tube equipped with a magnetic stir bar added heteroarenes **4** (0.1 mmol), CF_2_HSO_2_Na **2** (0.4 mmol), and RB (0.002–0.005 mmol, 2–5 mol.%) in DMSO (1.0 mL). Then the mixture was stirred and irradiated by two 3 W green LEDs at room temperature for 24 h. The residue was added water (10 mL) and extracted with ethyl acetate (5 mL × 3). The combined organic phase was dried over Na_2_SO_4_. The resulting crude residue was purified via column chromatography on silica gel to afford desired products.

## Supplementary information

Supplementary Information

## Data Availability

The authors declare that the data supporting the findings of this study are available within the article and its Supplementary Information files. Extra data are available from the author upon reasonable request. The X-ray crystallographic coordinates for structures of **3a** reported in this article have been deposited at the Cambridge Crystallographic Data Center as CCDC 1920406. These data can be obtained free of charge from The Cambridge Crystallographic Data Centre via http://www.ccdc.cam.ac.uk/data_request/cif.
