# Supplementary material for: Direct C–H difluoromethylation of heterocycles via organic photoredox catalysis
Source: Nat Commun. 2020 Jan 31;11:638. doi: 10.1038/s41467-020-14494-8 (PMC6994692; doi:10.1038/s41467-020-14494-8)
Supplement: Supplementary file 1 — Supplementary Information [file 41467_2020_14494_MOESM1_ESM.pdf]

Supplementary Information

Direct C-H Difluoromethylation of Heterocycles via Organic Photoredox Catalysis

Zhang *et al*.

Supplementary Methods

General information

Commercial reagents were used as received, unless otherwise stated. ^{1}H and ^{13}C NMR were recorded on a Bruker-DPX 400 spectrometer. Chemical shifts are reported in ppm from tetramethylsilane with the solvent resonance as the internal standard. The following abbreviations were used to designate chemical shift mutiplicities: s = singlet, d = doublet, t = triplet, q = quartet, h = heptet, m = multiplet, br = broad. All first-order splitting patterns were assigned on the basis of the appearance of the multiplet. Splitting patterns that could not be easily interpreted are designated as multiplet (m) or broad (br). ^{19}F NMR were recorded on a Varian NMR 400 spectrometer. Mass spectra were obtained using electrospray ionization (ESI) mass spectrometer. Substrates **1** was synthesized according to the literature method.[5] sodium difluoromethane sulfonate (CF_2HSO_2Na) was purchased from *J&K* without further purification.

Experimental procedure

General procedure for synthesis of quinoxalin-2(1*H*)-ones

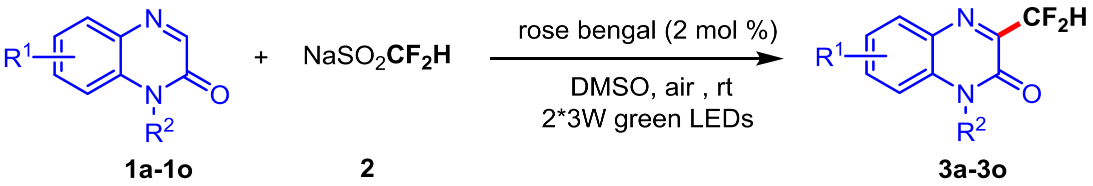

To a 10 mL Schlenk tube equipped with a magnetic stir bar, added quinoxalin-2(1*H*)-ones **1** (0.2 mmol), CF_2HSO_2Na **2** (0.4 mmol) and rose bengal (0.004 mmol, 2 mol%) in DMSO (1.0 mL). Then the mixture was stirred and irradiated by the two 3W green LEDs at room temperature for 12 h. The residue was added water (10 mL) and extracted with ethyl acetate (5 mL × 3). The combined organic phase was dried over Na_2SO_4. The resulting crude residue was purified *via* column chromatography on silica gel to afford the desired products.

General procedure for synthesis of heteroarenes

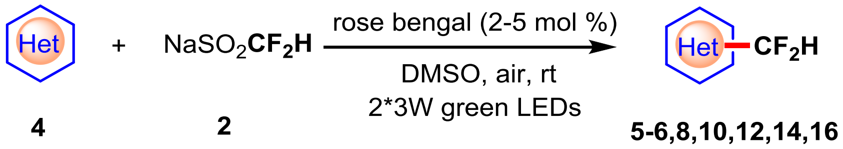

To a 10 mL Schlenk tube equipped with a magnetic stir bar, added heteroarenes **4** (0.1 mmol), CF_2HSO_2Na **2** (0.4 mmol) and rose bengal (0.002-0.005 mmol, 2-5 mol%) in DMSO (1.0 mL). Then the mixture was stirred and irradiated by the two 3W green LEDs at room temperature for 24 h. The residue was added water (10 mL) and extracted with ethyl acetate (5 mL × 3). The combined organic phase was dried over Na_2SO_4. The resulting crude residue was purified *via* column chromatography on silica gel to afford desired products.

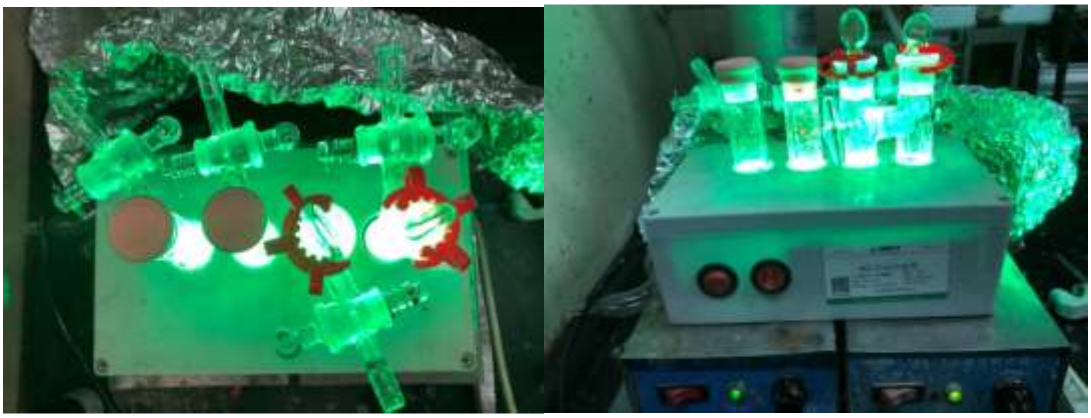

Supplementary Figure 1. Reaction set-up

Analytical data of compounds quinoxalin-2(1*H*)-ones

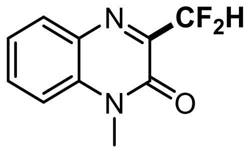

3a: yellow solid, 30.3 mg, 72% yield; R_f=0.6 (30% EtOAc/petroleum ether); ^{1}H NMR (400 MHz, $CDCl_3$) δ 7.97-7.93 (m, 1H), 7.68-7.64 (m, 1H), 7.42-7.34 (m, 2H), 6.93 (t, *J*=53.6 Hz, 1H), 3.70 (s, 3H); ^{13}C NMR (101 MHz, $CDCl_3$) δ 153.2, 148.51 (t, *J* = 22.6 Hz), 134.0, 132.7, 131.8, 131.3, 124.4, 114.0, 110.1 (t, *J* = 241.6 Hz), 29.0; ^{19}F NMR (376 MHz, $CDCl_3$) δ -125.1 (d, *J* = 58.8 Hz, 2F); HRMS (ESI) calcd for $C_{10}H_9F_2N_2O$ [M+H$^+$]: 211.0677, found: 211.0678.

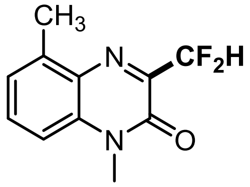

3b: yellow solid, 30.5 mg, 68% yield; R_f=0.6 (30% EtOAc/petroleum ether); ^{1}H NMR (400 MHz, $CDCl_3$) δ 7.55 (t, *J* = 8.0 Hz, 1H), 7.28-7.25 (m, 1H), 7.20 (d, *J* = 8.4 Hz, 1H), 6.92 (t, *J* = 53.8 Hz, 1H), 3.72 (s, 3H), 2.71 (s, 3H); ^{13}C NMR (101 MHz, $CDCl_3$) δ 153.1, 146.6 (t, *J* = 22.8 Hz), 140.7, 132.4 (t, *J* = 187.7 Hz), 125.6, 113.1, 111.7, 110.7, 108.3, 29.1, 17.4; ^{19}F NMR (376 MHz, $CDCl_3$) δ -123.6 (d, *J* = 54.1 Hz, 2F); HRMS (ESI) calcd for $C_{11}H_{10}F_2N_2NaO$ [M+Na$^+$]: 247.0653, found: 247.0658.

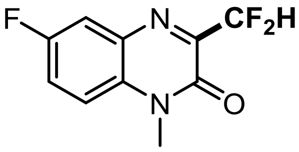

3c: orange solid, 27.4mg, 60% yield; R_f=0.4 (30% EtOAc/petroleum ether); ^{1}H NMR (400 MHz, $CDCl_3$) δ 7.73-7.69 (m, 1H), 7.50-7.45 (m, 1H), 7.41-7.37 (m, 1H), 6.97 (t, *J* = 53.6 Hz, 1H), 3.76 (s, 3H); ^{13}C NMR (101 MHz, $CDCl_3$) δ 158.9 (d, *J* = 245.7 Hz), 152.9, 150.0 (t, *J* = 22.8 Hz), 132.38 (d, *J* = 11.2 Hz), 130.8, 120.7(d, *J* = 24.1 Hz), 116.7(d, *J* = 22.4 Hz), 115.3(d, *J* = 8.7 Hz), 109.9 (t, *J* = 242.0 Hz), 29.3; ^{19}F NMR (376 MHz, $CDCl_3$) δ -117.2 (s, 1F), -124.6 (d, *J* = 53.5 Hz, 2F); HRMS (ESI) calcd

for $C_{10}H_7F_3N_2NaO$ [M+Na$^+$]: 251.0403, found: 251.0406.

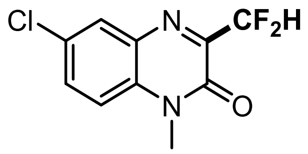

3d: yellow solid, 31.4mg, 70% yield; R_f=0.4 (20% EtOAc/petroleum ether); ^{1}H NMR (400 MHz, $CDCl_3$) δ 7.97 (d, *J* = 2.4 Hz, 1H), 7.63 (dd, *J* = 9.0, 2.4 Hz, 1H), 7.32 (d, *J* = 9.0 Hz, 1H), 6.92 (t, *J* = 53.6 Hz, 1H), 3.72 (s, 3H); ^{13}C NMR (101 MHz, $CDCl_3$) δ152.8, 151.4, 149.9 (t, *J* = 22.8 Hz), 132.7, 132.3, 131.1, 130.6, 129.8, 115.2, 109.9 (t, *J* = 242.5 Hz), 29.2; ^{19}F NMR (376 MHz, $CDCl_3$) δ -124.6 (d, *J* = 53.6 Hz, 2F); HRMS (ESI) calcd for $C_{10}H_7ClF_2N_2NaO$ [M+Na$^+$]: 267.0111, found: 267.0107.

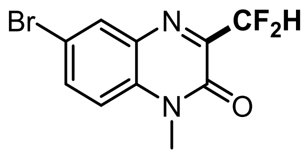

3e: yellow solid, 47.4mg, 82% yield; R_f=0.2 (20% EtOAc/petroleum ether); ^{1}H NMR (400 MHz, $CDCl_3$) δ 8.14 (d, *J* = 2.2 Hz, 1H), 7.77 (dd, *J* = 8.9, 2.2 Hz, 1H), 7.28-7.26 (m, 1H), 6.93 (t, *J* = 53.6 Hz, 1H), 3.72 (s, 3H); ^{13}C NMR (101 MHz, $CDCl_3$) δ 152.8, 149.8 (t, *J* = 22.8 Hz), 135.4, 133.7, 133.2, 132.6, 117.0, 115.4, 112.3, 109.9 (t, *J* = 243.0 Hz), 29.2; ^{19}F NMR (376 MHz, $CDCl_3$) δ -124.6 -124.55 (d, *J* = 53.6 Hz, 2F); HRMS (ESI) calcd for $C_{10}H_7BrF_2N_2NaO$ [M+Na$^+$]: 310.9602, found: 310.9605.

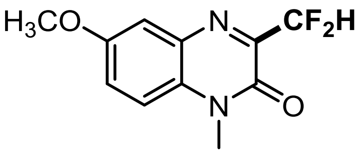

3f: white solid, 39.9mg, 83% yield; R_f=0.5 (30% EtOAc/petroleum ether); ^{1}H NMR (400 MHz, $CDCl_3$) δ 7.45 (s, 1H), 7.30 (d, *J* = 1.6 Hz, 2H), 6.98 (t, *J* = 53.8 Hz, 1H), 3.89 (s, 3H), 3.72 (s, 3H); ^{13}C NMR (101 MHz, $CDCl_3$) δ 156.4, 153.0, 149.0 (t, *J* = 22.1 Hz), 132.7, 128.4, 122.5, 114.9, 112.1, 109.9 (t, *J* = 242.4 Hz), 55.9, 29.1; ^{19}F NMR (376 MHz, $CDCl_3$) δ -124.4 (d, *J* = 55.5Hz, 2F); HRMS (ESI) calcd for $C_{11}H_{10}F_2N_2NaO_2$ [M+Na$^+$]: 263.0603, found: 263.0606.

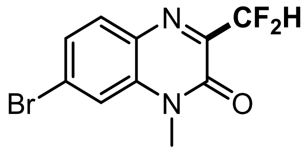

3g: yellow solid, 34.7mg, 60% yield; R_f=0.4 (20% EtOAc/petroleum ether); ^{1}H NMR (400 MHz, $CDCl_3$) δ 7.83 (d, *J* = 8.5 Hz, 1H), 7.54-7.51 (m, 2H), 6.91 (t, *J* = 53.6 Hz, 1H), 3.70 (s, 3H); ^{13}C NMR (101 MHz, $CDCl_3$) δ 152.9, 148.8 (t, *J* = 23.0 Hz), 134.9, 132.6, 130.7, 127.8, 127.3, 117.1, 109.9 (t, *J* = 241.9 Hz), 29.1; ^{19}F NMR (376 MHz, $CDCl_3$) δ -124.4 (d, *J* = 53.6 Hz, 2F); HRMS (ESI) calcd for $C_{10}H_7BrF_2N_2NaO$ [M+Na$^+$]: 310.9602, found: 310.9606.

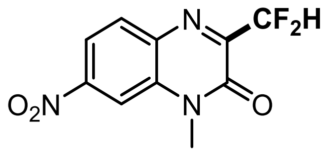

3h: yellow solid, 20.4mg, 40% yield; R_f=0.5 (30% EtOAc/petroleum ether); ^{1}H NMR (400 MHz, $CDCl_3$) δ 8.28-8.16 (m, 3H), 6.95 (t, *J* = 53.3 Hz, 1H), 3.81 (s, 3H); ^{13}C NMR (101 MHz, $CDCl_3$) δ 152.6, 152.2 (t, *J* = 22.9 Hz), 149.3, 134.9, 134.5, 132.7, 118.7, 109.9, 109.5 (t, *J* = 244.0 Hz), 29.6; ^{19}F NMR (376 MHz, $CDCl_3$) δ -125.0 (d, *J* = 53.5 Hz, 2F); HRMS (ESI) calcd for $C_{10}H_7F_2N_3NaO_3$ [M+Na$^+$]: 278.0348, found: 278.0350.

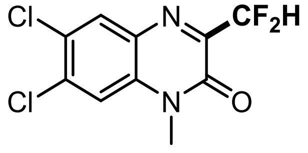

3i: yellow solid, 41.9mg, 75% yield; R_f=0.6 (30% EtOAc/petroleum ether); ^{1}H NMR (400 MHz, $CDCl_3$) δ 8.24 (s, 1H), 8.00 (s, 1H), 7.09 (t, *J* = 53.0 Hz, 1H), 3.63 (s, 3H); ^{13}C NMR (101 MHz, DMSO-d_6) δ 152.3, 149.8 (t, *J* = 21.6 Hz), 134.9, 134.0, 130.9, 130.4, 126.1, 117.0, 110.1 (t, *J* = 239.9 Hz), 29.3; ^{19}F NMR (376 MHz, DMSO-d_6) δ -119.9 (d, *J* = 52.9Hz, 2F); HRMS (ESI) calcd for $C_{10}H_6Cl_2F_2N_2NaO$ [M+Na$^+$]: 300.9717, found: 300.9720.

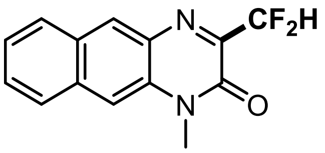

3j: yellow solid, 21.9mg, 42% yield; R_f=0.3 (20% EtOAc/petroleum ether); ^{1}H NMR (400 MHz, $CDCl_3$) δ 8.52 (s, 1H), 7.97 (dd, *J* = 28.4, 8.3 Hz, 2H), 7.66-7.62 (m, 2H), 7.54 (t, *J* = 7.6 Hz, 1H), 6.99 (t, *J* = 53.7 Hz, 1H), 3.79 (s, 3H); ^{13}C NMR (101 MHz, $CDCl_3$) δ 153.0, 149.2 (t, *J* = 22.3 Hz), 134.8, 131.5, 131.5, 131.0, 129.8, 129.2, 128.9, 127.3, 125.9, 110.5, 110.1 (t, *J* = 242.8 Hz), 28.9; ^{19}F NMR (376 MHz, $CDCl_3$) δ -124.3 (d, *J* = 53.9 Hz, 2F); HRMS (ESI) calcd for $C_{14}H_{10}F_2N_2NaO$ [M+Na$^+$]: 283.0653, found: 283.0653.

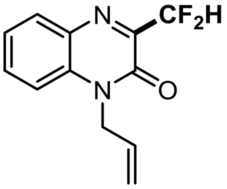

3k: white solid,16.1mg, 34% yield; R_f=0.6 (20% EtOAc/petroleum ether); ^{1}H NMR (400 MHz, $CDCl_3$) δ 8.01 (dd, *J* = 8.1, 1.5 Hz, 1H), 7.67-7.63 (m, 1H), 7.44-7.40 (m, 1H), 7.36 (dd, *J* = 8.5, 1.1 Hz, 1H), 6.98 (t, *J* = 53.7 Hz, 1H), 5.98-5.89 (m, 1H), 5.31 (d, *J* = 8.7 Hz, 1H), 5.21 (d, *J* = 17.2 Hz, 1H), 4.95-4.92 (m, 1H); ^{13}C NMR (101 MHz, $CDCl_3$) δ 152.8, 148.7 (t, *J* = 22.3 Hz), 133.3, 132.6, 132.1, 131.5, 130.0, 124.4, 118.8, 114.5, 110.0 (t, *J* = 241.6 Hz), 44.5; ^{19}F NMR (376 MHz, $CDCl_3$) δ -124.2 (d, *J* = 53.6 Hz, 2F); HRMS (ESI) calcd for $C_{12}H_{10}F_2N_2NaO$ [M+Na$^+$]: 259.0653, found: 259.0658.

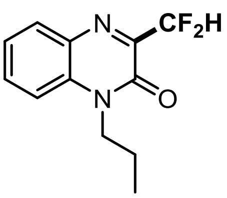

3l: yellow solid, 26.7mg, 56% yield; R_f=0.5 (20% EtOAc/petroleum ether); ^{1}H NMR (400 MHz, $CDCl_3$) δ 8.00 (dd, *J* = 8.0, 1.5 Hz, 1H), 7.69-7.65 (m, 1H), 7.43-7.36 (m, 2H), 6.97 (t, *J* = 53.7 Hz, 1H), 4.26-4.22 (m, 2H), 1.86-1.77 (m, 2H), 1.06 (t, *J* = 7.4 Hz, 3H); ^{13}C NMR (101 MHz, $CDCl_3$) δ 153.0, 148.6 (t, *J* = 22.4 Hz), 133.3, 132.5,

132.2, 131.7, 124.1, 114.0, 110.0 (t, *J* = 22.4 Hz) 43.8, 20.7, 11.4; ^{19}F NMR (376 MHz, $CDCl_3$) δ -129.0 (d, *J* = 53.6 Hz, 2F); HRMS (ESI) calcd for $C_{12}H_{12}F_2N_2NaO$ [M+Na$^+$]: 261.0810, found: 261.0815.

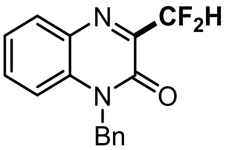

3m: yellow solid, 29.8mg, 52% yield; R_f=0.6 (20% EtOAc/petroleum ether); ^{1}H NMR (400 MHz, $CDCl_3$) δ 8.01 (d, *J* = 8.0 Hz, 1H), 7.56 (t, *J* = 7.9 Hz, 1H), 7.40-7.25 (m, 7H), 7.03 (t, *J* = 53.7 Hz, 1H), 5.52 (s, 2H); ^{13}C NMR (101 MHz, $CDCl_3$) δ 153.4, 148.8 (t, *J* = 22.7 Hz), 134.5, 133.4, 132.6, 132.2, 131.6, 129.1, 128.0, 126.9, 124.5, 114.7, 110.0 (t, *J* = 241.7 Hz); ^{19}F NMR (376 MHz, $CDCl_3$) δ -124.1 (d, *J* = 53.6 Hz, 2F); HRMS (ESI) calcd for $C_{16}H_{12}F_2N_2NaO$ [M+Na$^+$]: 309.0810, found: 309.0815.

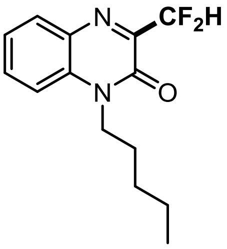

3n: yellow solid, 38.9mg, 73% yield; R_f=0.6 (20% EtOAc/petroleum ether); ^{1}H NMR (400 MHz, $CDCl_3$) δ 8.00 (d, *J* = 7.7 Hz, 1H), 7.69-7.65 (m, 1H), 7.43-7.36 (m, 2H), 6.97 (t, *J* = 53.7 Hz, 1H), 4.28-4.24 (m, 2H), 1.81-1.73 (m, 2H), 1.49-1.35 (m, 4H), 0.93 (t, *J* = 6.9 Hz, 3H); ^{13}C NMR (101 MHz, $CDCl_3$) δ 153.0, 148.5 (d, *J* = 22.5 Hz), 133.3, 132.5, 132.2, 131.7, 124.2, 113.9, 110.0 (t, *J* = 241.5 Hz), 42.4, 29.0, 26.9, 22.3, 13.9; ^{19}F NMR (376 MHz, $CDCl_3$) δ -124.3 (d, *J* = 53.7 Hz, 2F); HRMS (ESI) calcd for $C_{14}H_{16}F_2N_2NaO$ [M+Na$^+$]: 289.1123, found: 289.1126.

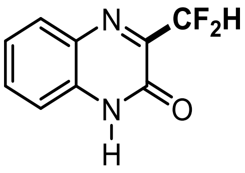

3o: white solid, 34.1mg, 87% yield; R_f=0.4 (50% EtOAc/petroleum ether); ^{1}H NMR

(400 MHz, DMSO-d_6) δ 12.90 (s, 1H), 7.93-7.85 (m, 1H), 7.71-7.64 (m, 1H), 7.44-7.34 (m, 2H), 7.08 (td, J = 53.1, 16.2 Hz, 1H); ^{13}C NMR (101 MHz, DMSO-d_6) δ 153.7, 150.2 (t, J = 22.2 Hz), 133.3, 132.8, 131.2, 130.0, 124.4, 116.3, 110.8 (t, J = 238.4 Hz); ^{19}F NMR (376 MHz, DMSO-d_6) δ -124.3 (d, J = 53.5 Hz, 2F); HRMS (ESI) calcd for $C_9H_5F_2N_2O$ [M-H$^+$]: 195.0370, found: 195.0373.

Analytical data of heteroarenes

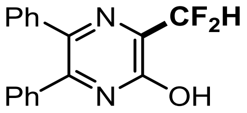

5a: yellow solid, 24.5mg, 82% yield; R_f=0.5 (30% EtOAc/petroleum ether); ^{1}H NMR (400 MHz, $CDCl_3$) δ 12.93 (s, 1H), 7.47-7.42 (m, 1H), 7.39-7.38 (m, 4H), 7.32-7.29 (m, 2H), 7.27-7.23 (m, 3H), 6.77 (t, J = 53.7 Hz, 1H); ^{13}C NMR (101 MHz, $CDCl_3$) δ 155.3, 144.7 (t, J = 24.9 Hz), 139.6, 136.1, 133.7, 131.4, 130.7, 129.5, 129.5, 128.9, 128.3, 128.1, 110.9 (t, J = 240.7 Hz); ^{19}F NMR (376 MHz, $CDCl_3$) δ -122.3 (d, J = 53.8 Hz, 2F); HRMS (ESI) calcd for $C_{17}H_{11}F_2N_2O$ [M-H$^+$]: 297.0839, found: 297.0842.

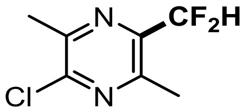

5b: colorless oil, 12.5mg, 65% yield; R_f=0.7 (5% EtOAc/petroleum ether); ^{1}H NMR (400 MHz, $CDCl_3$) δ 6.67 (t, J = 54.1 Hz, 1H), 2.69 (s, 3H), 2.63 (s, 3H); Other spectral data of **5b** were consistent with previous reported data.[3]

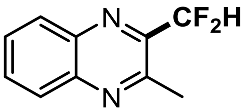

5c: white solid, 12.1mg, 62% yield; R_f=0.8 (20% EtOAc/petroleum ether); ^{1}H NMR (400 MHz, $CDCl_3$) δ 8.10-8.06 (m, 2H), 7.85-7.75 (m, 2H), 6.83 (t, J = 54.3 Hz, 1H), 2.93 (s, 3H); Other spectral data of **5c** were consistent with previous reported data.[1]

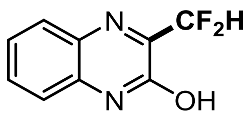

5d: white solid, 17.6mg, 90% yield; R_f=0.5 (50% EtOAc/petroleum ether); ^{1}H NMR (400 MHz, DMSO-d_6) δ 12.9 (s, 1H), 7.89 (dd, J = 8.1, 1.3 Hz, 1H), 7.66 (td, J = 7.7, 1.4 Hz, 1H), 7.41-7.36 (m, 2H), 7.06 (t, J = 53.3 Hz, 1H); ^{13}C NMR (101 MHz, DMSO-d_6) δ 153.2, 149.7 (t, J = 21.4 Hz), 132.8, 132.3, 130.7, 129.4, 123.9, 115.7, 110.3 (t, J = 239.3 Hz); ^{19}F NMR (376 MHz, DMSO-d_6) δ -119.5 (d, J = 53.1 Hz, 2F); HRMS (ESI) calcd for $C_9H_5F_2N_2O$ [M-H$^+$]: 195.0370, found: 195.0370.

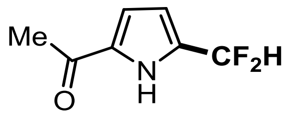

5e: white solid, 9.2mg, 61% yield; R_f=0.7 (35% EtOAc/petroleum ether); ^{1}H NMR (400 MHz, $CDCl_3$) δ 9.71 (s, 1H), 6.87 (s, 1H), 6.71 (t, J = 55.3 Hz, 1H), 6.48-6.45 (m, 1H), 2.47 (s, 3H); Other spectral data of **5e** were consistent with previous reported data.[1]

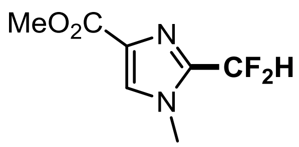

5f: white solid, 12.3mg, 64% yield; R_f=0.2 (50% EtOAc/petroleum ether); ^{1}H NMR (400 MHz, $CDCl_3$) δ 7.56 (t, J = 52.7 Hz, 1H), 7.51 (s, 1H), 3.92 (s, 3H), 3.86 (s, 3H); ^{13}C NMR (101 MHz, $CDCl_3$) δ 162.7, 140.3, 133.7, 129.4 (t, J = 23.0 Hz), 108.7 (t, J = 233.9 Hz), 52.2, 33.6; ^{19}F NMR (376 MHz, $CDCl_3$) δ -114.4 (d, J = 51.8 Hz, 2F); HRMS (ESI) calcd for $C_{12}H_{12}F_2N_2NaO$ [M+Na$^+$]: 261.0810, found: 261.0815.

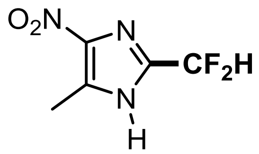

5g: white solid, 14.4mg, 81% yield; R_f=0.3 (35% EtOAc/petroleum ether); ^{1}H NMR (400 MHz, DMSO-d_6) δ 13.96 (s, 1H), 7.46 (t, J = 53.1 Hz, 1H), 2.39 (s, 3H); ^{13}C NMR (101 MHz, DMSO-d_6) δ 145.9, 130.1, 124.0, 108.6 (t, J = 235.6 Hz), 14.17; ^{19}F

NMR (376 MHz, DMSO-d_6) δ -113.2 (s, 2F); HRMS (ESI) calcd for $C_5H_5F_2N_3NaO_2$ [M+Na$^+$]: 200.0242, found: 200.0245.

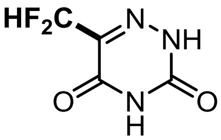

5h: colorless oil, 11.9 mg, 73% yield; R_f=0.2 (50% EtOAc/petroleum ether); ^{1}H NMR (400 MHz, DMSO-d_6) δ 12.75 (s, 1H), 12.31 (s, 1H), 6.77 (t, J = 52.7 Hz, 1H); ^{13}C NMR (101 MHz, DMSO-d_6) δ 155.1, 149.0, 135.9 (t, J = 23.7 Hz), 110.7 (t, J = 237.9 Hz); ^{19}F NMR (376 MHz, DMSO-d_6) δ -117.4 (d, J = 52.7 Hz, 2F); HRMS (ESI) calcd for $C_4H_2F_2N_3O_2$ [M-H$^+$]: 162.0115, found: 162.0124.

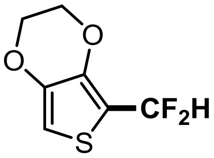

5i: colorless oil, 7.8mg, 41% yield; R_f=0.3 (3% EtOAc/petroleum ether); ^{1}H NMR (400 MHz, $CDCl_3$) δ 6.88 (t, J = 55.3, Hz), 6.50 (s, 1H), 4.30-4.23 (m, 4H); Other spectral data of **5i** were consistent with previous reported data.[4]

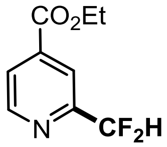

5j: colorless oil, 8.5mg, 42% yield; R_f = 0.4 (25% dichloromethane/ petroleum ether); ^{1}H NMR (400 MHz, $CDCl_3$) δ 8.79 (d, J = 5.0 Hz, 1H), 8.17 (s, 1H), 7.96 (d, J = 5.0 Hz, 1H), 6.68 (t, J = 55.2 Hz, 2H), 1.41 (t, J = 7.1 Hz, 3H); Other spectral data of **5j** were consistent with previous reported data.[1]

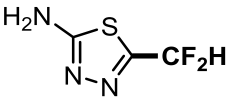

5k: white solid, 5.6mg, 34% yield; R_f=0.5 (80% EtOAc/petroleum ether); ^{1}H NMR (400 MHz, CD_3OD) δ 6.94 (t, J = 53.7 Hz, 1H); Other spectral data of **5k** were consistent with previous reported data.[1]

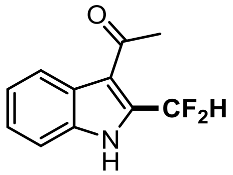

5l: white solid, 15.9mg, 38% yield; R_f=0.7 (30% EtOAc/petroleum ether); ^{1}H NMR (400 MHz, DMSO-d_6) δ 12.82 (s, 1H), 8.04 (d, J = 7.7 Hz, 1H), 7.73-7.46 (m, 2H), 7.59 (t, J = 53.6 Hz, 1H), 7.56 (d, J = 7.7 Hz, 1H) 2.67 (s, 3H); ^{13}C NMR (101 MHz, DMSO-d_6) δ 193.6, 135.4, 134.7 (t, J = 22.9 Hz), 124.8, 123.9, 122.4, 121.3, 115.8 (t, J = 5.9 Hz), 113.0, 109.7 (t, J = 235.2 Hz), 30.9; ^{19}F NMR (376 MHz, DMSO-d_6) δ -108.1 (d, J = 56.3 Hz, 2F); HRMS (ESI) calcd for $C_{11}H_{10}F_2NO$ [M+H$^+$]: 210.0725, found: 210.0728.

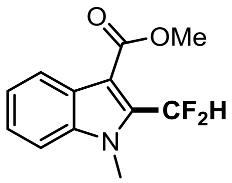

5m: white solid, 16.3mg, 68% yield; R_f=0.3 (3% EtOAc/petroleum ether); ^{1}H NMR (400 MHz, $CDCl_3$) δ 8.19 (d, J = 8.0 Hz, 1H), 7.93 (t, J = 52.0 Hz, 1H), 7.41-7.30 (m, 3H), 3.98 (s, 6H); ^{13}C NMR (101 MHz, $CDCl_3$) δ 165.0, 137.5, 135.3 (t, J = 21.6 Hz), 125.2, 124.8, 122.9, 122.6, 109.9, 109.6 (t, J = 235.0 Hz), 108.0 (t, J = 6.4 Hz), 51.5, 31.8; ^{19}F NMR (376 MHz, DMSO-d_6) δ -113.8 (d, J = 52.9 Hz, 2F); HRMS (ESI) calcd for $C_{12}H_{11}F_2NNaO_2$ [M+Na$^+$]: 262.0650, found: 262.0653.

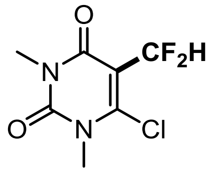

5n: white solid, 11.7mg, 52% yield; R_f=0.6 (30% EtOAc/petroleum ether); ^{1}H NMR (400 MHz, $CDCl_3$) δ 6.94 (t, J = 53.2 Hz, 1H), 3.65 (s, 3H), 3.37 (s, 3H). Other spectral data of **5n** were consistent with previous reported data.[2]

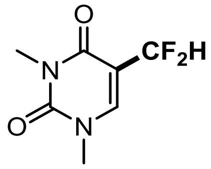

5o: white solid, 8.0mg, 42% yield; R_f=0.2 (10% EtOAc/petroleum ether); ^{1}H NMR (400 MHz, $CDCl_3$) δ 7.56 (s, 1H), 6.68 (t, J = 55.2 Hz, 1H), 3.47 (s, 3H), 3.36 (s, 3H). Other spectral data of **5n** were consistent with previous reported data.[2]

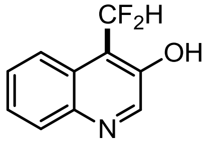

5p: white solid, 10.0mg, 51% yield; R_f=0.5 (30% EtOAc/petroleum ether); ^{1}H NMR (400 MHz, DMSO-d_6) δ 10.60 (s, 1H), 8.67 (s, 1H), 8.01 (d, J = 8.2 Hz, 1H), 7.82 (t, J = 55.2 Hz, 1H), 7.78 (d, J = 7.3 Hz, 1H), 7.65-7.61 (m, 2H); ^{13}C NMR (101 MHz, DMSO-d_6) δ151.5, 144.3, 139.1 (t, J = 5.0 Hz), 130.4 (t, J = 21.1 Hz), 129.6, 129.1, 126.3, 122.6 (t, J = 6.5 Hz), 115.7, 112.7 (t, J = 234.6 Hz); ^{19}F NMR (376 MHz, DMSO-d_6) δ -114.1 (d, J = 56.8 Hz, 2F); HRMS (ESI) calcd for $C_{10}H_8F_2NO$ [M+Na$^+$]: 196.0568, found: 196.0573.

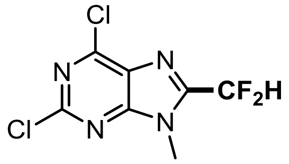

5q: white solid, 17.6mg, 70% yield; R_f=0.8 (20% EtOAc/petroleum ether); ^{1}H NMR (400 MHz, $CDCl_3$) δ 6.92 (t, J = 51.9 Hz, 1H), 4.04 (s, 3H); ^{13}C NMR (101 MHz, $CDCl_3$) δ 154.6, 154.3, 153.3, 147.8 (t, J = 27.8 Hz), 129.2, 110.2 (t, J = 240.2 Hz), 30.4; ^{19}F NMR (376 MHz, $CDCl_3$) δ -116.0 (d, J = 51.9 Hz, 2F); HRMS (ESI) calcd for $C_7H_5Cl_2F_2N_4$ [M+H$^+$]: 252.9854, found: 252.9856.

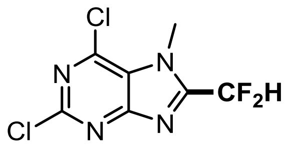

5r: white solid, 18.5mg, 73% yield; R_f=0.4 (20% EtOAc/petroleum ether); ^{1}H NMR (400 MHz, $CDCl_3$) δ 7.00 (t, *J* = 51.9 Hz, 1H), 4.30 (s, 3H); ^{13}C NMR (101 MHz, $CDCl_3$) δ 161.0, 153.9, 151.2 (t, *J* = 27.7 Hz), 145.4, 123.7, 110.1 (t, *J* = 240.7 Hz); 33.6; ^{19}F NMR (376 MHz, $CDCl_3$) δ -115.9 (d, *J* = 51.9 Hz, 2F); HRMS (ESI) calcd for $C_7H_5Cl_2F_2N_4$ [M+H$^+$]: 252.9854, found: 252.9857.

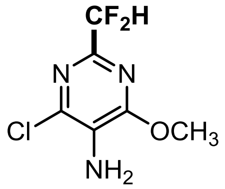

5s: colorless oil, 11.3mg, 54% yield; R_f=0.6 (2% EtOAc/petroleum ether); ^{1}H NMR (400 MHz, DMSO-d_6) δ 6.70 (t, *J* = 54.3 Hz, 1H), 5.97 (s, 2H), 4.01 (s, 1H); ^{13}C NMR (101 MHz, DMSO-d_6) δ 157.9, 144.4 (t, *J* = 25.6 Hz), 137.0, 129.5, 112.4 (t, *J* = 239.4 Hz), 55.2; ^{19}F NMR (376 MHz, DMSO-d_6) δ -115.6 (d, *J* = 54.6 Hz, 2F); HRMS (ESI) calcd for $C_6H_7ClF_2N_3O$ [M+H$^+$]: 210.0240, found: 210.0242.

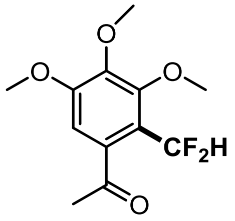

5t: colorless oil, 17.0mg, 65% yield; R_f=0.5 (10% EtOAc/petroleum ether); ^{1}H NMR (400 MHz, $CDCl_3$) δ 6.98 (t, *J* = 54.8 Hz, 1H), 6.72 (s, 1H), 3.94 (s, 3H), 3.90 (s, 3H), 3.89 (s, 3H), 2.57 (s, 3H); ^{13}C NMR (101 MHz, $CDCl_3$) δ 202.1, 155.1, 152.9, 143.7, 136.3, 118.0 (t, *J* = 22.8 Hz), 112.0 (t, *J* = 236.4 Hz), 106.5, 61.9, 60.9, 56.2, 30.9; ^{19}F NMR (376 MHz, $CDCl_3$) δ -109.2 (d, *J* = 54.9 Hz, 2F); HRMS (ESI) calcd for $C_{12}H_{14}F_2NaO_4$ [M+Na$^+$]: 283.0752, found: 283.0754.

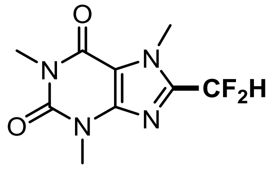

6a: white solid, 18.0mg, 74% yield; R_f = 0.6 (60% EtOAc/ petroleum ether); ^{1}H NMR (400 MHz, DMSO-d_6) δ 7.13 (t, J = 52.9 Hz, 1H), 3.43 (s, 3H), 3.37 (s, 3H), 3.24 (s, 3H); Other spectral data of **6a** were consistent with previous reported data.[1]

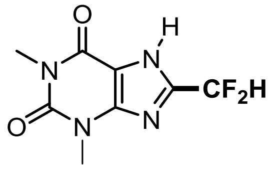

6b: white solid, 15.1mg, 70% yield; R_f = 0.6 (80% EtOAc/ petroleum ether); ^{1}H NMR (400 MHz, DMSO-d_6) δ 14.6 (s, 1H), 7.13 (t, J = 52.9 Hz, 1H), 3.44 (s, 3H), 3.24 (s, 3H); Other spectral data of **6b** were consistent with previous reported data.[1]

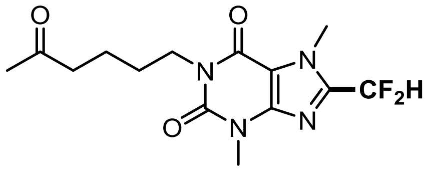

6c: white solid, 12.5mg, 38% yield; R_f = 0.3 (30% EtOAc/ petroleum ether); ^{1}H NMR (400 MHz, $CDCl_3$) δ 6.73 (t, J = 52.2 Hz, 1H), 4.13 (s, 3H), 4.02-3.98 (m, 2H), 3.53 (s, 3H), 2.49 (t, J = 6.9 Hz, 2H), 2.13 (s, 3H), 1.69-1.58 (m, 4H); Other spectral data of **6c** were consistent with previous reported data.[1]

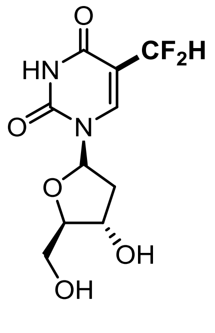

6d: grey solid, 22.5mg, 81% yield; R_f = 0.6 (100% EtOAc); ^{1}H NMR (400 MHz, CD_3CN) δ 9.30 (s, 1H), 8.15 (s, 1H), 6.38 (t, J = 54.8 Hz, 1H), 5.93 (t, J = 6.3 Hz, 1H), 4.16-4.11 (m, 1H), 3.67 (q, J = 3.3 Hz, 1H), 3.57-3.45 (m, 2H), 3.37-3.35 (m,1H), 3.19 (t, J = 4.8 Hz, 1H), 2.02-1.94 (m, 1H), 1.74-1.72 (m, 1H); ^{13}C NMR (101 MHz, CD_3CN) δ 161.5, 150.5, 141.4, 112.41 (t, J = 234.5 Hz), 108.08 (t, J = 23.2 Hz), 88.2,

86.4, 70.9, 61.6, 41.2; ^{19}F NMR (376 MHz, CD_3CN) δ -117.2 (d, *J* = 161.2 Hz, 2F); HRMS (ESI) calcd for $C_{10}H_{12}F_2N_2O_5$ [M-H^+]: 277.0636, found: 277.0640.

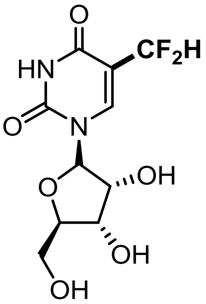

6e: yellow solid, 16.7mg, 57% yield; R_f = 0.5 (5% MeOH/ CH_2Cl_2); ^{1}H NMR (400 MHz, CD_3CN) δ 9.39 (s, 1H), 8.13 (s, 1H), 6.64 (t, *J* = 54.9 Hz, 1H), 6.08 (dd, *J* = 3.6, 1.6 Hz, 1H), 4.22 (d, *J* = 6.8 Hz, 1H), 4.11 (s, 2H), 3.94 (d, *J* = 3.7 Hz, 1H), 3.85-3.7 (m, 4H); ^{13}C NMR (101 MHz, CD_3CN) δ 160.3 (t, *J* = 3.9 Hz), 149.3, 141.4 (t, *J* = 7.7 Hz), 111.5 (t, *J* = 234.5 Hz), 106.1 (t, *J* = 23.3 Hz), 85.8, 84.7, 75.6, 75.2, 60.6; ^{19}F NMR (376 MHz, CD_3CN) δ -112.08 (dd, *J* = 54.1, 34.3 Hz, 2F); HRMS (ESI) calcd for $C_{10}H_{13}F_2N_2O_6$ [M+H^+]: 295.0736, found: 295.0731.

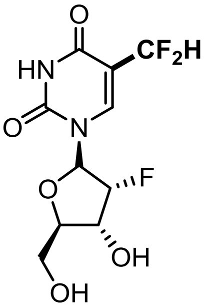

6f: colorless oil, 18.4mg, 62% yield; R_f = 0.7 (100% EtOAc); ^{1}H NMR (400 MHz, CD_3CN) δ 9.37 (s, 1H), 8.50 (s, 1H), 6.59 (t, *J* = 54.8 Hz, 1H), 5.92 (dd, *J* = 16.0, 1.2 Hz, 1H), 4.96 (dd, *J* = 52.9, 3.9 Hz, 1H), 4.31-4.20 (m, 1H), 4.02-3.93 (m, 1H), 3.97-3.93(m,1H), 3.76-3.66 (m, 2H), 3.48 (t, *J* = 4.7 Hz, 1H); ^{13}C NMR (101 MHz, CD_3CN) δ 161.3 (t, *J* = 4.0 Hz), 150.2, 140.9 (t, *J* = 7.5 Hz), 112.2 (t, *J* = 235.6 Hz), 108.1 (t, *J* = 23.3 Hz), 94.5 (d, *J* = 185.4 Hz), 88.6 (d, *J* = 34.2 Hz), 83.7, 67.8 (d, *J* = 16.3 Hz), 59.3; ^{19}F NMR (376 MHz, CD_3CN) δ -112.2 (dd, *J* = 236.0, 54.9 Hz, 2F), -199.1 (ddd, *J* = 52.7, 23.3, 16.1 Hz, 1F); HRMS (ESI) calcd for $C_{10}H_{10}F_3N_2O_5$

[M-H$^+$]: 295.0542, found: 295.0551.

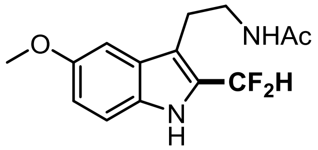

6g: yellow solid, 20.3 mg, 72% yield; R_f = 0.5 (80% EtOAc/ petroleum ether); ^{1}H NMR (400 MHz, CD_3OD) δ 7.95 (s, 1H), 7.18-6.87 (m, 3H), 6.78-6.74 (m, 1H), 3.72 (s, 3H), 3.32-3.27 (m, 2H), 3.21-3.20 (m, 1H), 2,92-2.88 (m, 2H), 1.79 (s, 3H); Other spectral data of **6g** were consistent with previous reported data.[4]

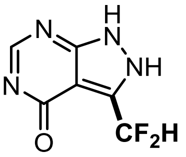

6h: white solid, 8.0 mg, 43% yield; R_f = 0.4 (80% EtOAc/ petroleum ether); ^{1}H NMR (400 MHz, DMSO-d_6) δ 8.11 (s, 1H), 7.14 (t, J = 53.4 Hz, 1H); ^{13}C NMR (101 MHz, DMSO-d_6) δ156.4, 154.5, 148.9, 141.3 (t, J = 27.9 Hz), 110.4 (t, J = 233.8 Hz), 102.8; ^{19}F NMR (376 MHz, DMSO-d_6) δ -112.6 (d, J = 53.2 Hz, 2F); HRMS (ESI) calcd for $C_6H_4F_2N_4O$ [M-H$^+$]: 185.0275, found: 185.0278.

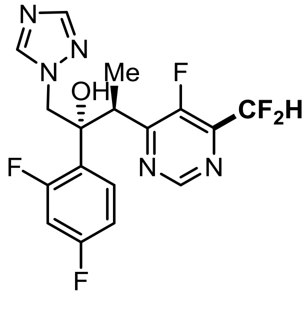

6i: colorless oil, 10.0 mg, 25% yield; R_f = 0.4 (60% EtOAc/ petroleum ether); ^{1}H NMR (400 MHz, $CDCl_3$) δ 8.95 (s, 1H), 7.97 (s, 1H), 7.67-7.60 (m, 1H), 7.53 (s, 1H), 6.89-6.80 (m, 1H), 6.26 (s, 1H) 4.55 (dd, J = 120.9, 14.2 Hz, 2H), 4.20 (qd, J = 7.1, 1.1 Hz, 1H), 1.13 (d, J = 7.1 Hz, 3H); ^{13}C NMR (101 MHz, $CDCl_3$) δ 162.9 (dd, J = 251.5, 12.4Hz), 161.9 (d, J = 13.0 Hz), 158.4 (dd, J = 246.4, 11.7 Hz), 153.3 (d, J = 270.1 Hz), 152.9 (d, J = 9.3 Hz), 151.1, 146.9 (td, J = 26.3, 11.7 Hz), 144.1, 130.6 (dd, J = 9.3, 5.5 Hz), 123.4 (dd, J = 12.2, 4.0 Hz), 111.8 (dd, J = 20.7, 3.3 Hz), 111.1 (t, J

= 244.3 Hz), 104.2 (t, *J* = 25.9Hz), 57.2 (d, *J* = 5.4 Hz), 37.4 (d, *J* = 5.0 Hz), 29.7, 16.1; ^{19}F NMR (376 MHz, $CDCl_3$) δ -113.7 (s, 1F), -114.4(s , 1F), -123.8(dd, *J* = 53.5, 10.2 Hz, 2F), -140.9 (s, 1F); HRMS (ESI) calcd for $C_{17}H_{15}F_5N_5O$ [M+H$^+$]: 400.1191, found: 400.1195.

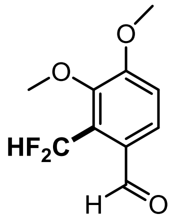

6j: colorless oil, 6.1mg, 28% yield; R_f = 0.5 (10% EtOAc/ petroleum ether); ^{1}H NMR (400 MHz, $CDCl_3$) δ 10.36 (s,1H), 7.87 (d, *J* = 8.7 Hz, 1H), 7.34 (t, *J* = 53.4 Hz, 1H); ^{13}C NMR (101 MHz, $CDCl_3$) δ 189.6 (t, *J* = 4.0 Hz), 157.1, 128.7 (t, *J* = 24.1 Hz), 127.9, 126.6, 113.6, 111.8(t, *J* = 4.0 Hz), 110.3, 62.0, 56.2; ^{19}F NMR (376 MHz, $CDCl_3$) δ-104.3 (d, *J* = 54.6 Hz, 2F); HRMS (ESI) calcd for $C_{10}H_{11}F_2O_3$ [M+H$^+$]: 217.0671, found: 217.0667.

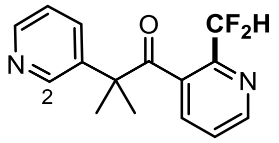

6k: colorless oil, 9.1mg, 33% yield; 3:1 r.r.; R_f = 0.6 (50% EtOAc/ petroleum ether); ^{1}H NMR (400 MHz, $CDCl_3$) δ 6.68-6.58 (m, 3H), 7.96-7.76 (m, 1H), 7.67-7.56 (m, 2H), 7.36-7.28 (m, 1H), 6.57 (t, *J* = 55.2 Hz, 1H), 1.68 (d, *J* = 2.0 Hz, 6H); ^{13}C NMR (101 MHz, $CDCl_3$) δ 200.5, 154.9 (t, *J* = 26.0 Hz), 152.6, 150.8, 147.4, 139.4, 138.4, 133.5, 132.5, 124.1, 119.8 (t, *J* = 3.0 Hz), 113.1 (t, *J* = 242.1 Hz), 50.5, 27.1; ^{19}F NMR (376 MHz, $CDCl_3$) -121.6 (d, *J* = 55.3 Hz, 2F); HRMS (ESI) calcd for $C_{15}H_{15}F_2N_2O$ [M+H$^+$]: 277.1147, found: 277.1156.

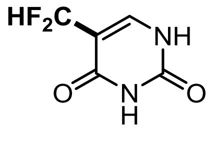

6l: white solid, 9.3mg, 57% yield; R_f = 0.3 (50% EtOAc/ petroleum ether); ^{1}H NMR

(400 MHz, DMSO-d_6) δ11.42 (s, 1H), 7.79 (s, 1H), 6.67 (t, J = 54.6 Hz, 1H); ^{13}C NMR (101 MHz, DMSO-d_6) δ 162.1 (t, J = 3.1 Hz), 151.3, 143.1 (t, J = 7.5 Hz), 112.7 (t, J = 233.4 Hz), 106.4 (t, J = 23.1 Hz); ^{19}F NMR (376 MHz, DMSO-d_6) δ -115.00 (d, J = 54.7 Hz, 2F); HRMS (ESI) calcd for $C_5H_3F_2N_2O_2$ [M-H$^+$]: 161.0163, found: 161.0173.

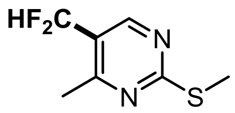

6m: colorless oil, 6.0mg, 31% yield; R_f = 0.7 (10% EtOAc/ petroleum ether); ^{1}H NMR (400 MHz, $CDCl_3$) δ 8.51 (s, 1H), 6.72 (t, J = 54.7 Hz, 1H), 2.58 (s, 3H), 2.56 (s, 3H); ^{13}C NMR (101 MHz, $CDCl_3$) δ 174.9, 165.8, 154.4, 121.1 (t, J = 22.7 Hz), 113.1 (t, J = 238.4 Hz), 21.8, 14.2; ^{19}F NMR (376 MHz, $CDCl_3$) δ -118.9 (d, J = 54.1 Hz, 2F); HRMS (ESI) calcd for $C_7H_9F_2N_2S$ [M+H$^+$]: 191.0449, found: 191.0448.

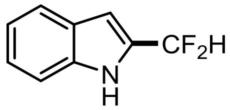

8: white solid, 9mg, 54% yield; 10:1 r.r.; R_f = 0.5 (15% EtOAc/ petroleum ether); ^{1}H NMR (400 MHz, $CDCl_3$) δ 8.40 (s, 1H), 7.67 (d, J = 7.9 Hz, 1H), 7.43 (d, J = 8.2 Hz, 1H), 7.30 (t, J = 7.6 Hz, 1H), 7.17 (t, J = 7.5 Hz, 1H), 6.84 (t, J = 54.8 Hz, 1H), 6.77 (s, 1H); ^{13}C NMR (101 MHz, $CDCl_3$) δ 136.3, 130.0 (t, J = 24.5 Hz), 127.0, 124.1, 121.7, 120.7, 111.6, 110.5 (t, J = 7.1 Hz), 104.0 (t, J = 7.1 Hz); ^{19}F NMR (376 MHz, $CDCl_3$) δ -109.9 (d, J = 53.0 Hz, 2F); HRMS (ESI) calcd for $C_9H_6F_2N$ [M-H$^+$]: 166.0468, found: 166.0474.

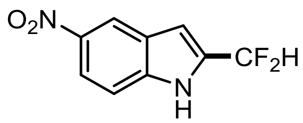

10: yellow solid, 15mg, 71% yield; R_f = 0.4 (40% EtOAc/ petroleum ether); ^{1}H NMR (400 MHz, $CDCl_3$) δ 8.61 (s, 1H), 7.96 (d, *J* = 8.9 Hz, 1H), 7.61 (t, *J* = 56.0 Hz, 1H), 7.56 (d, *J* = 8.9 Hz, 1H), 7.47 (d, *J* = 3.6 Hz, 1H), 7.12-7.09 (m ,1H); ^{13}C NMR (101 MHz, $CDCl_3$) δ 141.9, 138.7, 128.3, 125.3, 122.2 (t, *J* = 25.4 Hz), 118.8, 113.2, 112.3 (t, *J* = 239.2 Hz), 105.6 (t, *J* = 5.0 Hz); ^{19}F NMR (376 MHz, $CDCl_3$) δ -110.5 (dd, *J* = 54.2, 2.6Hz, 2F); HRMS (ESI) calcd for $C_9H_5F_2N_2O_2$ [M-H$^+$]: 211.0319, found: 211.0326.

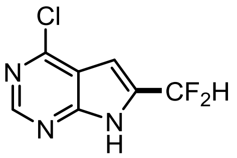

12: white solid, 7.8mg, 38% yield; R_f = 0.7 (30% EtOAc/ petroleum ether); ^{1}H NMR (400 MHz, DMSO-d_6) δ 13.41 (s, 1H), 8.73 (s, 1H), 7.31 (t, *J* = 53.7 Hz, 1H), 7.01 (s, 1H); ^{13}C NMR (101 MHz, DMSO-d_6) δ152.2, 152.2, 133.0 (t, *J* = 25.9 Hz), 129.6, 115.7, 110.1 (t, *J* = 234.2 Hz), 100.0 (t, *J* = 6.8 Hz); ^{19}F NMR (376 MHz, DMSO-d_6) δ -107.4 (d, J = 53.8 Hz, 2F); HRMS (ESI) calcd for $C_7H_3ClF_2N_3$ [M-H$^+$]: 201.9984, found: 201.9993.

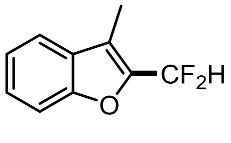

14: colorless oil, 16.7mg, 92% yield; R_f = 0.7 (2% EtOAc/ petroleum ether); ^{1}H NMR (400 MHz, $CDCl_3$) δ 7.58 (d, *J* = 7.8 Hz, 1H), 7.50 (d, *J* = 8.3 Hz, 1H), 7.39 (t, *J* = 7.7 Hz, 1H), 7.30 (t, *J* = 7.4 Hz, 1H), 6.81 (t, *J* = 53.4 Hz, 1H), 2.38 (t, *J* = 2.6 Hz, 3H); ^{13}C NMR (101 MHz, $CDCl_3$) δ154.3, 143.0 (t, *J* = 26.7 Hz), 128.7, 126.2, 123.0, 120.3, 117.0 (t, *J* = 4.5Hz) ,111.8, 109.2 (t, *J* = 234.7 Hz), 7.5; ^{19}F NMR (376 MHz, $CDCl_3$) δ -115.1 (d, *J* = 53.3 Hz, 2F); HRMS (EI) calcd for $C_{10}H_8F_2O$ (M$^+$): 182.0543, found: 182.0536.

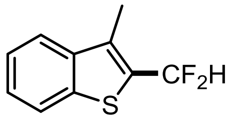

16: colorless oil, 12.3mg, 65% yield; R_f = 0.7 (2% EtOAc/ petroleum ether); ^{1}H NMR (400 MHz, $CDCl_3$) δ 7.89-7.82 (m, 1H), 7.77-7.74 (m, 1H), 7.45-7.40 (m, 2H), 7.04 (t, *J* = 55.6 Hz, 1H), 2.49 (t, *J* = 2.0 Hz, 3H); ^{13}C NMR (101 MHz, $CDCl_3$) δ 139.6, 139.1, 133.0 (t, *J* = 7.6 Hz), 130.1 (t, *J* = 24.3 Hz), 125.9, 124.5, 122.8, 122.6, 111.4 (t, *J* = 235.7 Hz), 11.8; ^{19}F NMR (376 MHz, $CDCl_3$) δ -104.4 (d, *J* = 55.4 Hz, 2F); HRMS (EI) calcd for $C_{10}H_8F_2S$ (M^+): 198.0315, found: 198.0306.

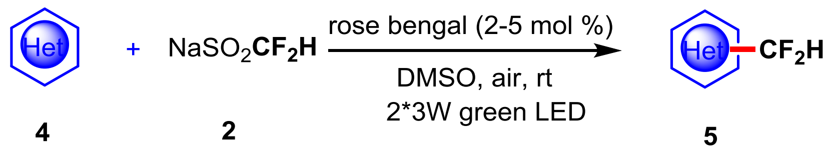

A many products form and most of starting materials remain

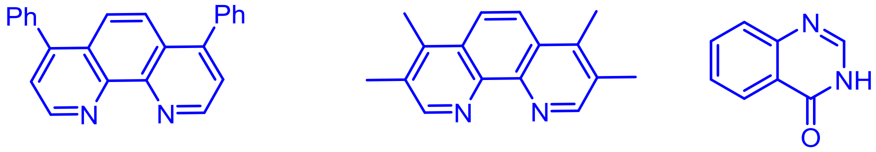

B no desired products formation

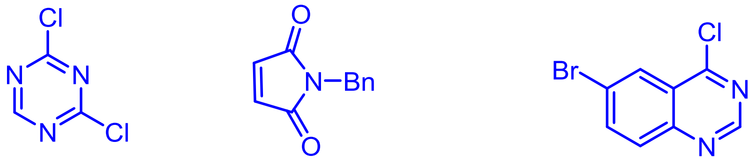

C no reaction

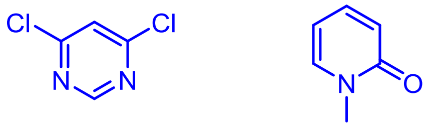

D trace products formation

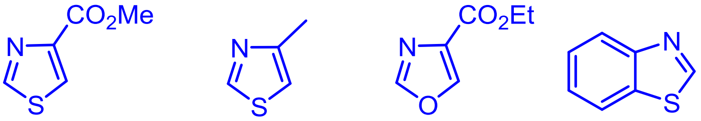

Supplementary Figure 2. Unsuccessful substrates

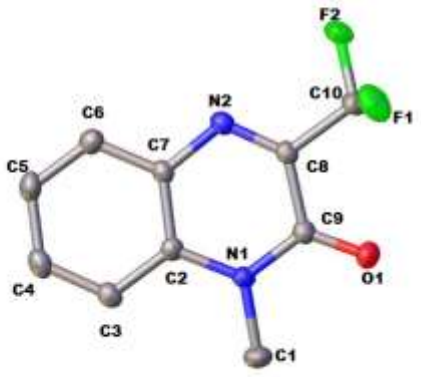

Supplementary Figure 3. The x-ray single crystal structure of **3a**.

Supplementary Table 1. Crystal data and structure refinement for shelxl.
X-ray crystallography data of 3a.

Identification code	shelxl
Empirical formula	$C_{10}H_8F_2N_2O$
Formula weight	210.18
Temperature	113(2) K
Wavelength	0.71073 A
Crystal system, space group	Monoclinic, P2(1)/c
Unit cell dimensions	a = 7.1342(14) A alpha = 90 deg.
	b = 10.658(2) A beta = 106.21(3) deg.
	c = 12.383(3) A gamma = 90 deg.
Volume	904.1(3) A^3
Z, Calculated density	4, 1.544 Mg/m^3
Absorption coefficient	0.130 mm^{-1}
F(000)	432
Crystal size	0.200 x 0.180 x 0.120 mm
Theta range for data collection	2.566 to 27.766 deg.
Limiting indices	-9<=h<=9, -13<=k<=13, -16<=l<=16
Reflections collected / unique	10502 / 2123 [R(int) = 0.0739]
Completeness to theta = 25.242	99.9 %
Absorption correction	Semi-empirical from equivalents
Max. and min. transmission	1 and 0.5621
Refinement method	Full-matrix least-squares on F^2
Data / restraints / parameters	2123 / 0 / 137

Goodness-of-fit on F^2	0.974
Final R indices [I>2sigma(I)]	R1 = 0.0472, wR2 = 0.1104
R indices (all data)	R1 = 0.0705, wR2 = 0.1232
Extinction coefficient	n/a
Largest diff. peak and hole	0.333 and -0.447 e.A^{-3}

Evaluation of anti-tumor activity

The *in vitro* anti-tumor activity of 6d against all cell lines was assessed using the CCK-8, according to the manufacturer's instructions. To test 6d, MCF-7 and HepG-2 cells were seeded into 96-well plates at a density of 5000 cells/well in 100 μL of RPMI 1640 medium containing 10% FBS, 1% penicillin, and 1% streptomycin and cultured for 24 h in 5% CO_2 at 37 ℃. HCT116 cells were seeded into 96-well plates at a density of 5000 cells/well in 100 μL of McCoy's 5A medium supplemented with 10% FBS, 1% penicillin, and 1% streptomycin and cultured for 24 h in 5% CO_2 at 37 ℃. Likewise, Hela cells were seeded into 96-well plates at a density of 5000 cells/well in 100 μL of MEM supplemented with 10% FBS, 1% penicillin, and 1% streptomycin and cultured for 24 h in 5% CO_2 at 37 ℃. 6d was dissolved in PBS contain 10% DMSO and then diluted to the required concentration. It was then added to the cell-containing wells and further incubated at 37 ℃ under 5% CO_2 for 72 h. Subsequently, 10 μL of CCK-8 was added into each well and incubated for another 1 h. The plates were then measured at 450 nm using a SpectraMax ® M5 plate reader (Molecular Devices, San Jose, CA, USA). All experiments were carried out five times. For trifluridine, the same procedures were performed by varying the concentration of the specie in question to determine the cytotoxicity.

Eight standard reaction mixtures in 10 mL schlenk tube were equipped with a magnetic stir bar, added 1a (0.2 mmol, 1.0 equiv), CF_2HSO_2Na (0.4 mmol, 2.0 equiv) and rose bengal (0.004 mmol, 2 mol%) in DMSO (1.0 mL). Then the mixture was stirred and irradiated by two 3W green LEDs at room temperature. After 1.5 h, the green LEDs were turned off, and one schlenk tube was removed from the irradiation setup for analysis. The remaining seven schlenk tubes were stirred in the absence of light for an additional 1.5 h. Then, one schlenk tube was removed for analysis, and the green LEDs were turned back on to irradiate the remaining six reaction mixtures. After an additional 1.5 h of irradiation, the green LEDs were turned off, and one schlenk tube was removed for analysis. The remaining five schlenk tubes were stirred in the absence of light for an additional 1.5 h. Then, schlenk tube was removed for analysis, and the green LED s were turned back on to irradiate the remaining four reaction mixtures. After 1.5 h, the green LEDs were turned off, and one schlenk tube was removed for analysis. The remaining three schlenk tubes were stirred in the absence of light for an additional 1.5 h, then, a schlenk tube was removed for analysis and the green LEDs were turned back on to 8 irradiate the remaining two reaction mixtures. After 1.5 h, the green LEDs were turned off, and one schlenk tube was removed for analysis. The last schlenk tube was stirred in the absence of light for an additional 1.5 h, and then it was analyzed. The yield was determined by ^{19}F NMR spectroscopy using benzotrifluoride as the internal standard.

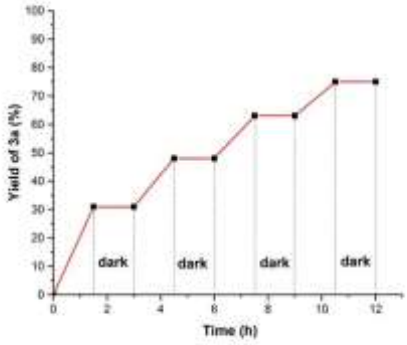

Supplementary Figure 4. Light on/ off experiment

Investigation on the effect of TEMPO and Oxygen.

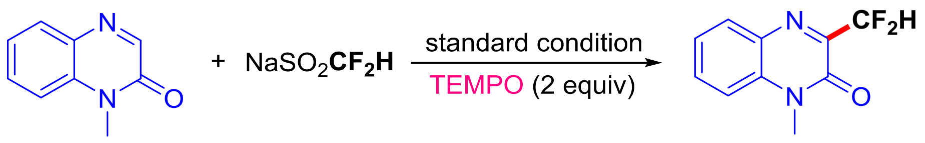

1a **3a**, 0%

Reaction conditions: a mixture of **1a** (0.1 mmol), $NaSO_2CF_2H$ (0.2 mmol), rose bengal (2 mol %) and 2,2,6,6-tetramethyl-1-piperidinyloxy (TEMPO, 0.2 mmol) in DMSO (1 mL) irradiated with two 3 W green LEDs for 12 hours at room temperature in air.

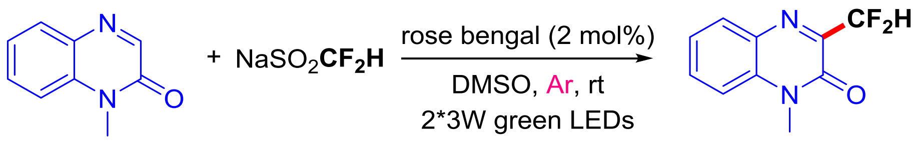

1a **3a**, 0%

Reaction conditions: a mixture of **1a** (0.1 mmol), $NaSO_2CF_2H$ (0.2 mmol), rose bengal (2 mol %) and 2,2,6,6-tetramethyl-1-piperidinyloxy (TEMPO, 0.2 mmol) in DMSO (1 mL) irradiated with two 3 W green LEDs for 12 hours at room temperature in argon.

The radical trapping experiments were conducted with and CF_2HSO_2Na under the standard conditions with a trapping agent 1,1-diphenylethylene (2.0 equiv) to capture the radical intermediate expected in our system, and the products were detected by HRMS techniques. Supplementary Figure 5 showed that 1,1-diphenylethylene, the most common trapping agent, captured diarylmethane radical with 1,1-diphenylethylene-trapped compound **17** observed. HRMS (ESI): compound **17**, HRMS (ESI) calcd for $C_{15}H_{13}F_2$ $[M+H]^+$: 231.0980, found: 231.0985.

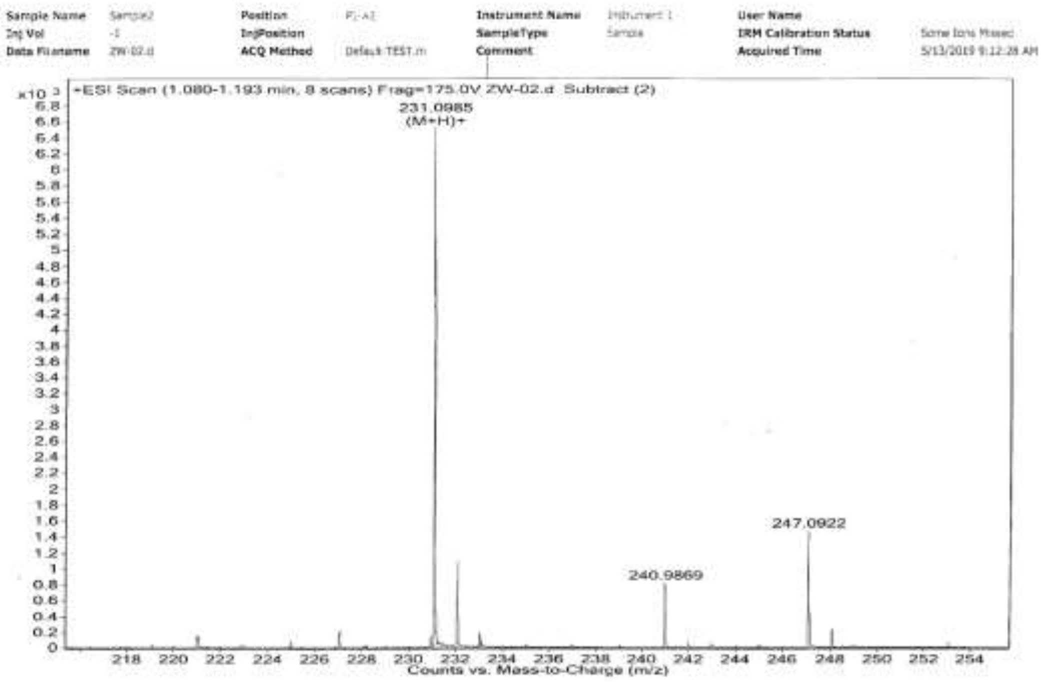

Supplementary Figure 5. Radical trapping experiment for **1a** and CF_2HSO_2Na under standard conditions with ethene-1,1-diyldibenzene (2.0 equiv)

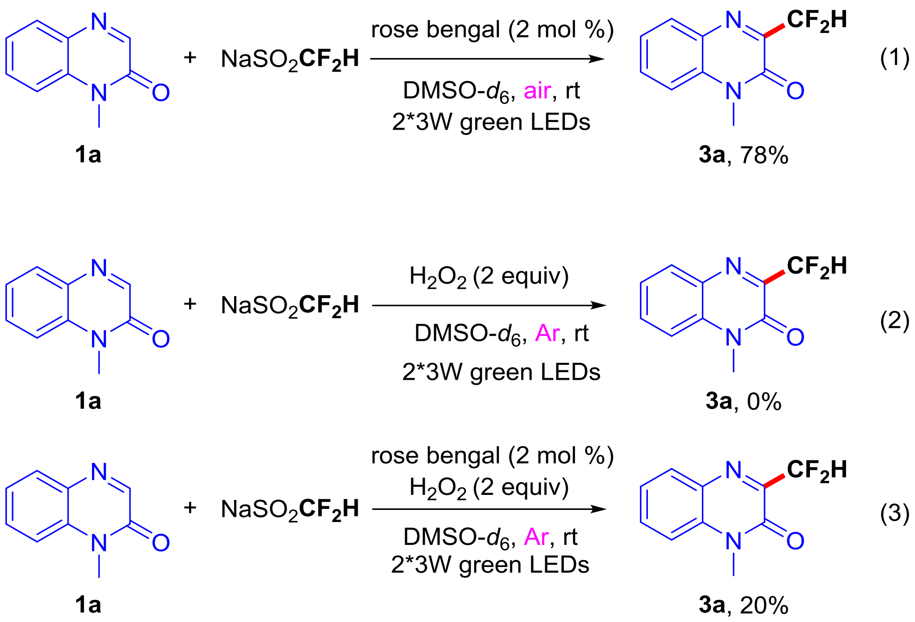

Supplementary Figure 6. Detection of hydrogen peroxide

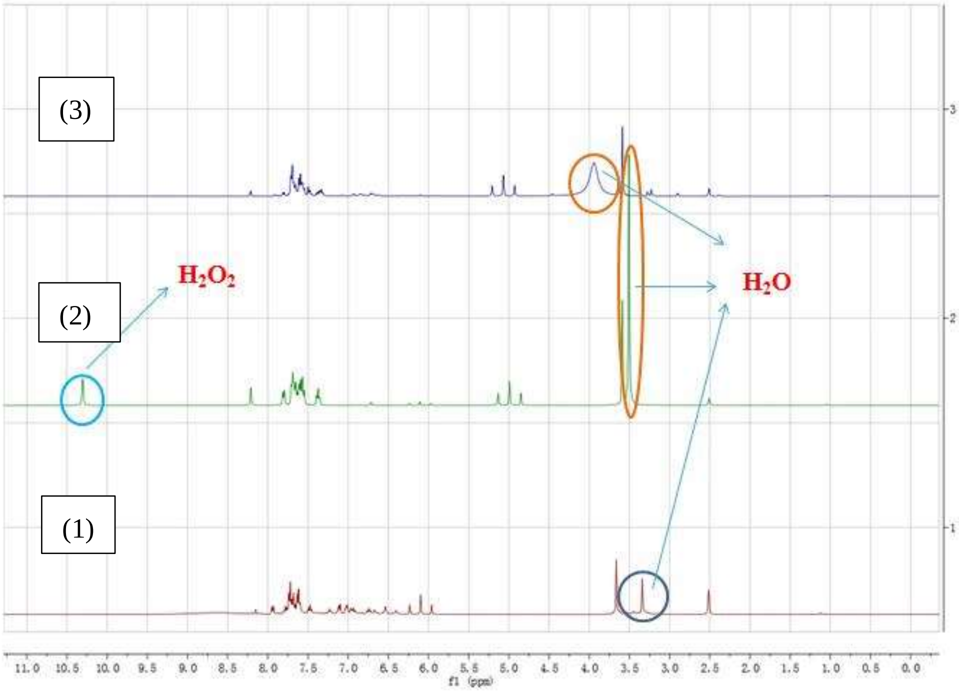

Supplementary Figure 7. ^{1}H NMR spectrum of detection of hydrogen peroxide

Supplementary Discussions

We did not observed the peak of hydrogen peroxide (H_2O_2) by *in situ* ^{1}H NMR analysis after the reaction mixture in DMSO-d_6 was irradiated with two 3W green LEDs for 12 hours in air at room temperature (Supplementary Figure 7, eq 1). However, the peak of water (H_2O) increased possibly due to the oxygen was finally converted to H_2O rather than H_2O_2. This is because (I) When the reaction was conducted in the H_2O_2 in the absence of rose bengal under the irradiation of two 3W green LEDs for 12h, no formation of desired product **3a** was observed by ^{1}H NMR analysis but the peak of hydrogen peroxide (H_2O_2) remained after the reaction (Supplementary Figure 7, eq 2). (II) When the reaction was conducted in the H_2O_2 (35 wt % aqueous solution) in the presence of rose bengal under the irradiation of two 3W green LEDs for 12h, a yield of 20% of **3a** was obtained and the peak of H_2O_2 in ^{1}H NMR spectrum disappeared after the reaction (Supplementary Figure 7, eq 3). Therefore, the generated H_2O_2 could participate in the catalytic cycle and ultimately convert to H_2O as the byproduct and and the result was similar to the previous report by Wu group.[6]

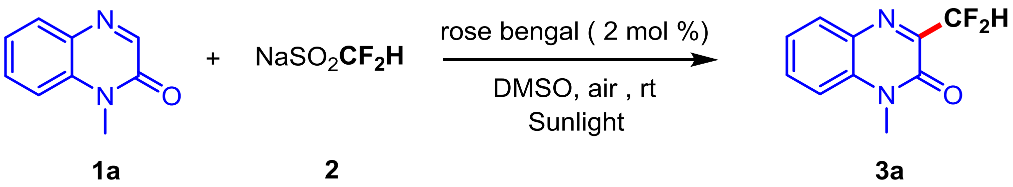

To a 10 mL Schlenk tube equipped with a magnetic stir bar, was added quinoxalin-2(1*H*)-ones **1a** (0.2mmol), CF_2HSO_2Na (0.4 mmol) and rose bengal (0.004 mmol, 2 mol%) in DMSO (1.0 mL) Then the mixture was stirred and irradiated by sunlight at room temperature for 12 h (Location: 39°6′2″ N, 117°9′51″ E). Afterward, the residue was added water (10 mL) and extracted with ethyl acetate (5 mL × 3). The combined organic phase was dried over Na_2SO_4. The resulting crude residue was purified *via* column chromatography on silica gel to afford **3a** in 68% yield.

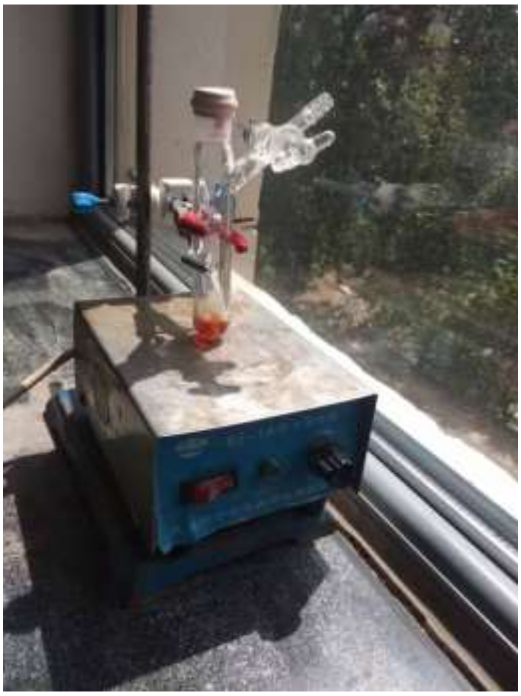

Supplementary Figure 8. Sunlight-driven experiment

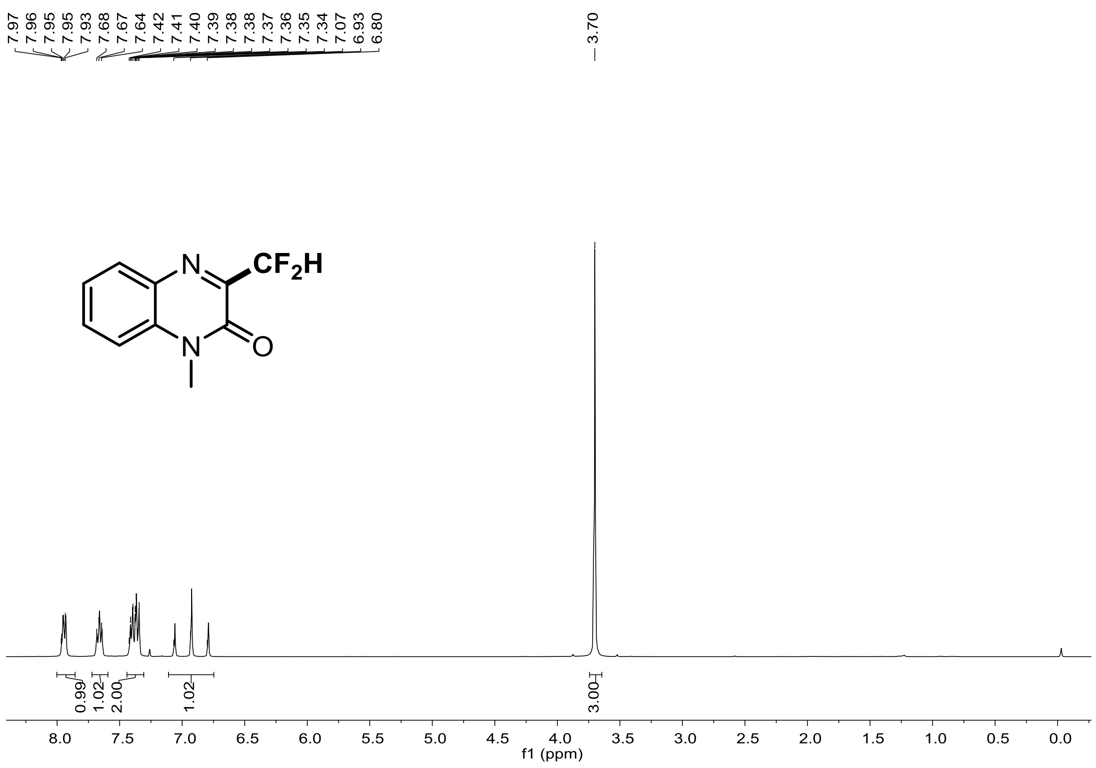

Supplementary Figure 9. ^{1}H NMR Spectrum of **3a**

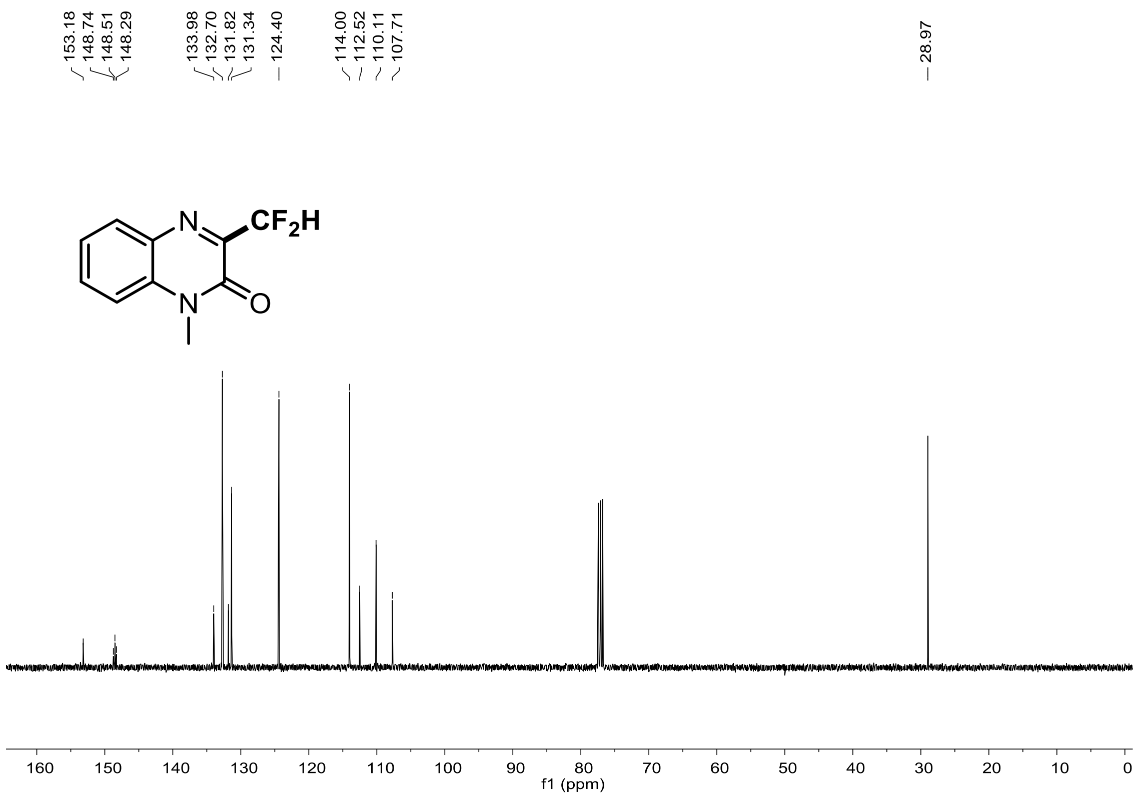

Supplementary Figure 10. ^{13}C NMR Spectrum of **3a**

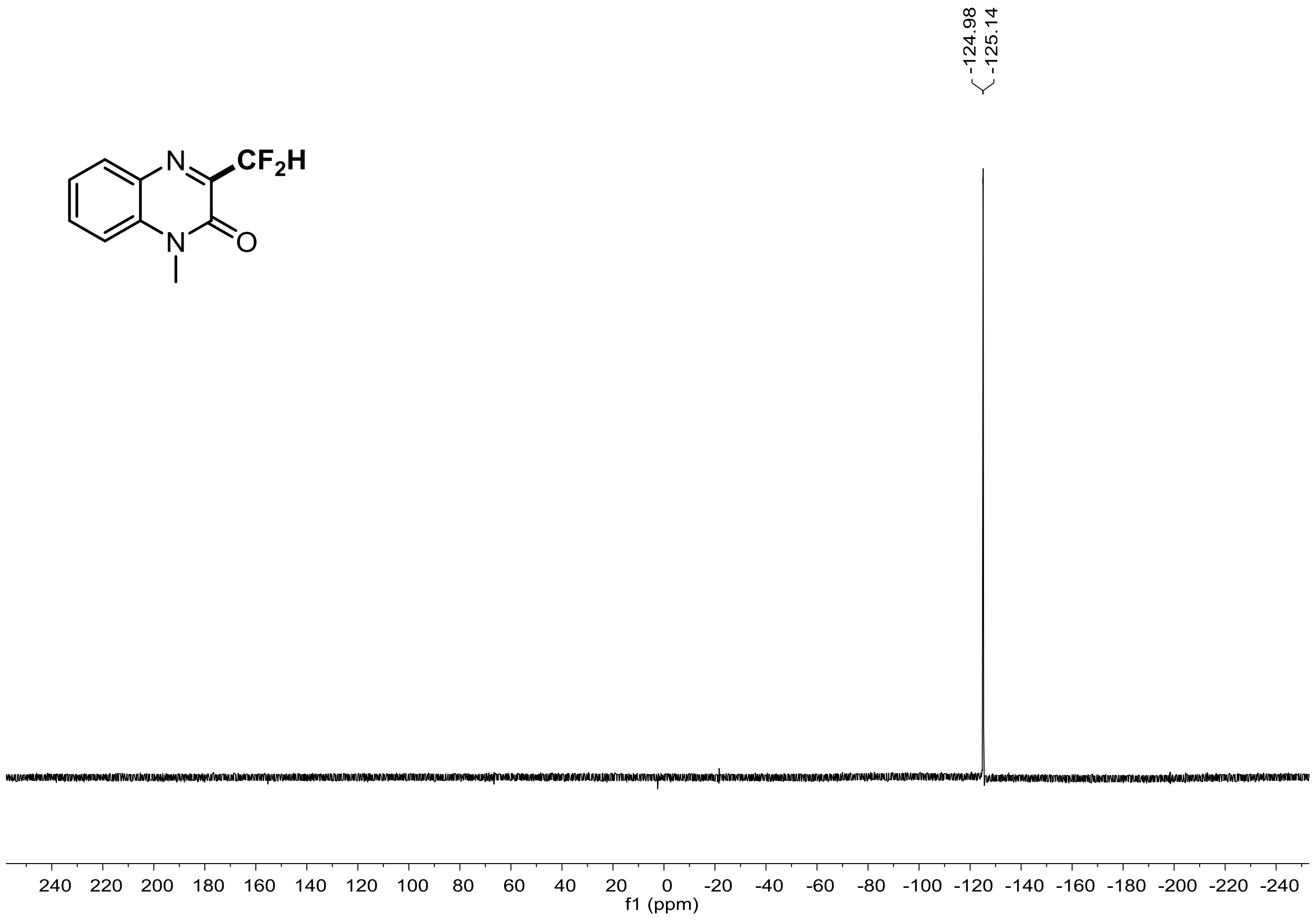

Supplementary Figure 11. ^{19}F NMR Spectrum of **3a**

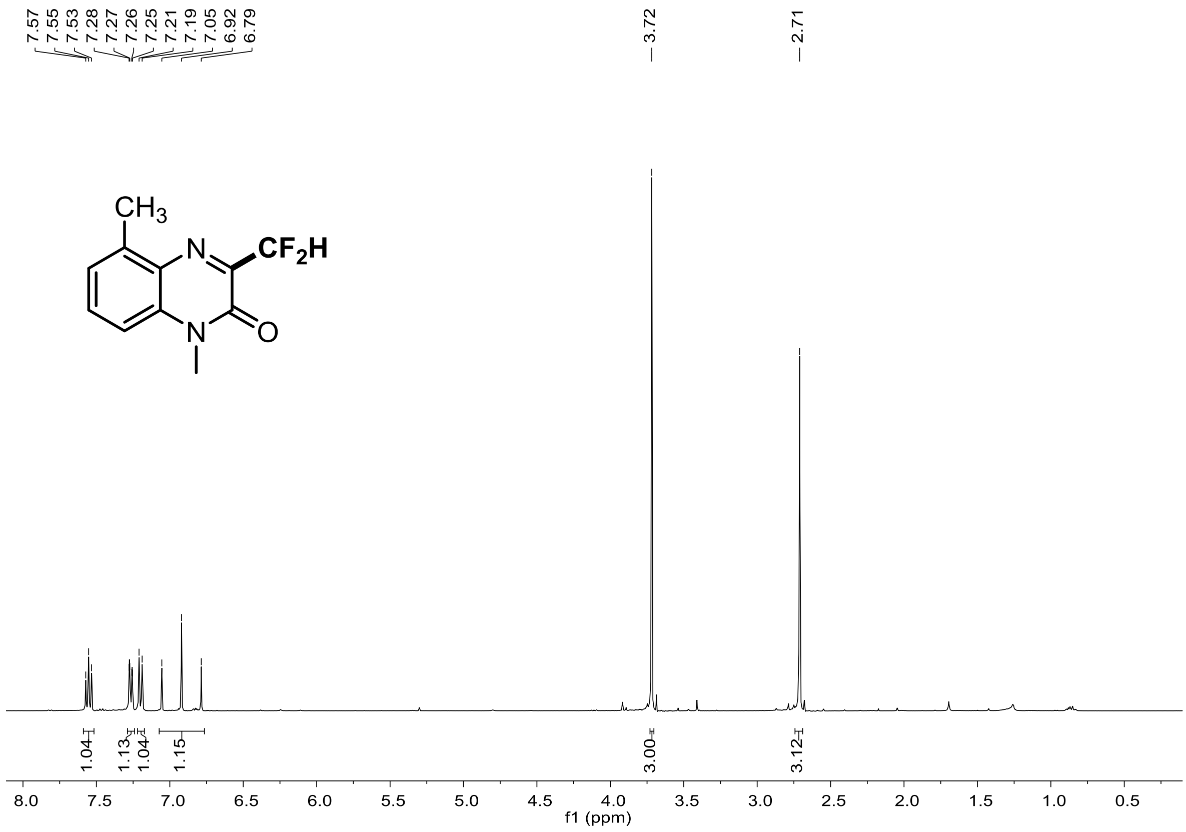

Supplementary Figure 12.^{1}H NMR Spectrum of **3b**

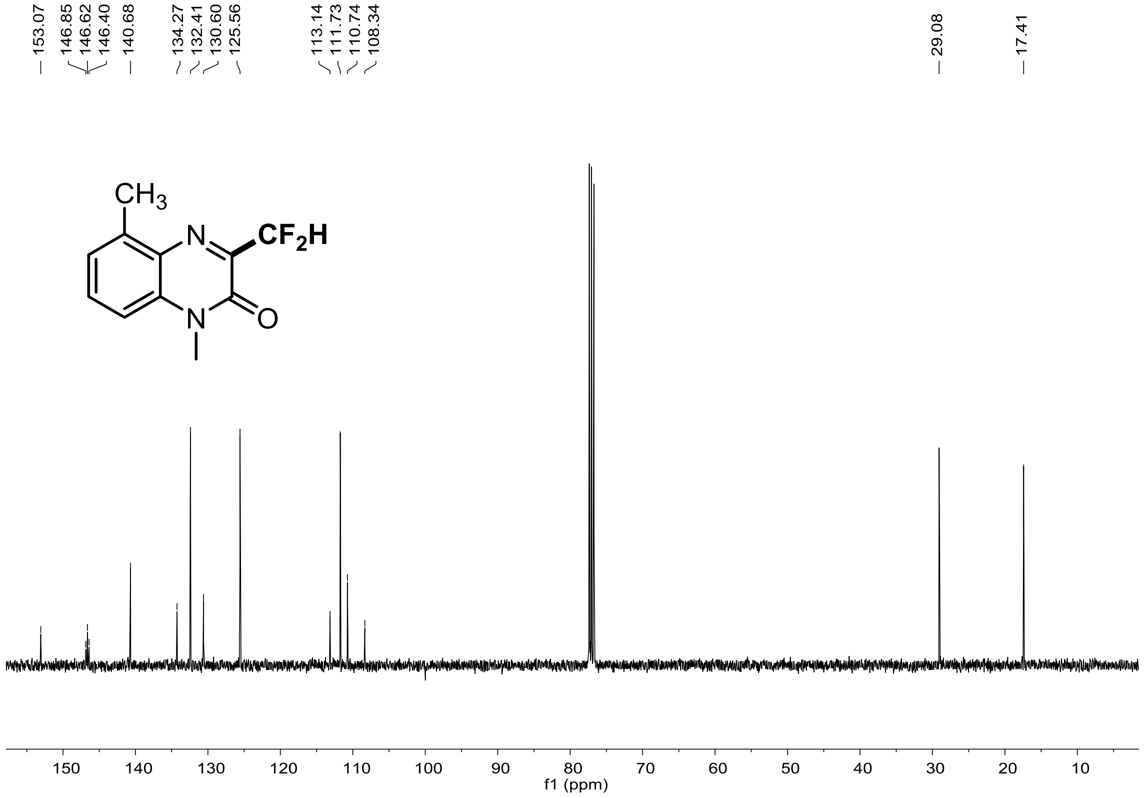

Supplementary Figure 13. ^{13}C NMR Spectrum of **3b**

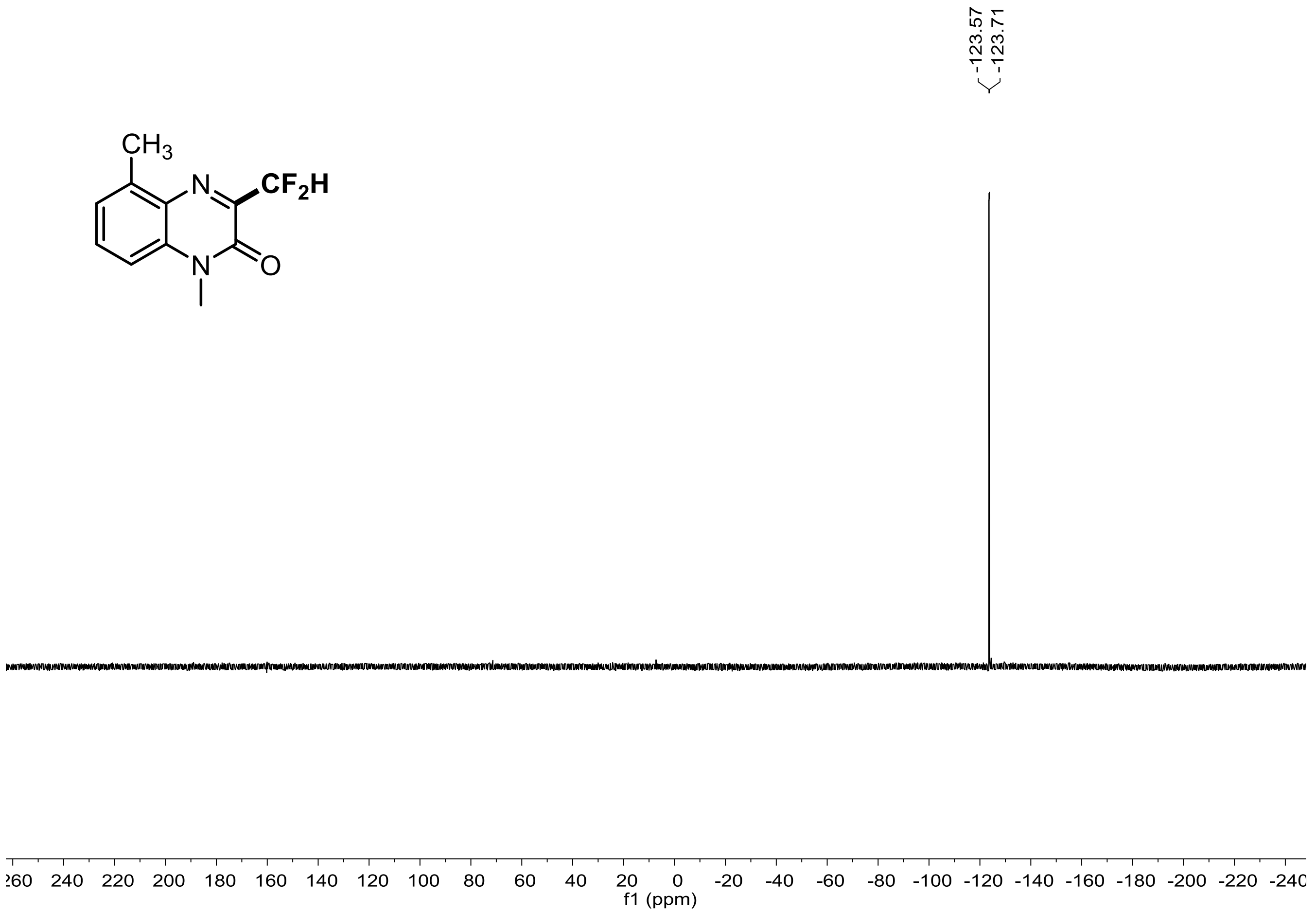

Supplementary Figure 14. ^{19}F NMR Spectrum of **3b**

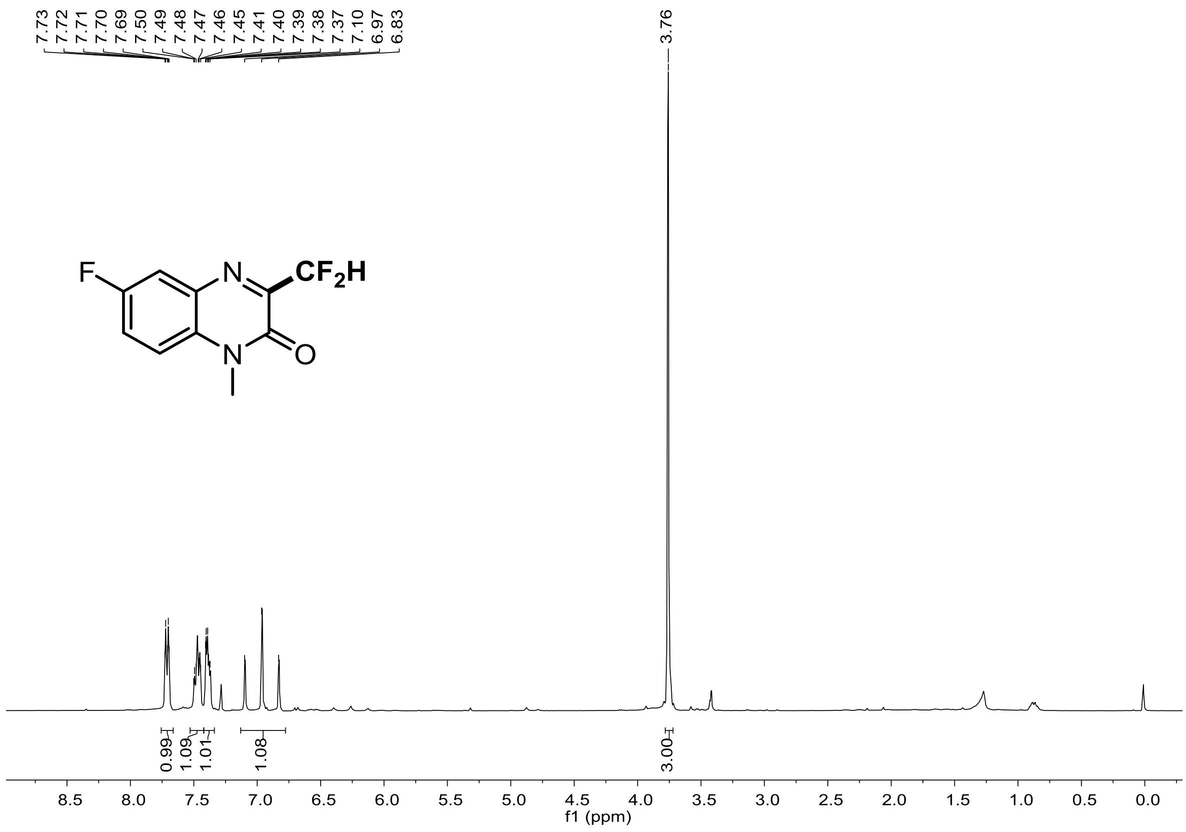

Supplementary Figure 15. ^{1}H NMR Spectrum of **3c**

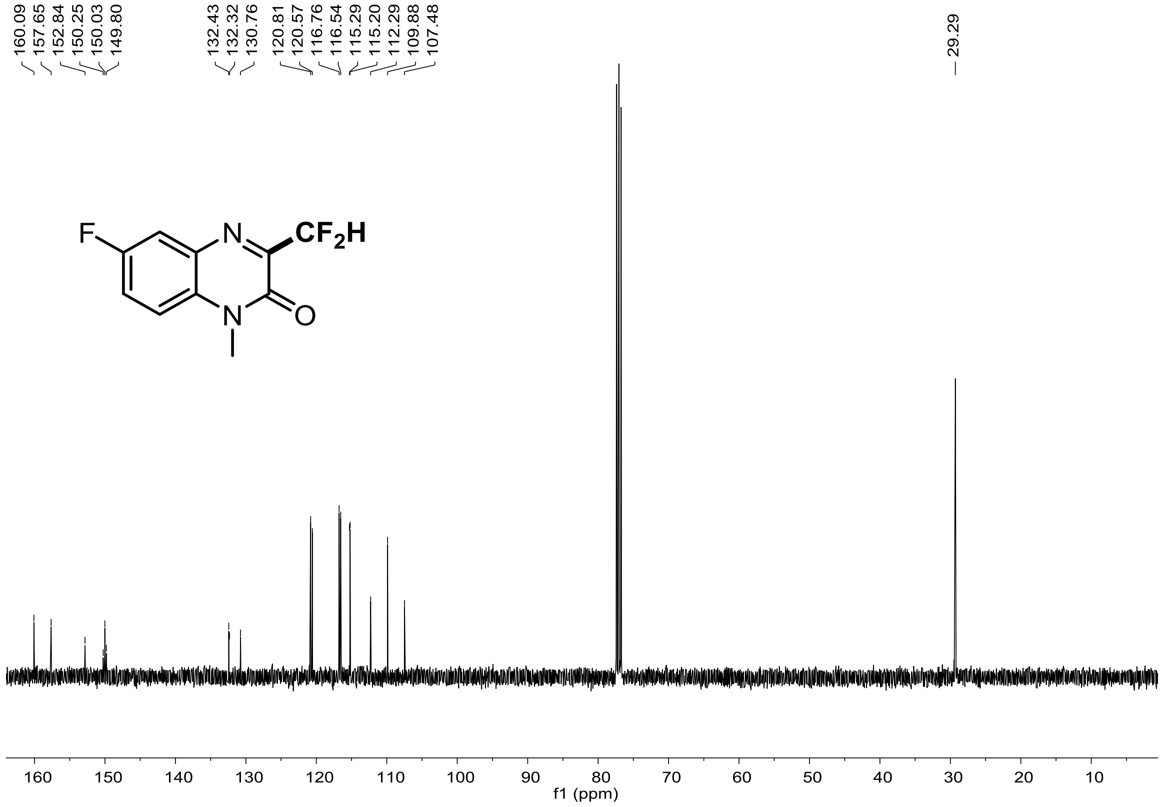

Supplementary Figure 16. ^{13}C NMR Spectrum of **3c**

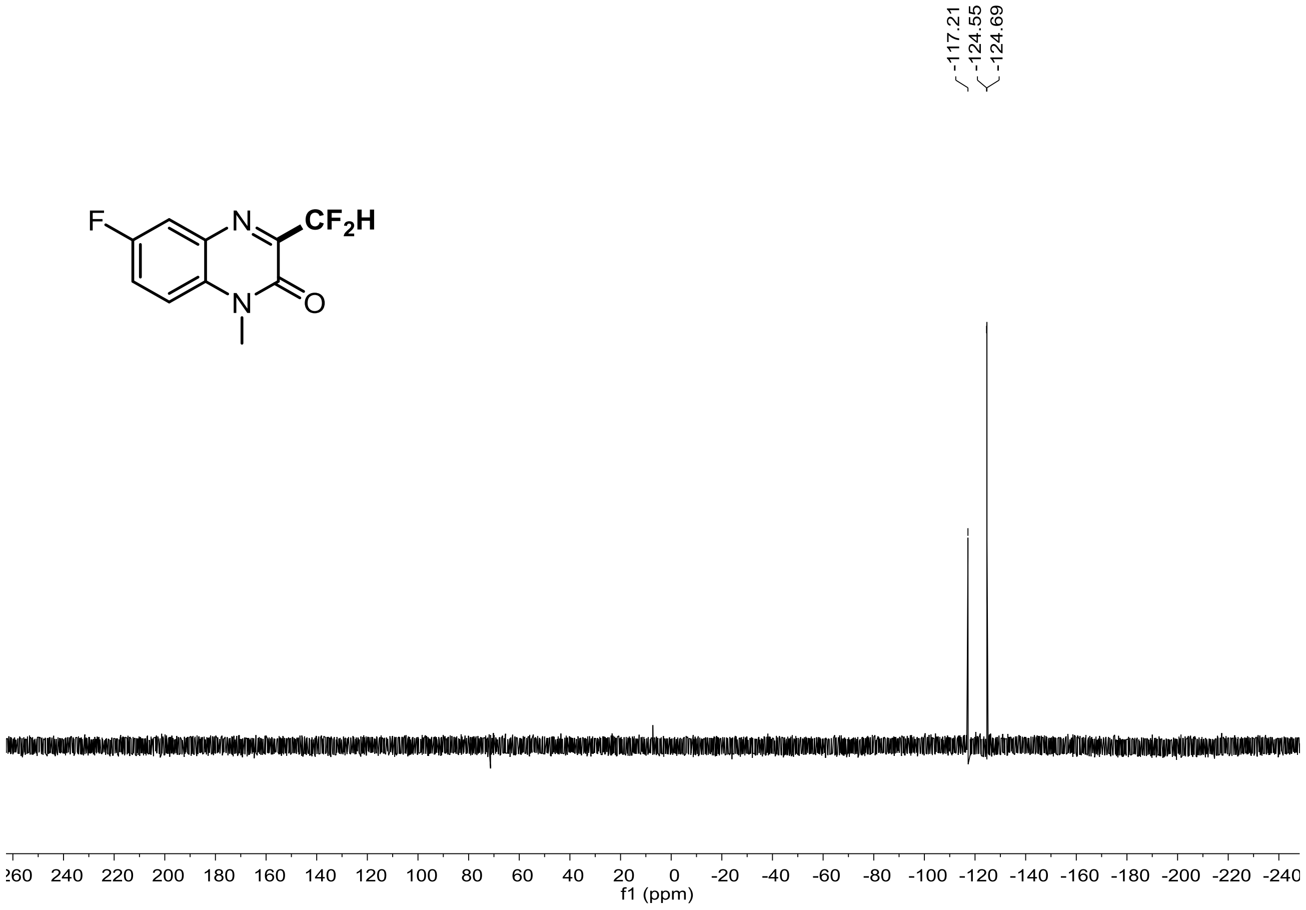

Supplementary Figure 17. ^{19}F NMR Spectrum of **3c**

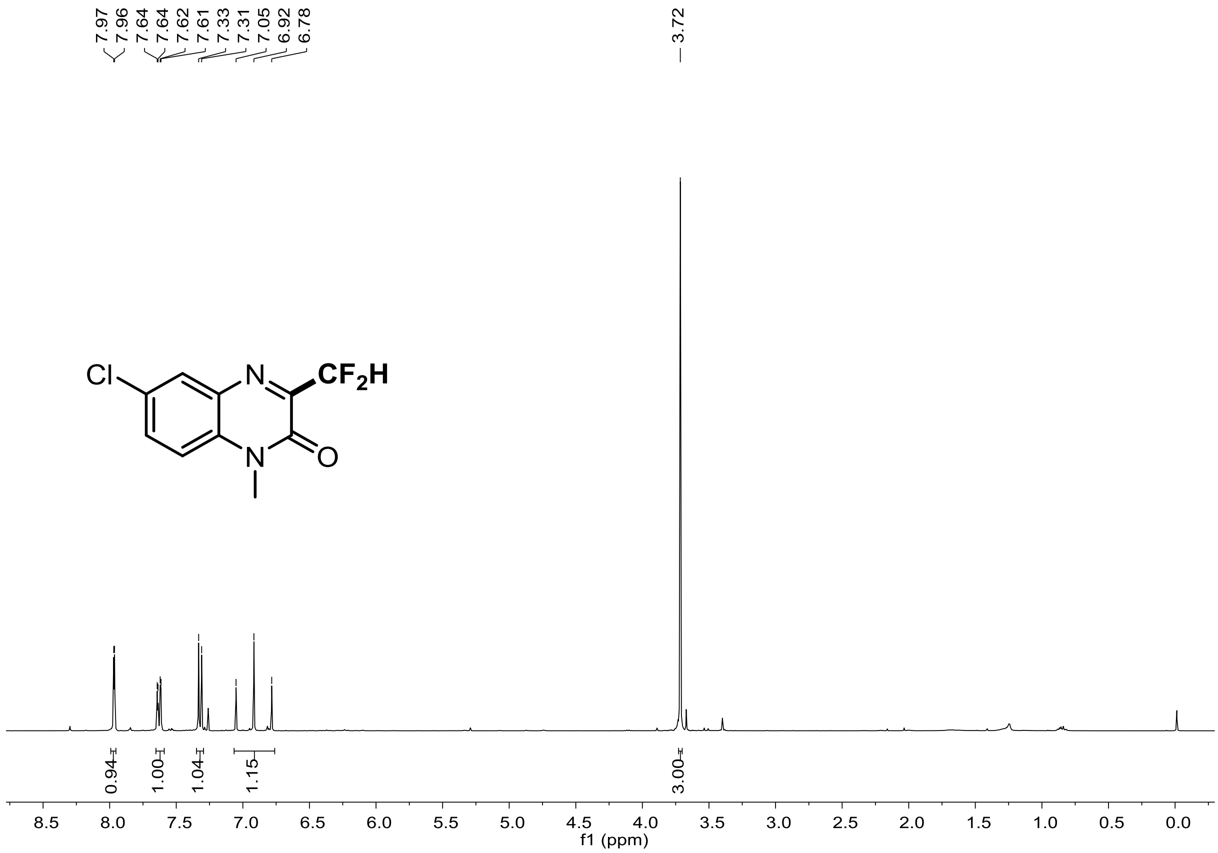

Supplementary Figure 18. ^{1}H NMR Spectrum of **3d**

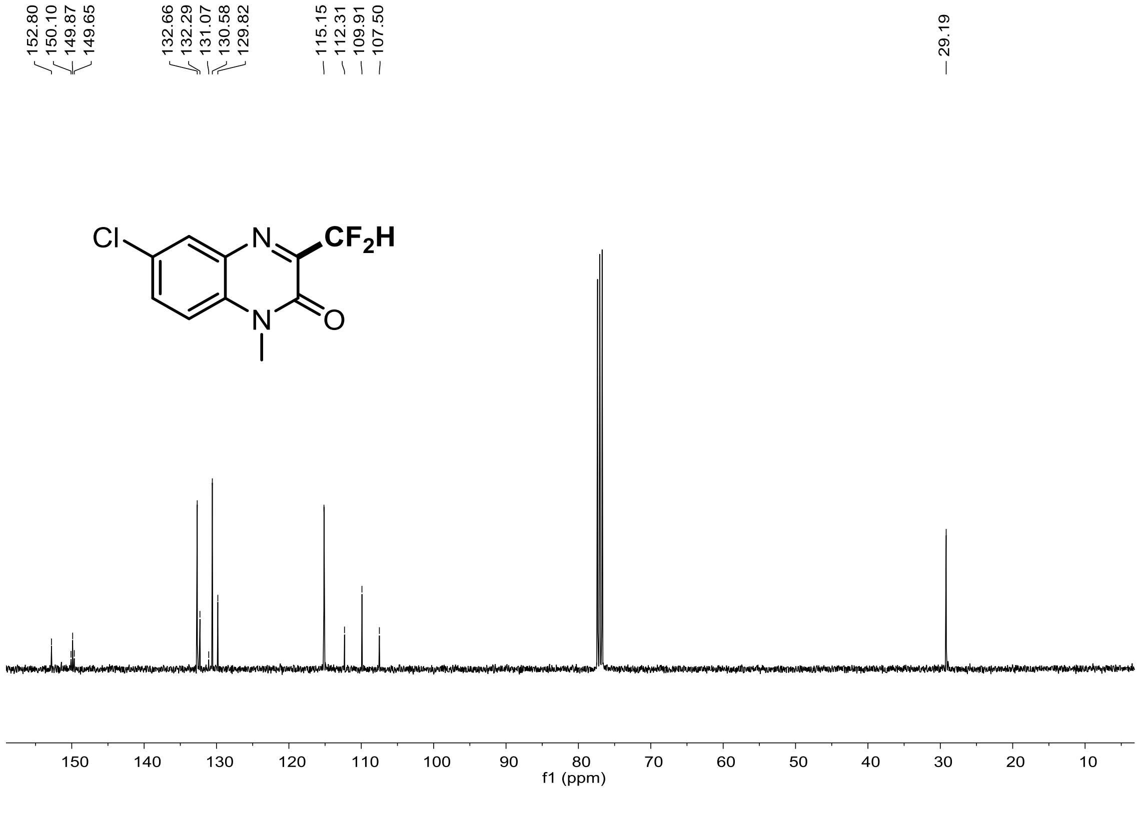

Supplementary Figure 19. ^{13}C NMR Spectrum of **3d**

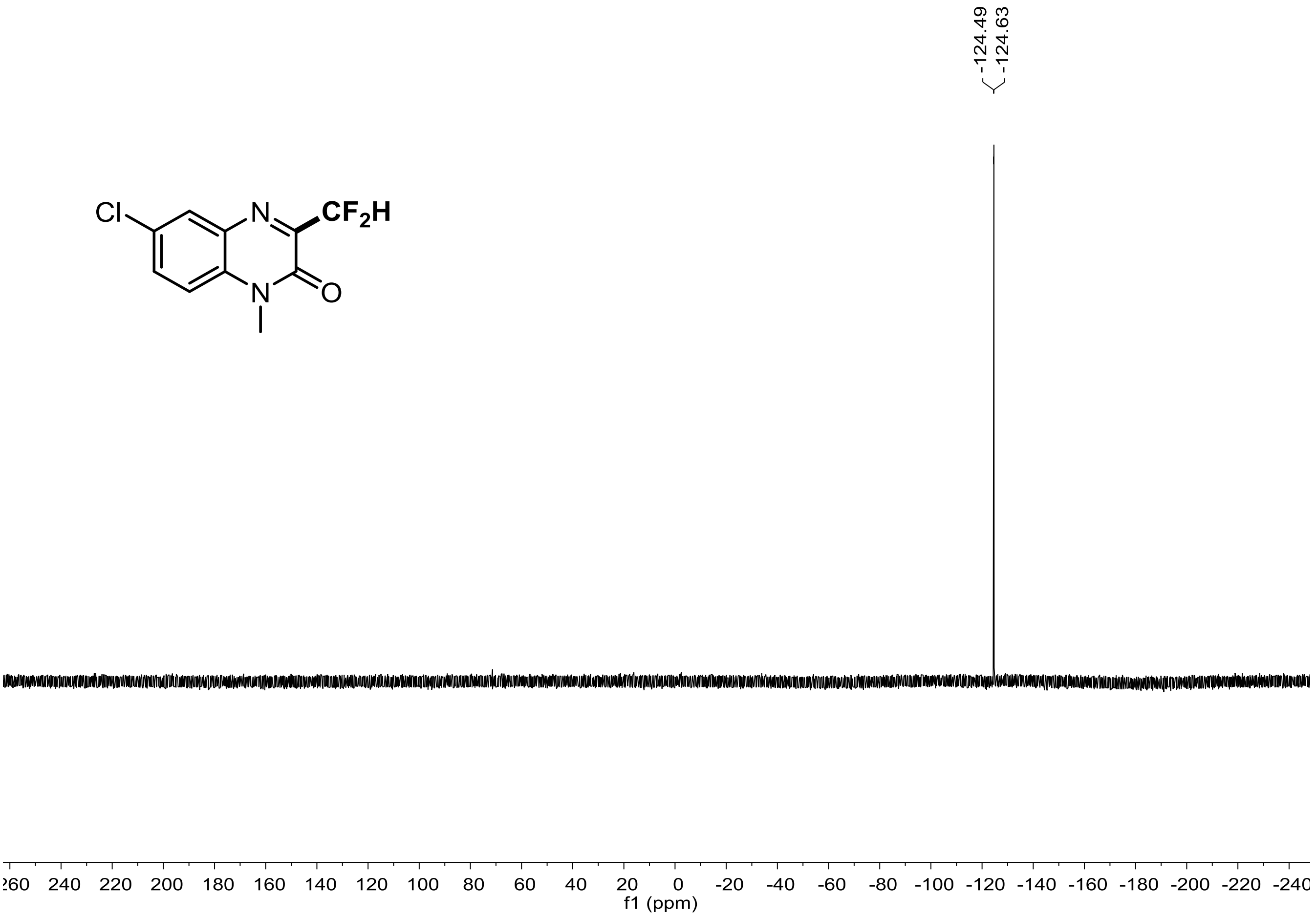

Supplementary Figure 20. ^{19}FNMR Spectrum of **3d**

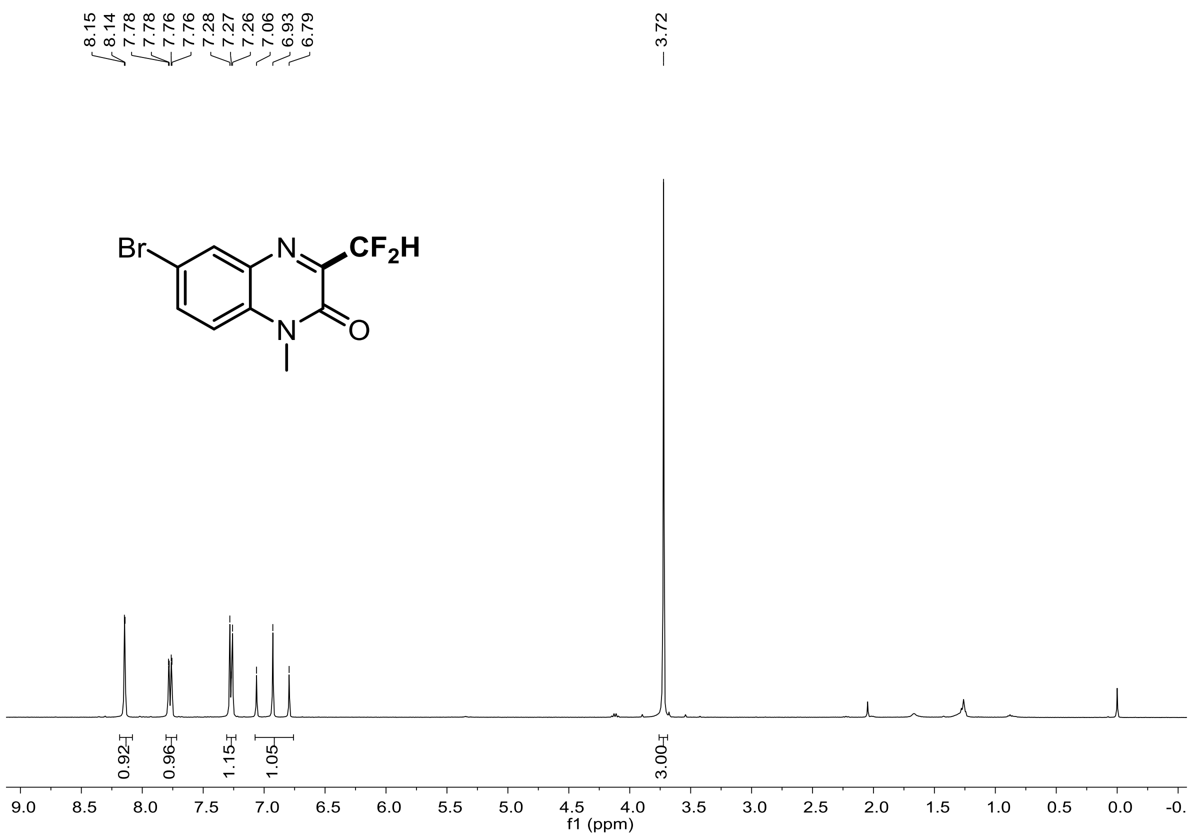

Supplementary Figure 21. ^{1}H NMR Spectrum of **3e**

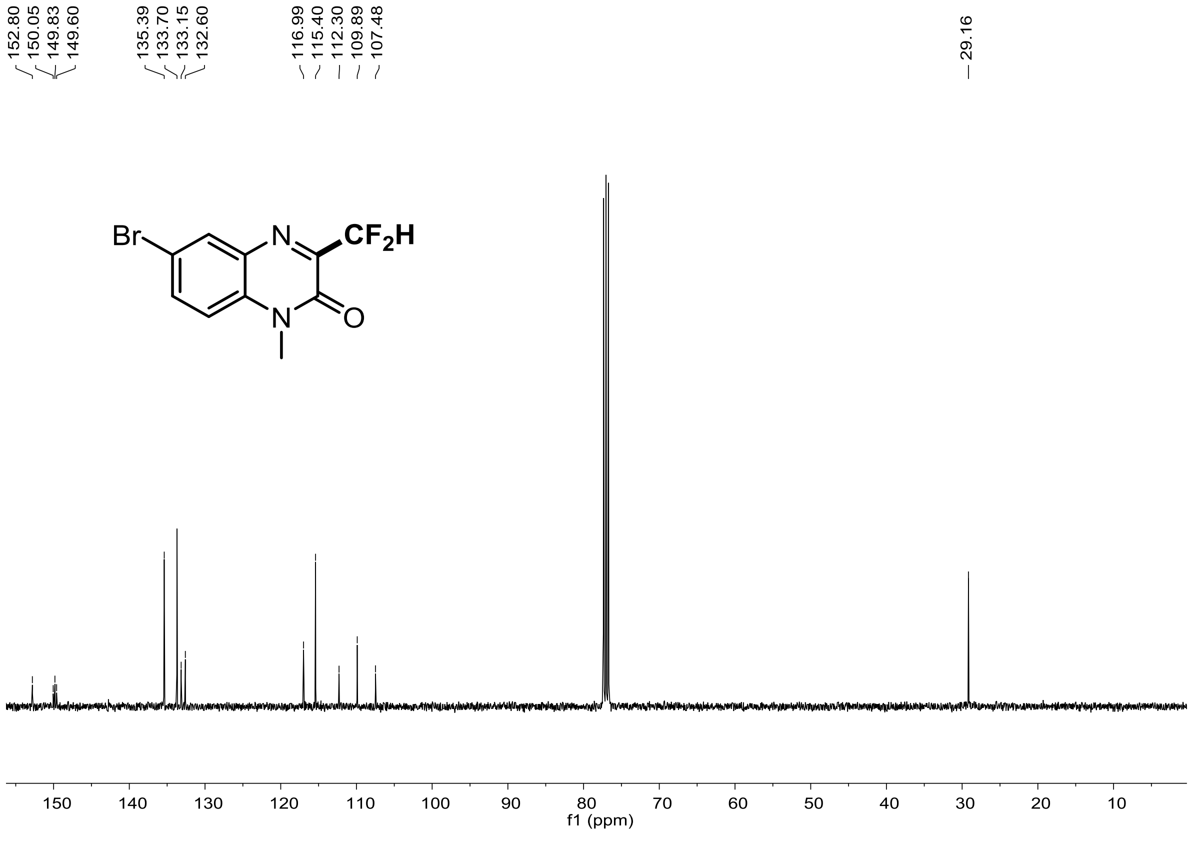

Supplementary Figure 22. ^{13}C NMR Spectrum of **3e**

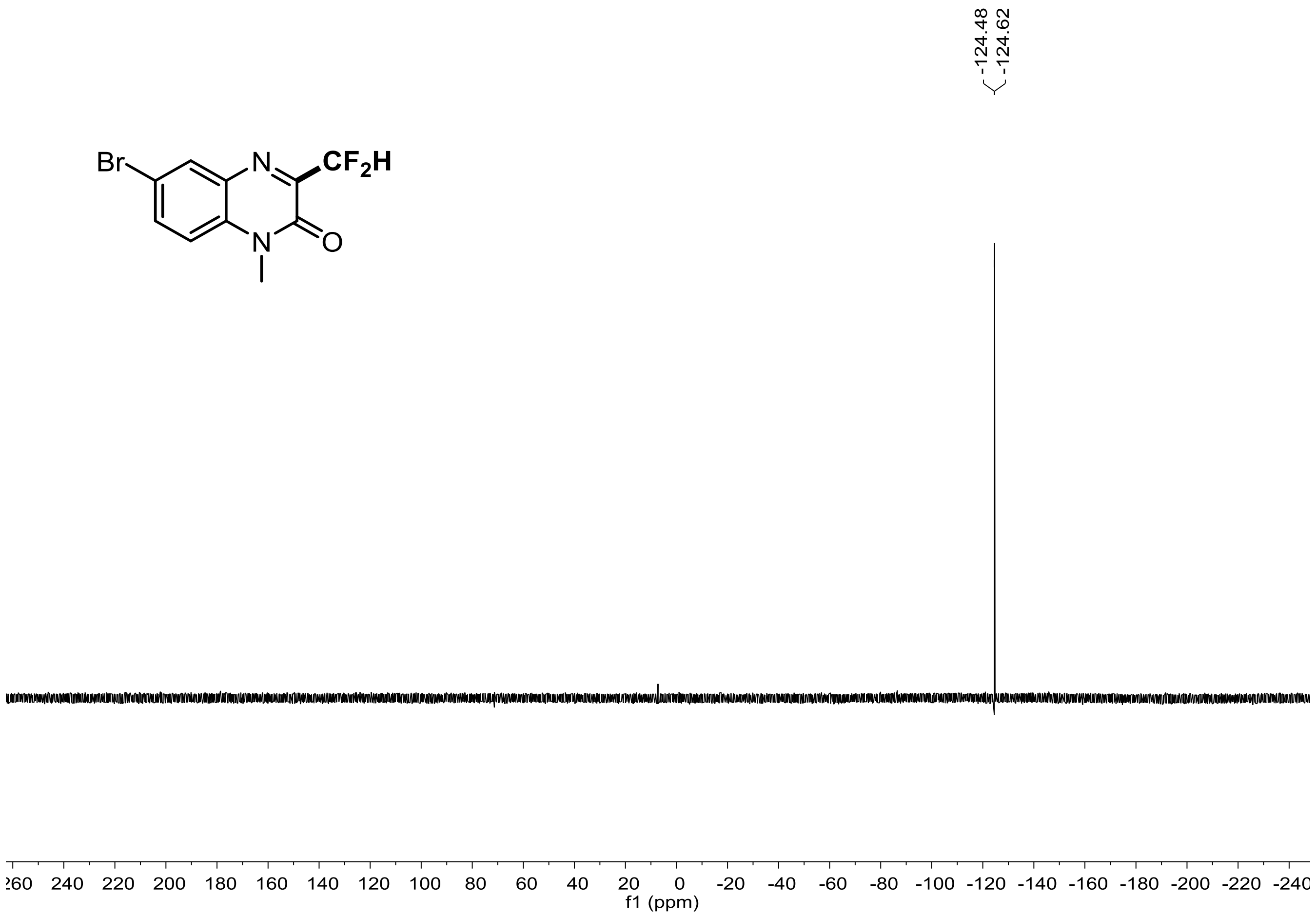

Supplementary Figure 23. ^{19}F NMR Spectrum of **3e**

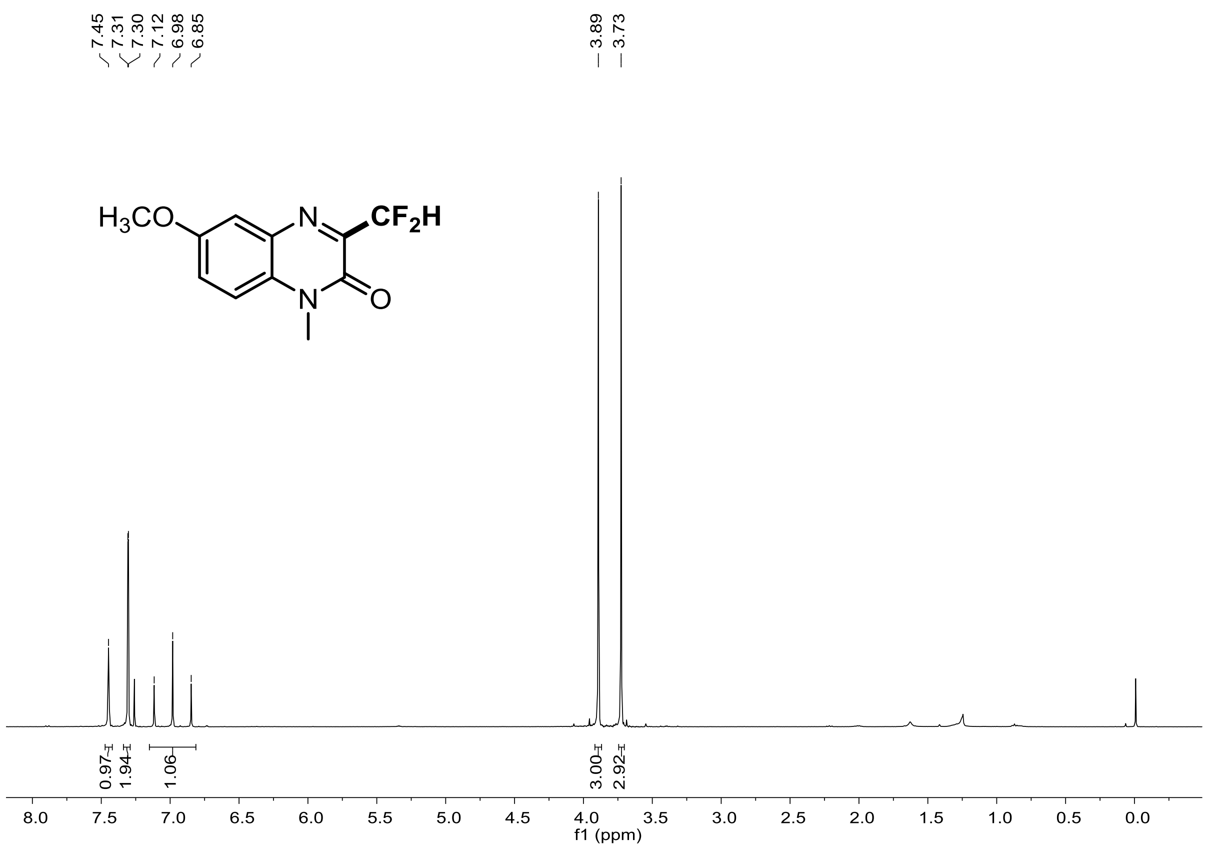

Supplementary Figure 24. ^{1}H NMR Spectrum of **3f**

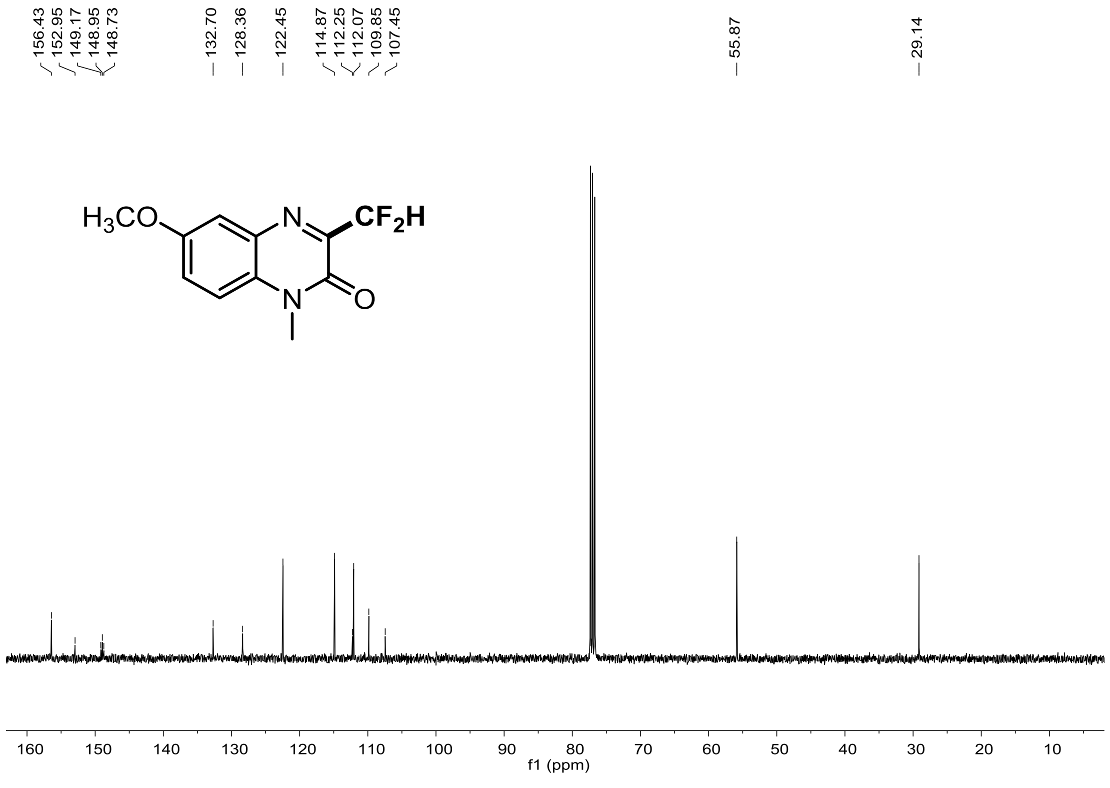

Supplementary Figure 25. ^{13}C NMR Spectrum of **3f**

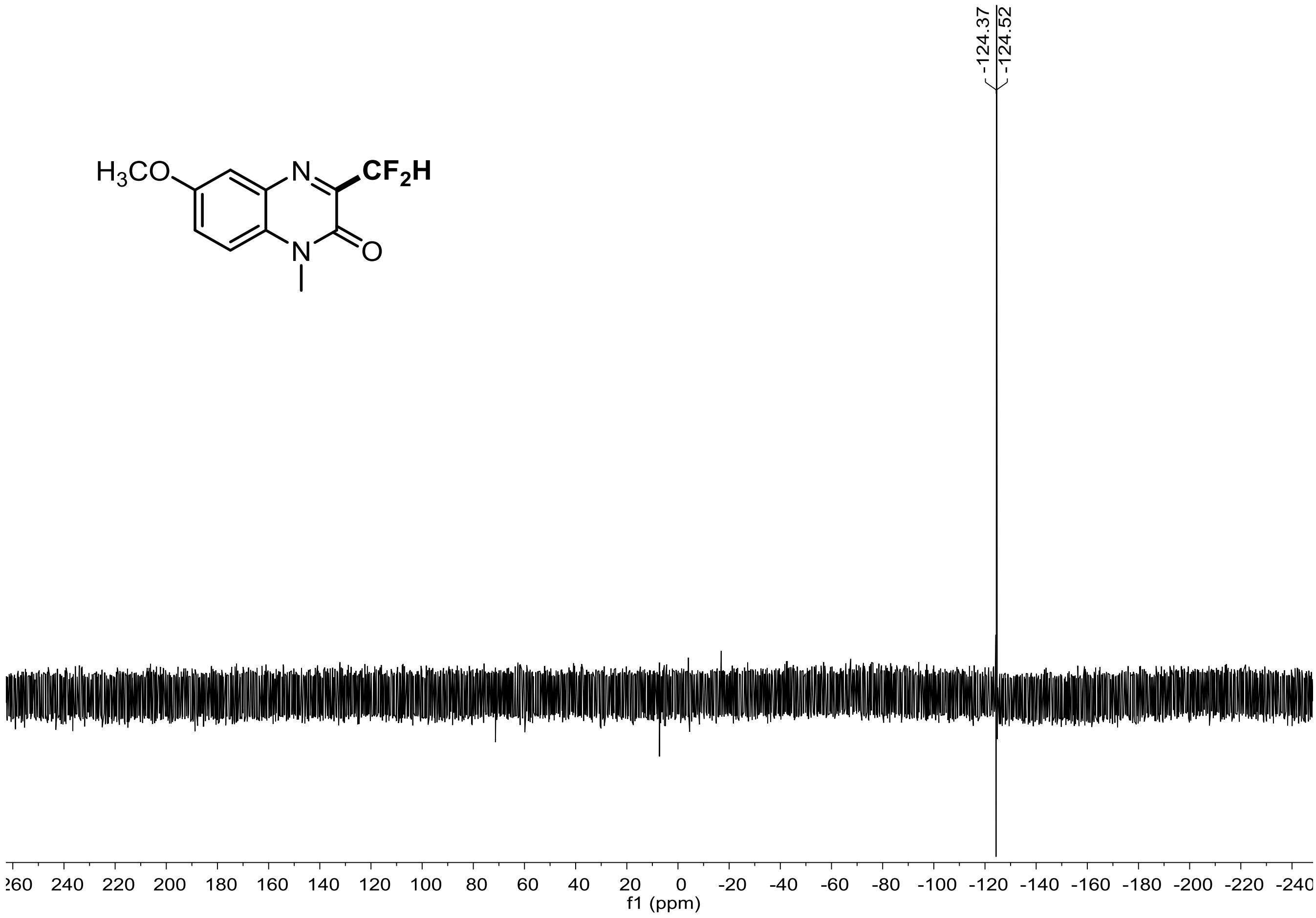

Supplementary Figure 26. ^{19}F NMR Spectrum of **3f**

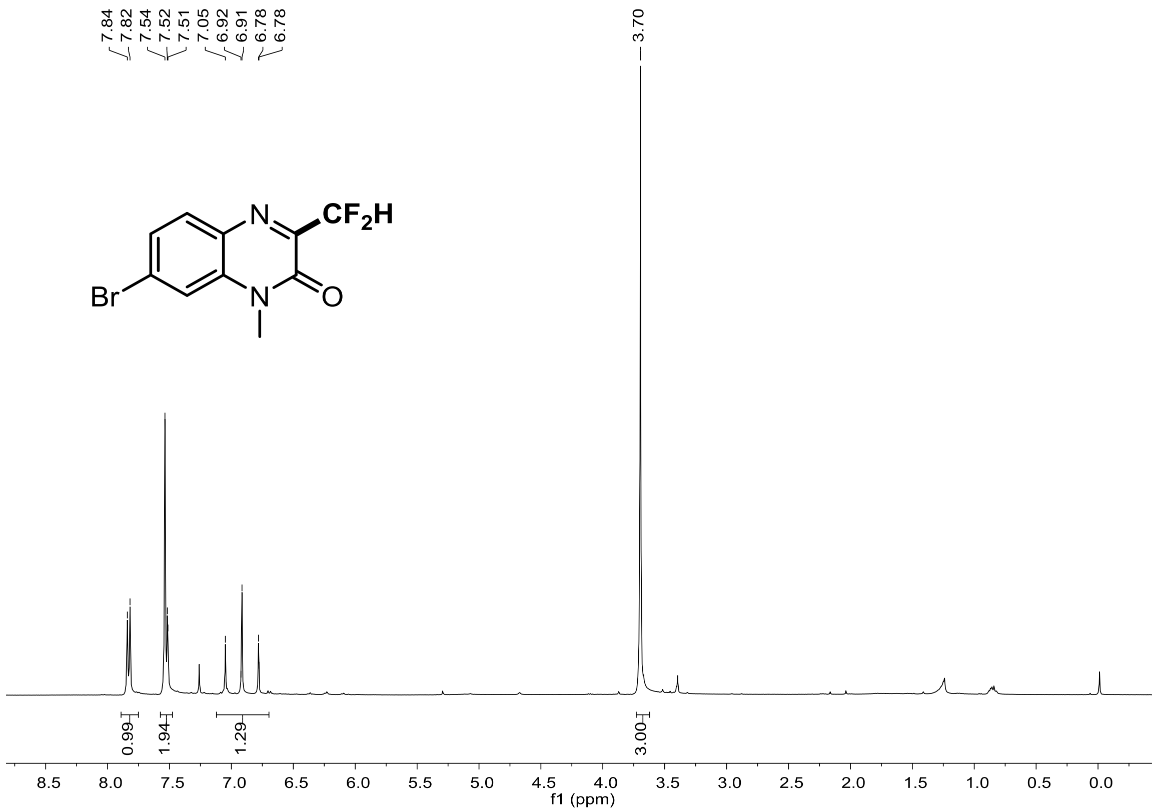

Supplementary Figure 27. ^{1}H NMR Spectrum of **3g**

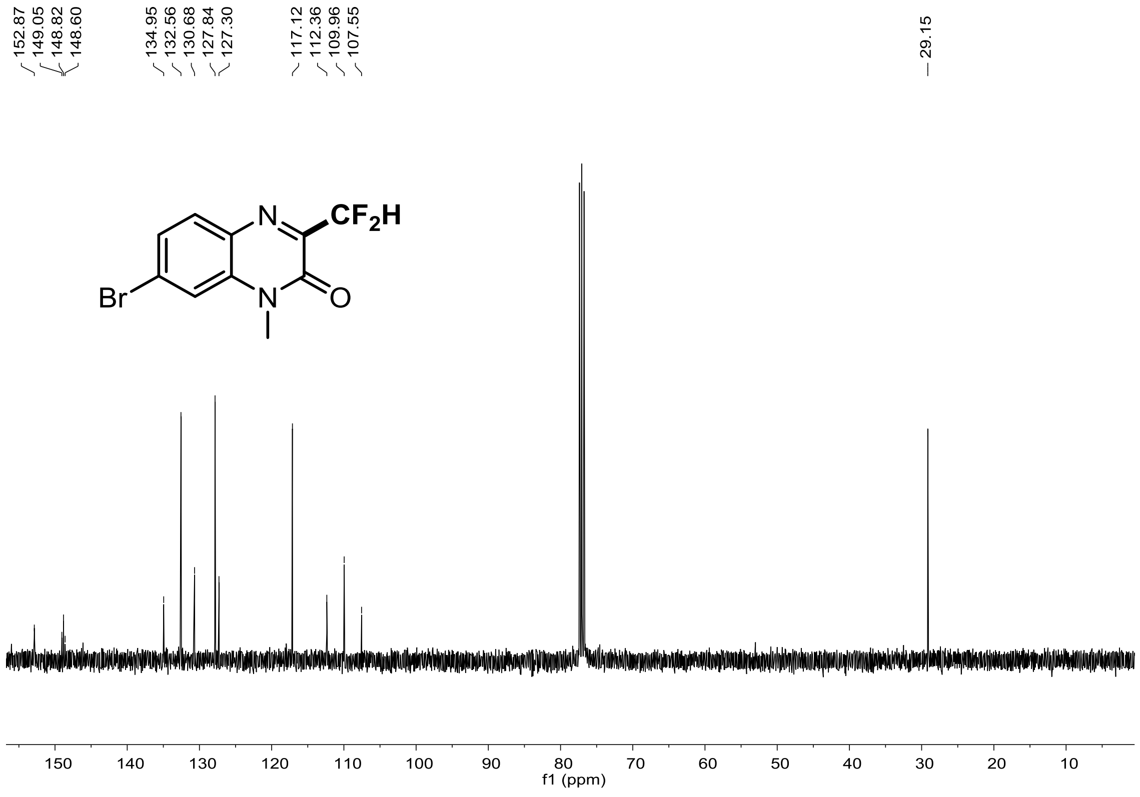

Supplementary Figure 28. ^{13}C NMR Spectrum of **3g**

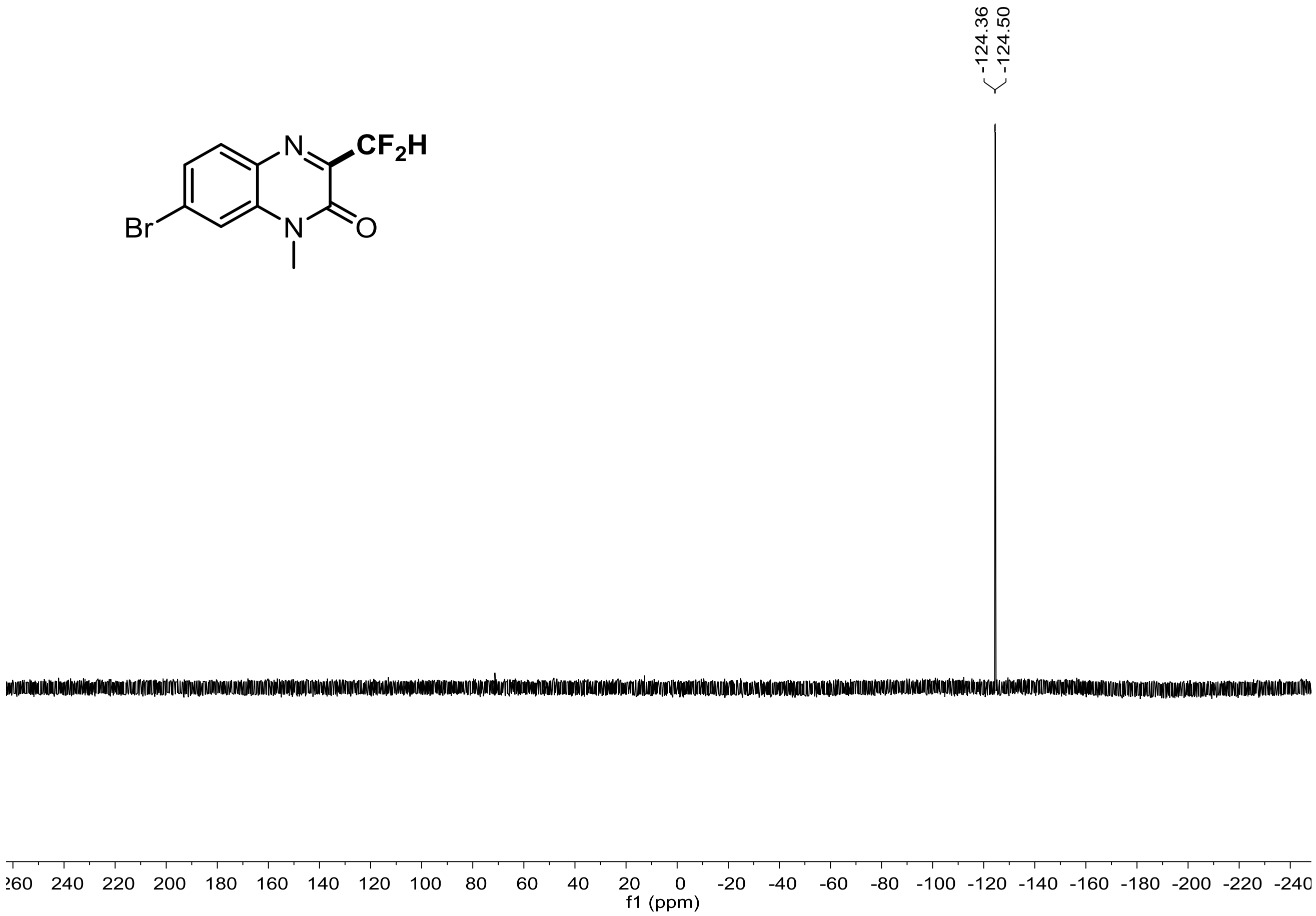

Supplementary Figure 29. ^{19}F NMR Spectrum of **3g**

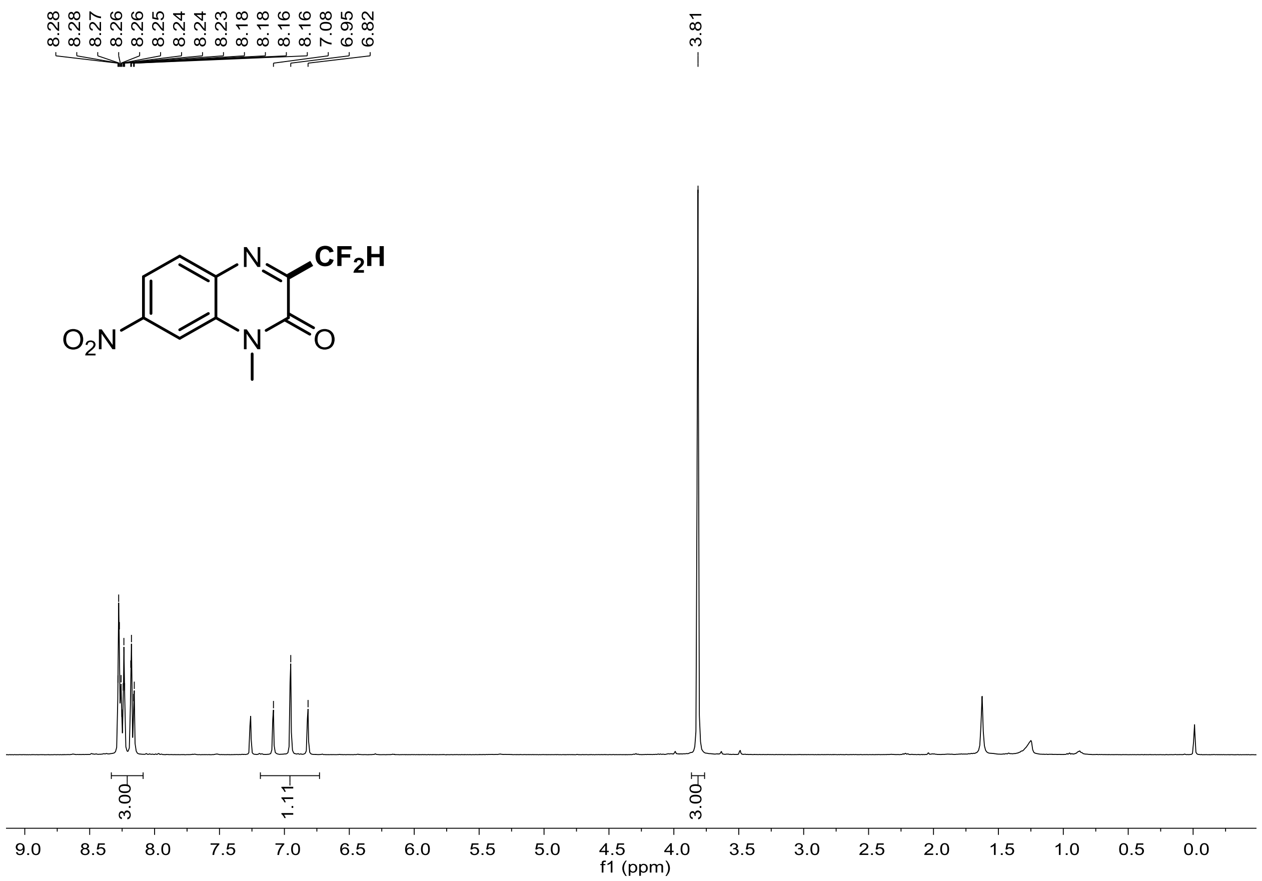

Supplementary Figure 30.^{1}H NMR Spectrum of **3h**

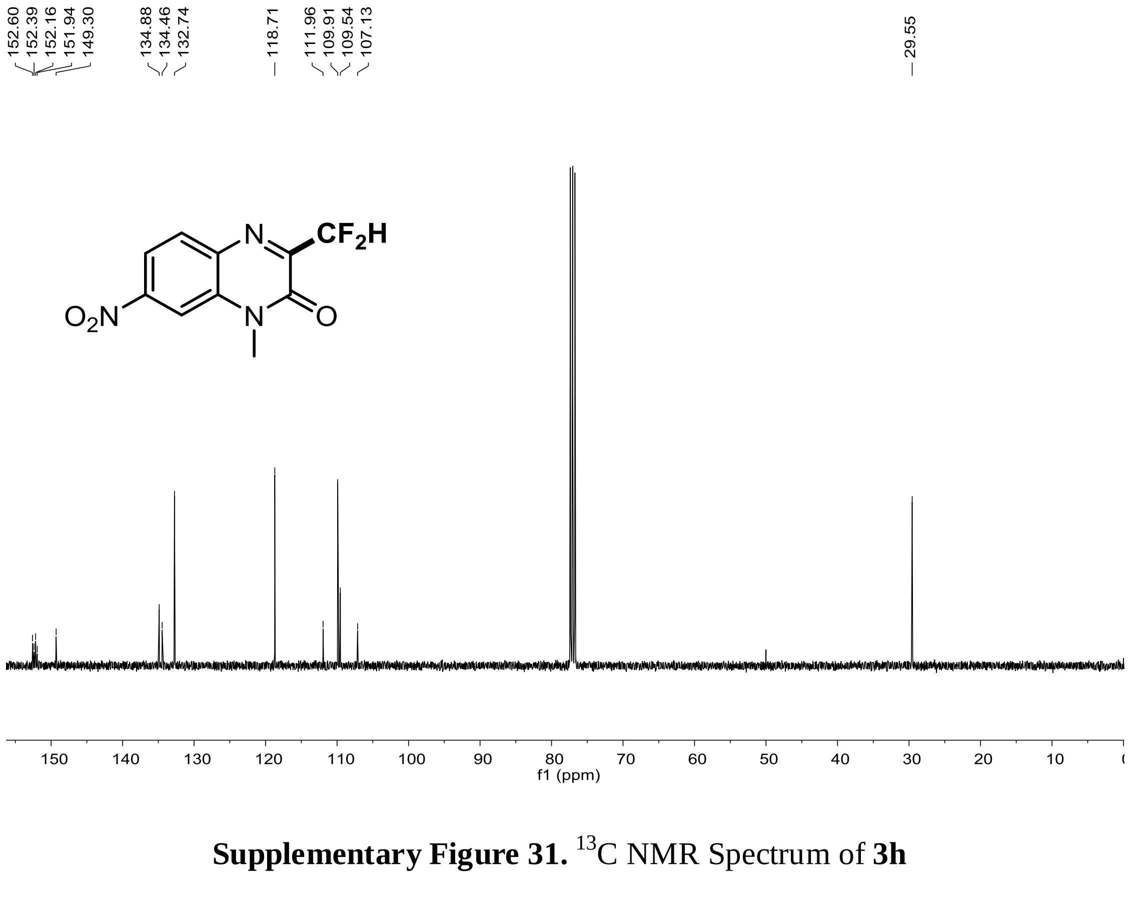

Supplementary Figure 31. ^{13}C NMR Spectrum of **3h**

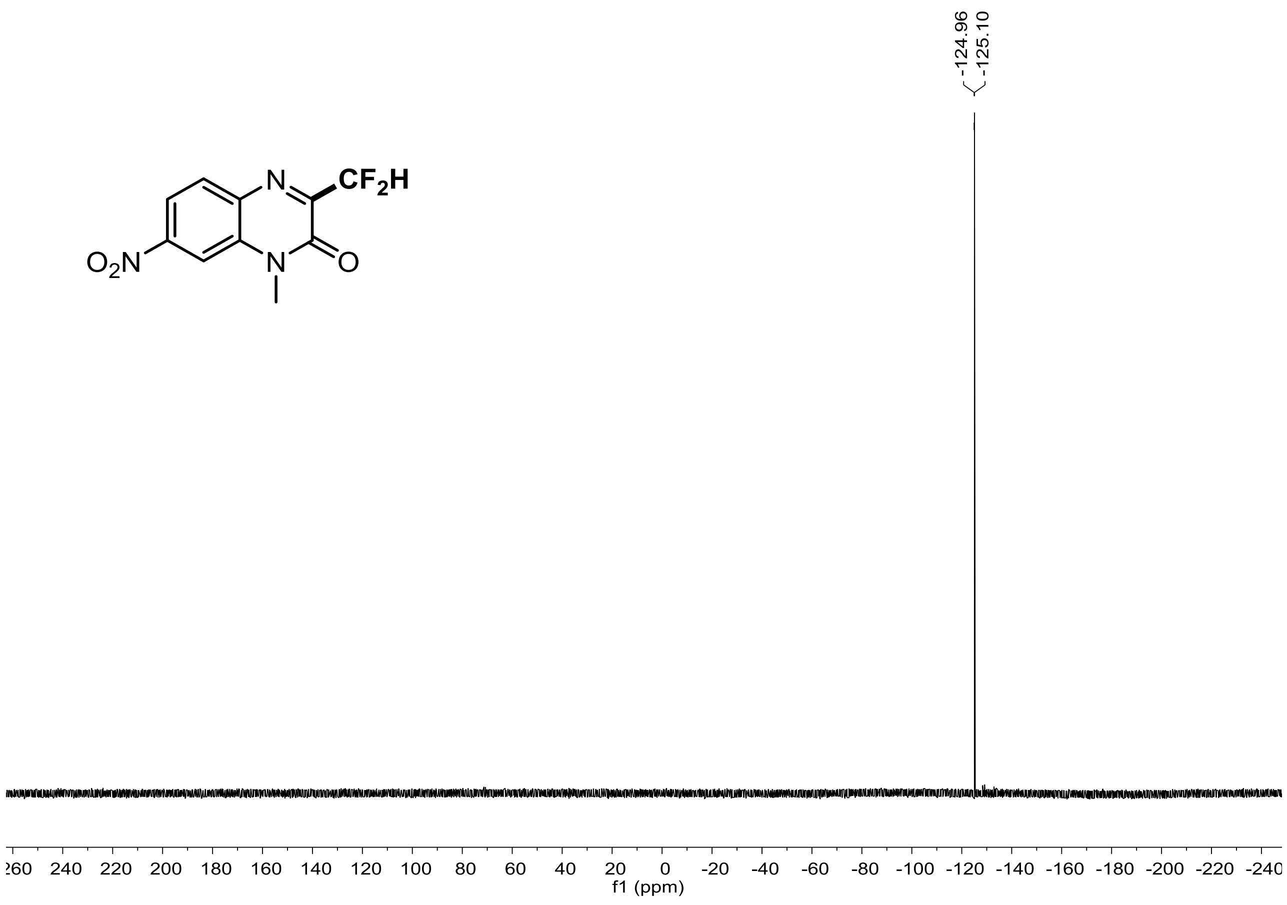

Supplementary Figure 32. ^{19}F NMR Spectrum of **3h**

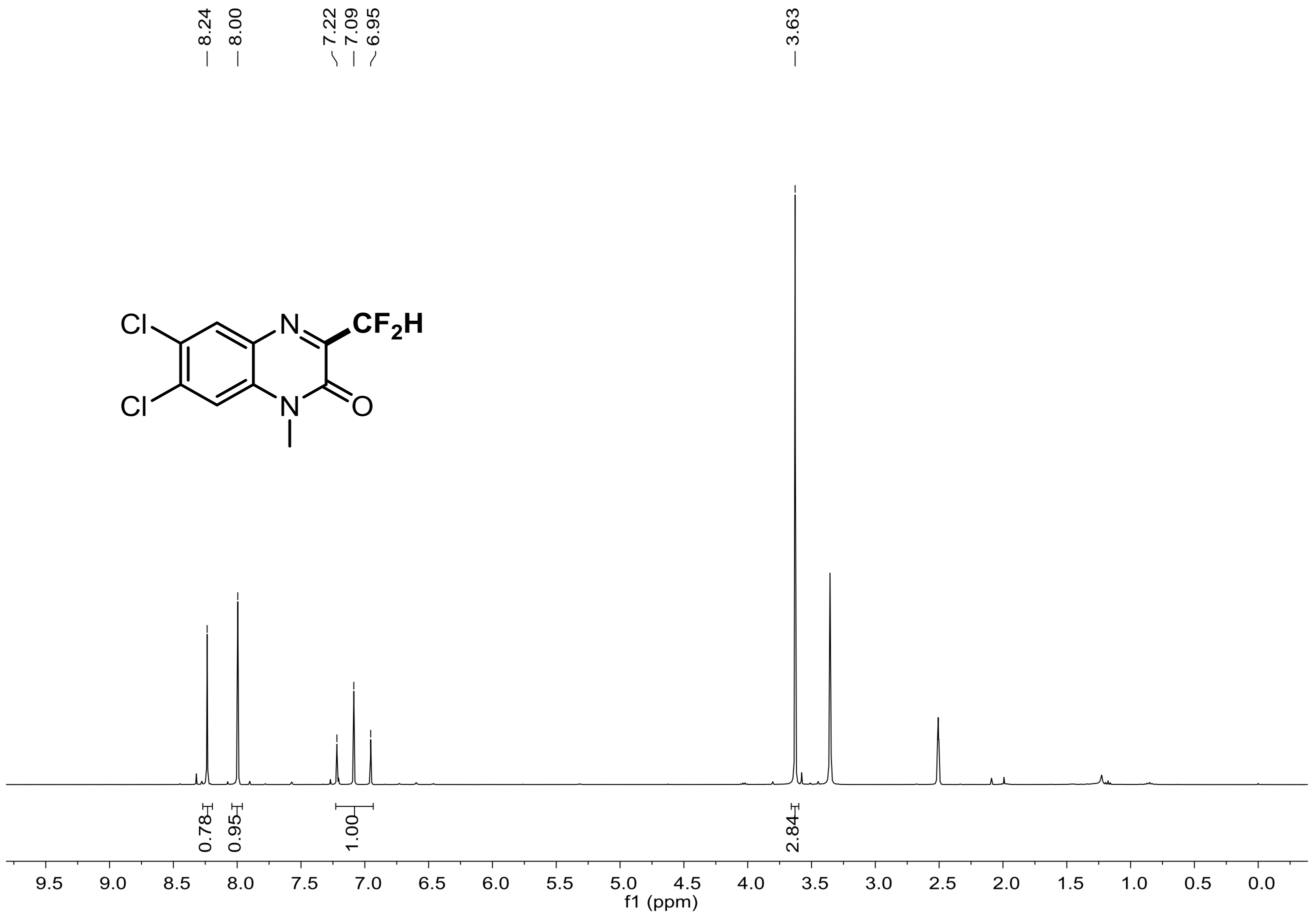

Supplementary Figure 33. ^{1}H NMR Spectrum of **3i**

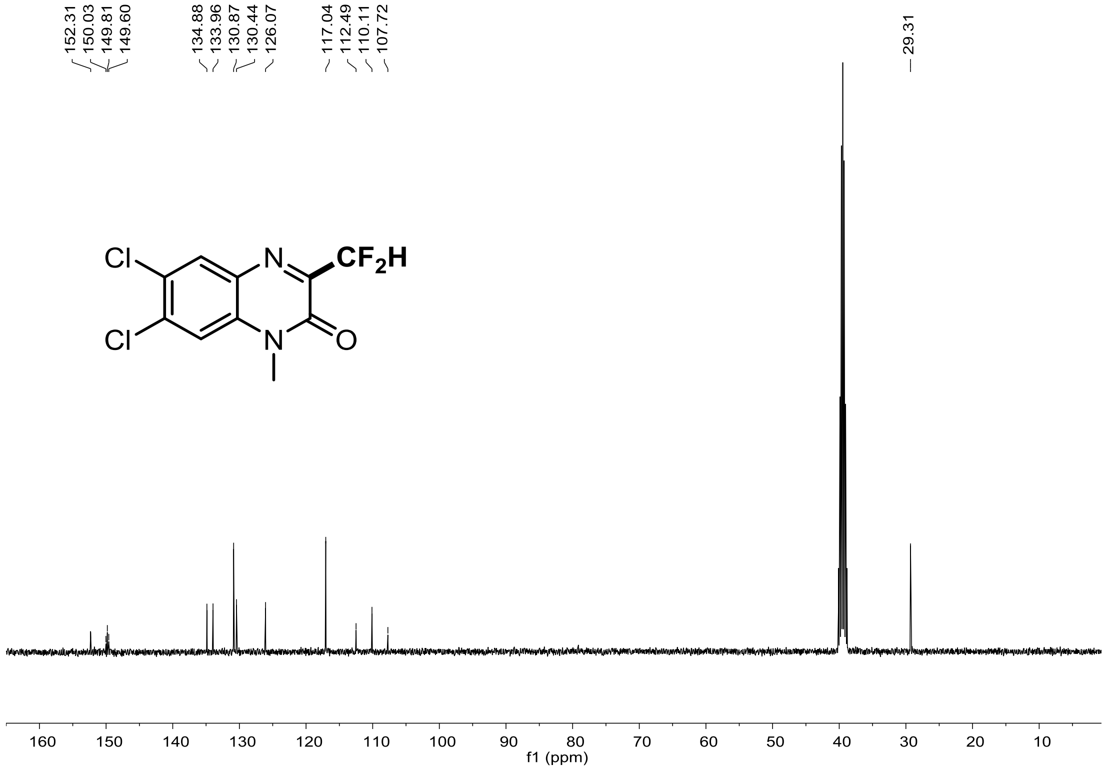

Supplementary Figure 34. ^{13}C NMR Spectrum of **3i**

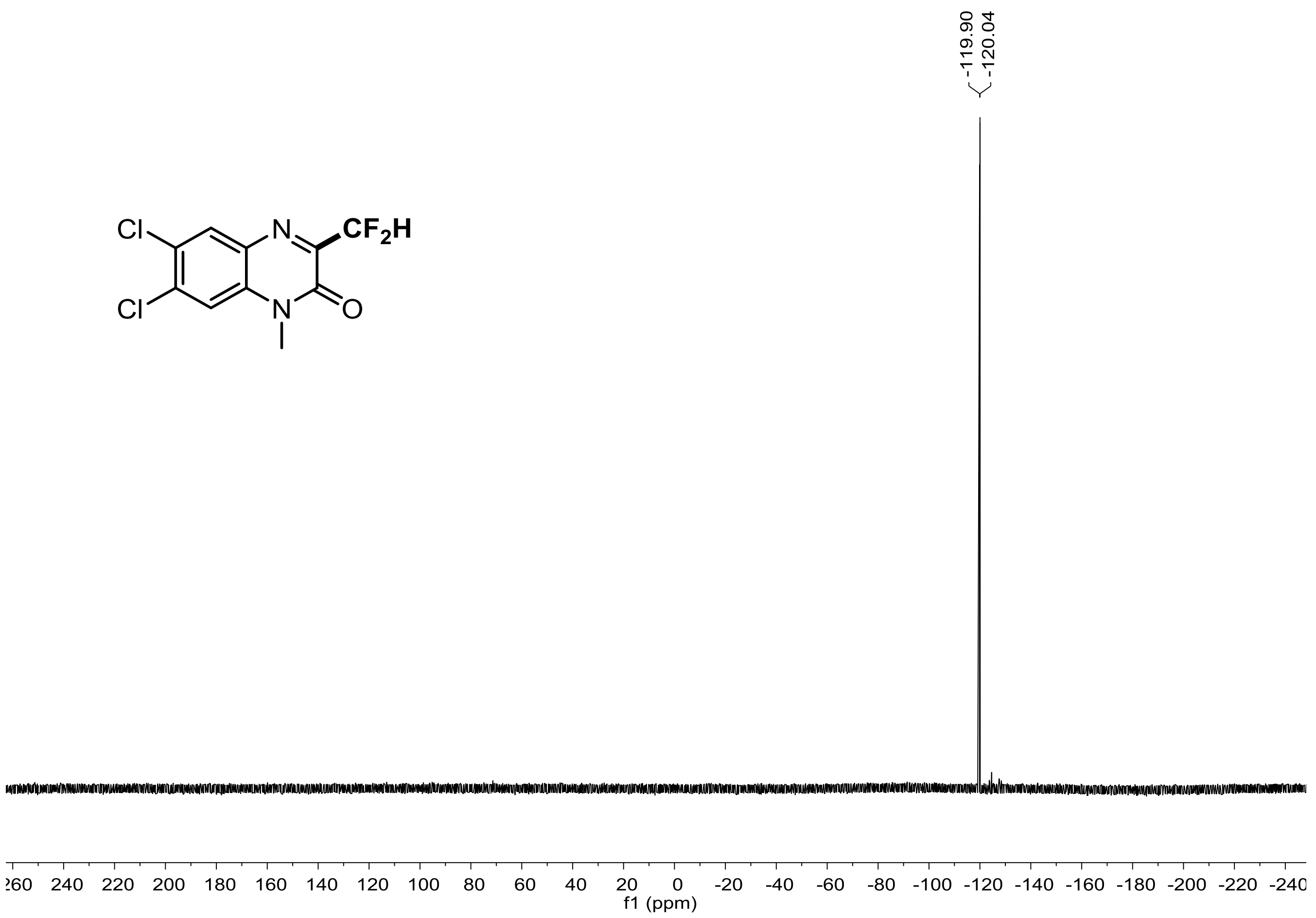

Supplementary Figure 35. ^{19}F NMR Spectrum of **3i**

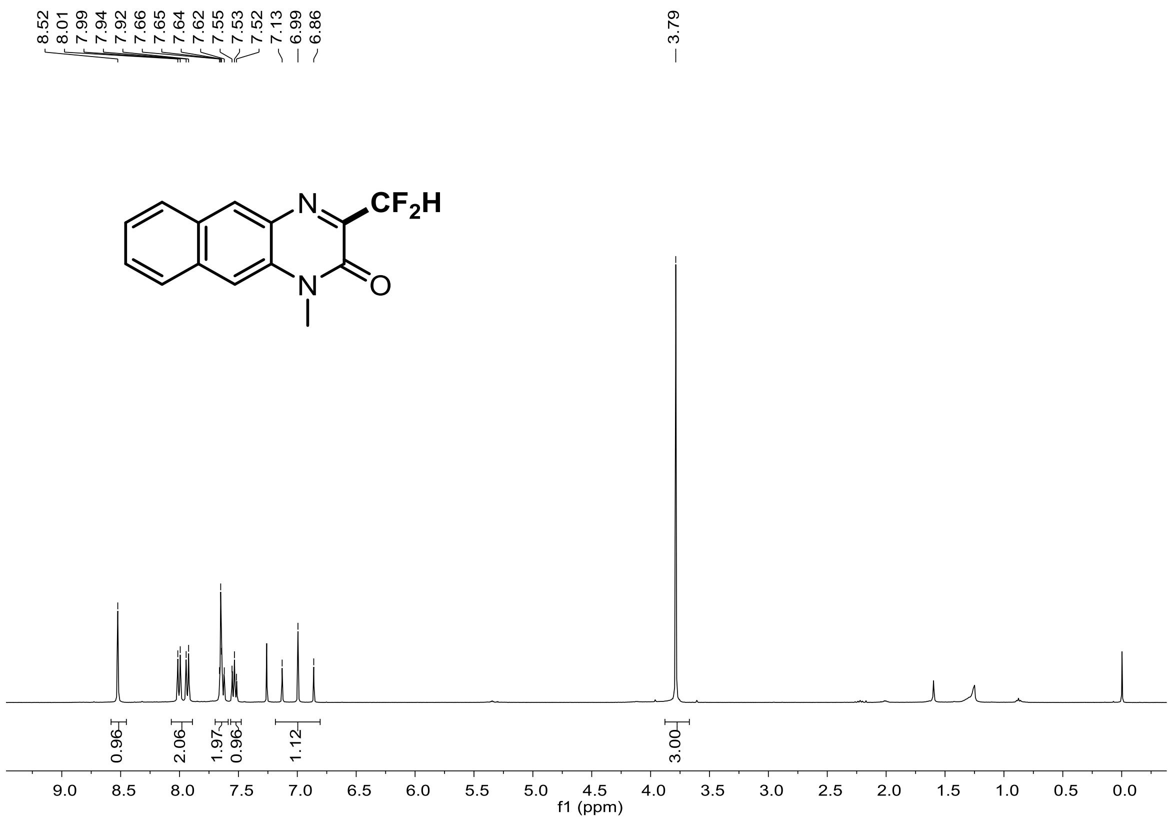

Supplementary Figure 36. ^{1}H NMR Spectrum of **3j**

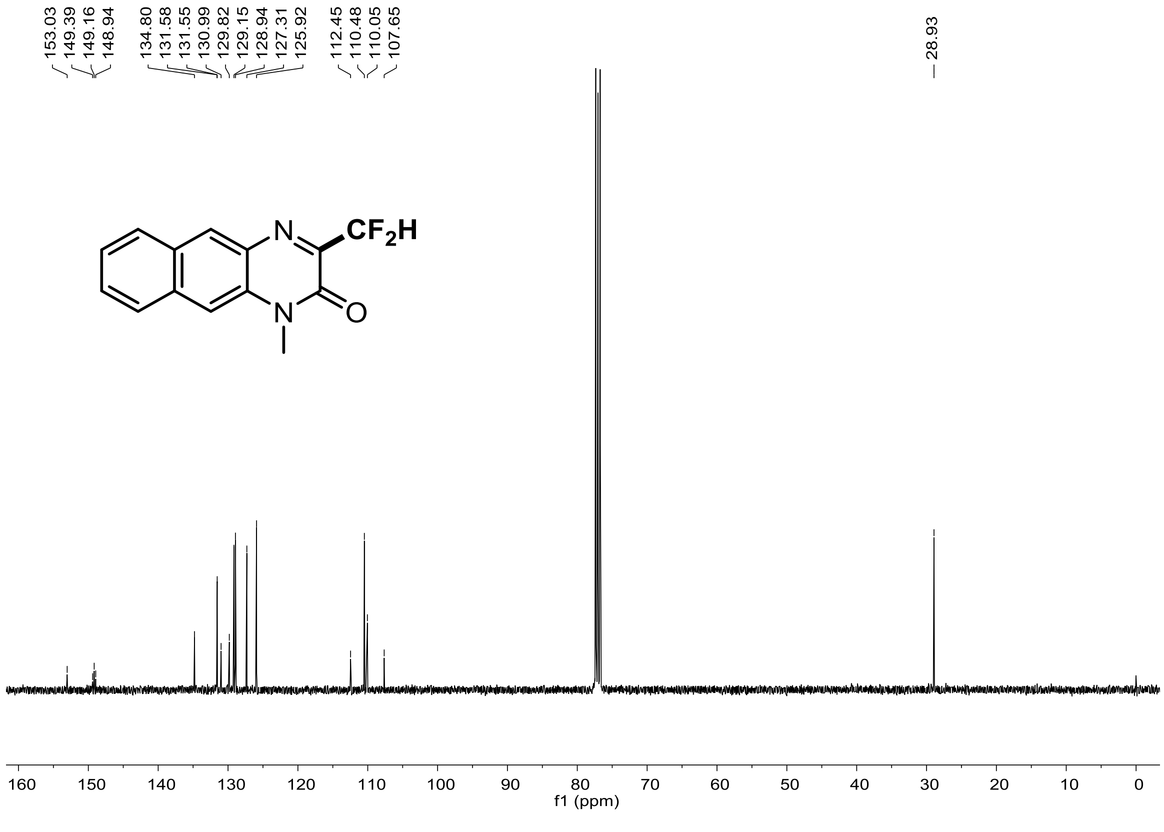

Supplementary Figure 37. ^{13}C NMR Spectrum of **3j**

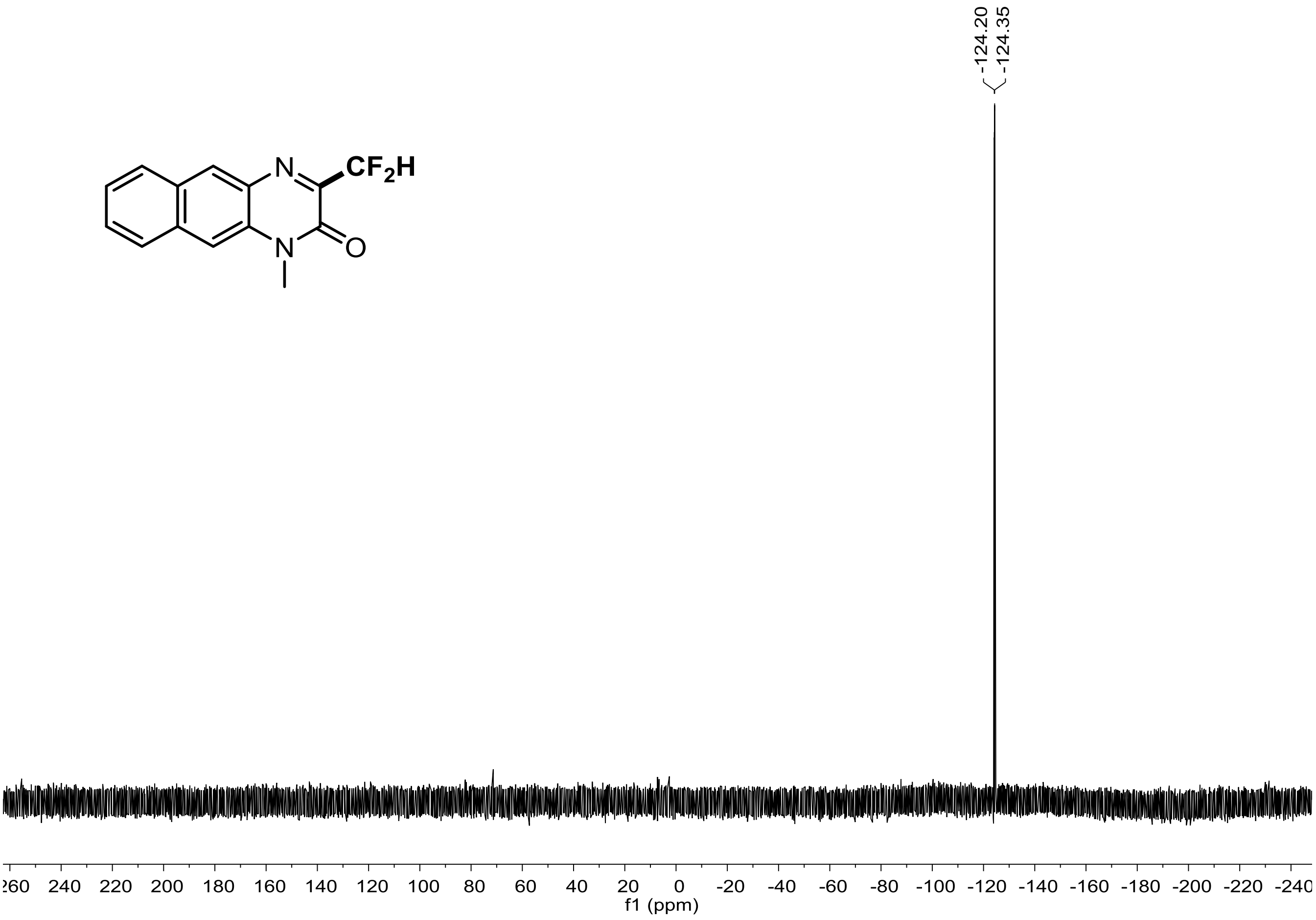

Supplementary Figure 38. ^{19}F NMR Spectrum of **3j**

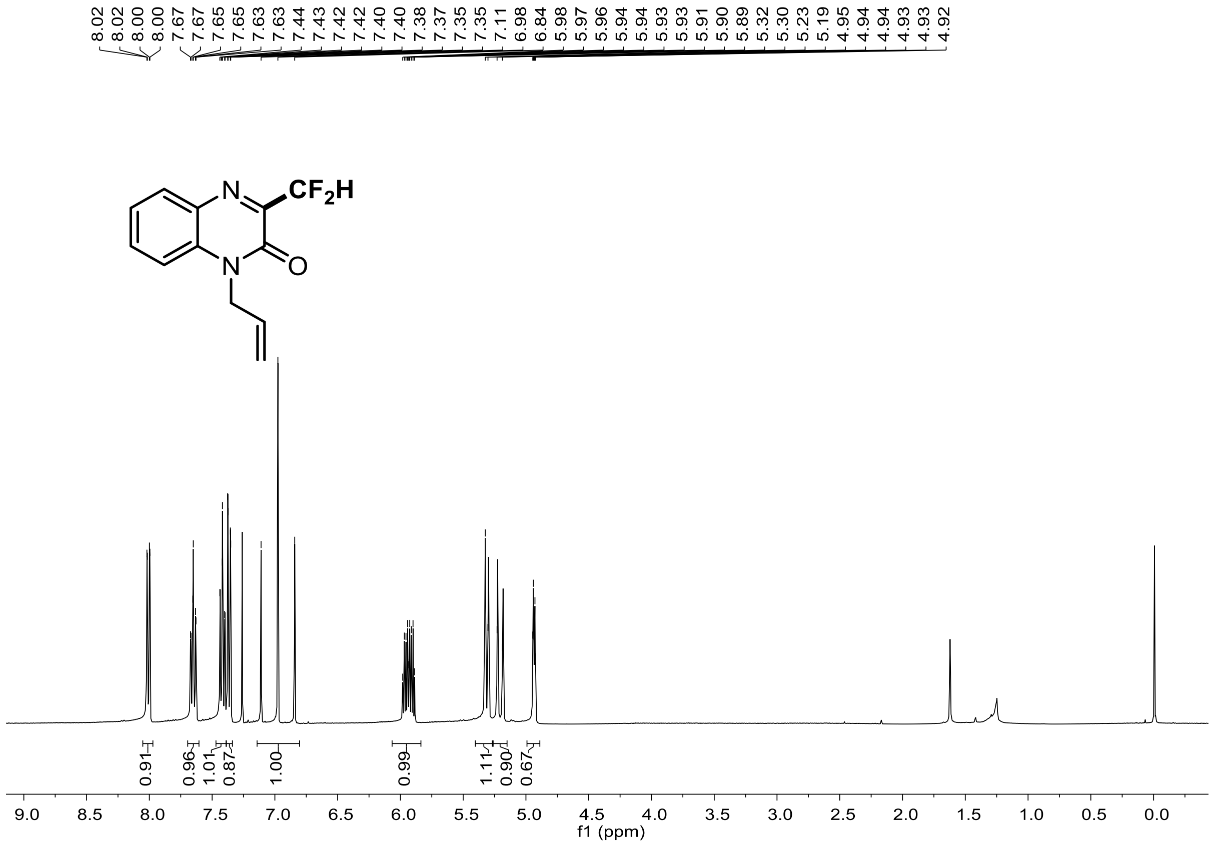

Supplementary Figure 39. ^{1}H NMR Spectrum of **3k**

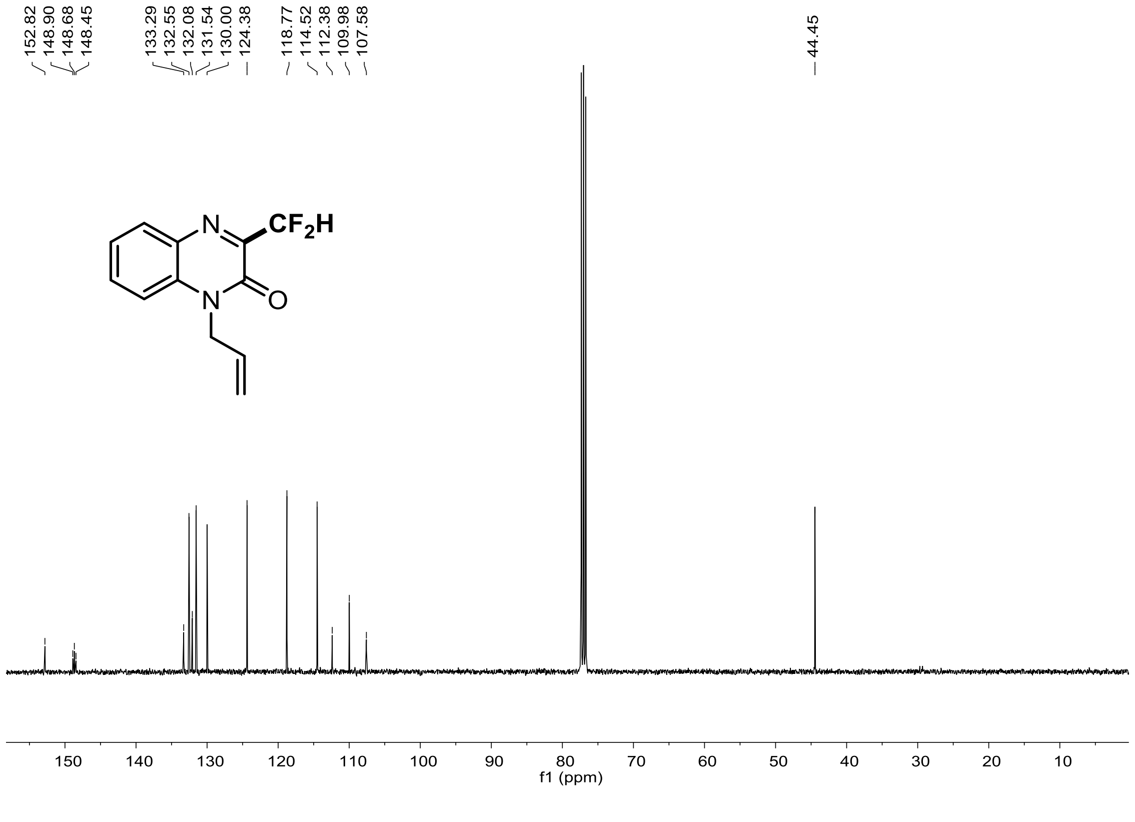

Supplementary Figure 40. ^{13}C NMR Spectrum of **3k**

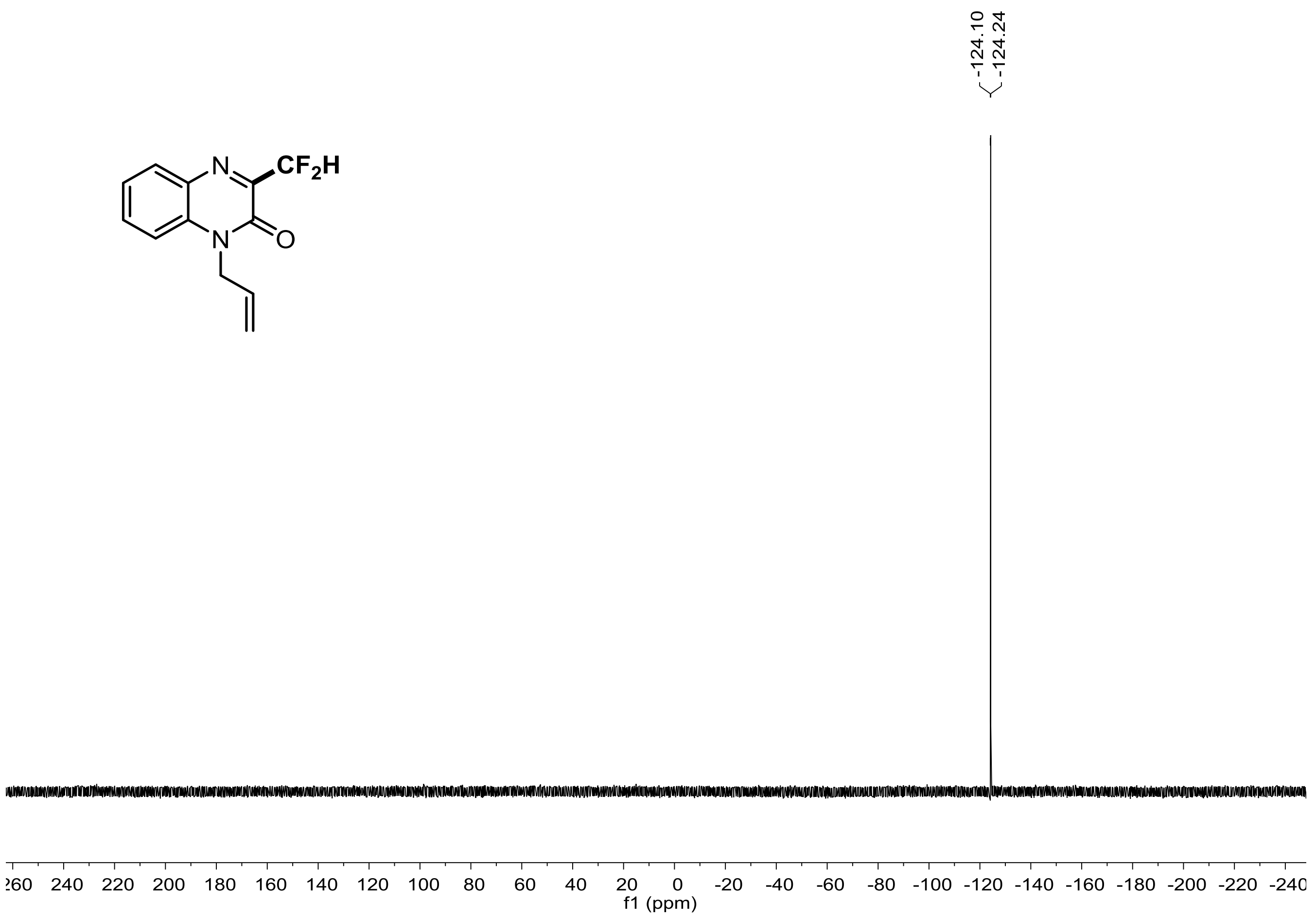

Supplementary Figure 41. ^{19}F NMR Spectrum of **3k**

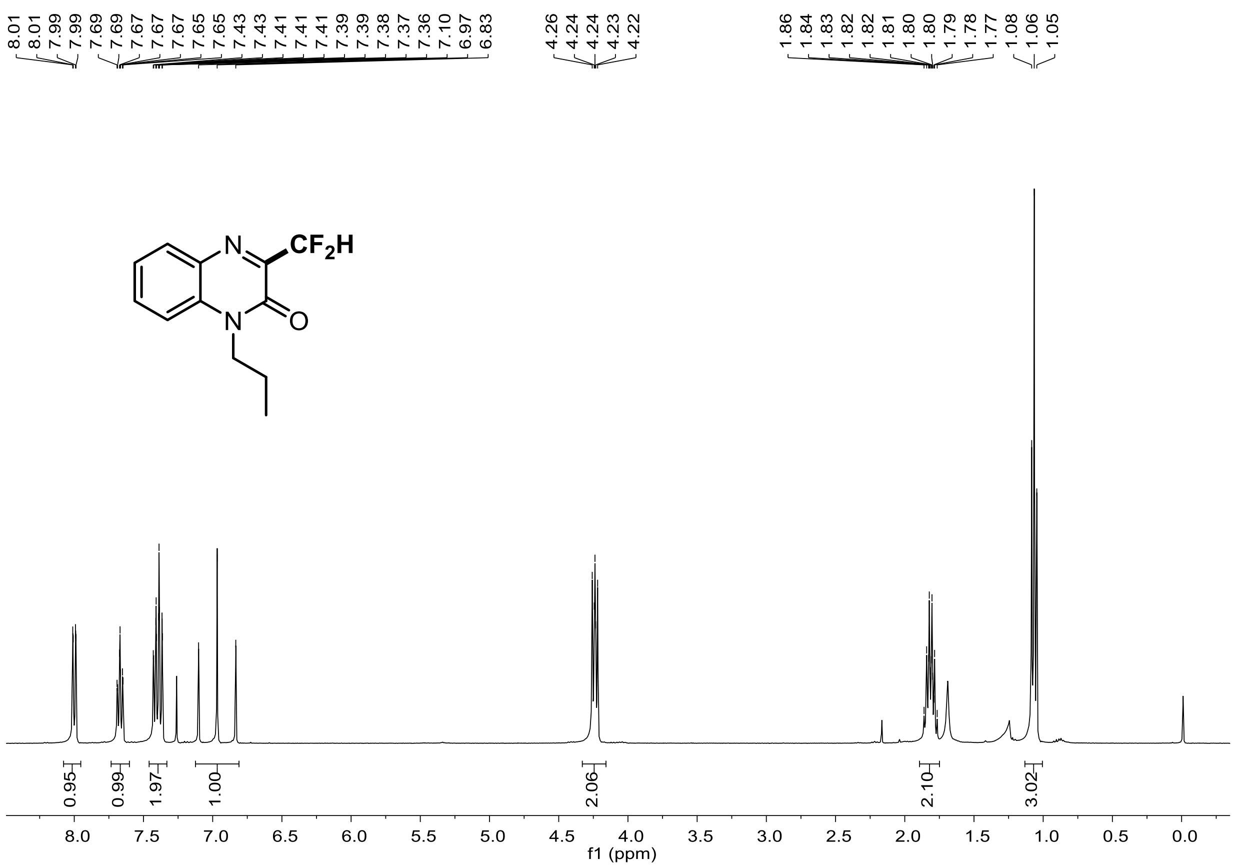

Supplementary Figure 42. ^{1}H NMR Spectrum of **3l**

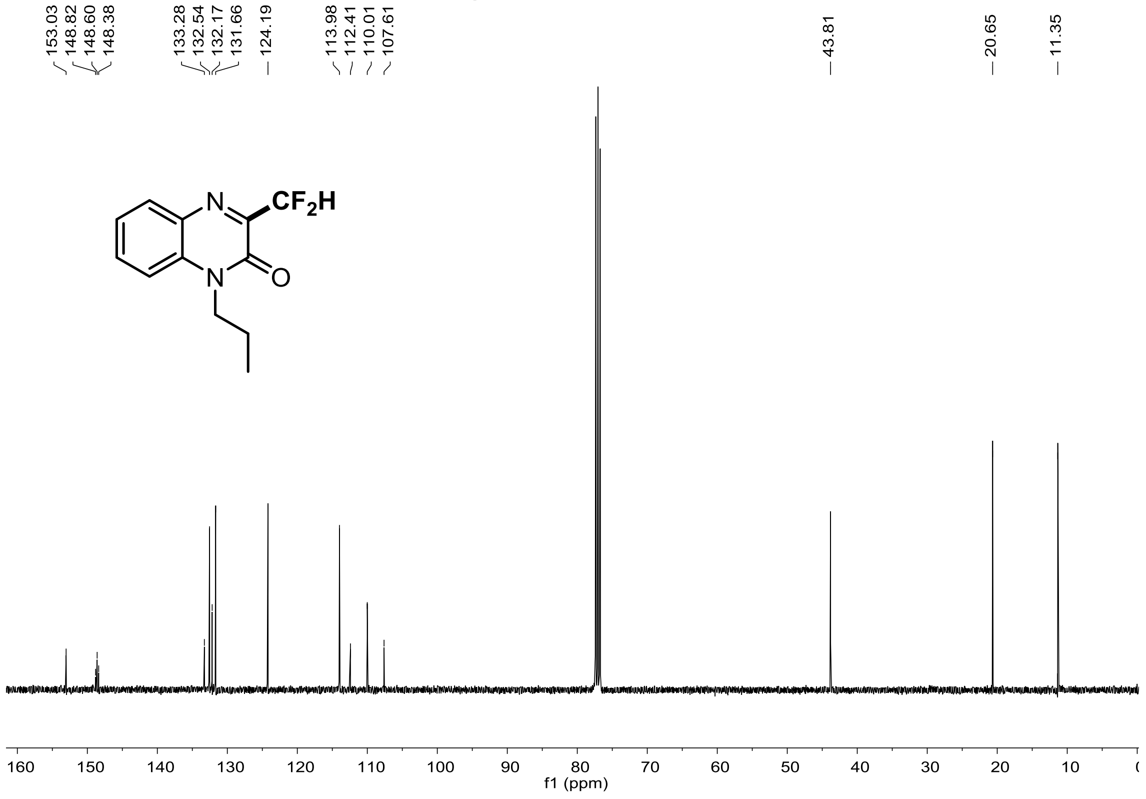

Supplementary Figure 43. ^{13}C NMR Spectrum of **3l**

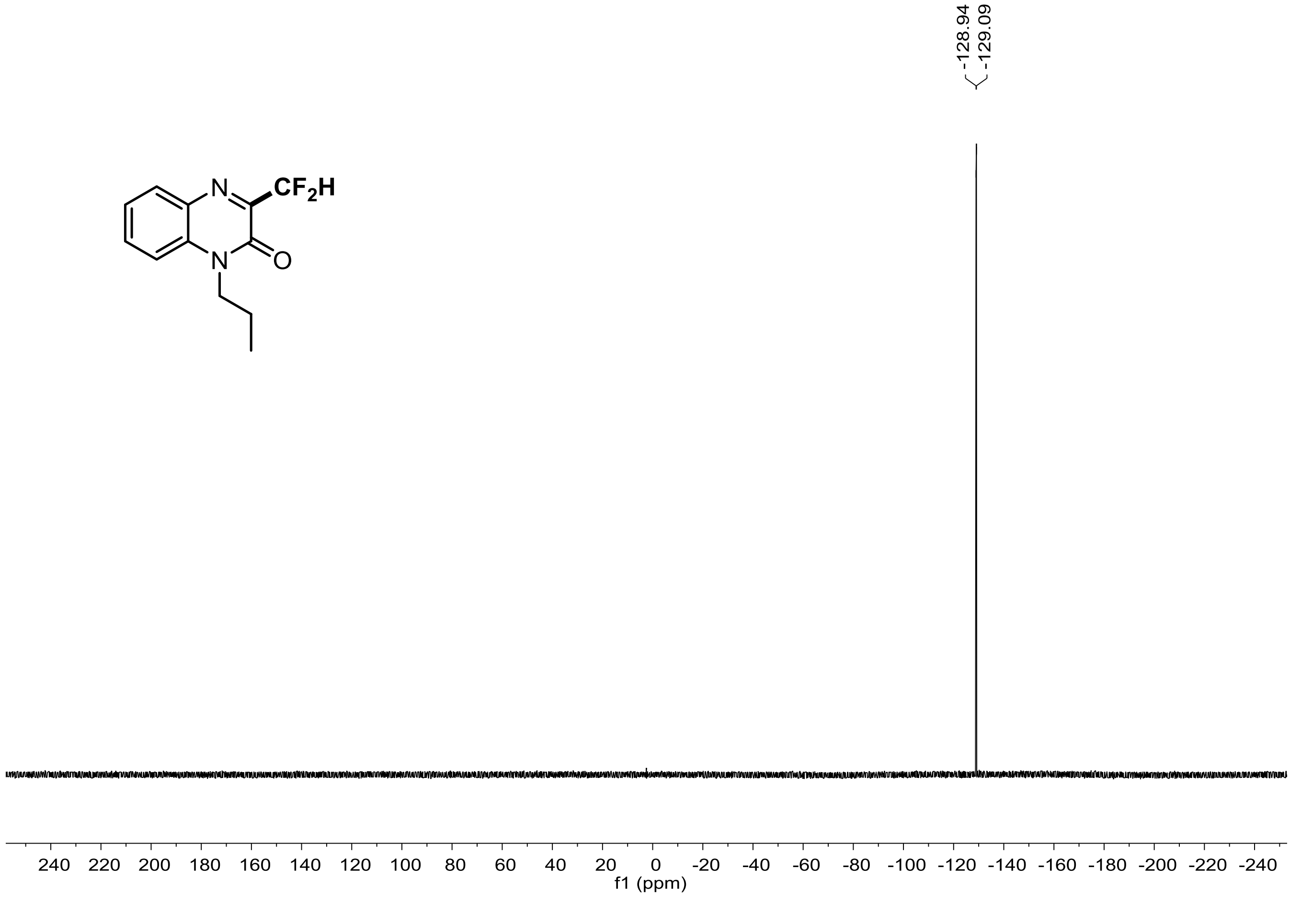

Supplementary Figure 44. ^{19}F NMR Spectrum of **3l**

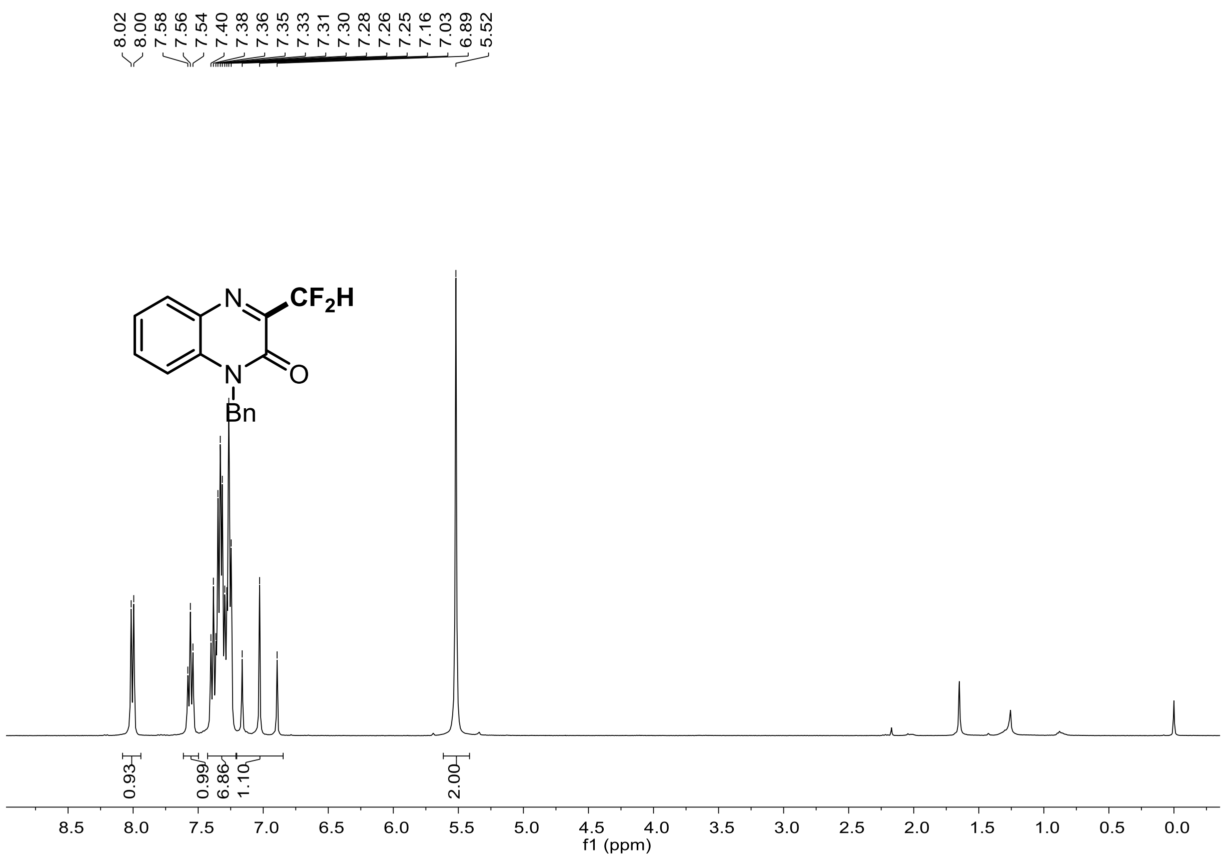

Supplementary Figure 45. ^{1}H NMR Spectrum of **3m**

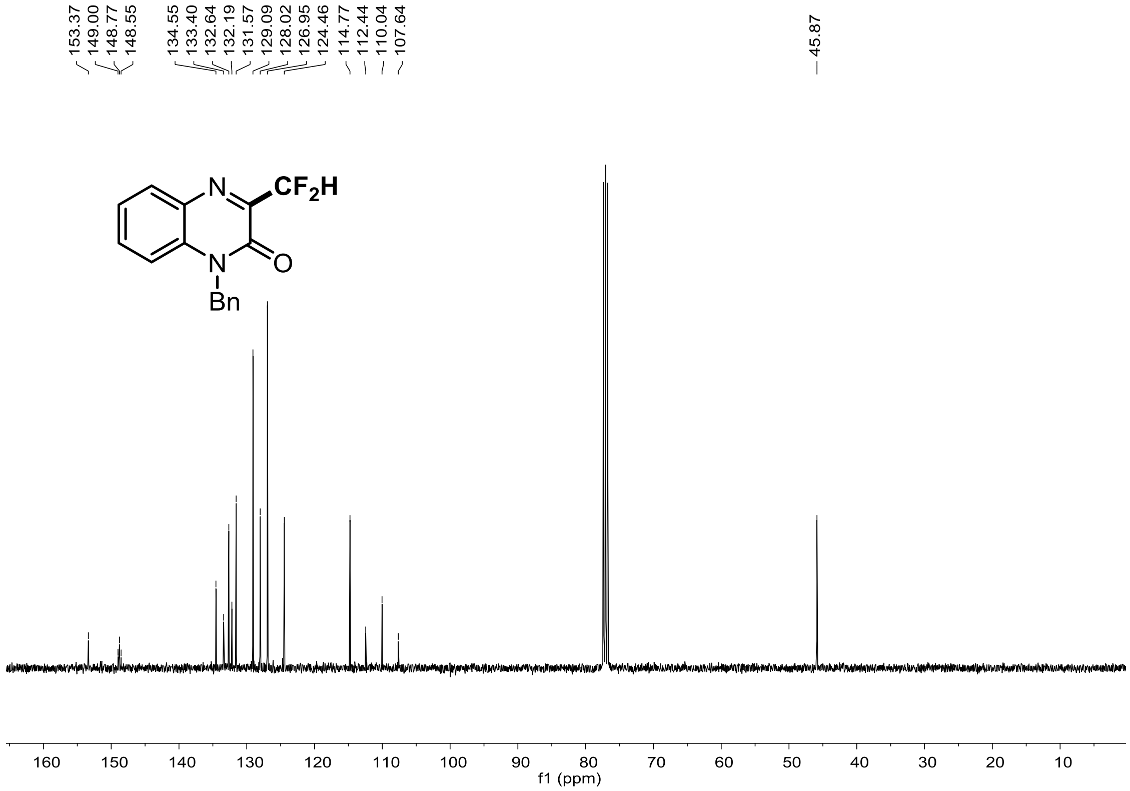

Supplementary Figure 46. ^{13}C NMR Spectrum of **3m**

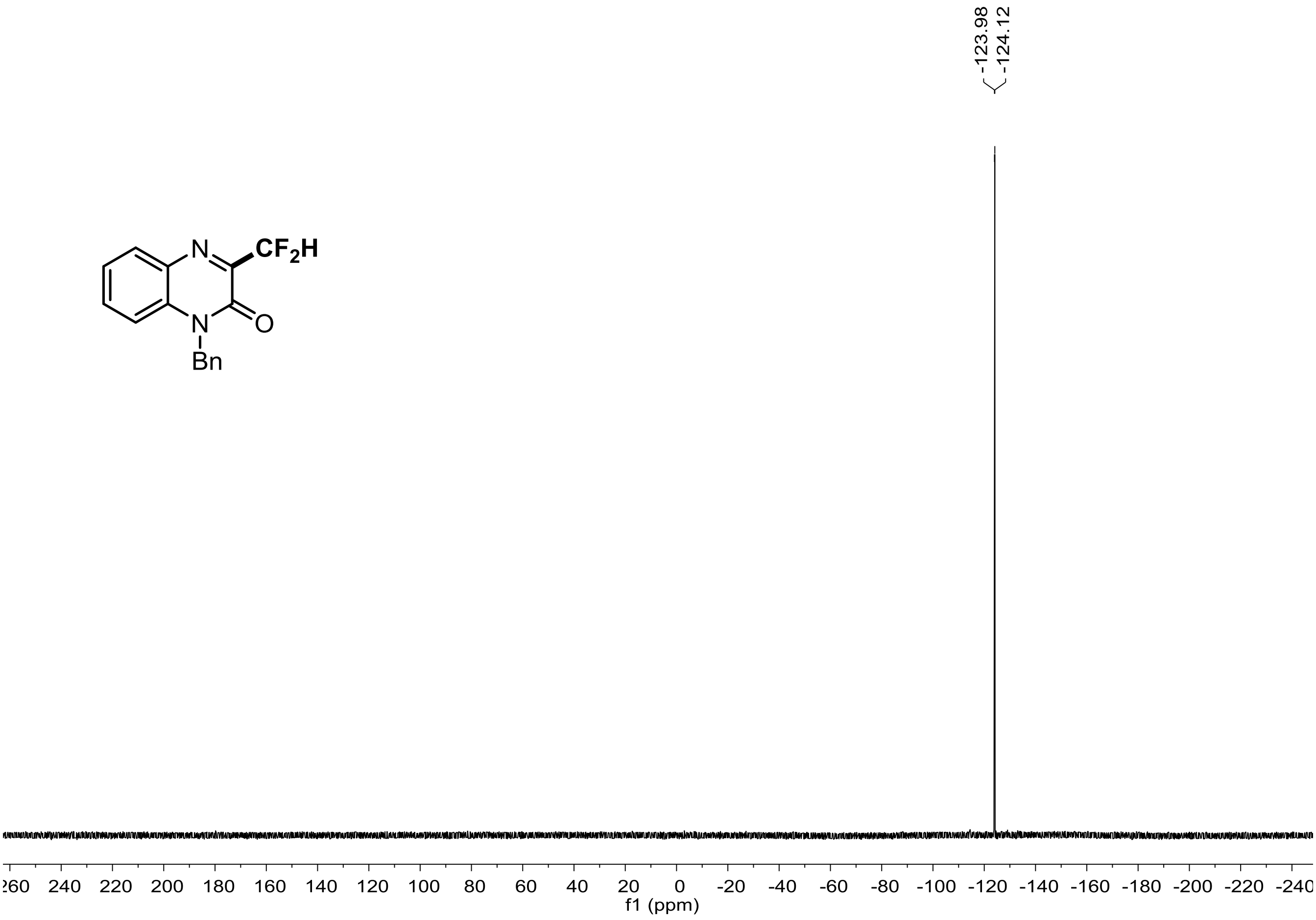

Supplementary Figure 47. ^{19}F NMR Spectrum of **3m**

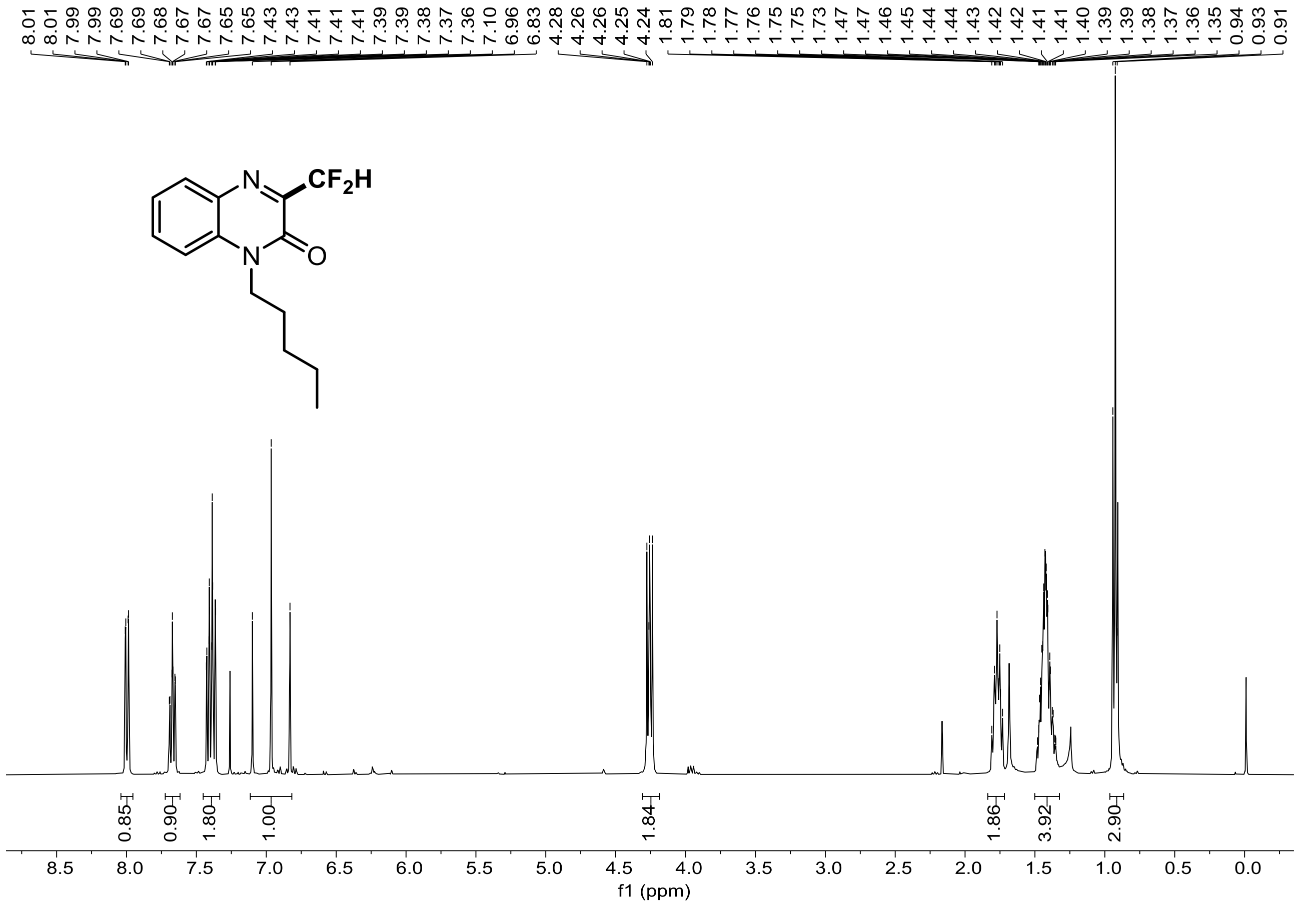

Supplementary Figure 48. ^{1}H NMR Spectrum of **3n**

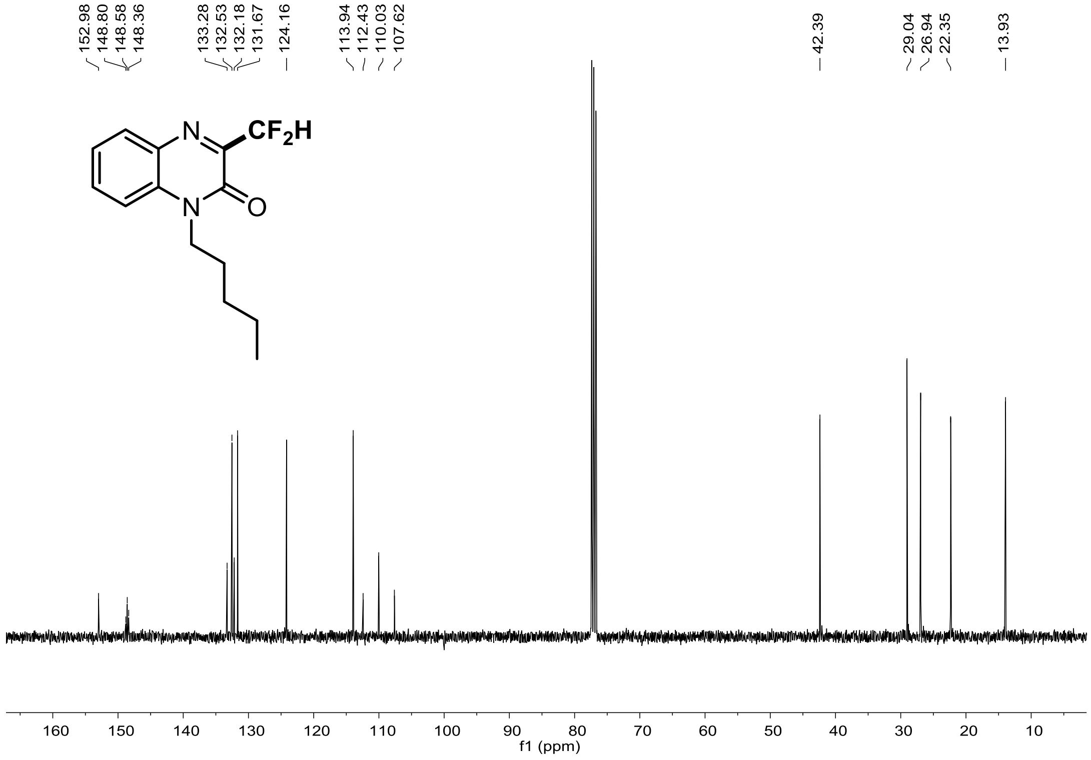

Supplementary Figure 49. ^{13}C NMR Spectrum of **3n**

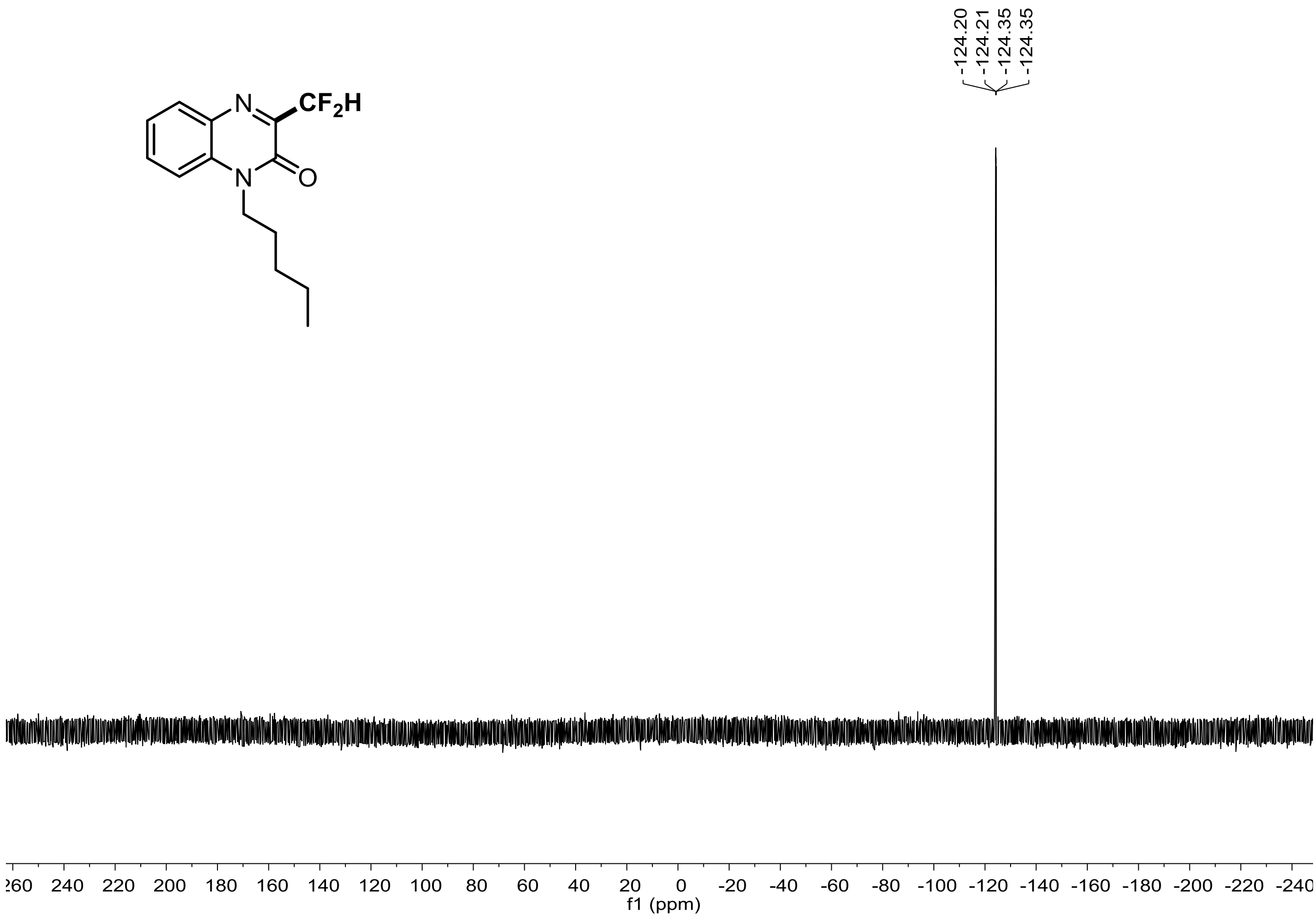

Supplementary Figure 50. ^{19}F NMR Spectrum of **3n**

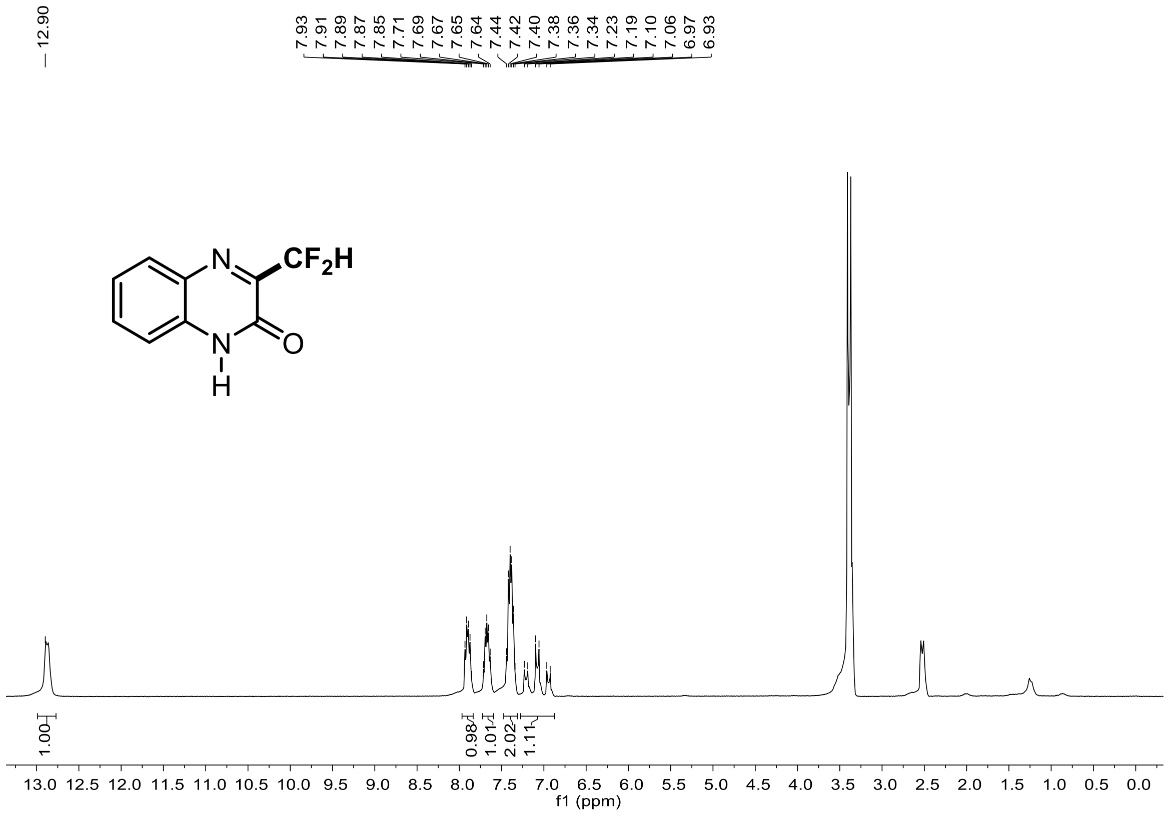

Supplementary Figure 51. ^{1}H NMR Spectrum of **3o**

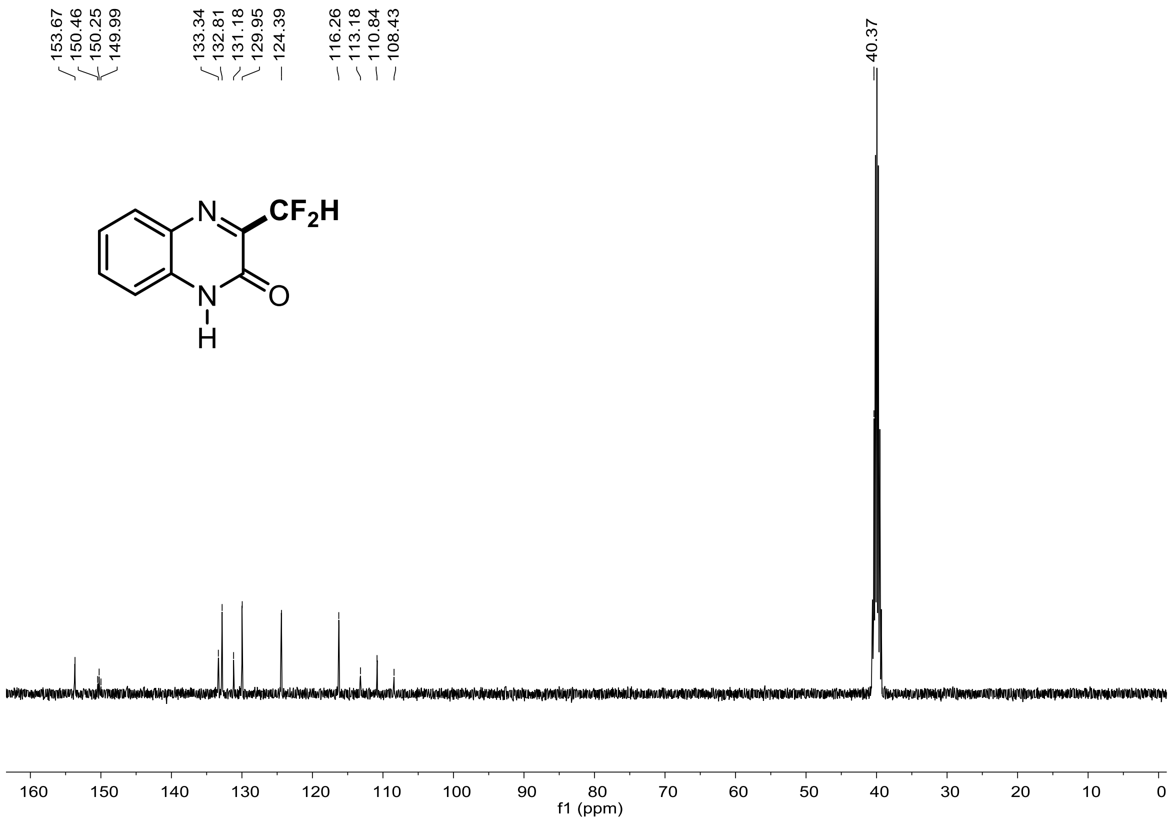

Supplementary Figure 52. ^{13}C NMR Spectrum of **3o**

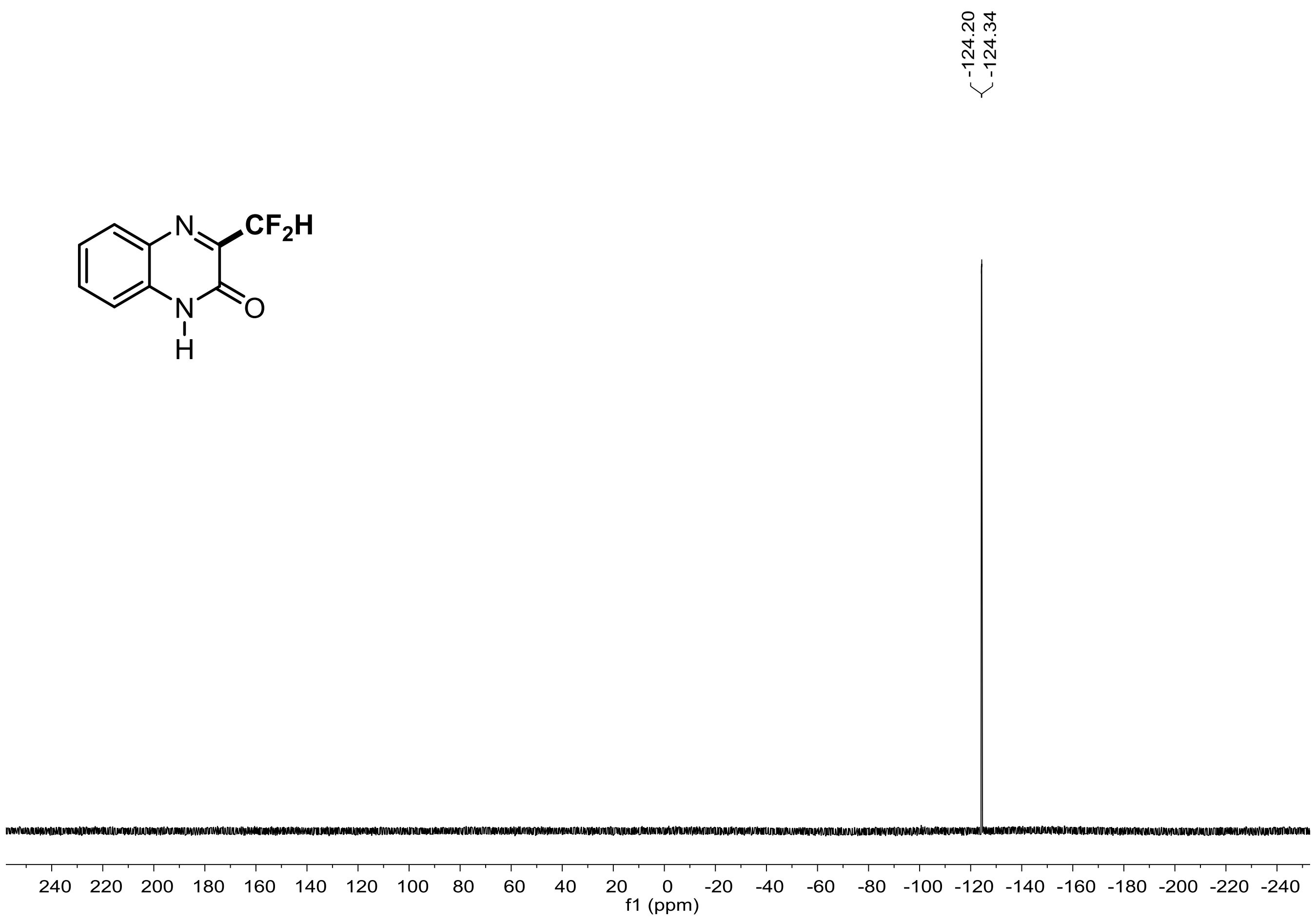

Supplementary Figure 53. ^{19}F NMR Spectrum of **3o**

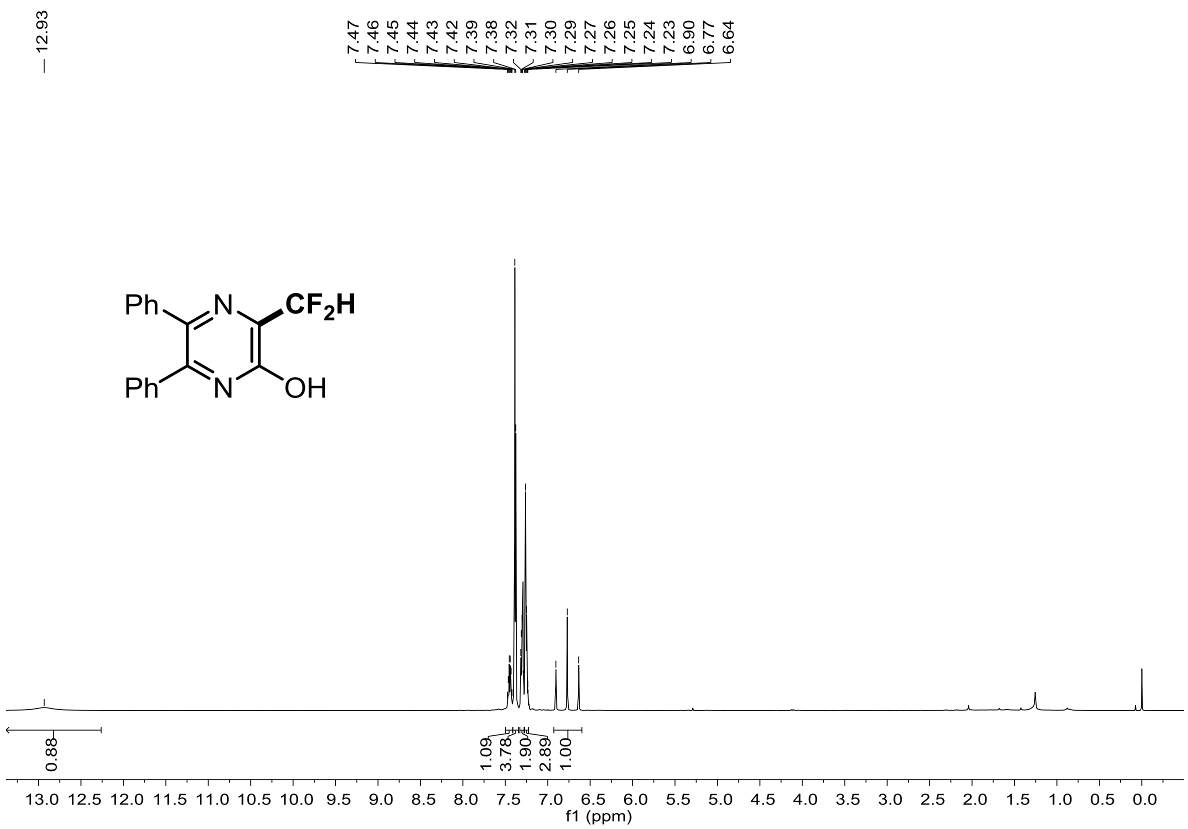

Supplementary Figure 54. ^{1}H NMR Spectrum of **5a**

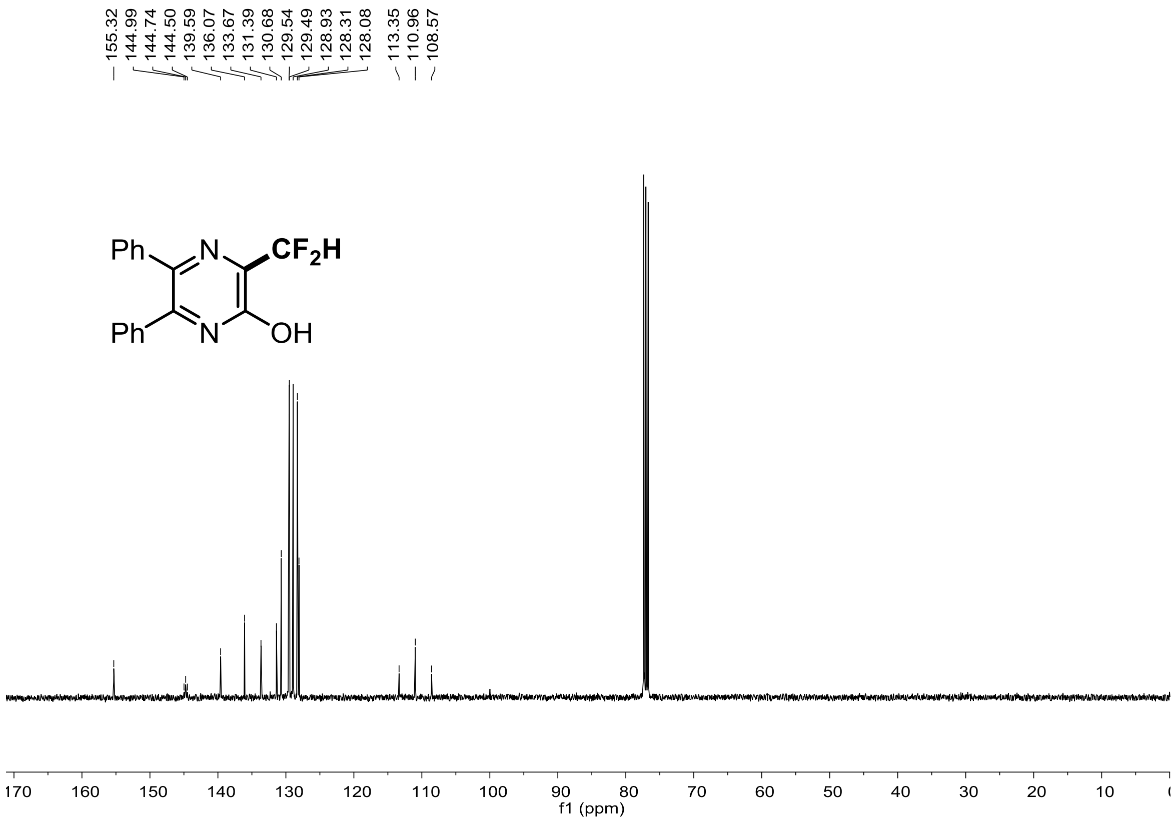

Supplementary Figure 55. ^{13}C NMR Spectrum of **5a**

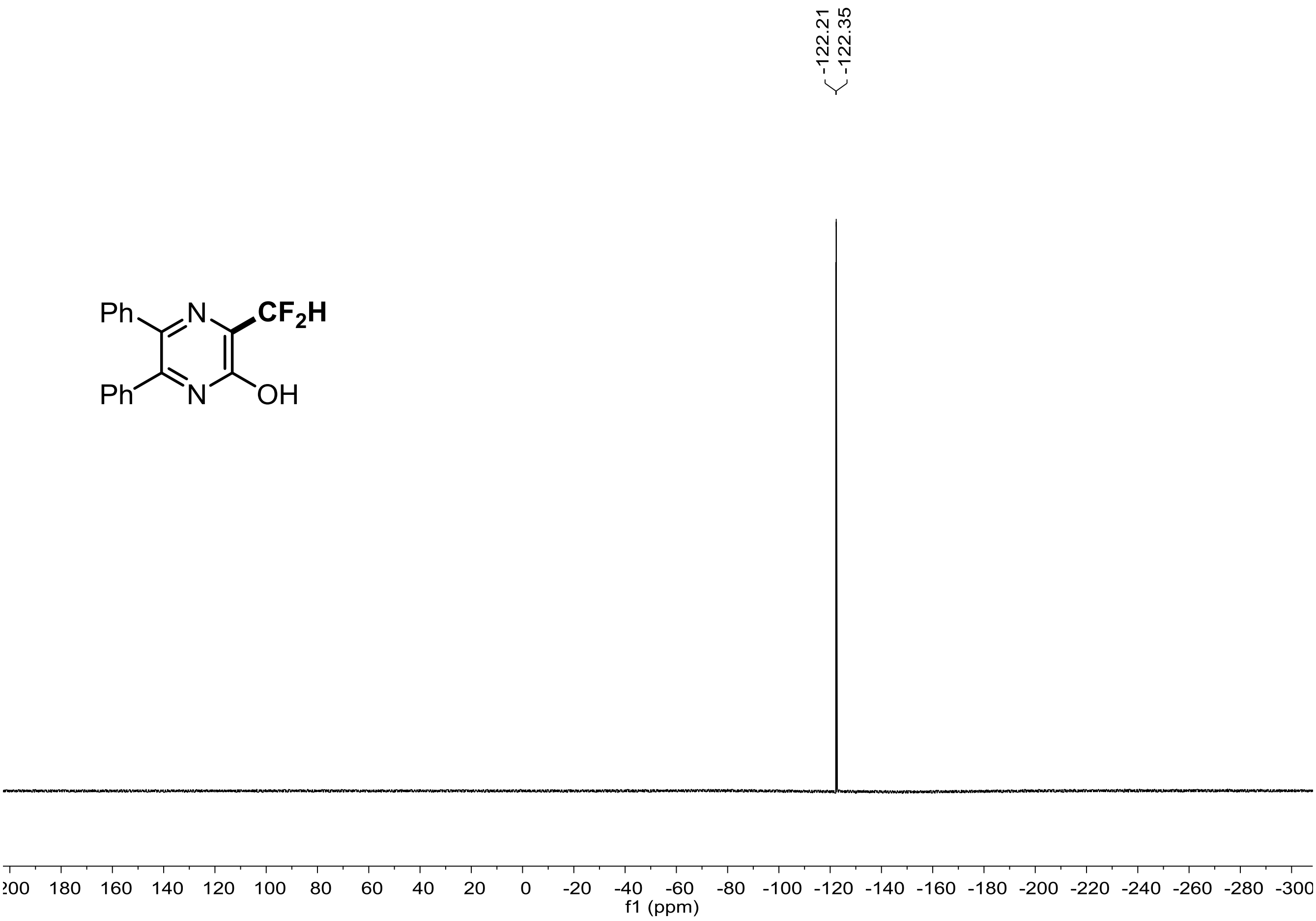

Supplementary Figure 56. ^{19}F NMR Spectrum of **5a**

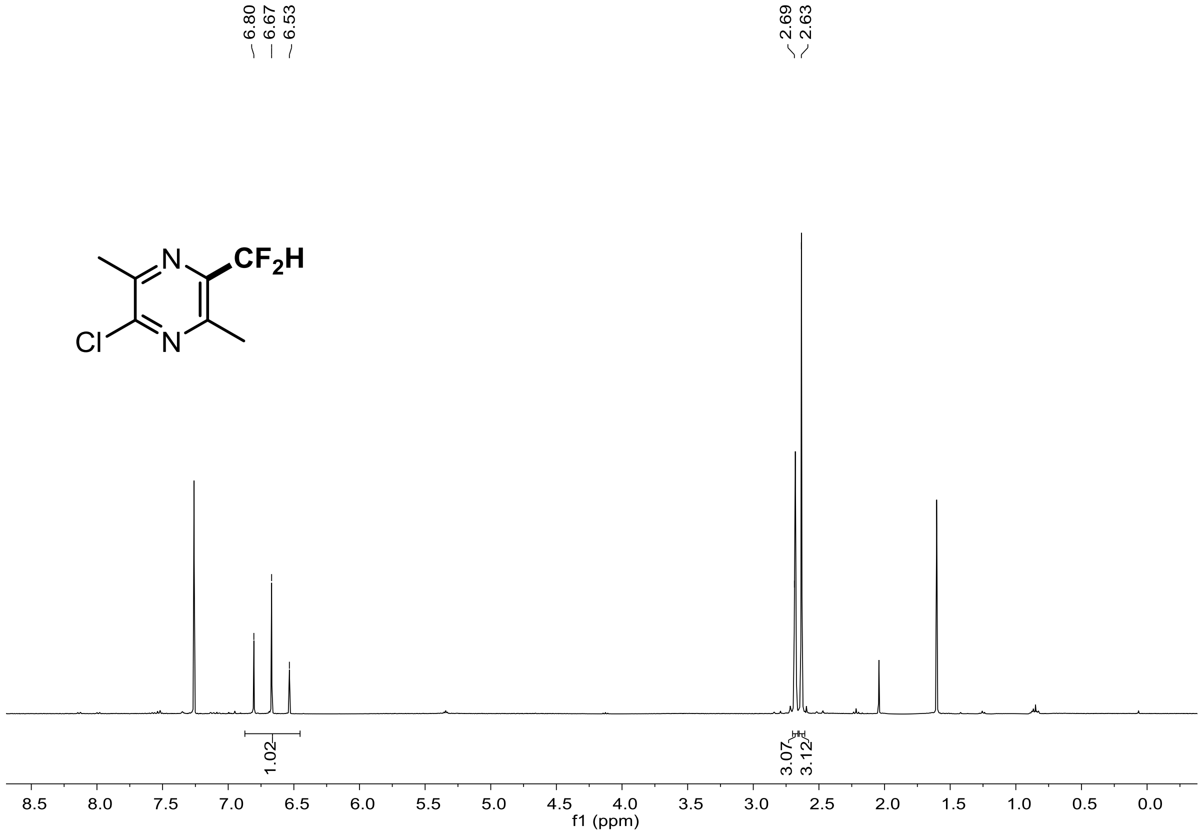

Supplementary Figure 57. ^{1}H NMR Spectrum of **5b**

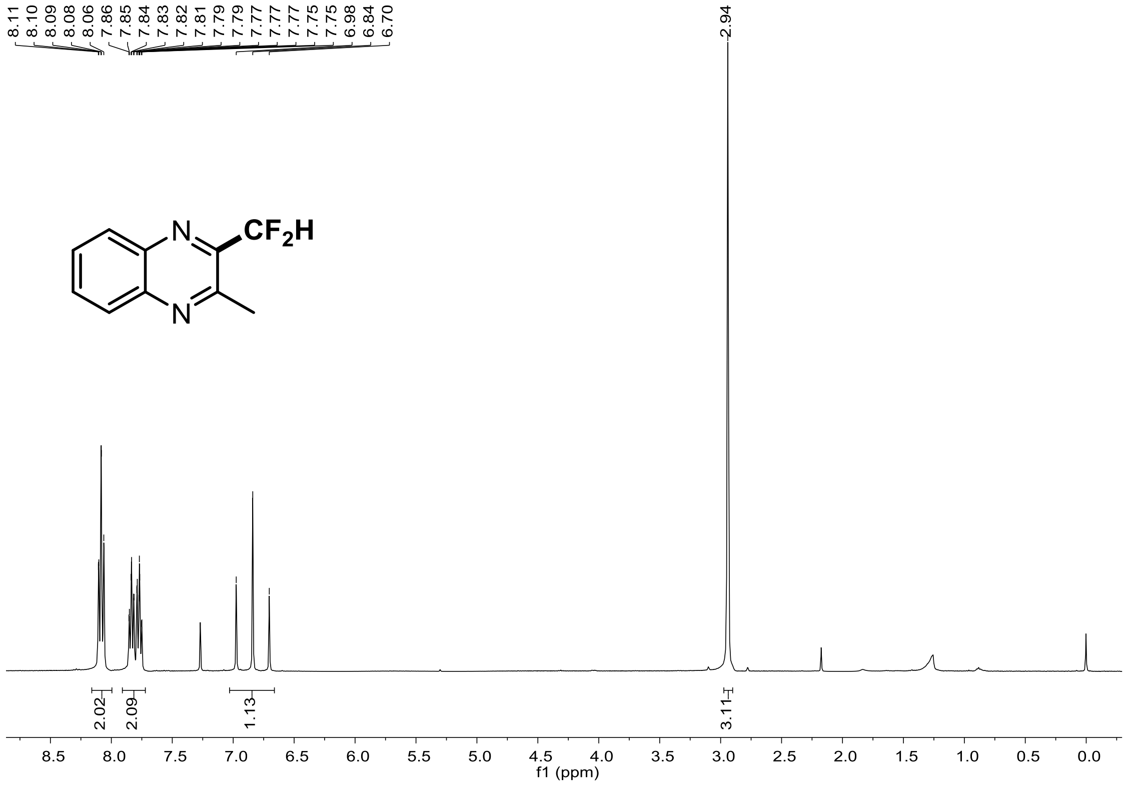

Supplementary Figure 58. ^{1}H NMR Spectrum of **5c**

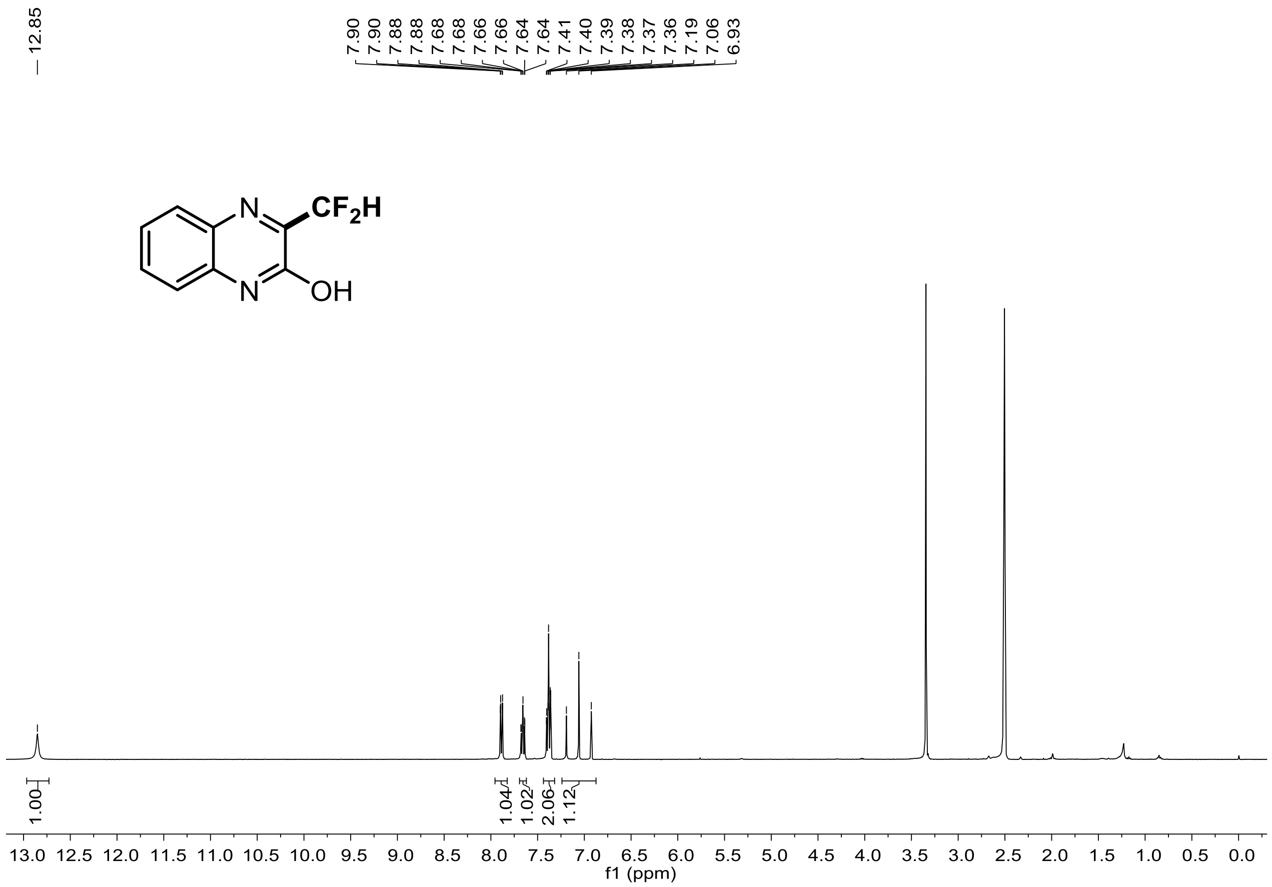

Supplementary Figure 59. ^{1}H NMR Spectrum of **5d**

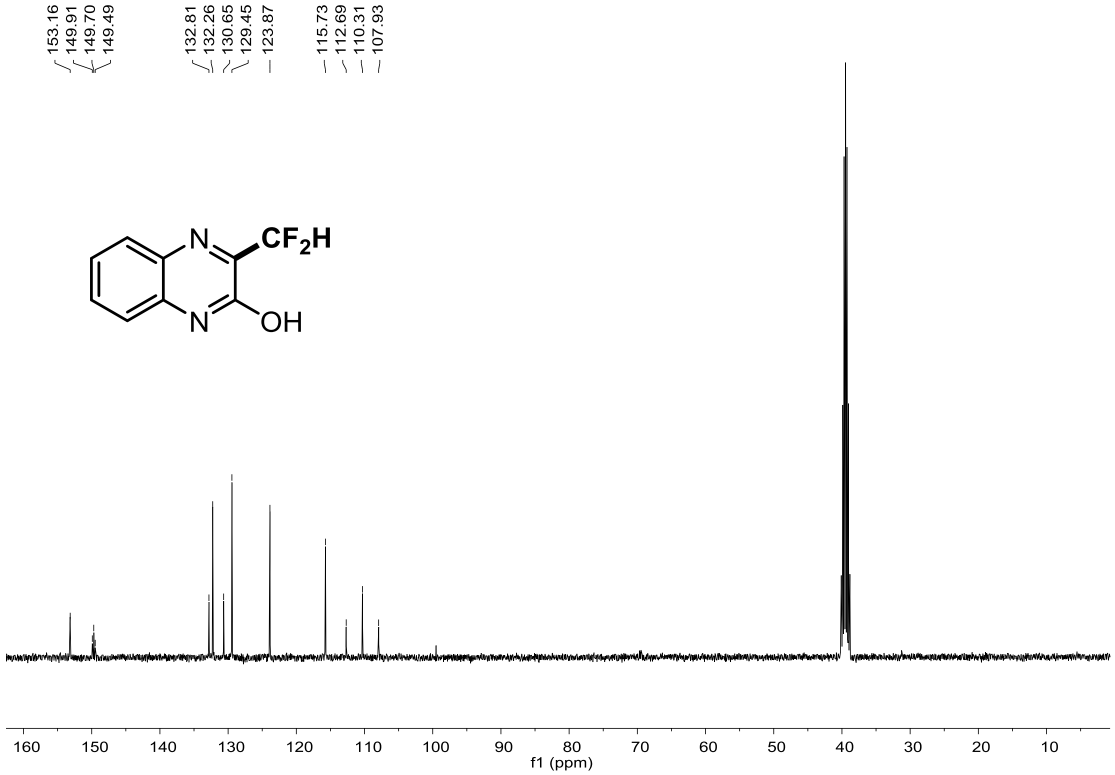

Supplementary Figure 60. ^{13}C NMR Spectrum of **5d**

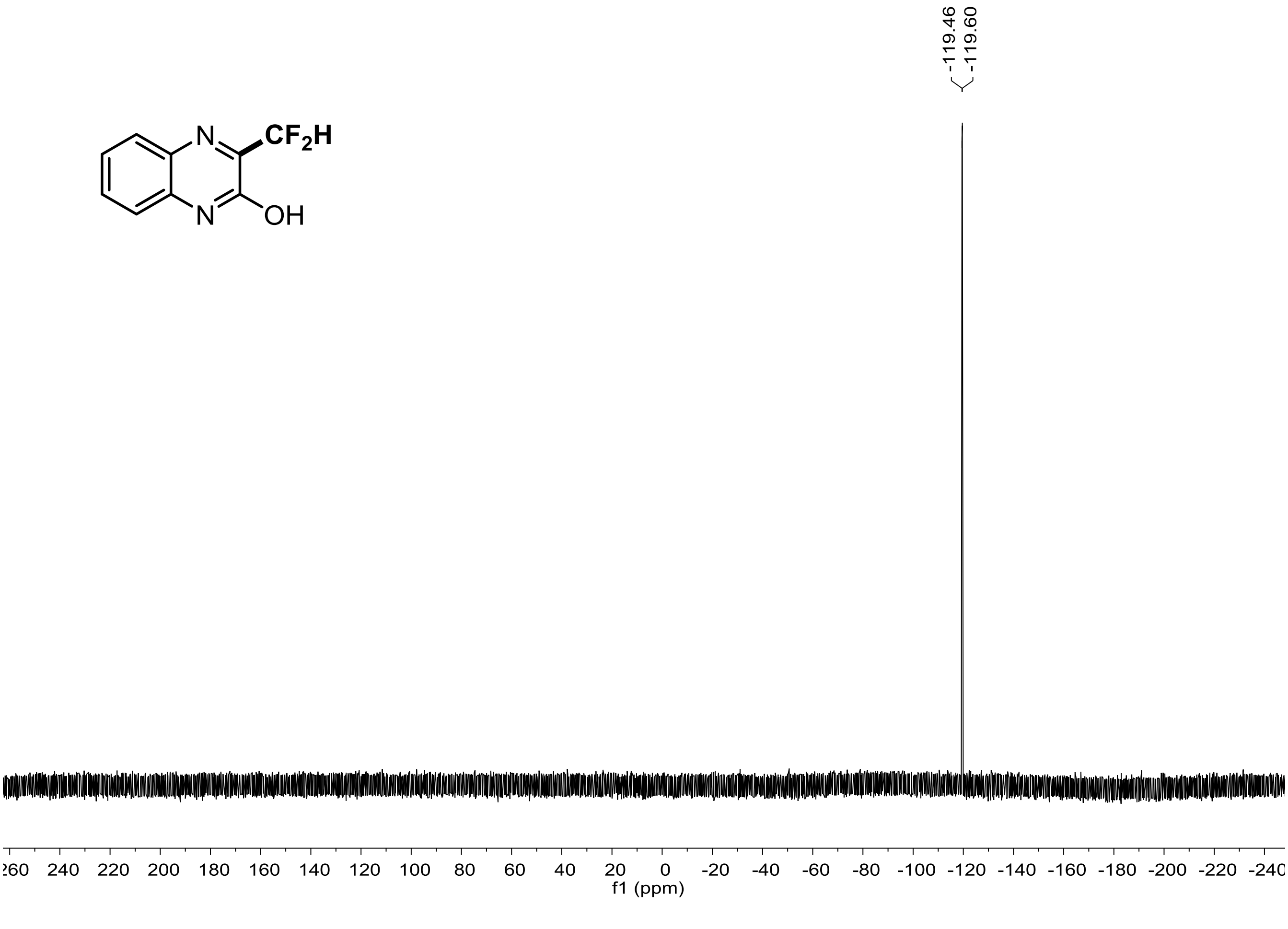

Supplementary Figure 61. ^{19}F NMR Spectrum of **5d**

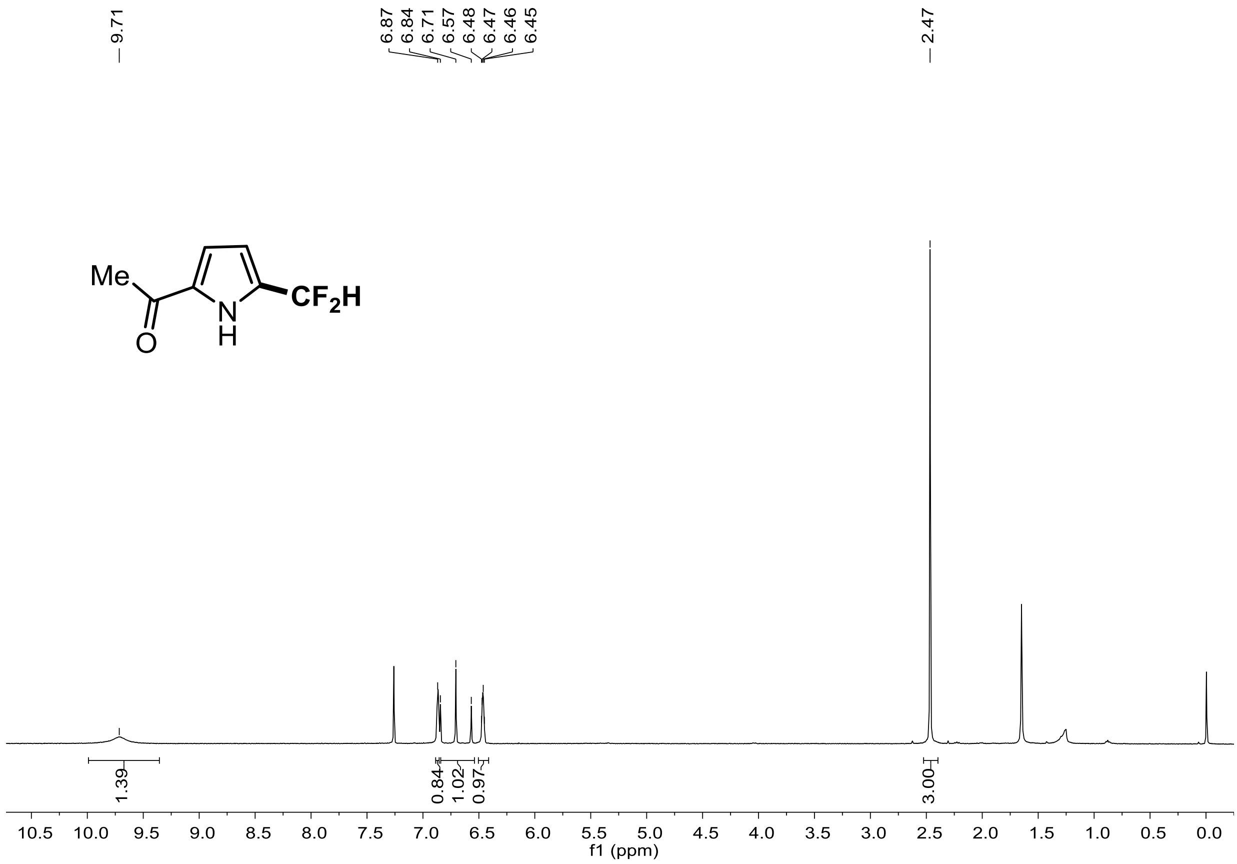

Supplementary Figure 62. ^{1}H NMR Spectrum of **5e**

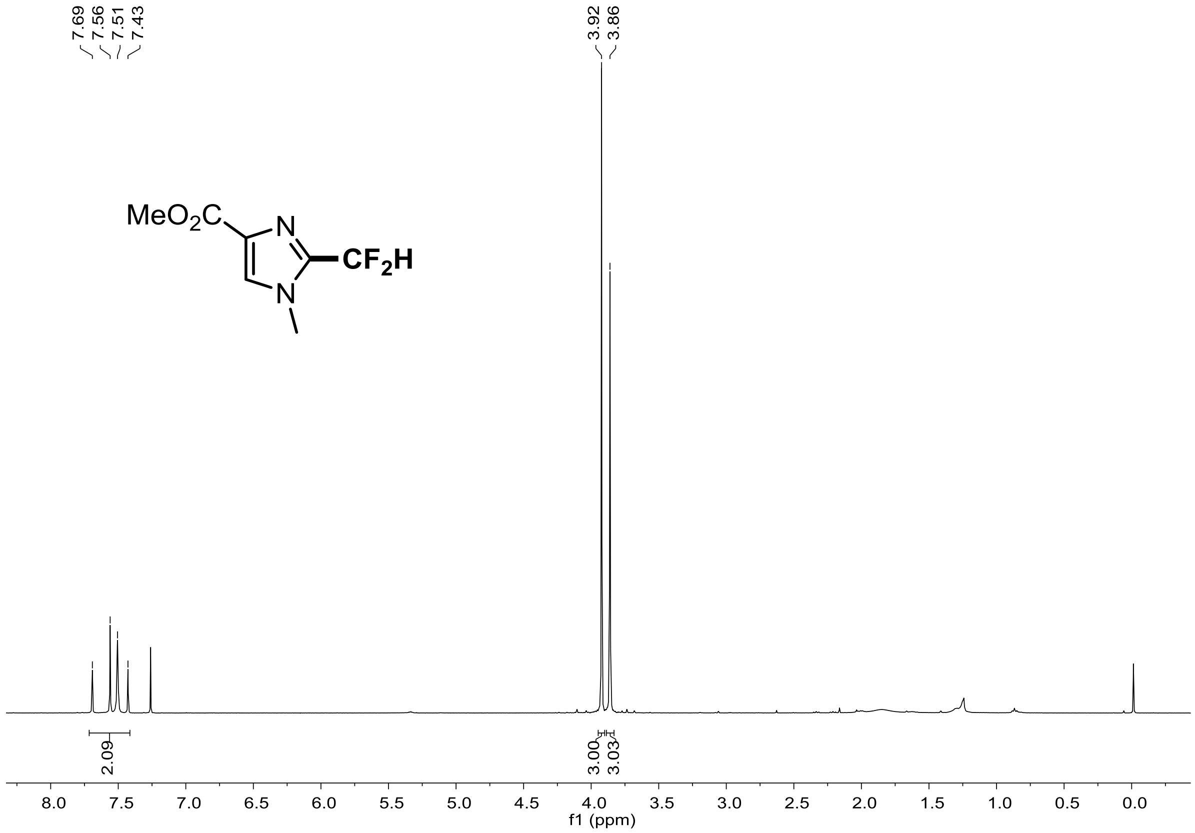

Supplementary Figure 63. ^{1}H NMR Spectrum of **5f**

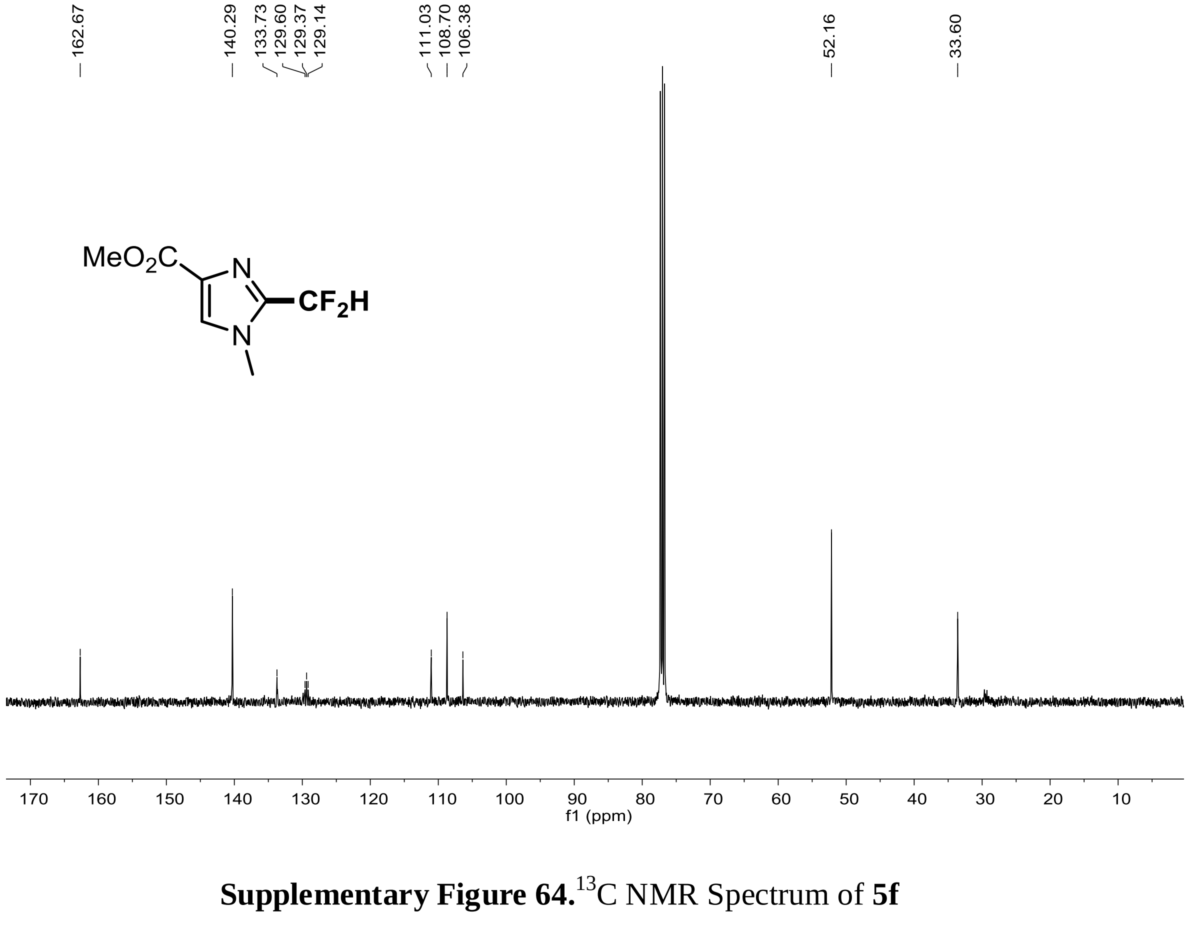

Supplementary Figure 64. ^{13}C NMR Spectrum of **5f**

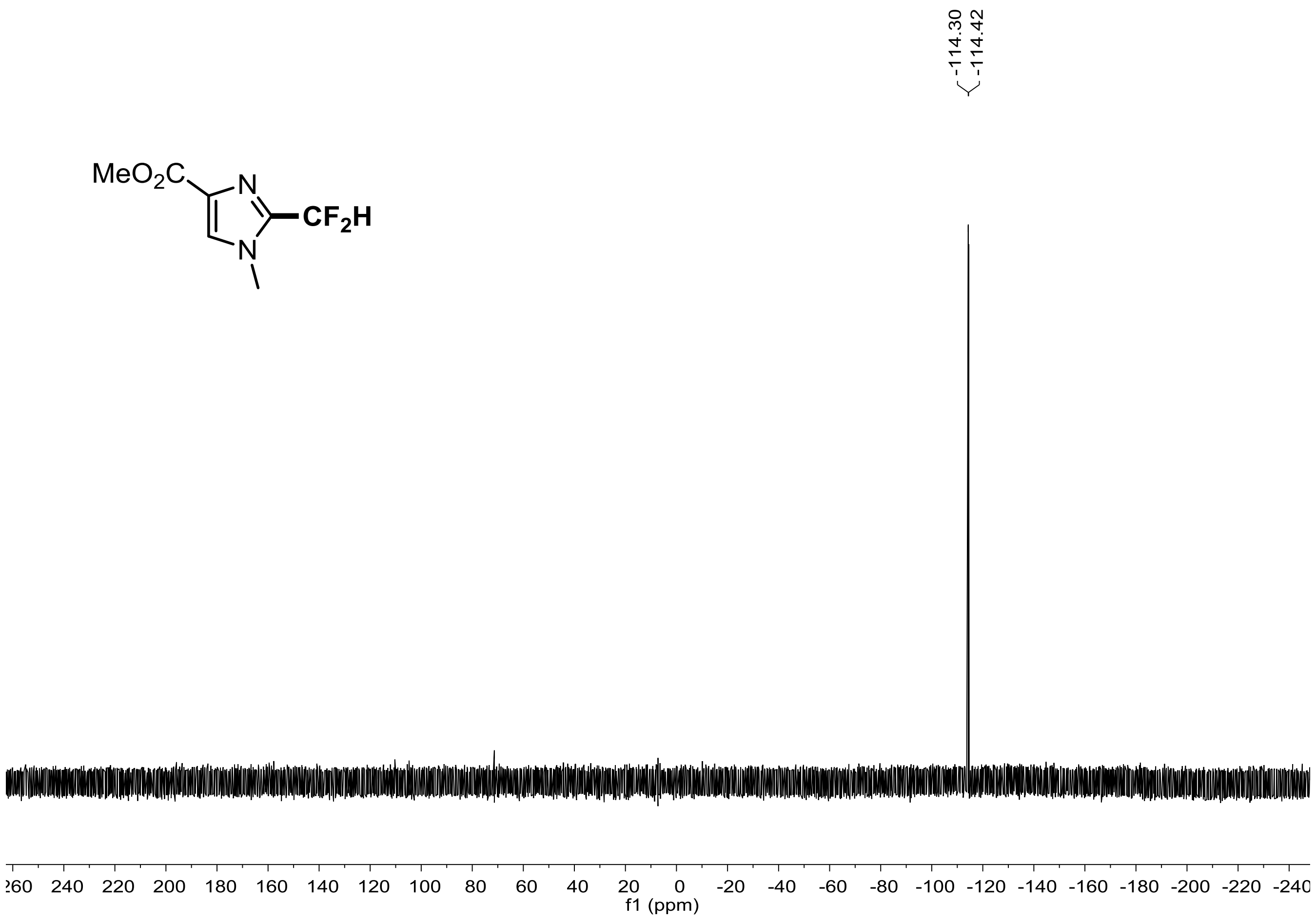

Supplementary Figure 65. ^{19}F NMR Spectrum of **5f**

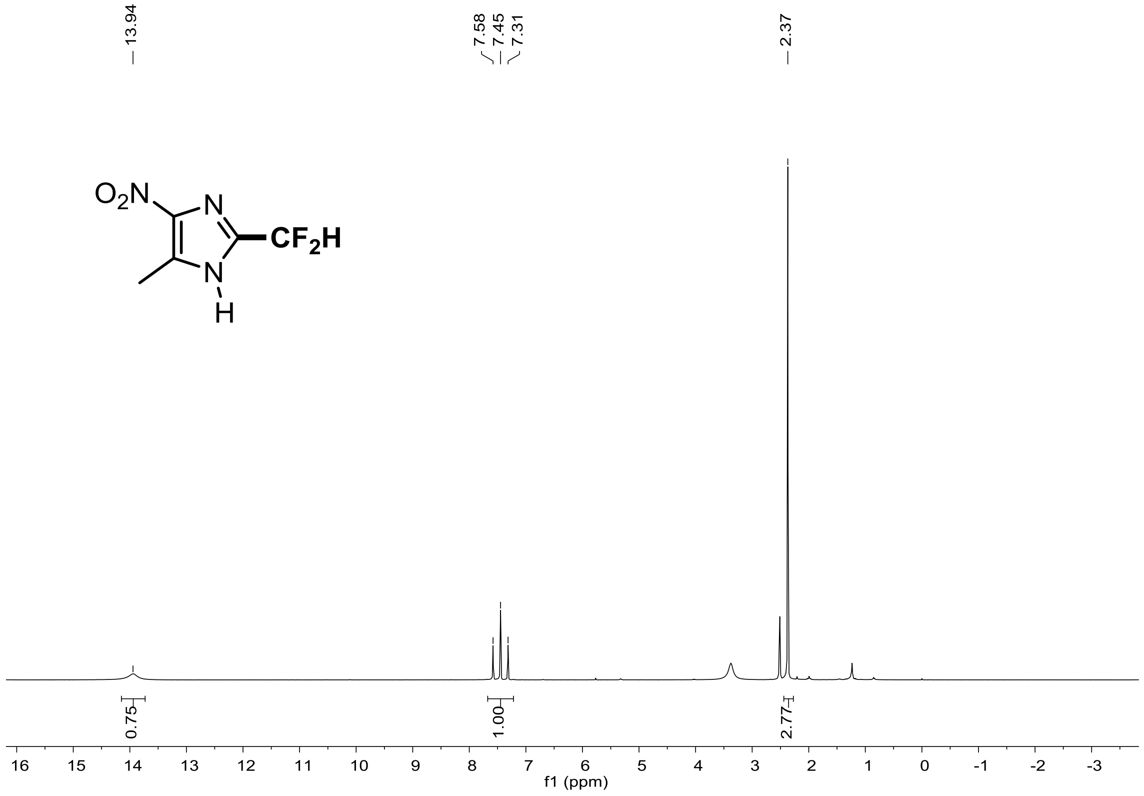

Supplementary Figure 66. ^{1}H NMR Spectrum of **5g**

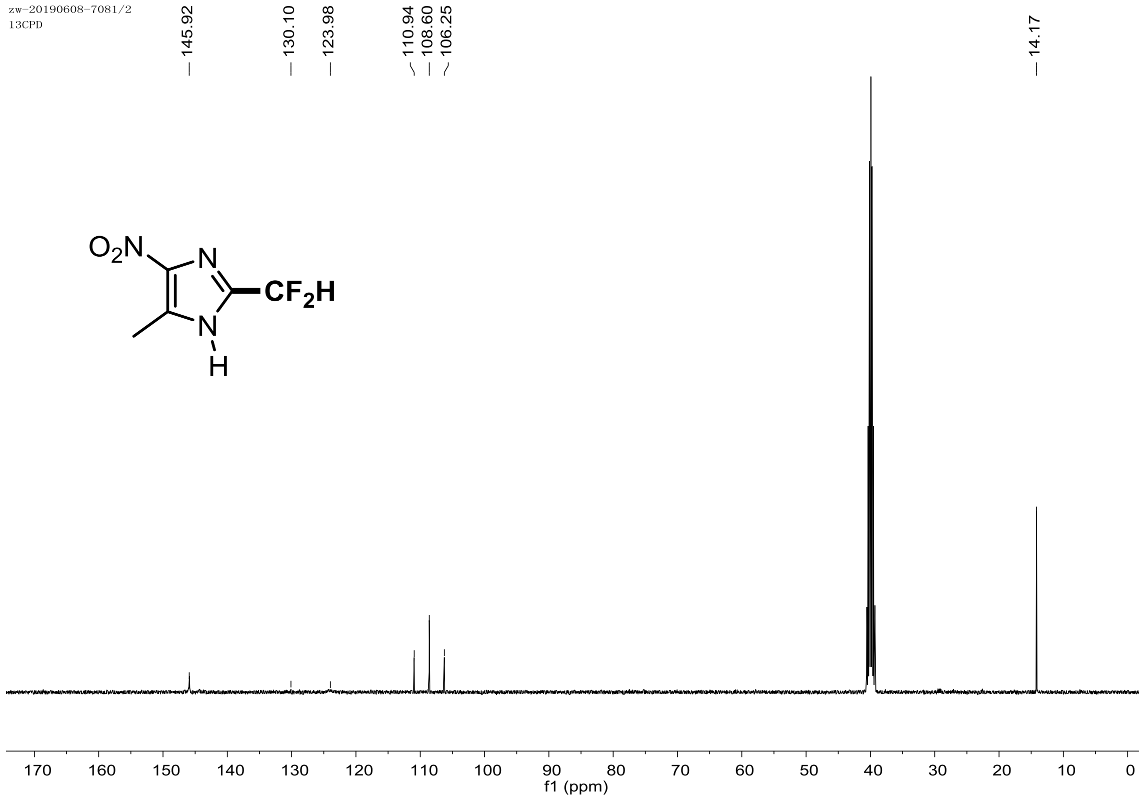

Supplementary Figure 67. ^{13}C NMR Spectrum of **5g**

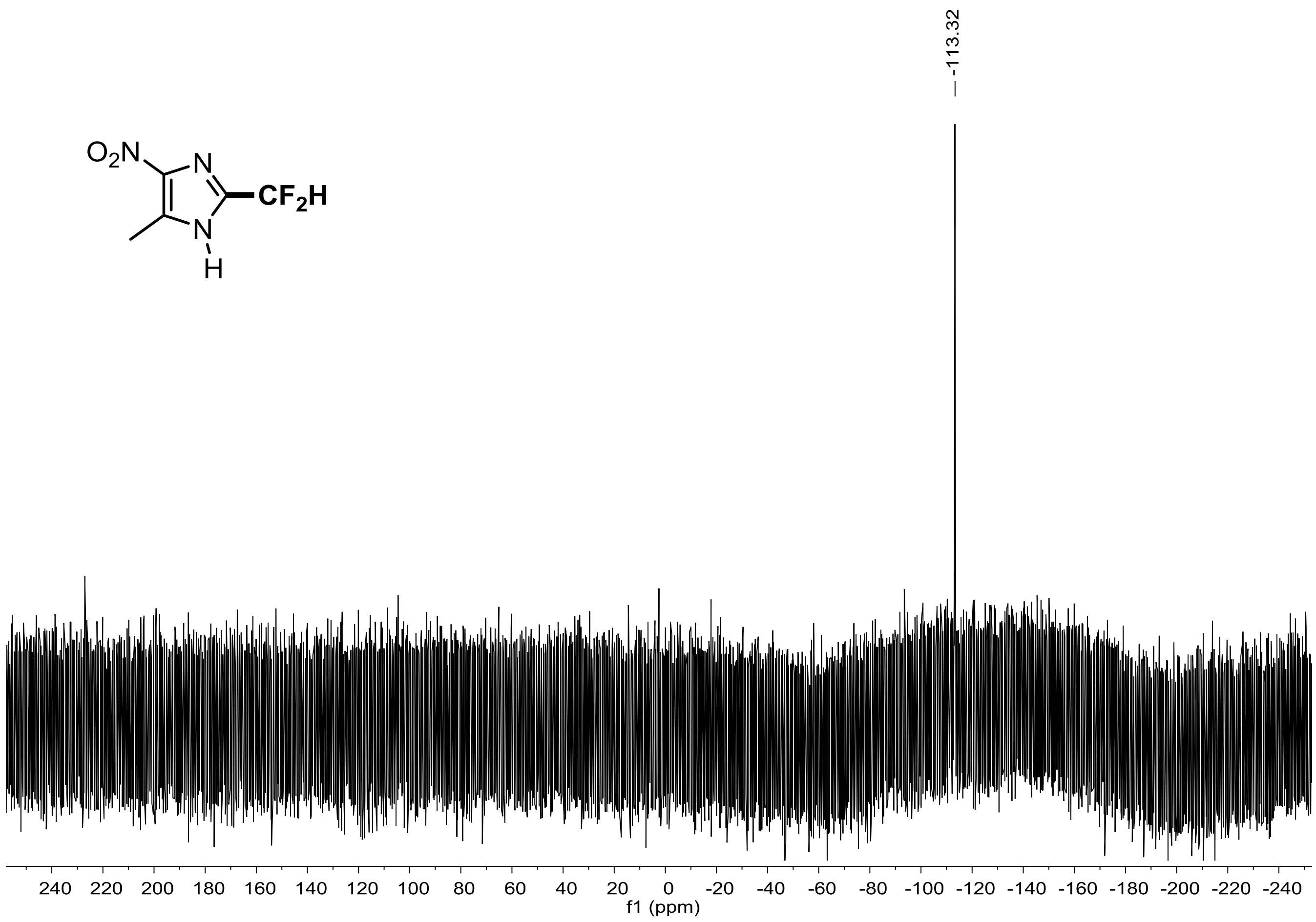

Supplementary Figure 68. ^{19}F NMR Spectrum of **5g**

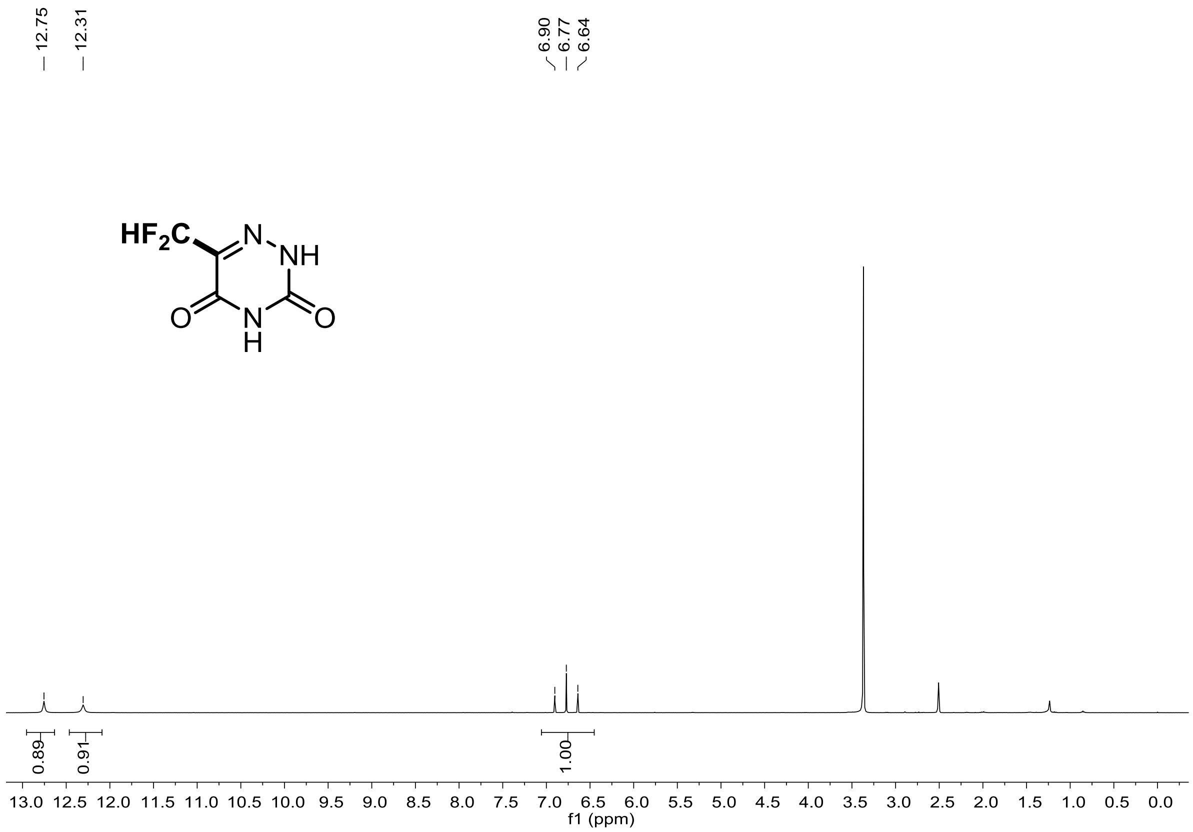

Supplementary Figure 69. ^{1}H NMR Spectrum of **5h**

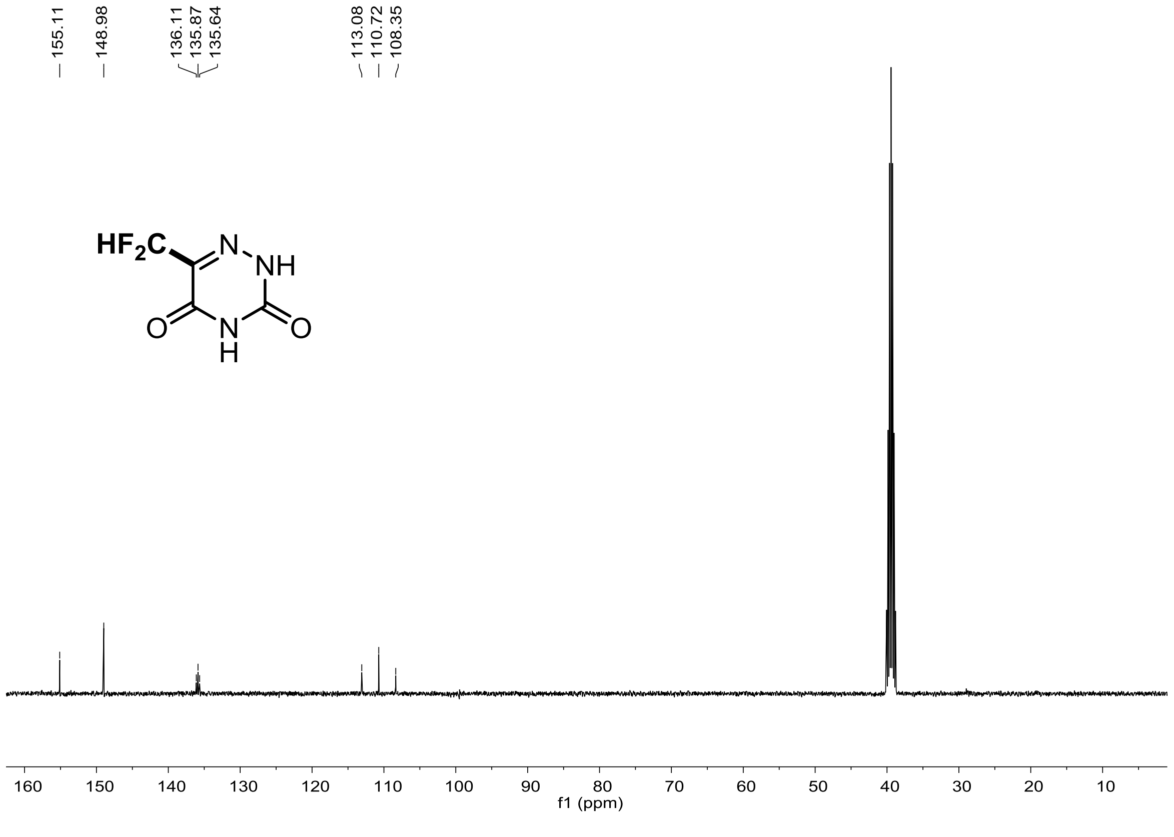

Supplementary Figure 70. ^{13}C NMR Spectrum of **5h**

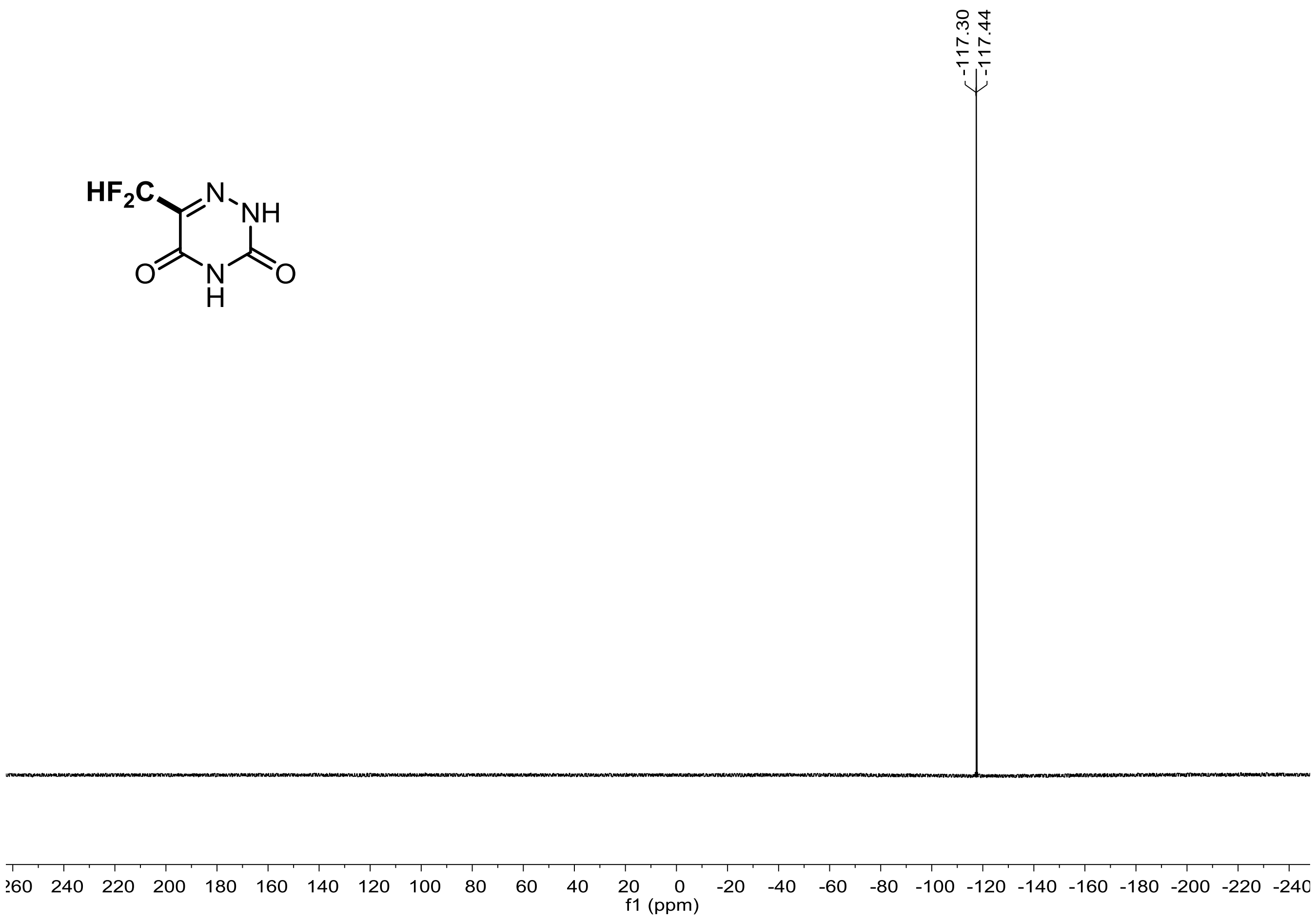

Supplementary Figure 71. ^{19}F NMR Spectrum of **5h**

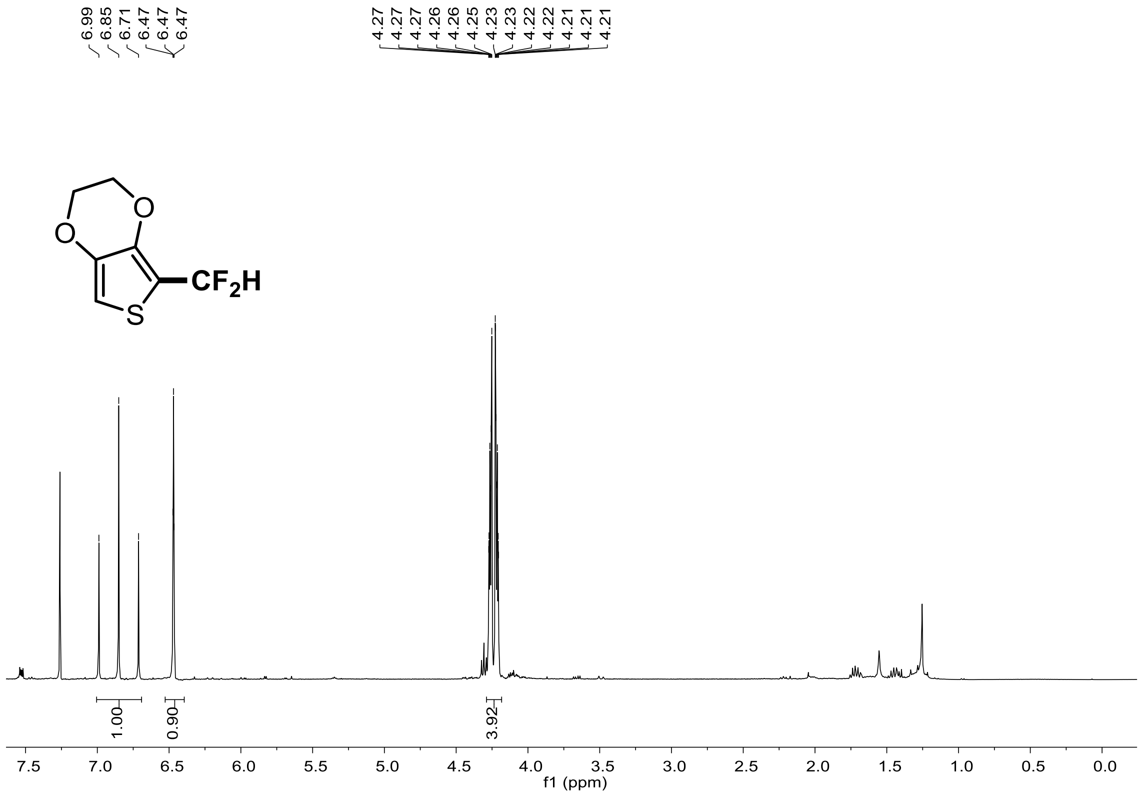

Supplementary Figure 72. ^{1}H NMR Spectrum of **5i**

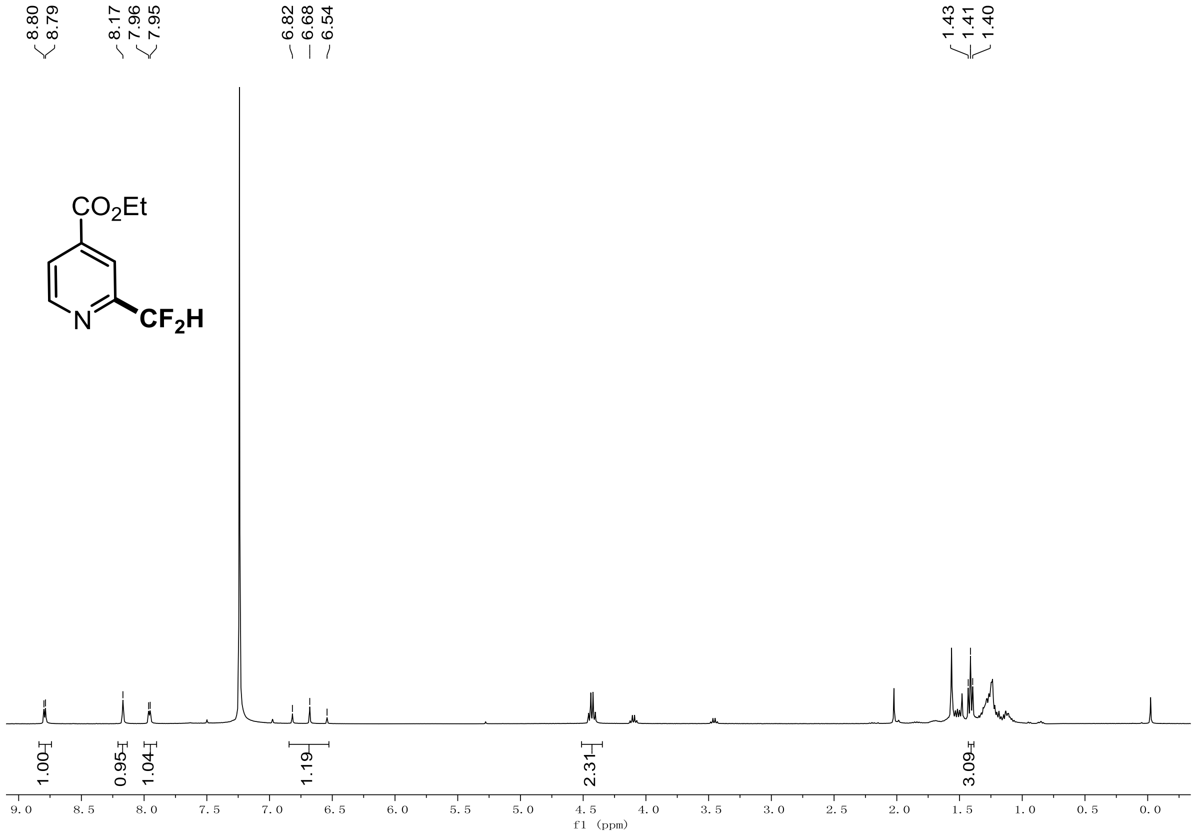

Supplementary Figure 73. ^{1}H NMR Spectrum of **5j**

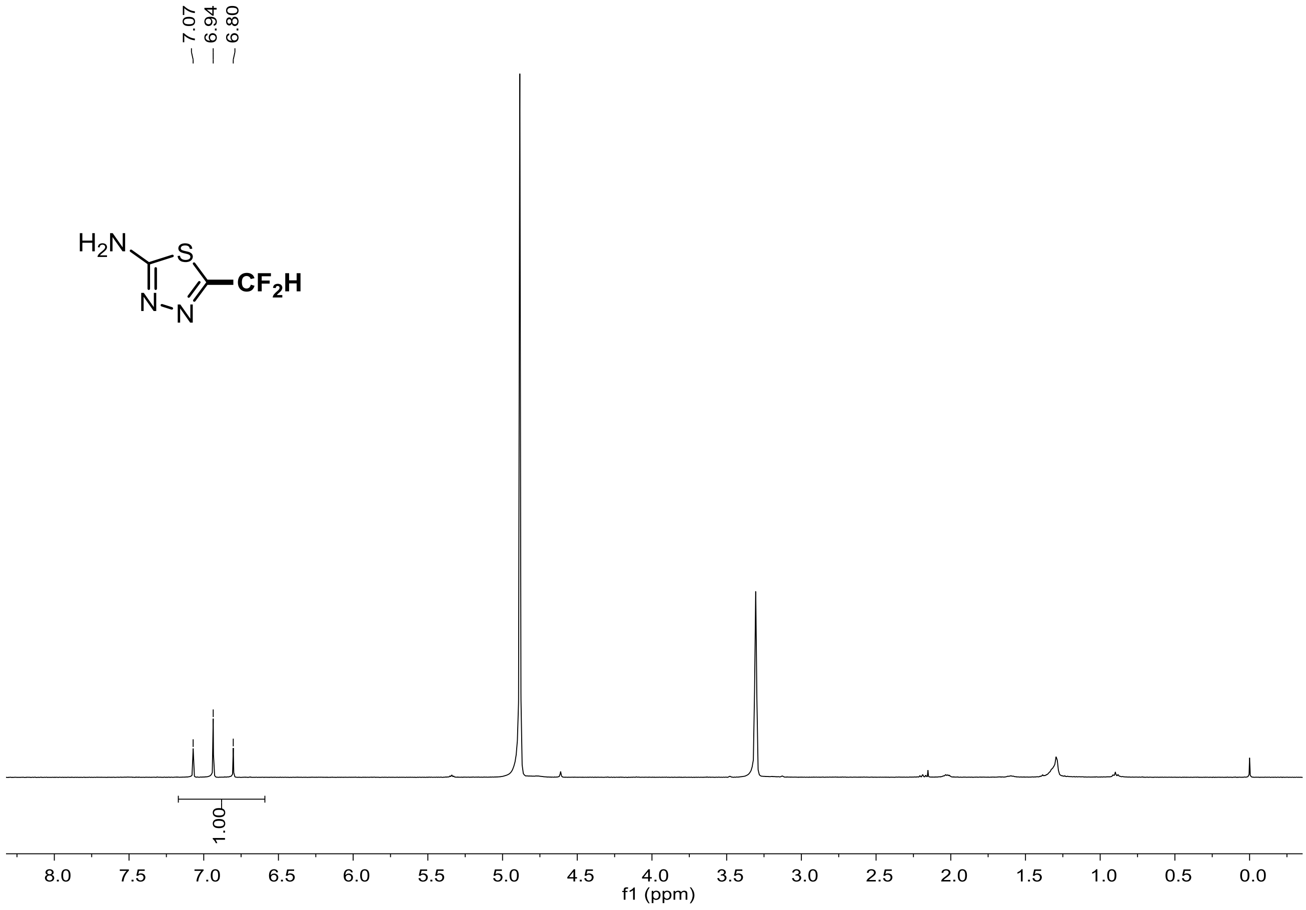

Supplementary Figure 73. ^{1}H NMR Spectrum of **5k**

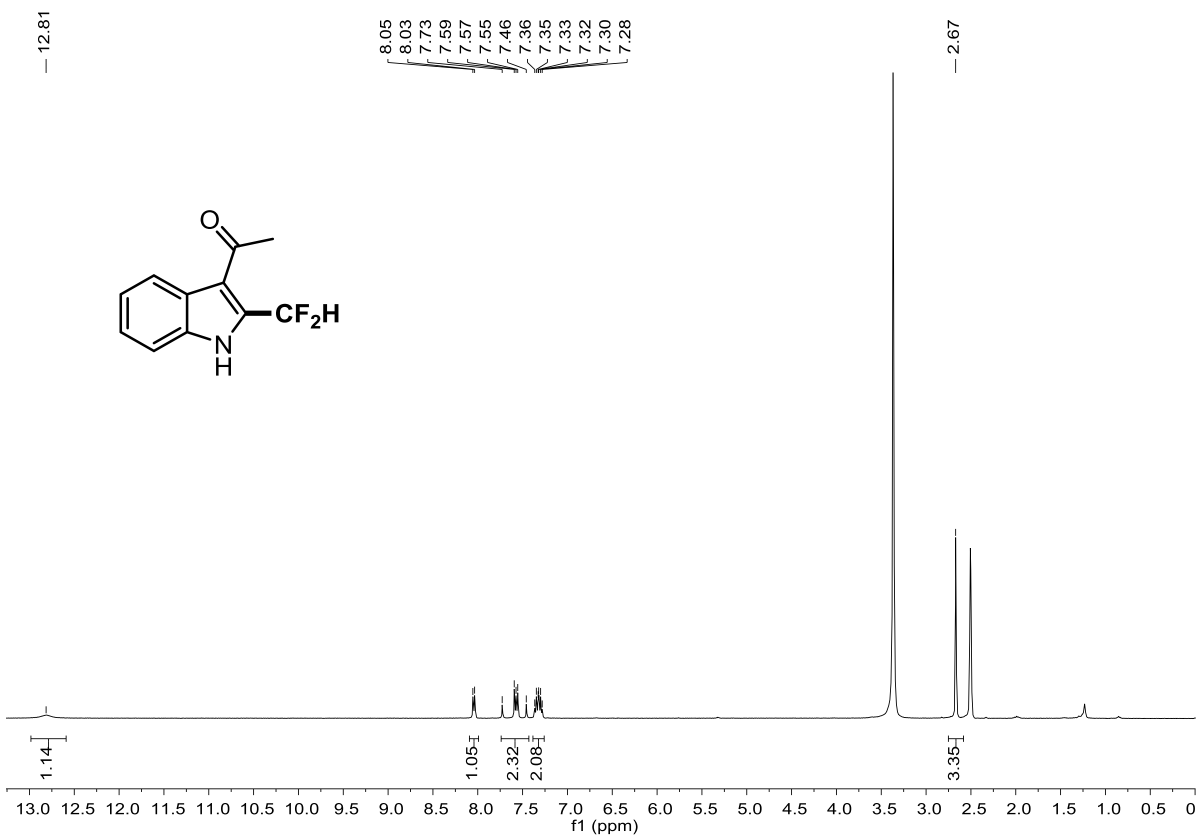

Supplementary Figure 74.^{1}H NMR Spectrum of **5l**

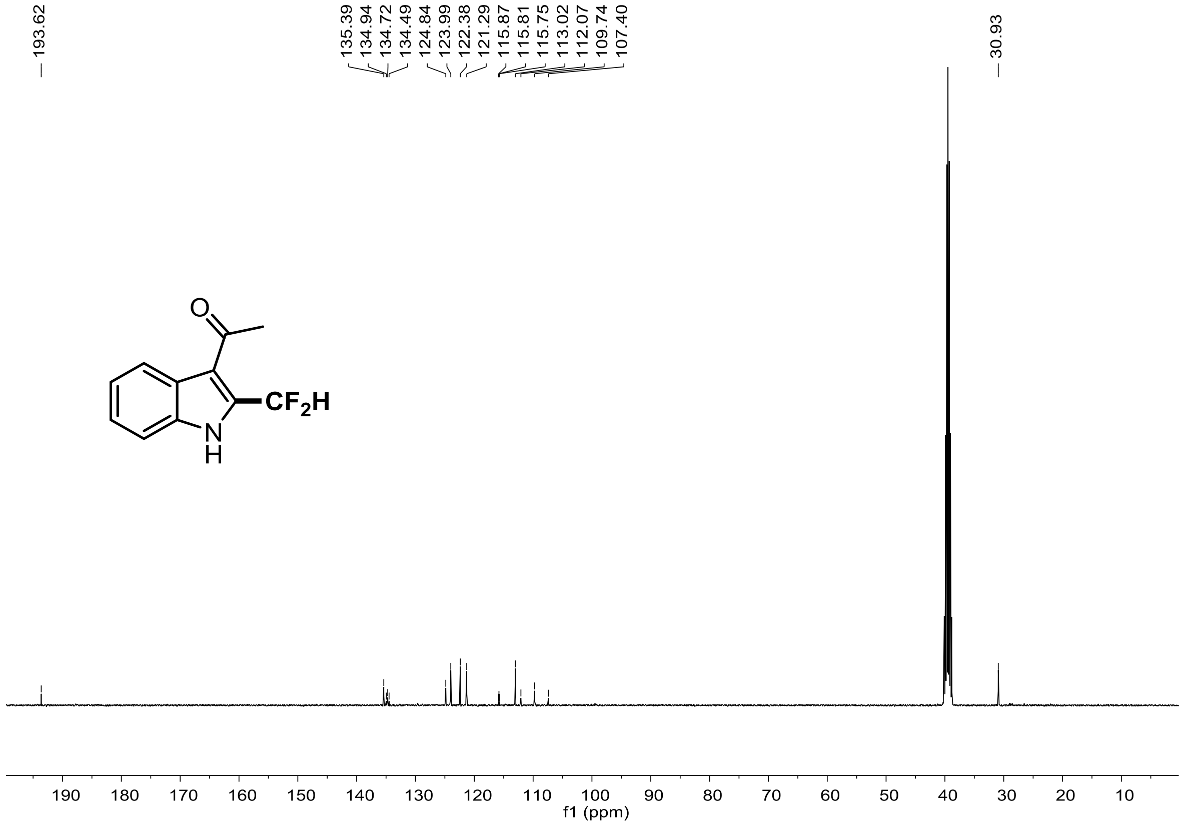

Supplementary Figure 75.^{13}C NMR Spectrum of **5l**

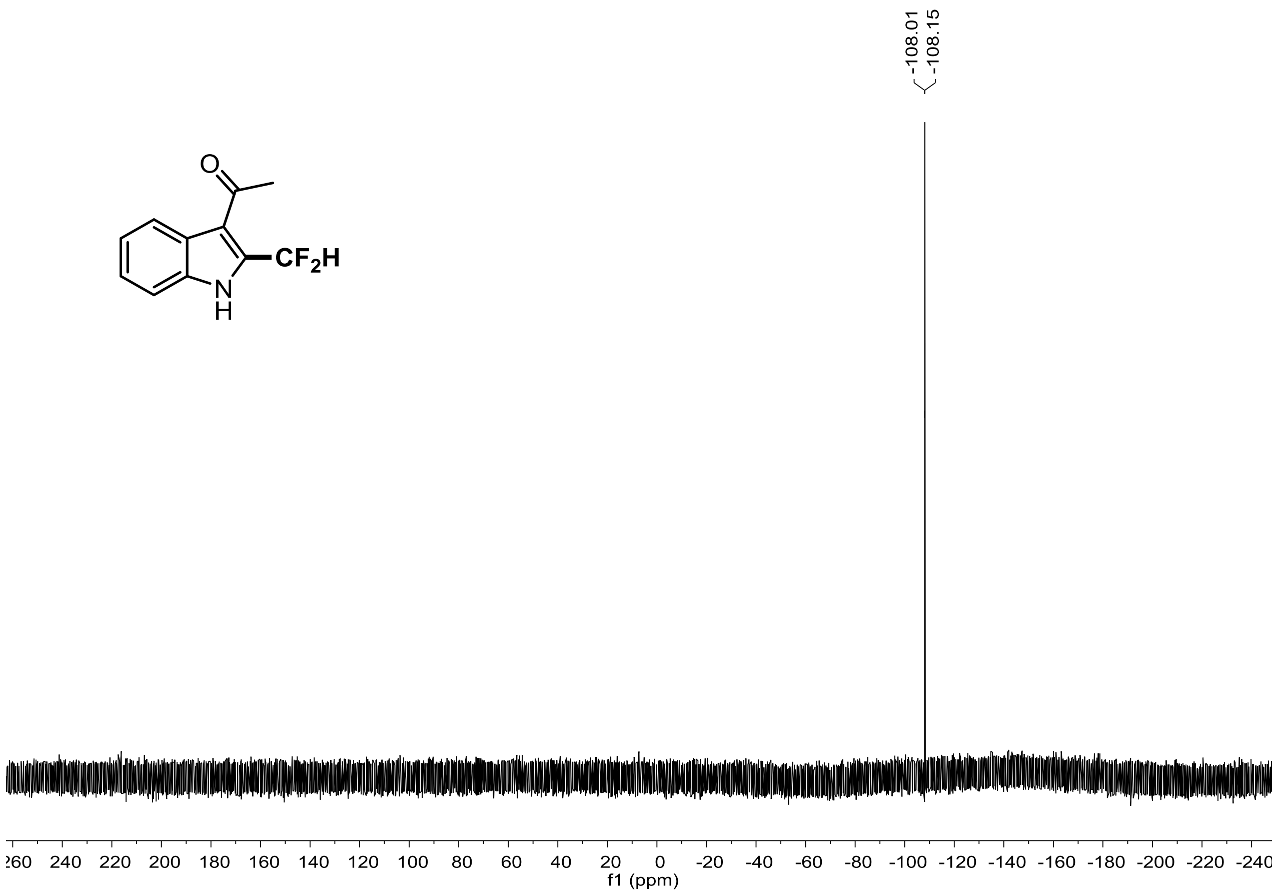

Supplementary Figure 76. ^{19}F NMR Spectrum of **5l**

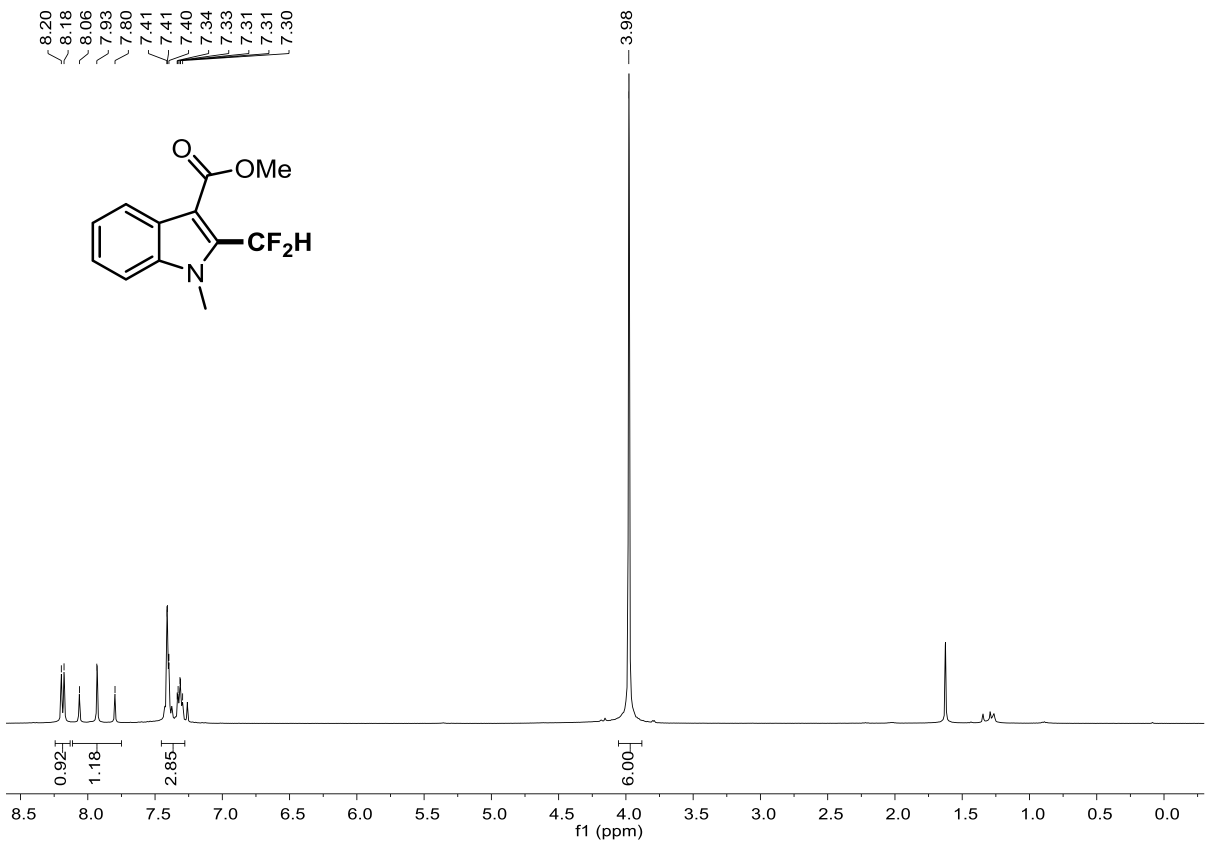

Supplementary Figure 77. ^{1}H NMR Spectrum of **5m**

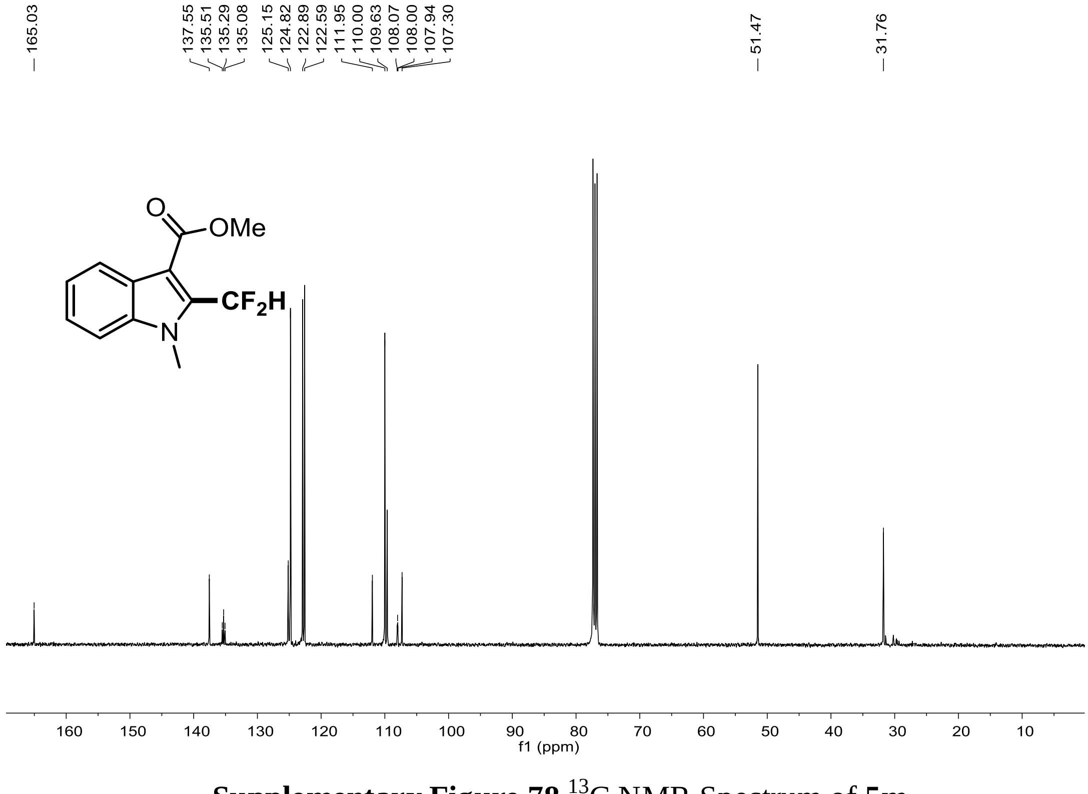

Supplementary Figure 78.^{13}C NMR Spectrum of **5m**

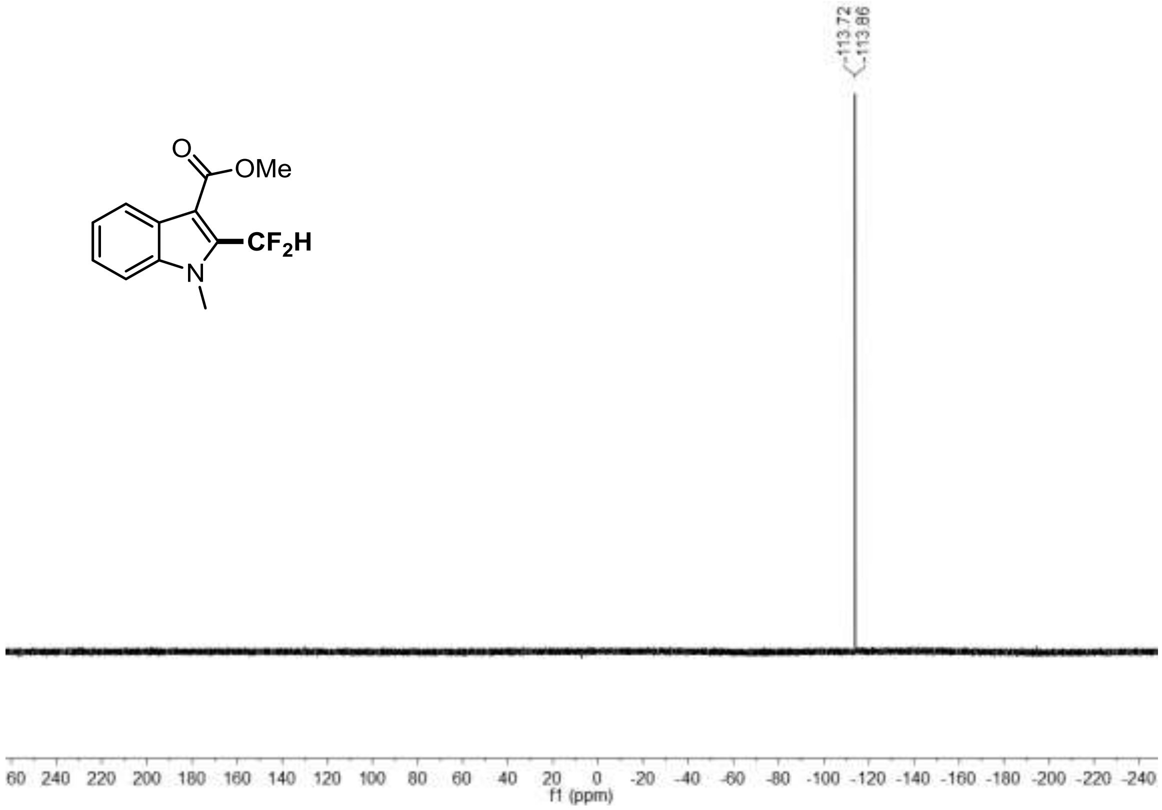

Supplementary Figure 79. ^{19}F NMR Spectrum of **5m**

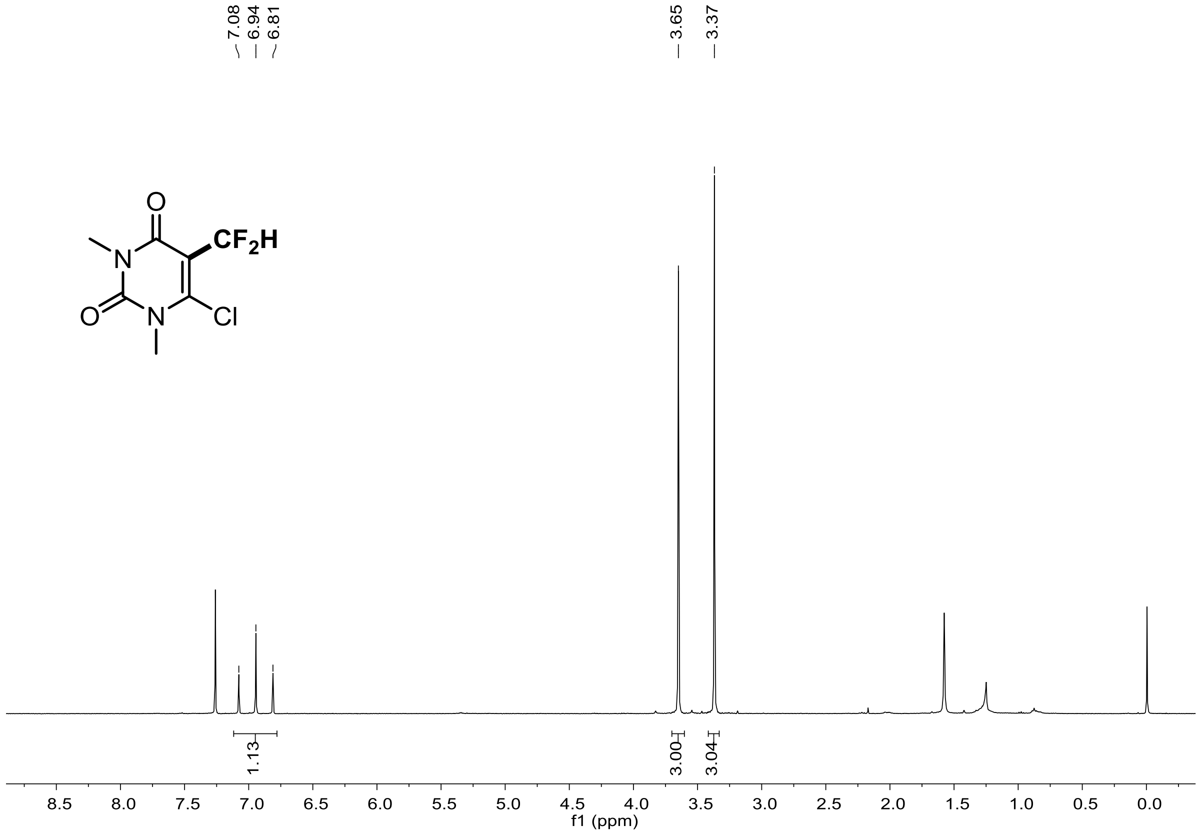

Supplementary Figure 80. ^{1}H NMR Spectrum of **5n**

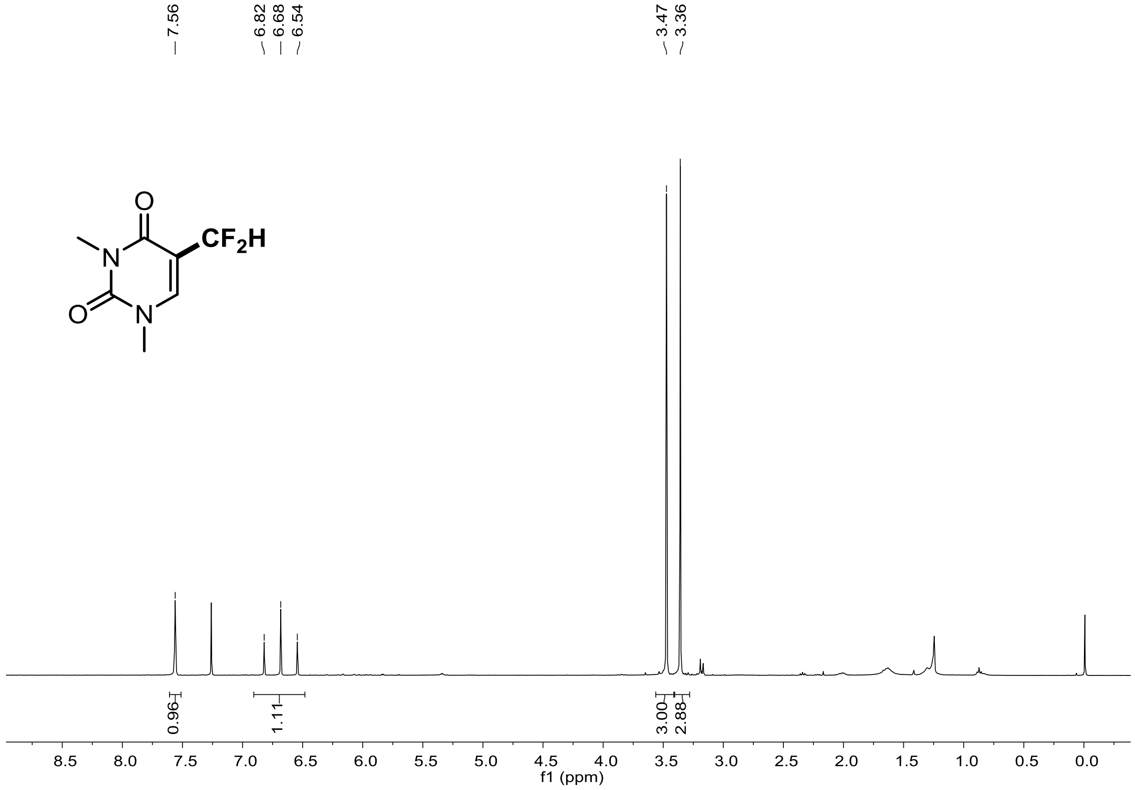

Supplementary Figure 81. ^{1}H NMR Spectrum of **5o**

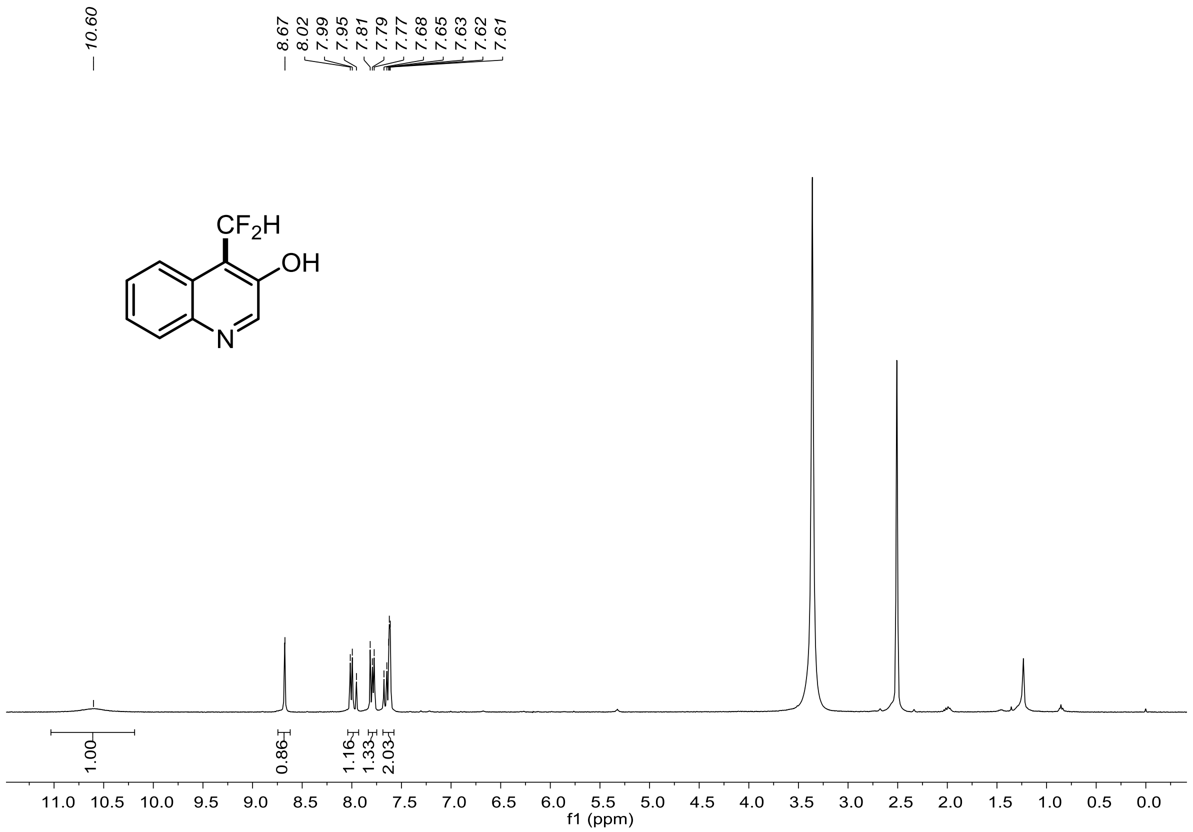

Supplementary Figure 82. ^{1}H NMR Spectrum of **5p**

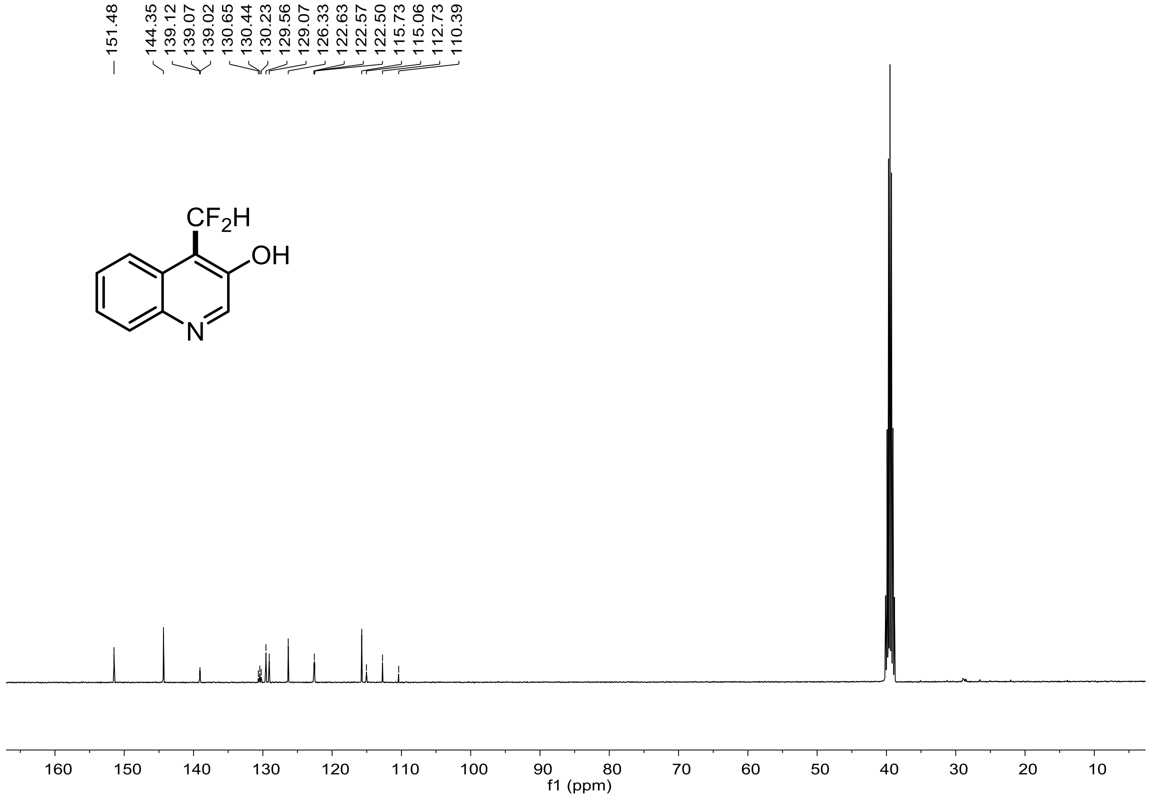

Supplementary Figure 83. ^{13}C NMR Spectrum of **5p**

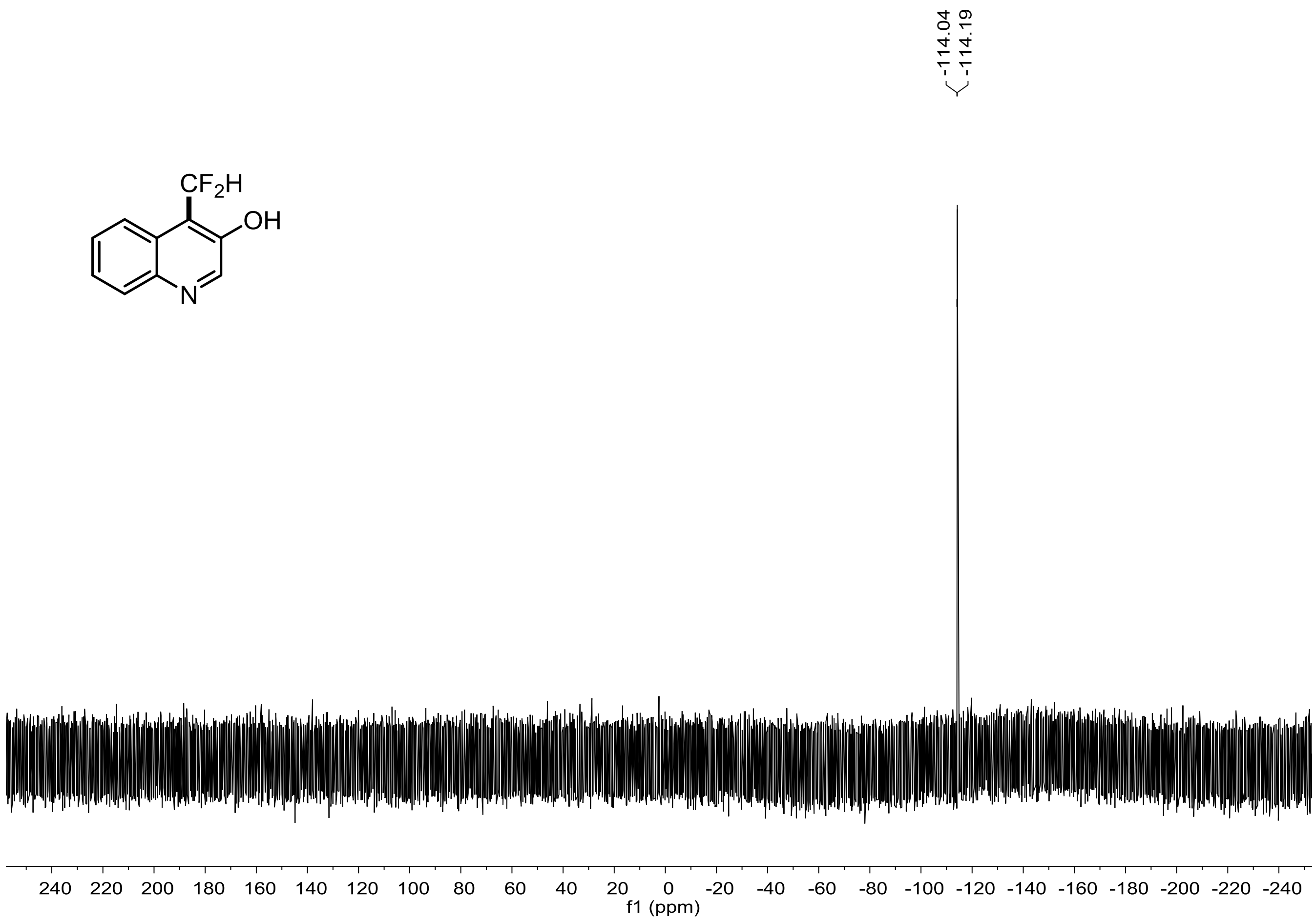

Supplementary Figure 84. ^{19}F NMR Spectrum of **5p**

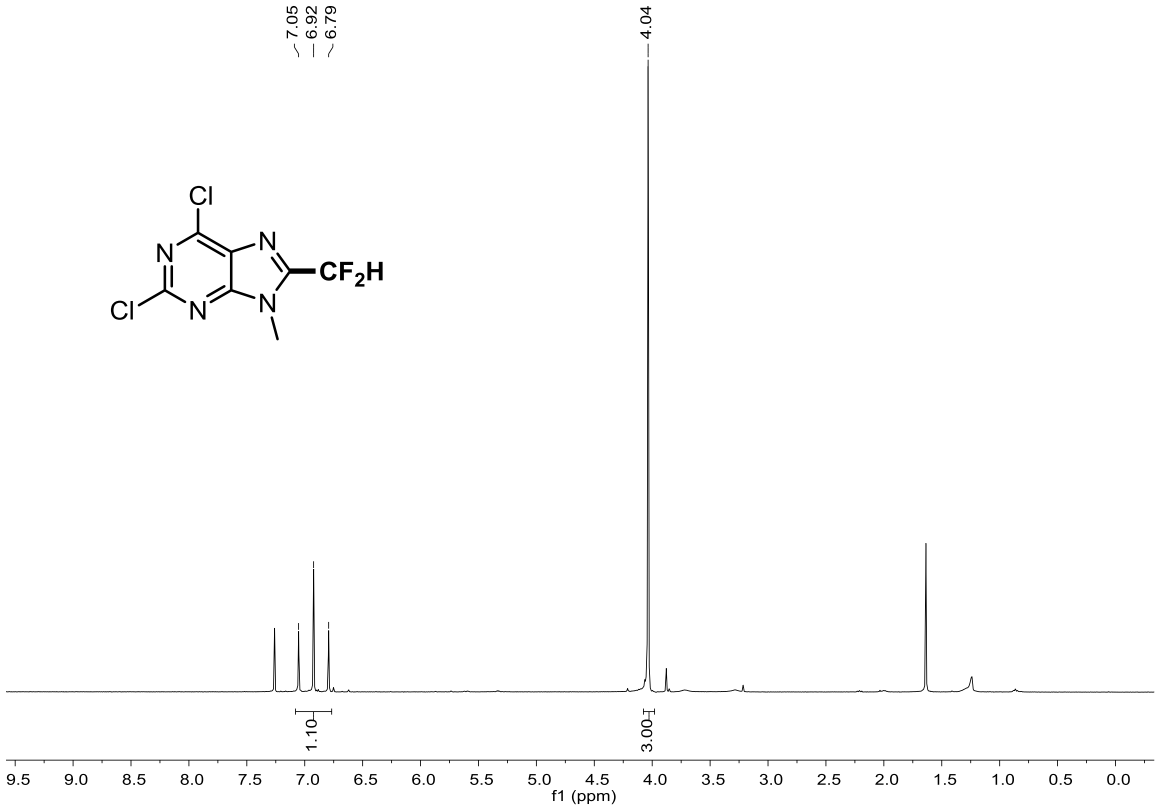

Supplementary Figure 85. ^{1}H NMR Spectrum of **5q**

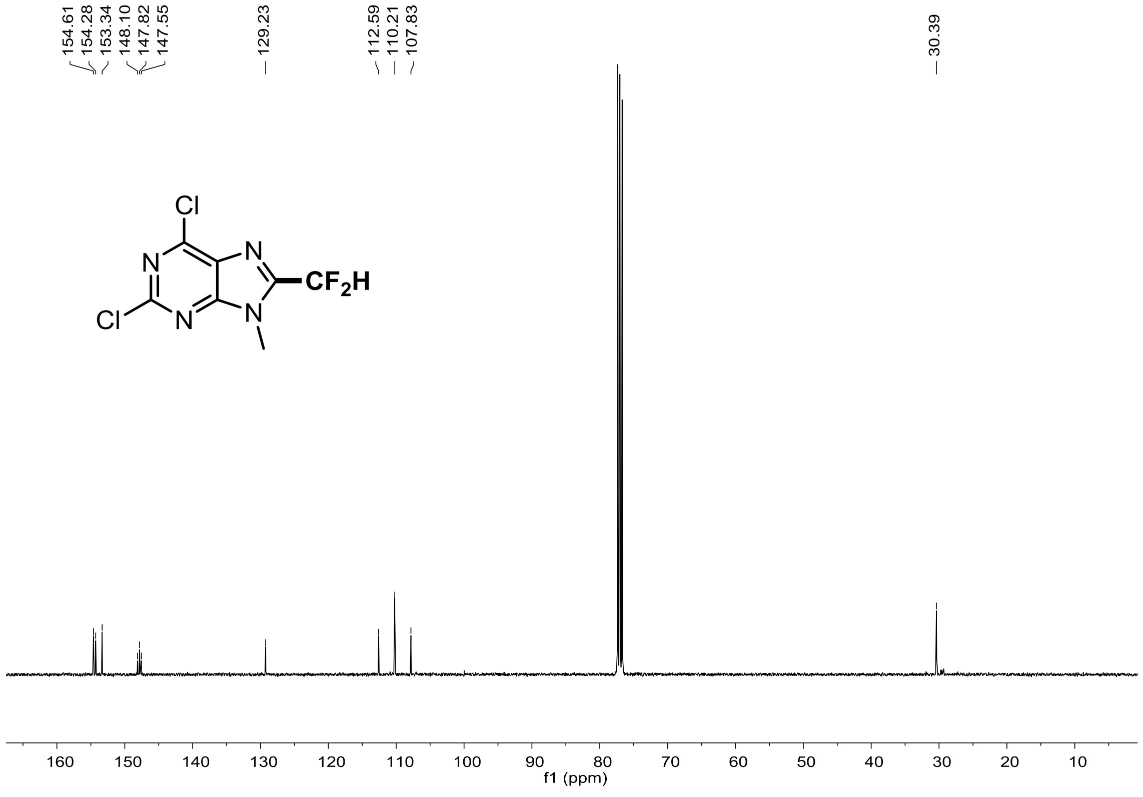

Supplementary Figure 86. ^{13}C NMR Spectrum of **5q**

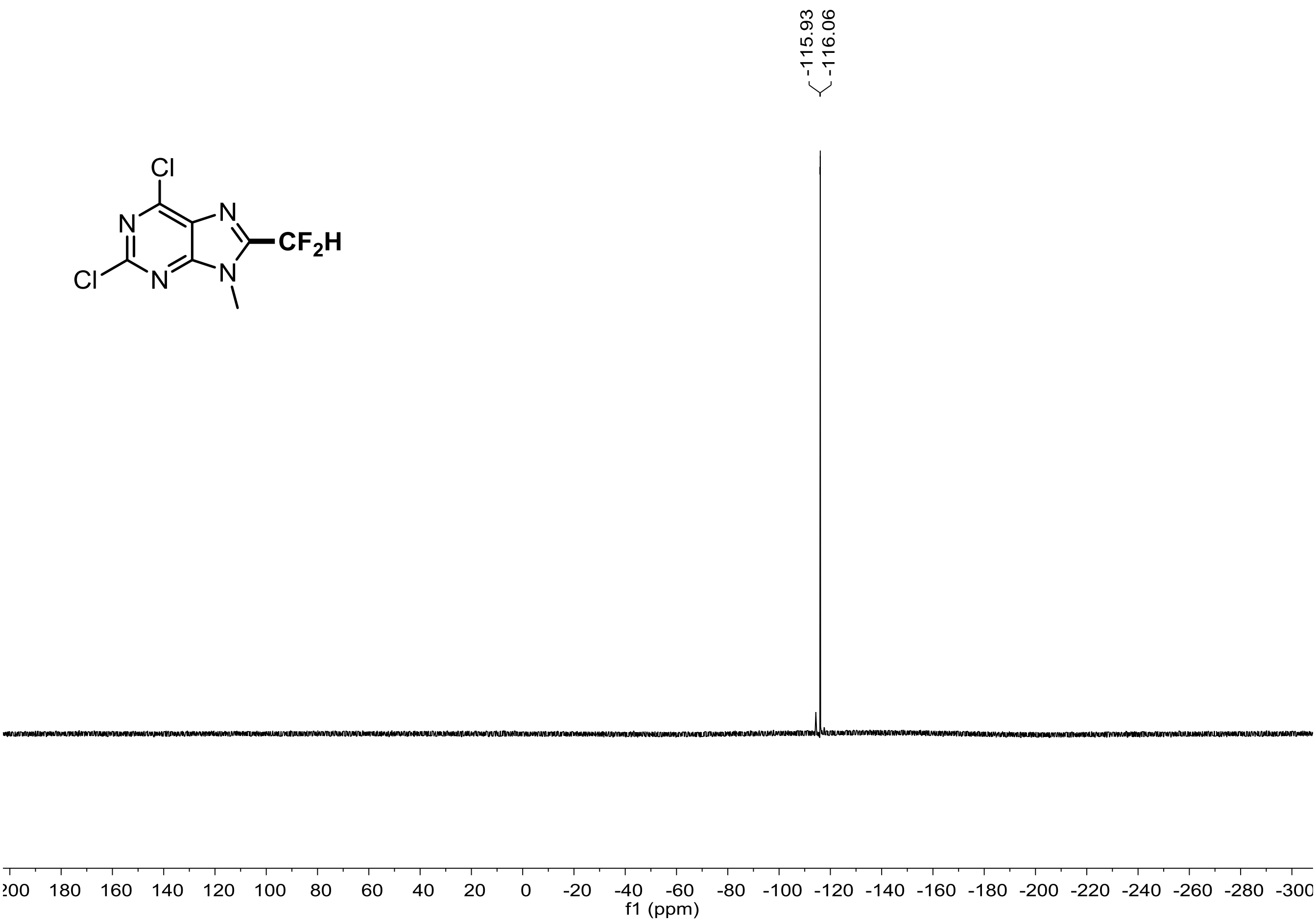

Supplementary Figure 87. ^{19}F NMR Spectrum of **5q**

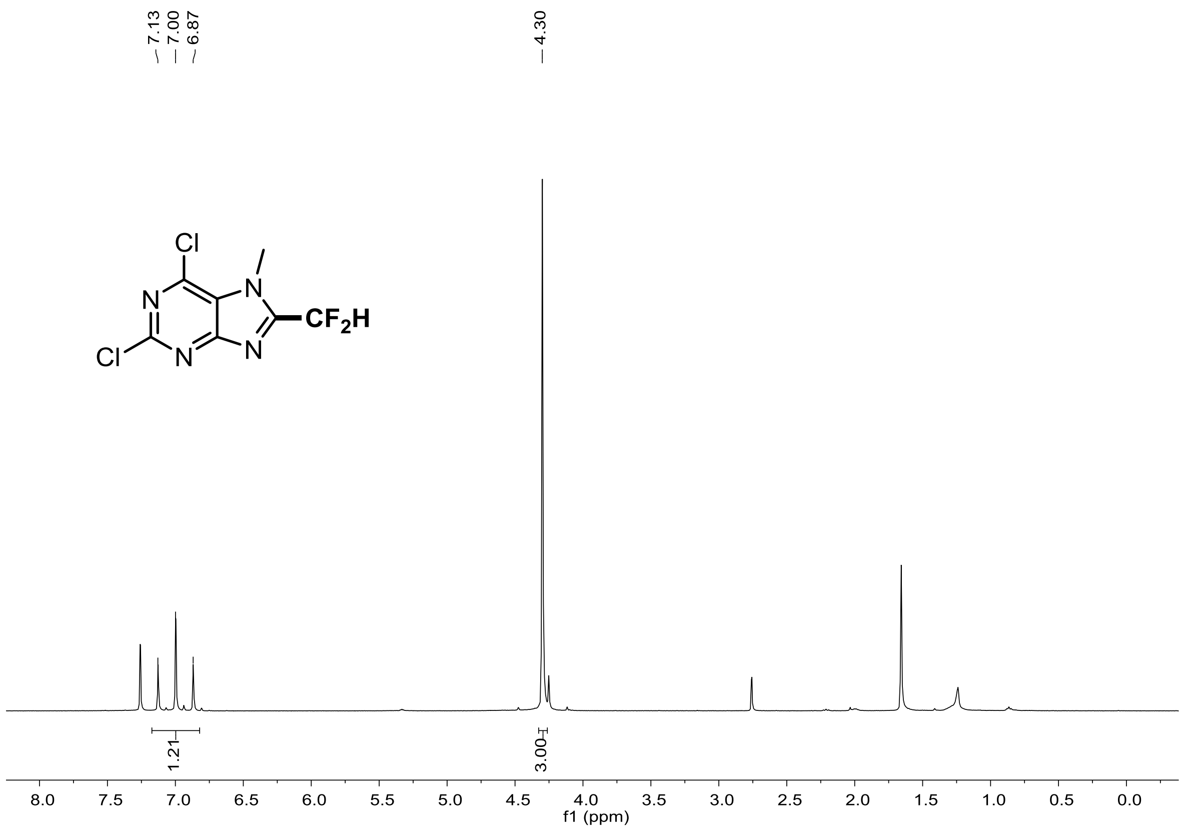

Supplementary Figure 88. ^{1}H NMR Spectrum of **5r**

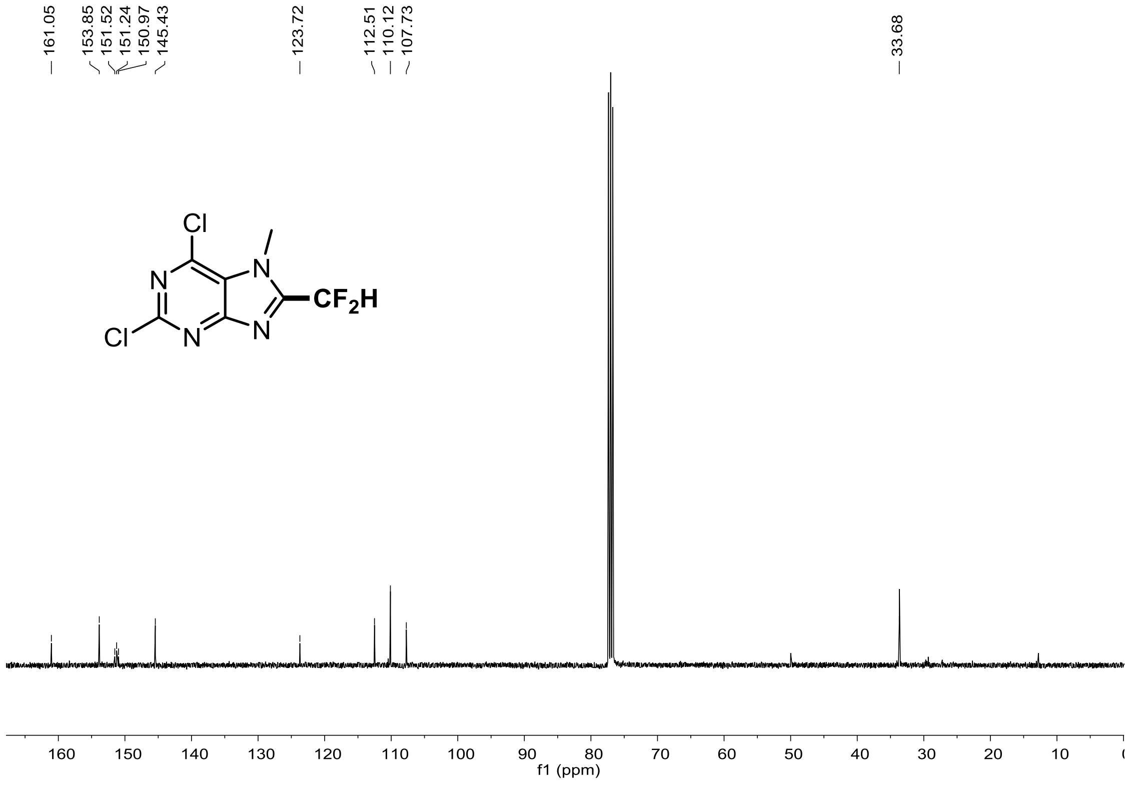

Supplementary Figure 89. ^{13}C NMR Spectrum of **5r**

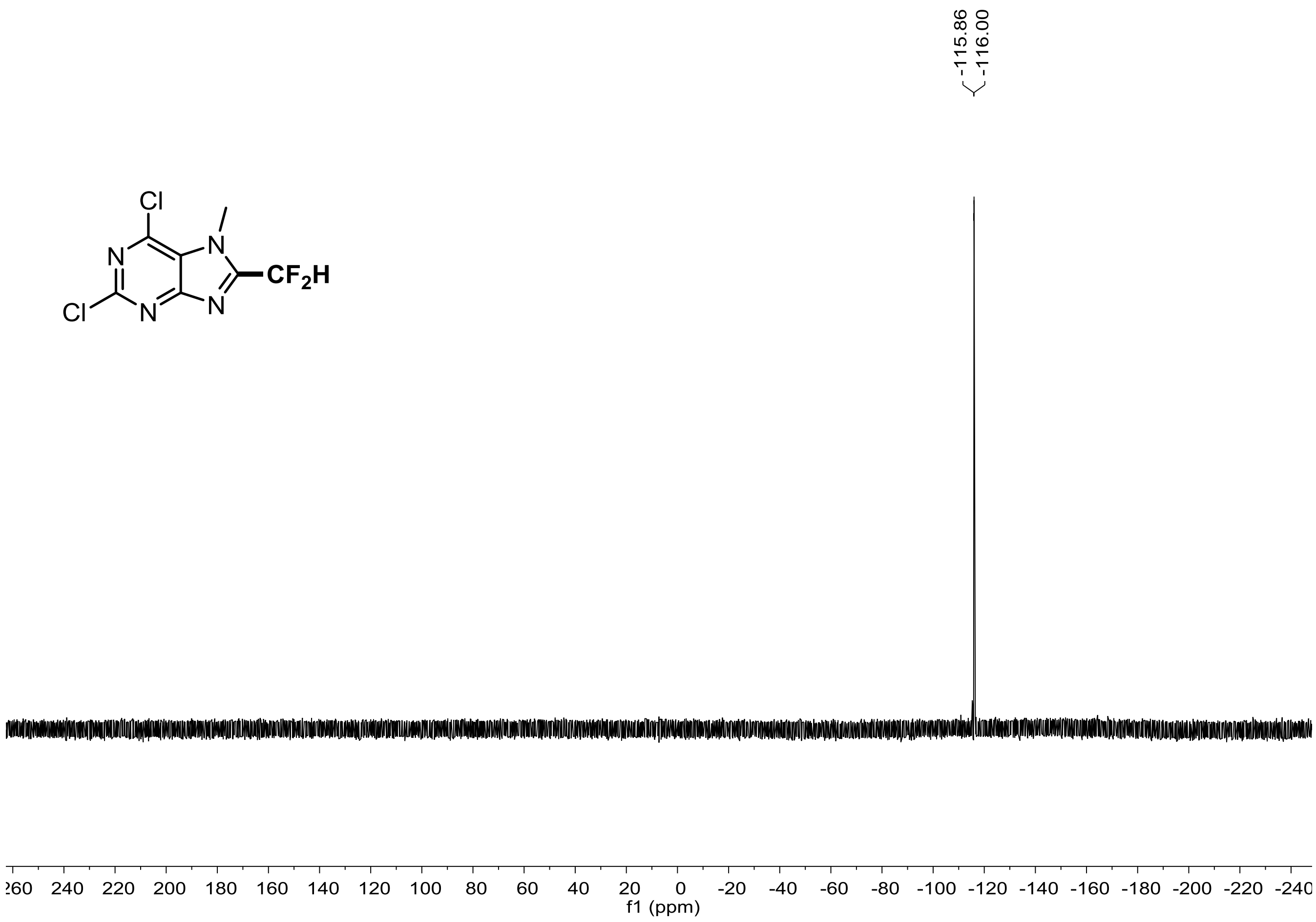

Supplementary Figure 90. ^{19}F NMR Spectrum of **5r**

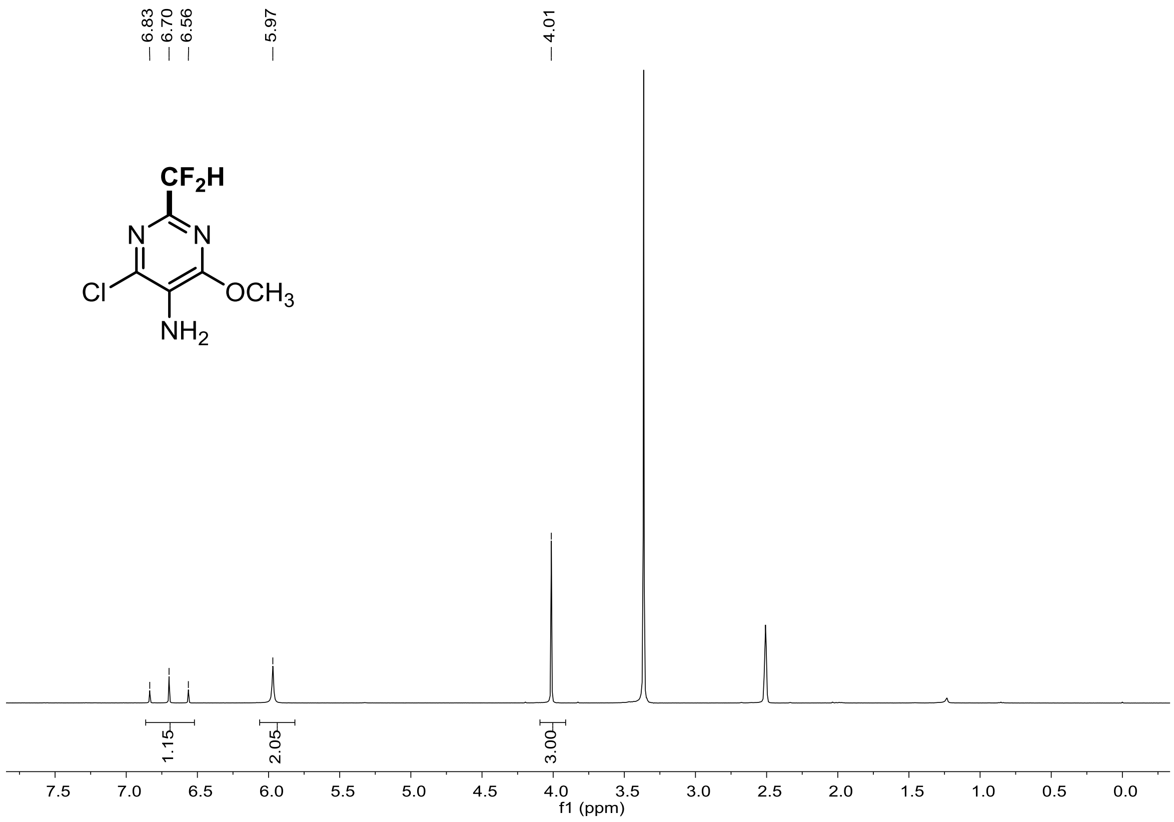

Supplementary Figure 91. ^{1}H NMR Spectrum of **5s**

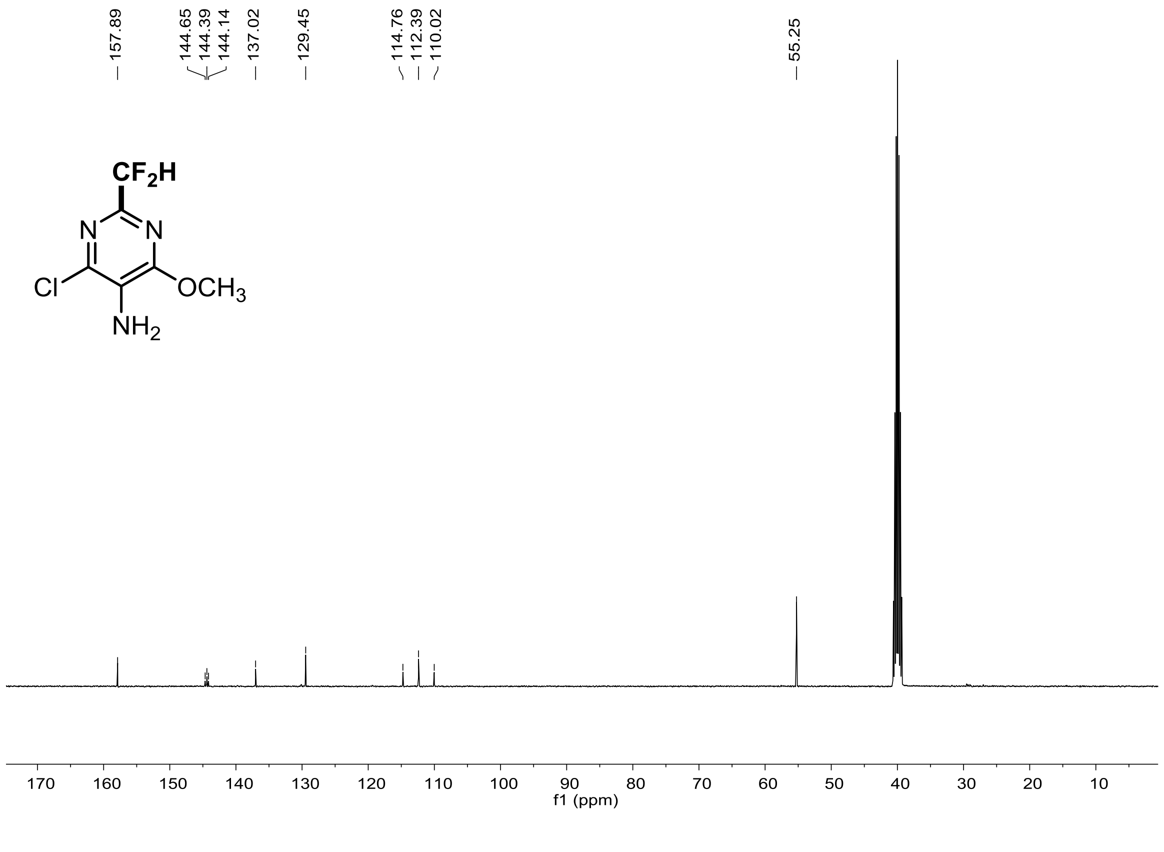

Supplementary Figure 92. ^{13}C NMR Spectrum of **5s**

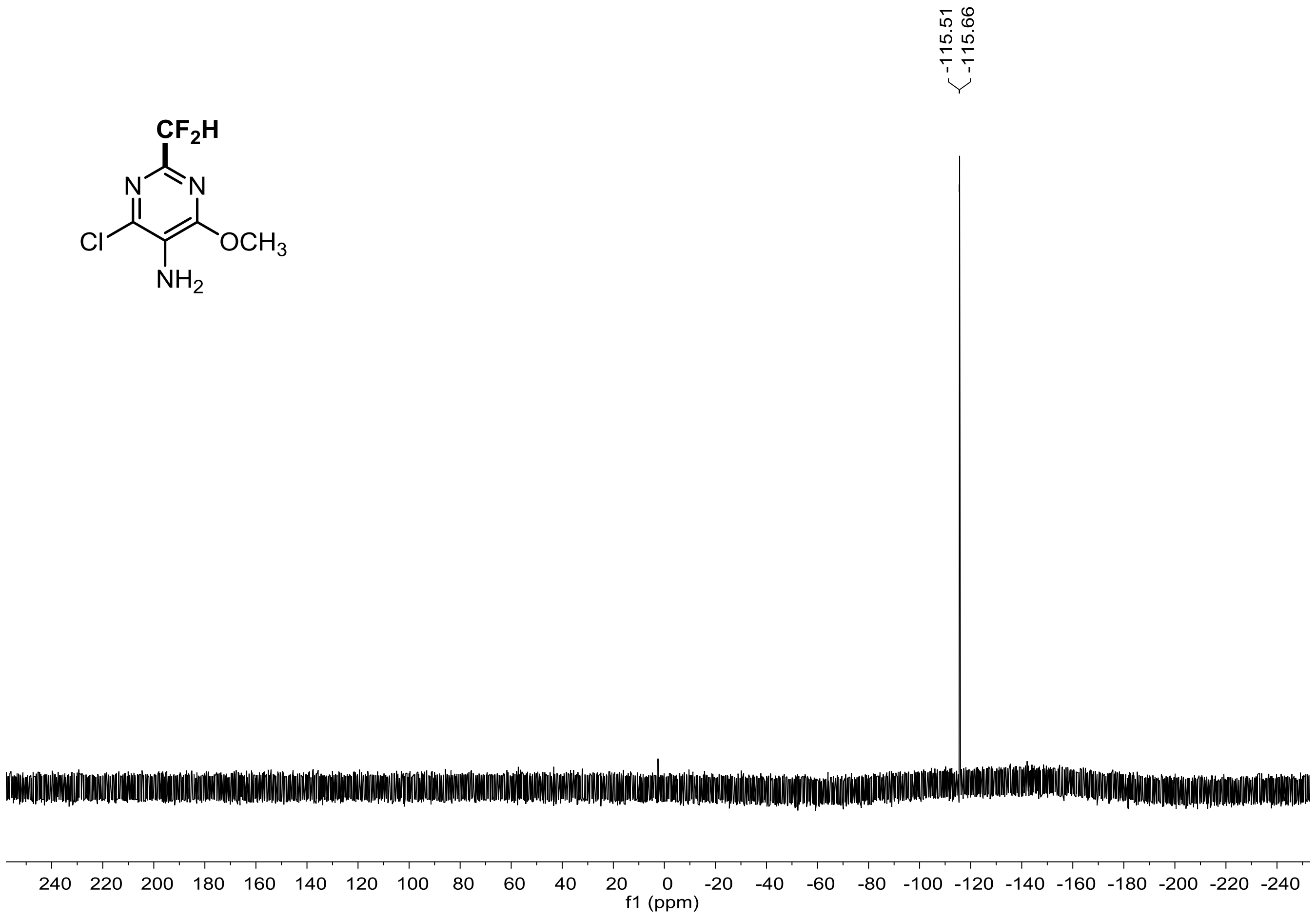

Supplementary Figure 93. ^{19}F NMR Spectrum of **5s**

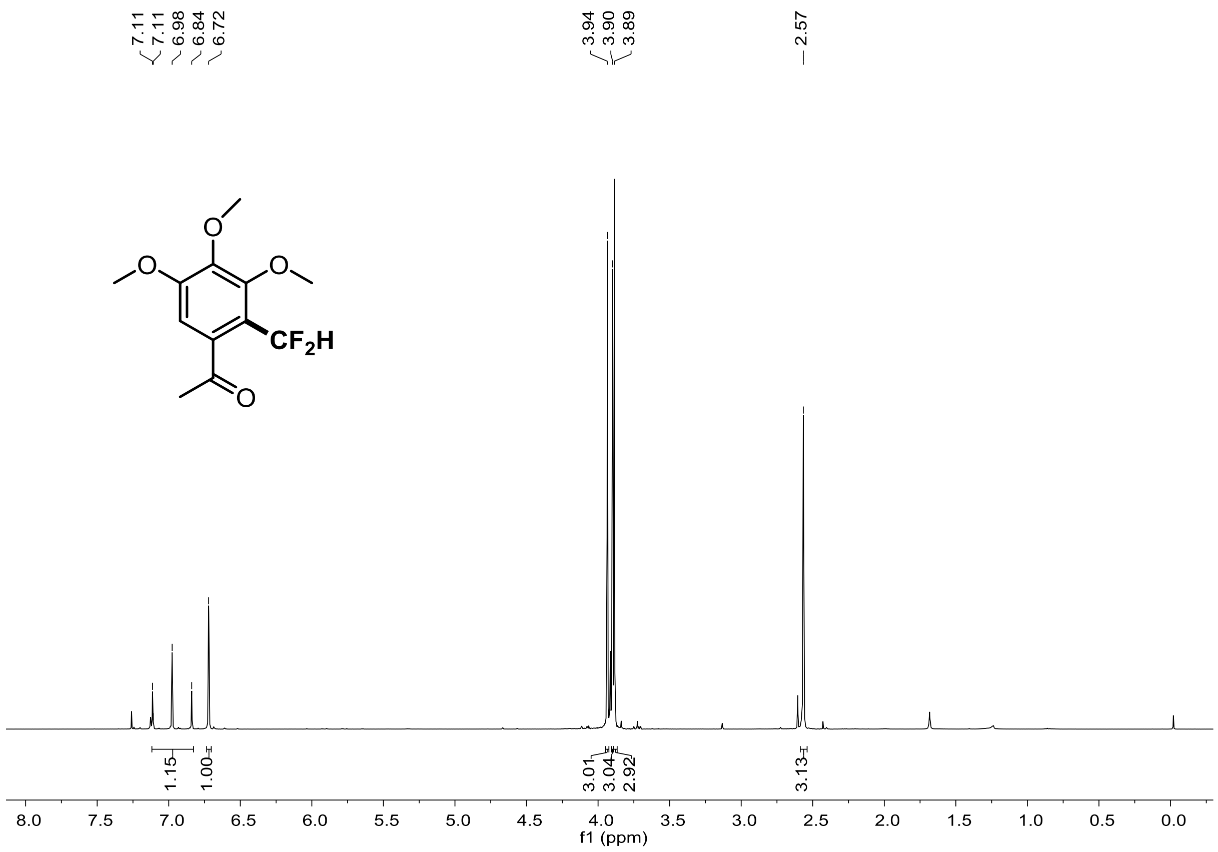

Supplementary Figure 94. ^{1}H NMR Spectrum of **5t**

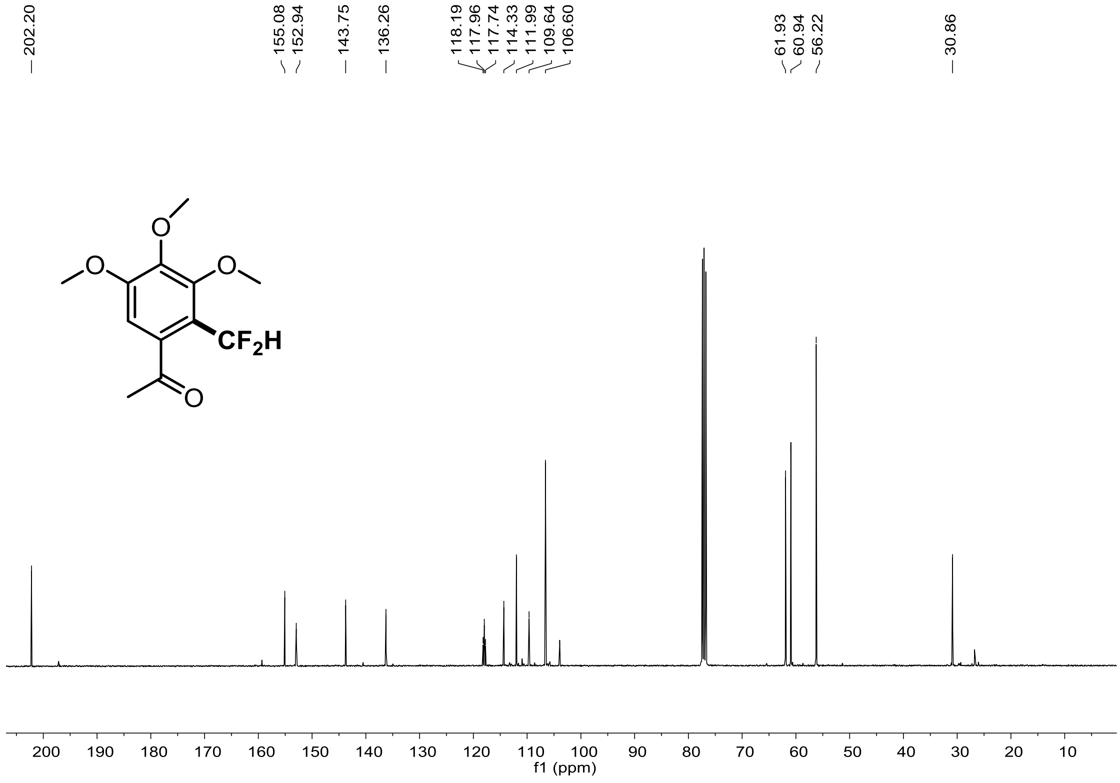

Supplementary Figure 95. ^{13}C NMR Spectrum of **5t**

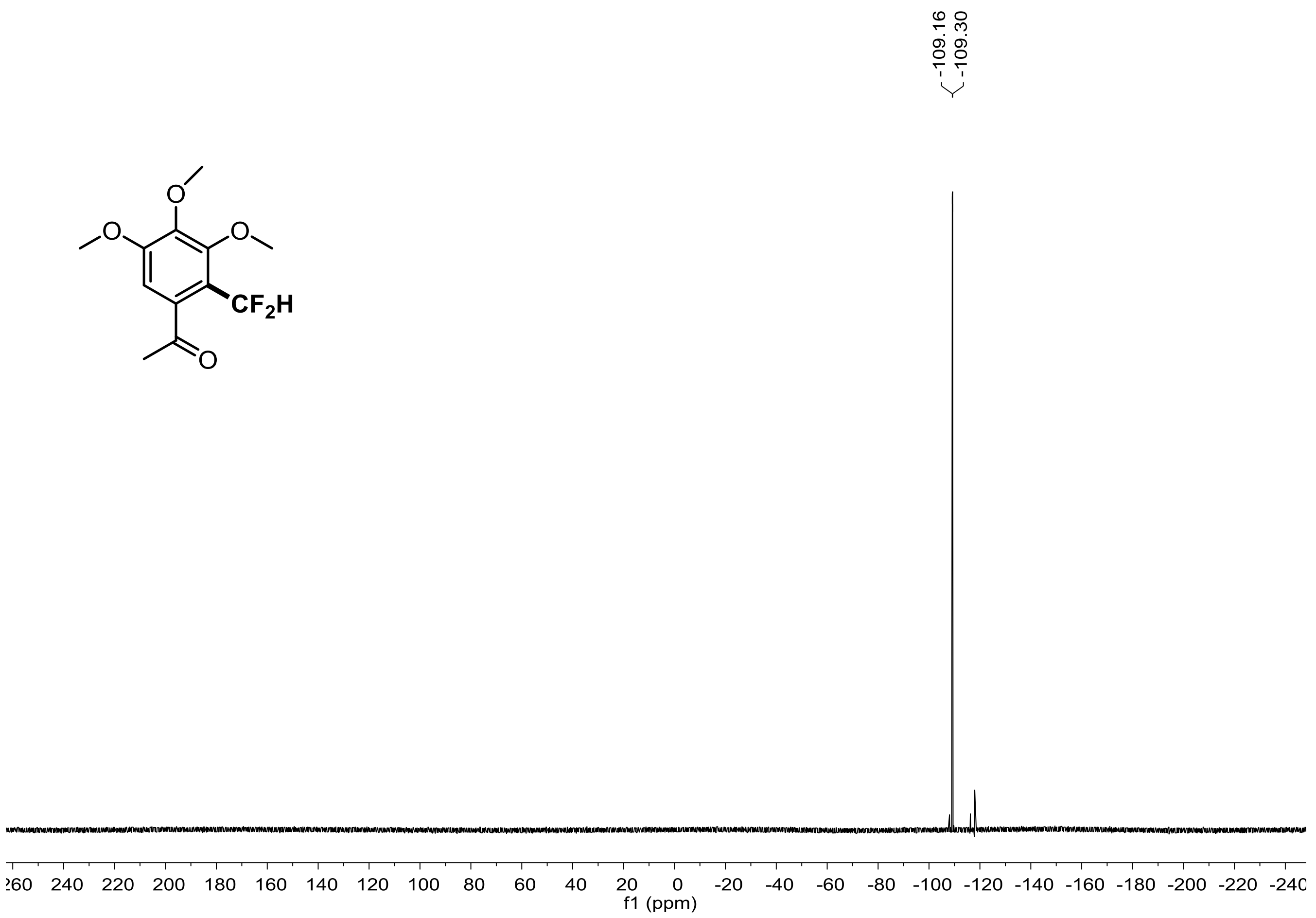

Supplementary Figure 96. ^{19}F NMR Spectrum of **5t**

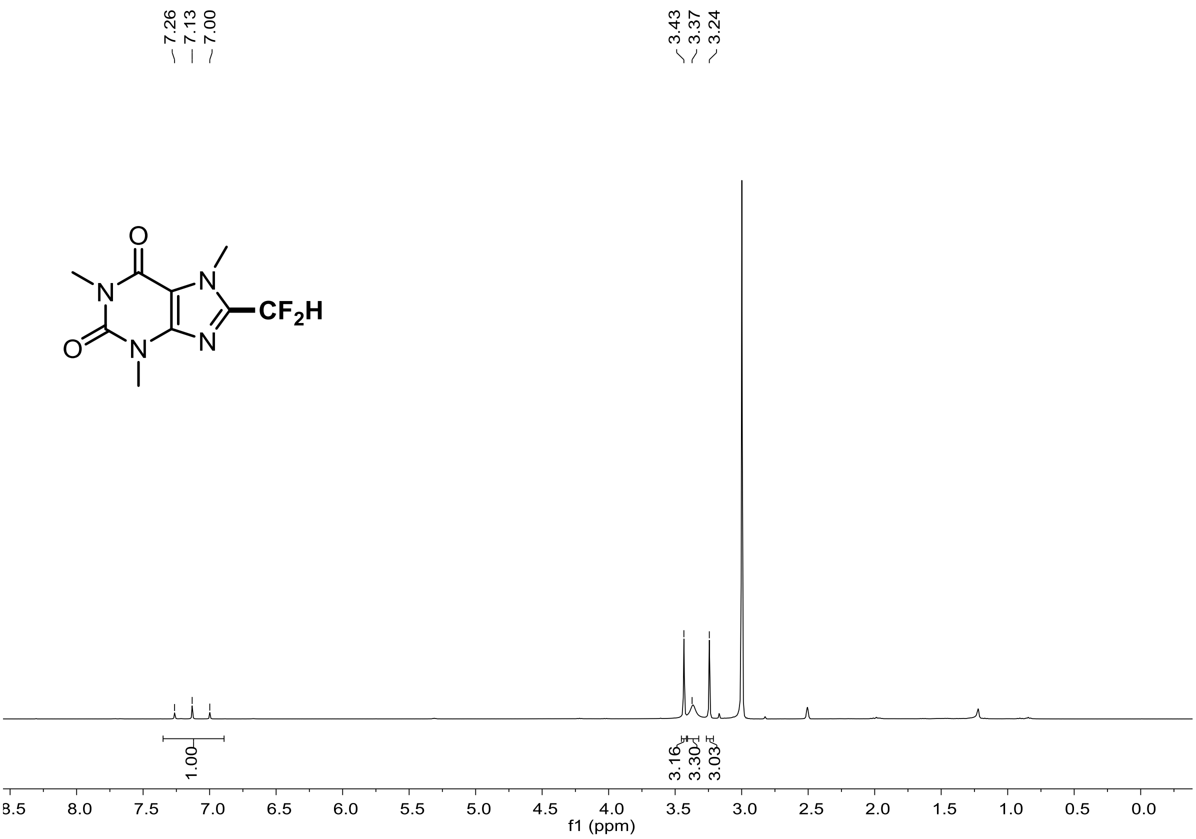

Supplementary Figure 97. ^{1}H NMR Spectrum of **6a**

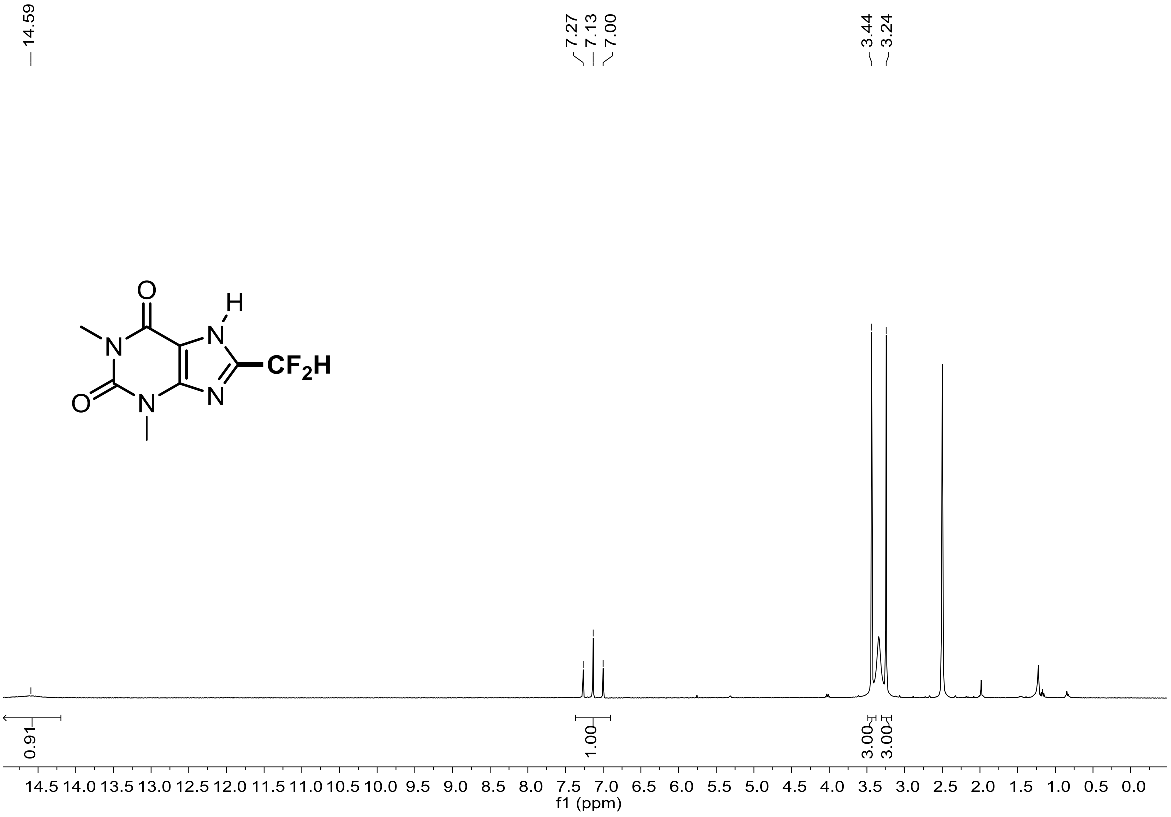

Supplementary Figure 98. ^{1}H NMR Spectrum of **6b**

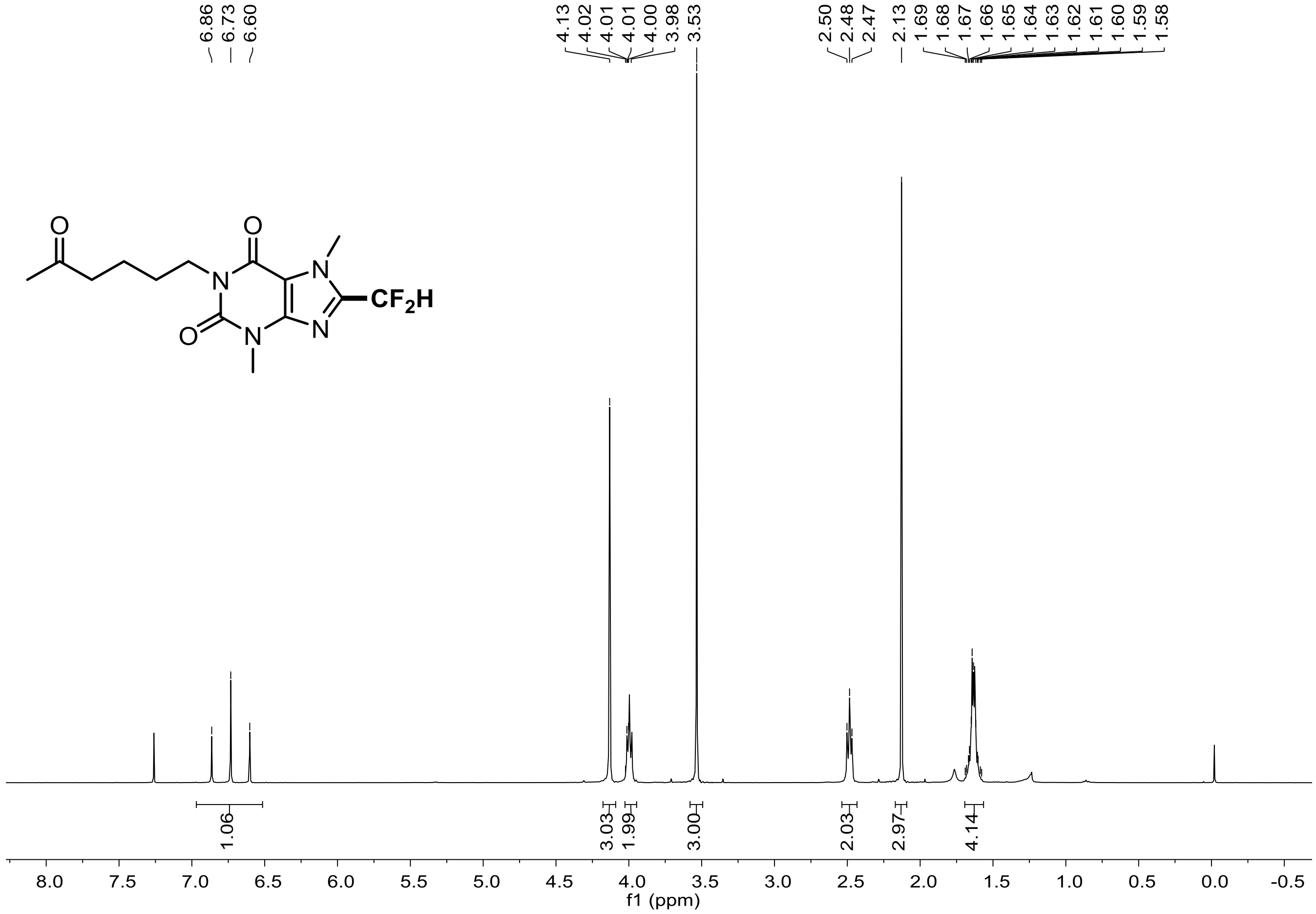

Supplementary Figure 99. ^{1}H NMR Spectrum of **6c**

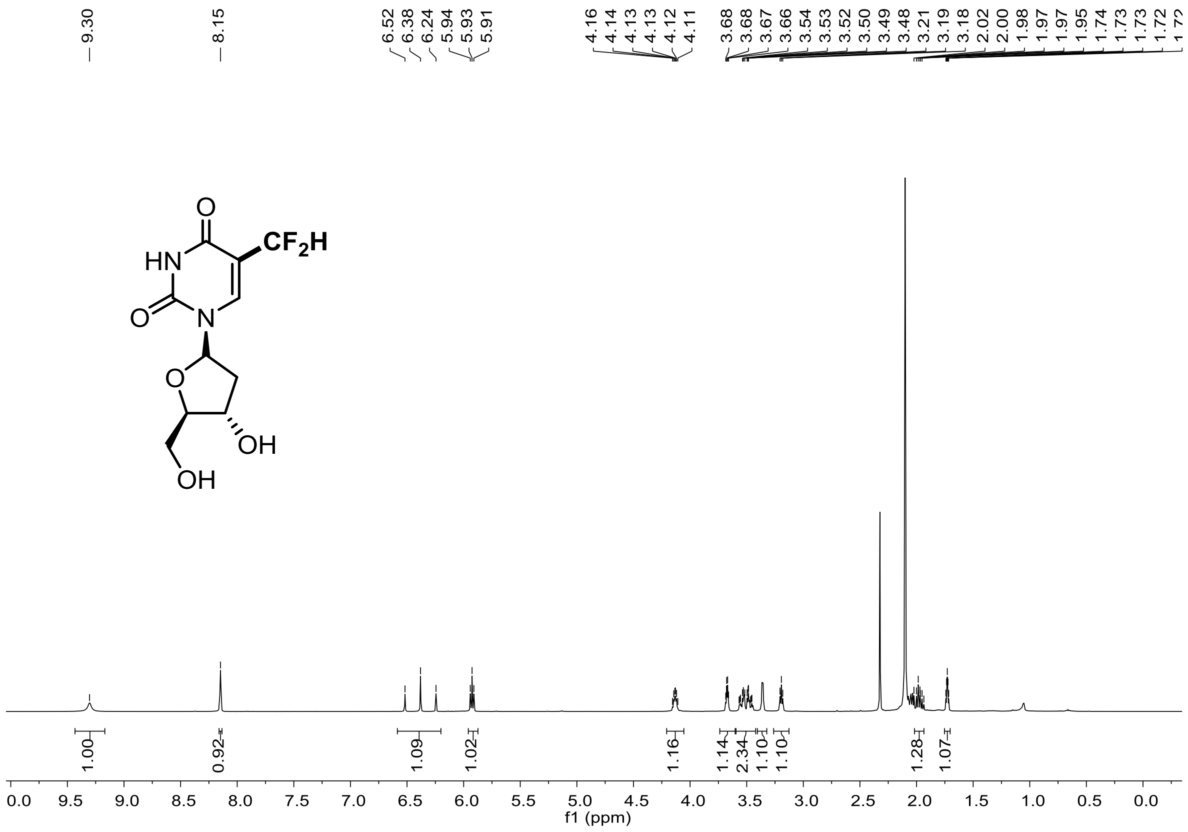

Supplementary Figure 100. ^{1}H NMR Spectrum of **6d**

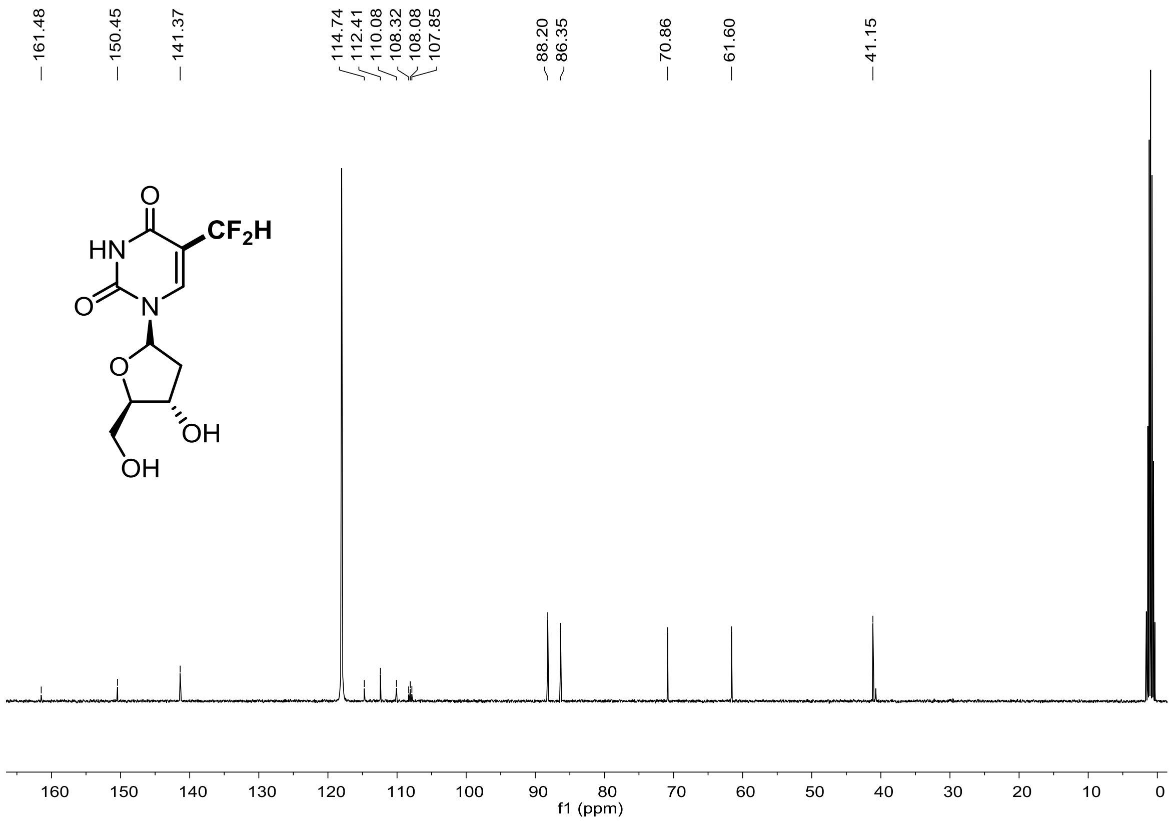

Supplementary Figure 101. ^{13}C NMR Spectrum of **6d**

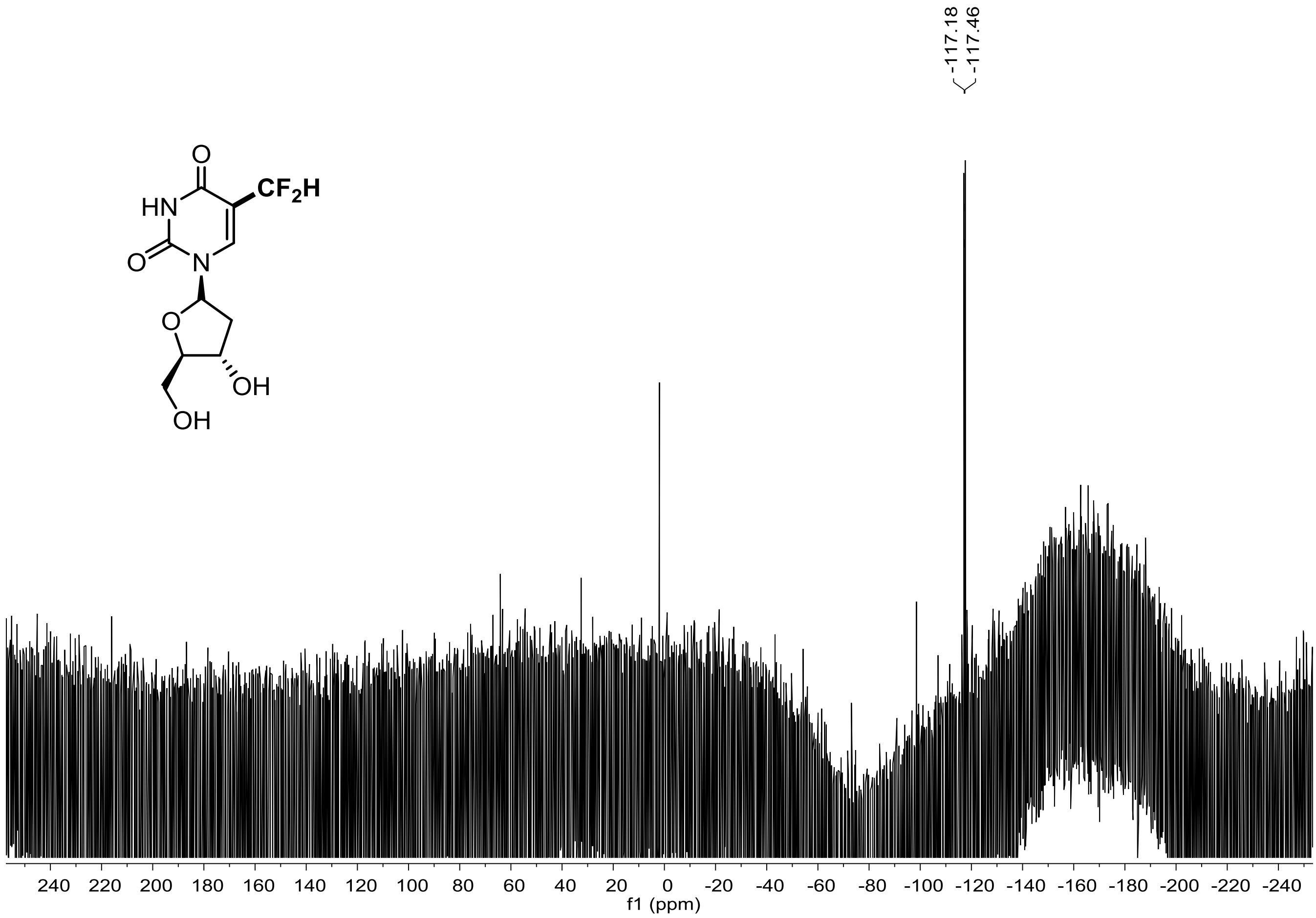

Supplementary Figure 102. ^{19}F NMR Spectrum of **6d**

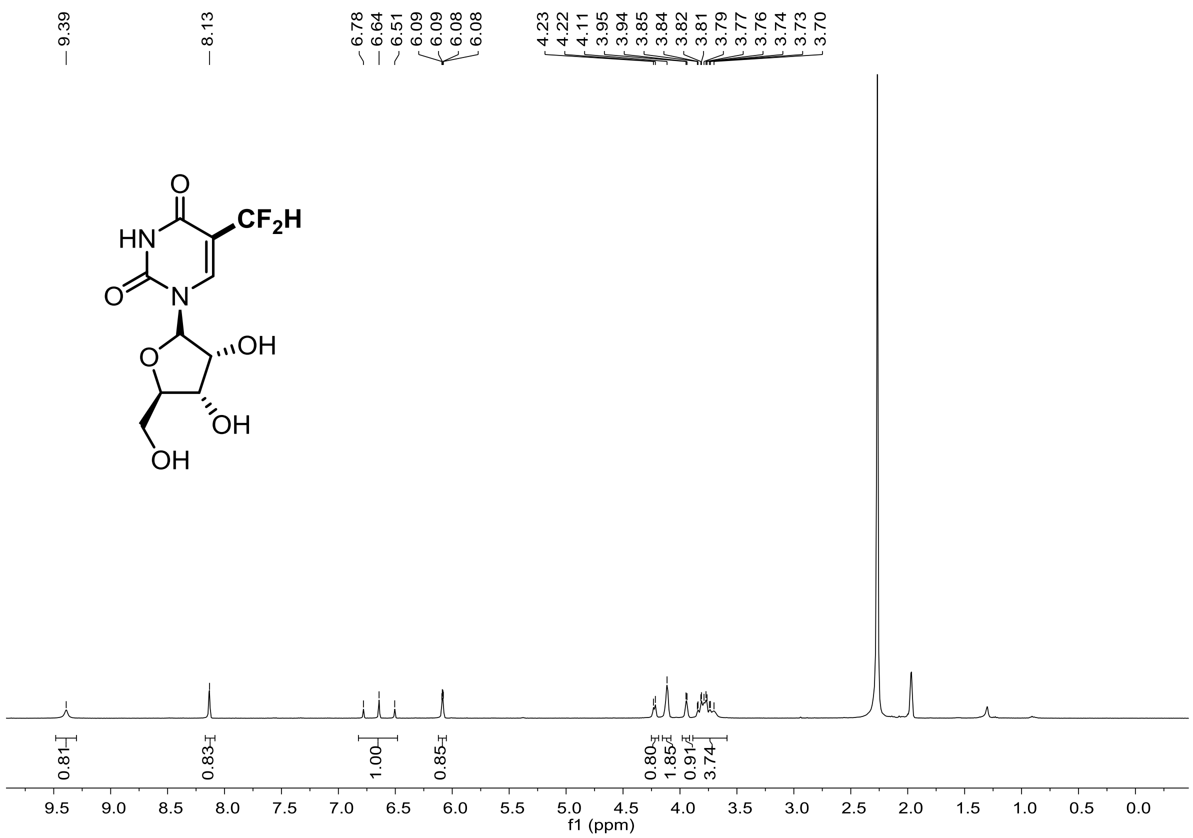

Supplementary Figure 102. ^{1}H NMR Spectrum of **6e**

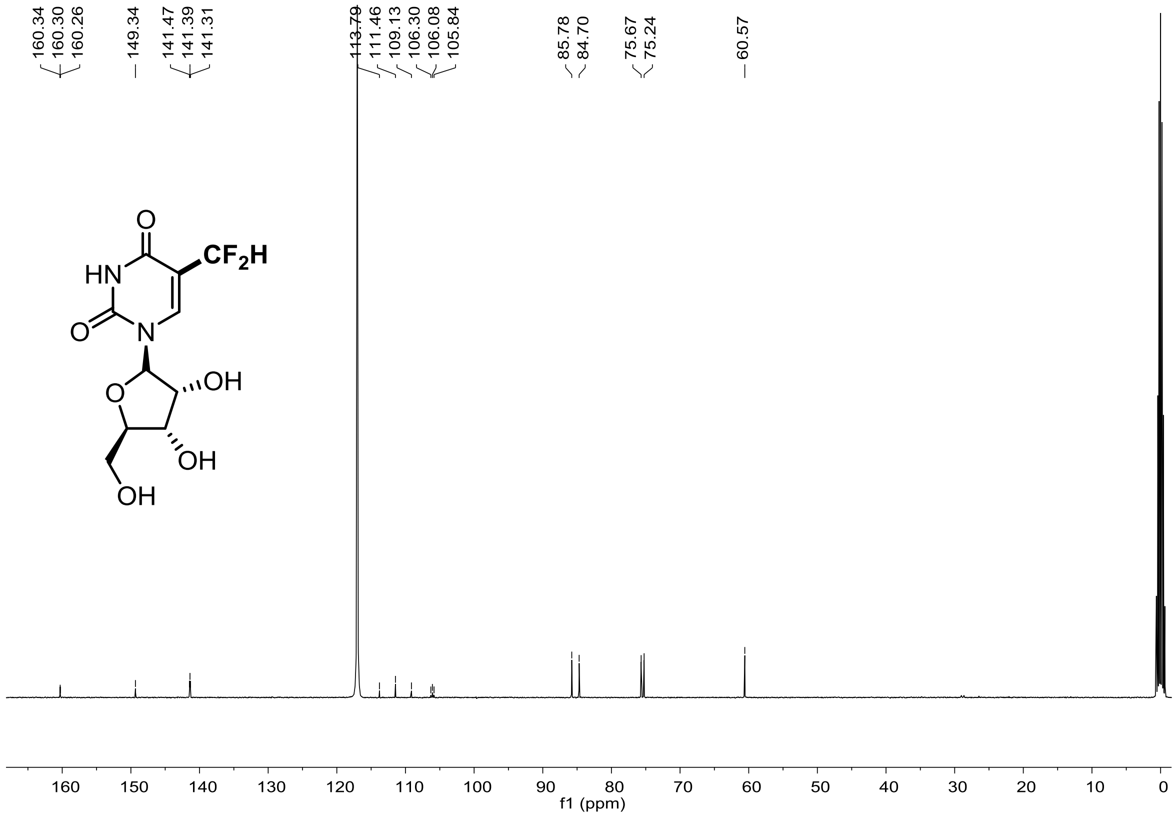

Supplementary Figure 103. ^{13}C NMR Spectrum of **6e**

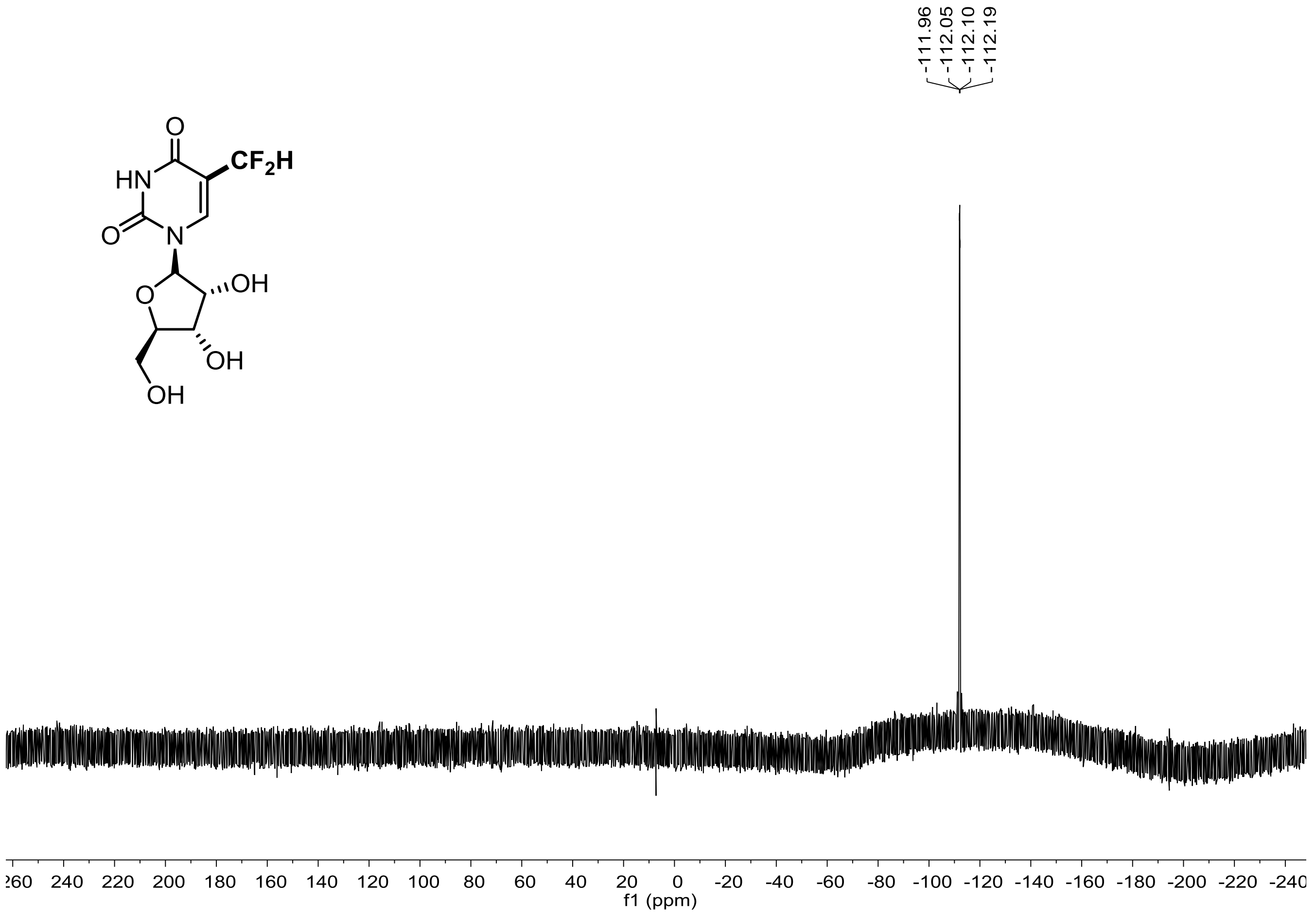

Supplementary Figure 104. [19] F NMR Spectrum of **6e**

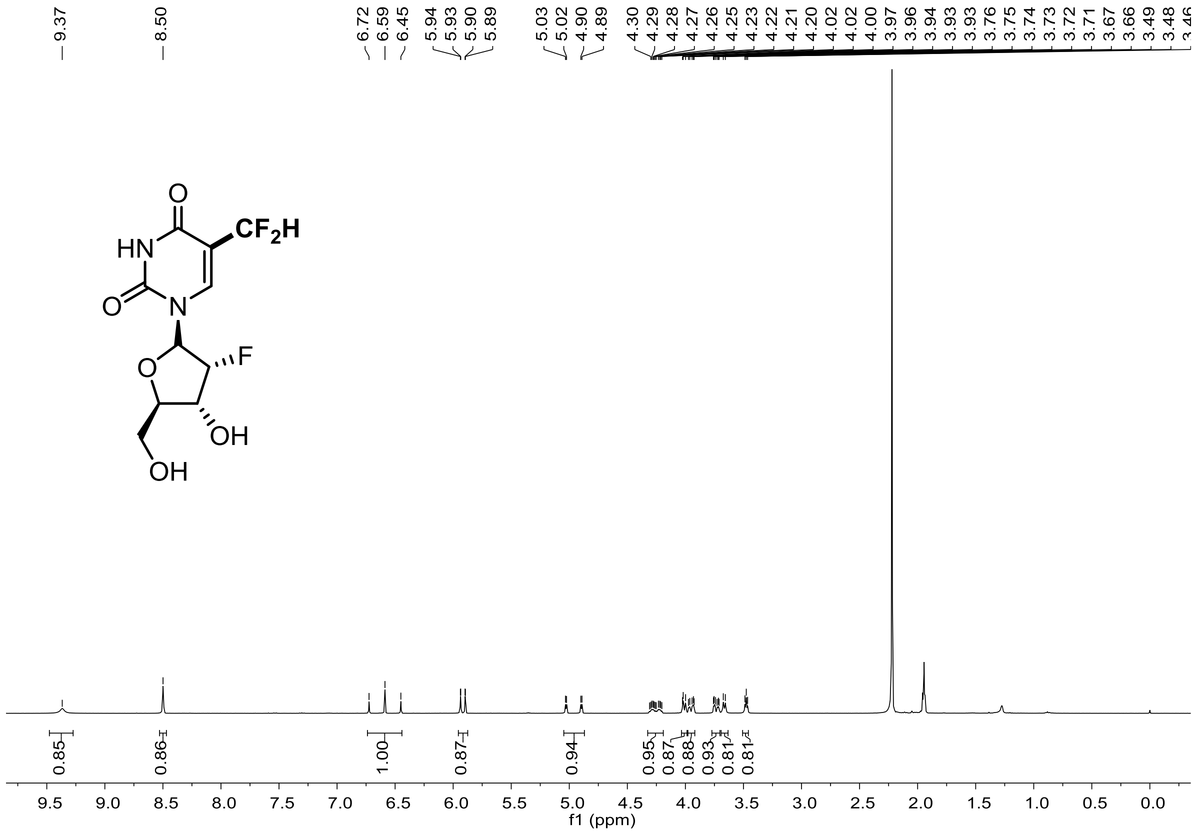

Supplementary Figure 105. ^{1}H NMR Spectrum of **6f**

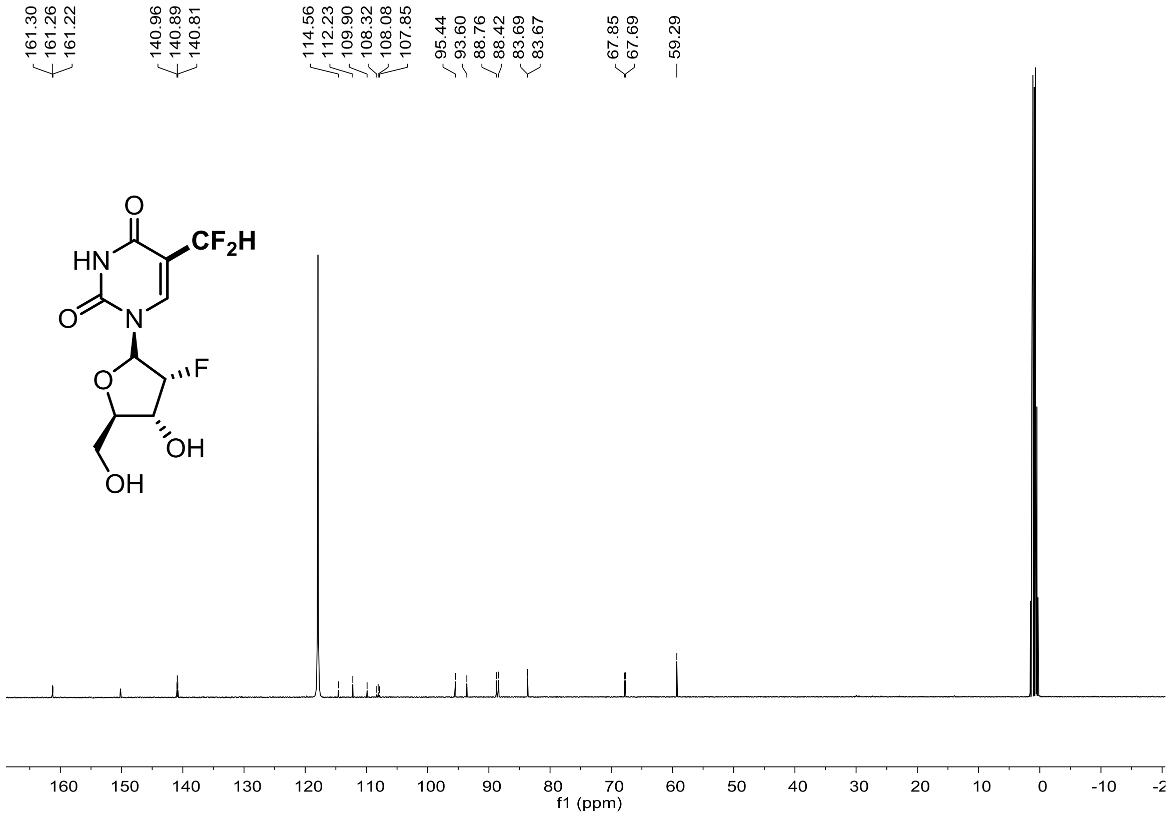

Supplementary Figure 106. ^{13}C NMR Spectrum of **6f**

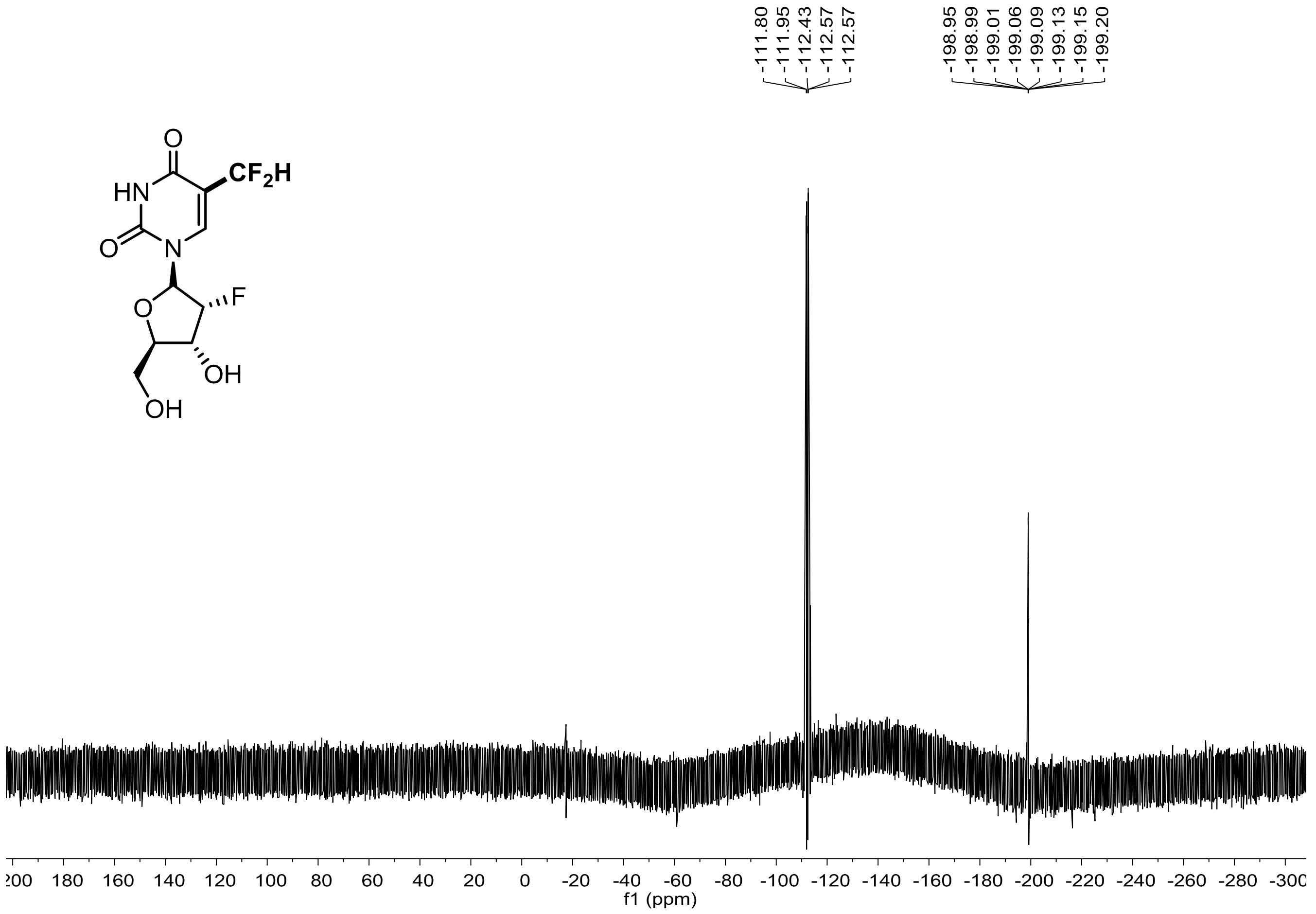

Supplementary Figure 107. ^{19}F NMR Spectrum of **6f**

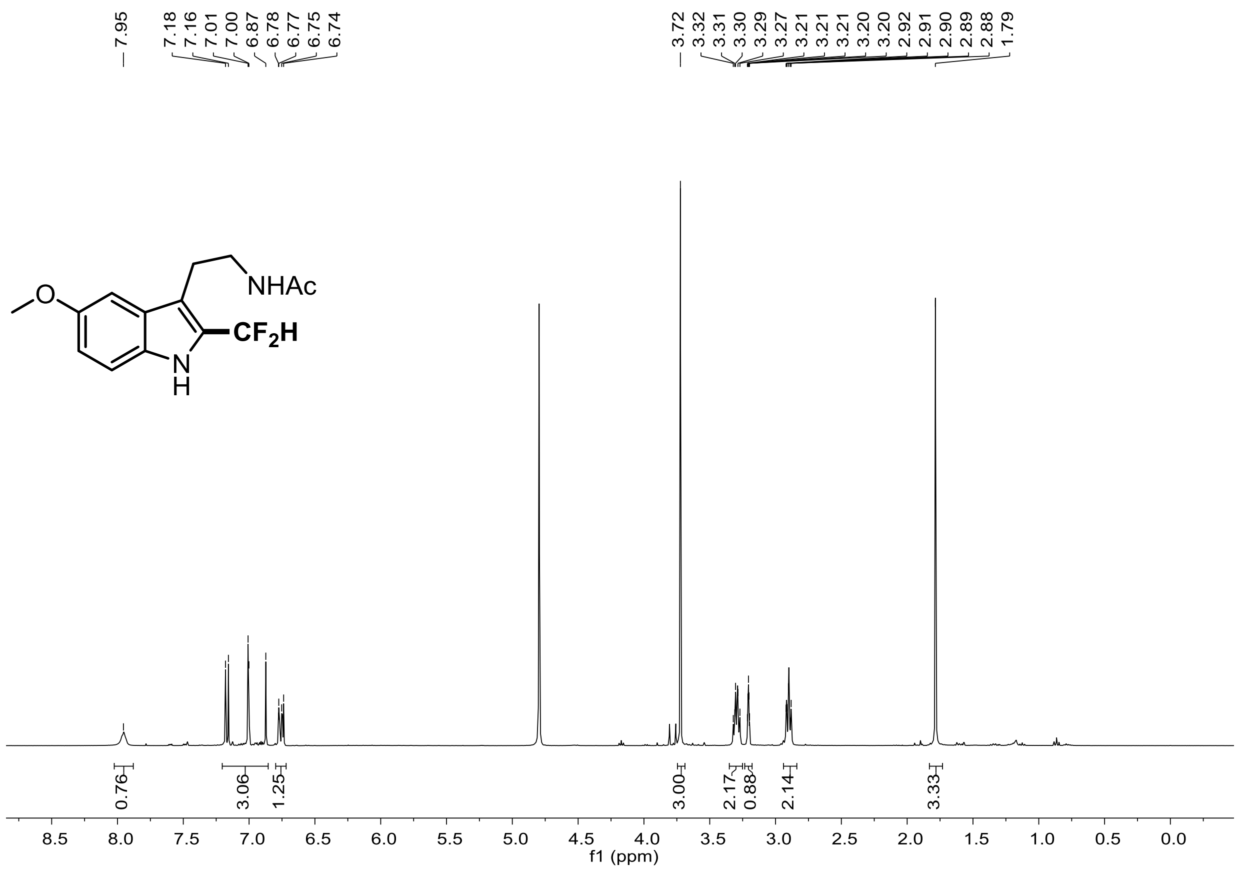

Supplementary Figure 108. ^{1}H NMR Spectrum of **6g**

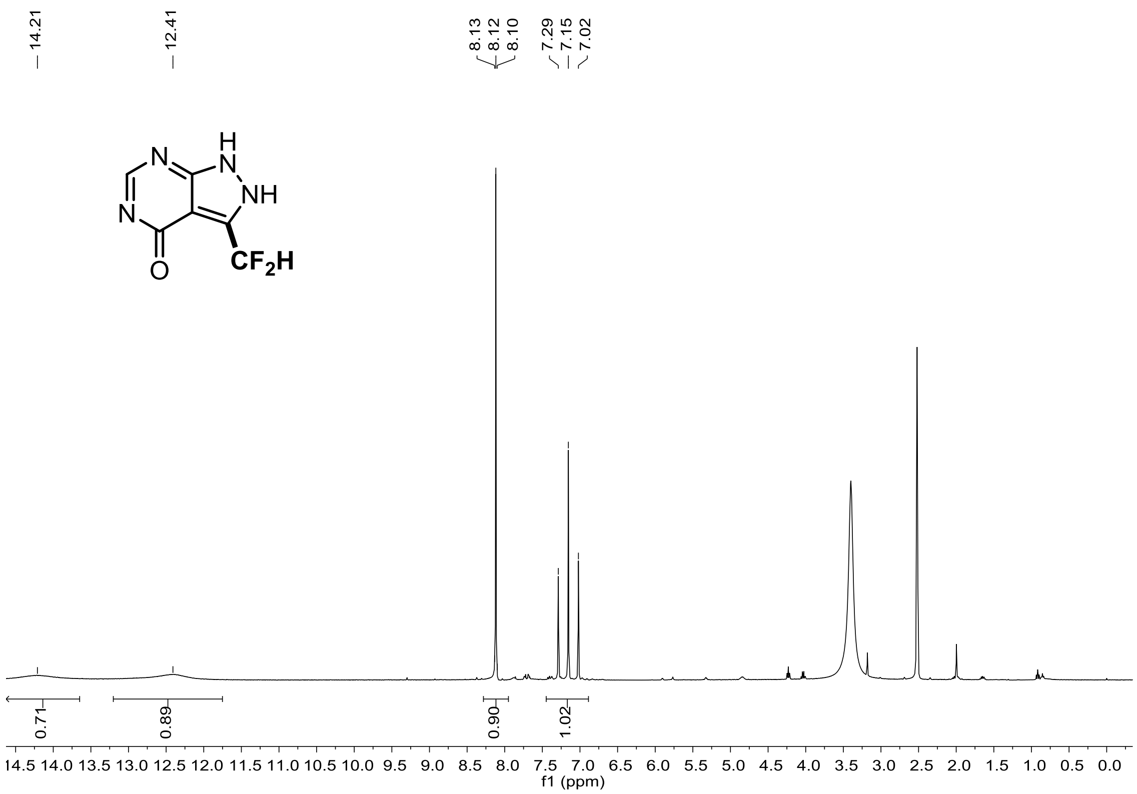

Supplementary Figure 109. ^{1}H NMR Spectrum of **6h**

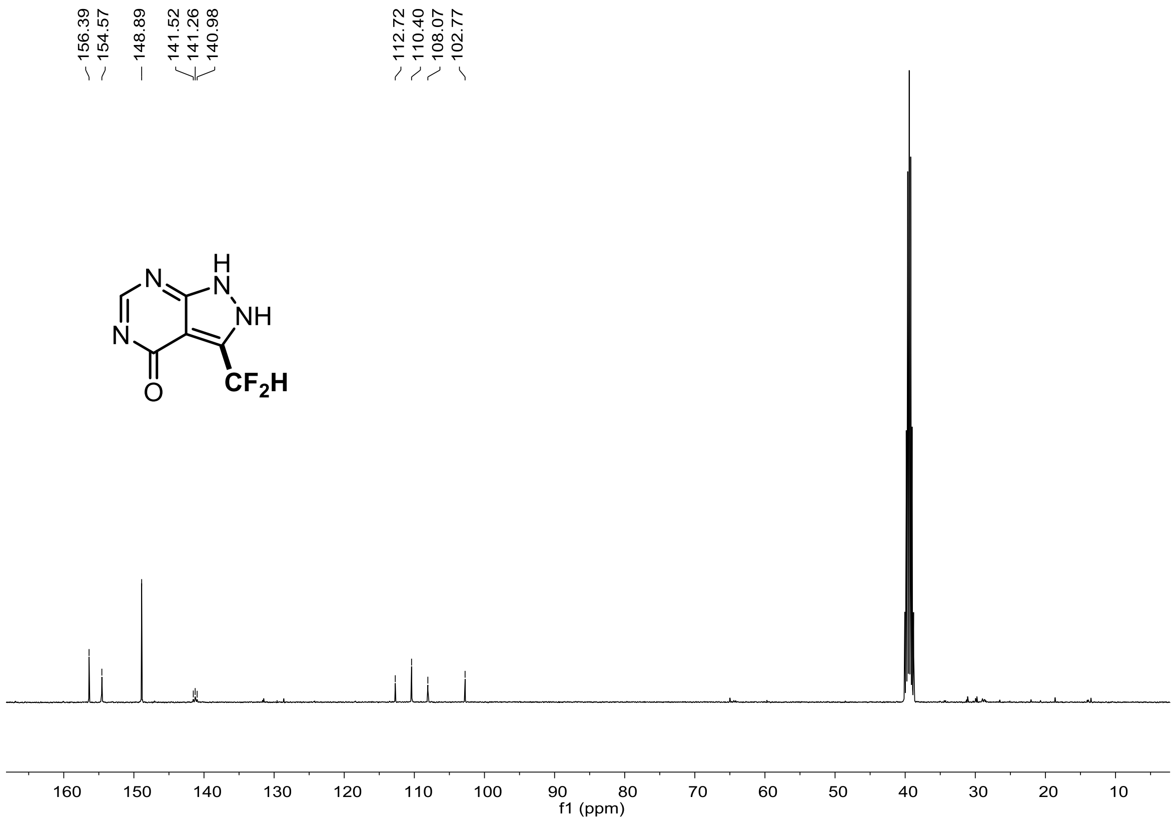

Supplementary Figure 110. ^{13}C NMR Spectrum of **6h**

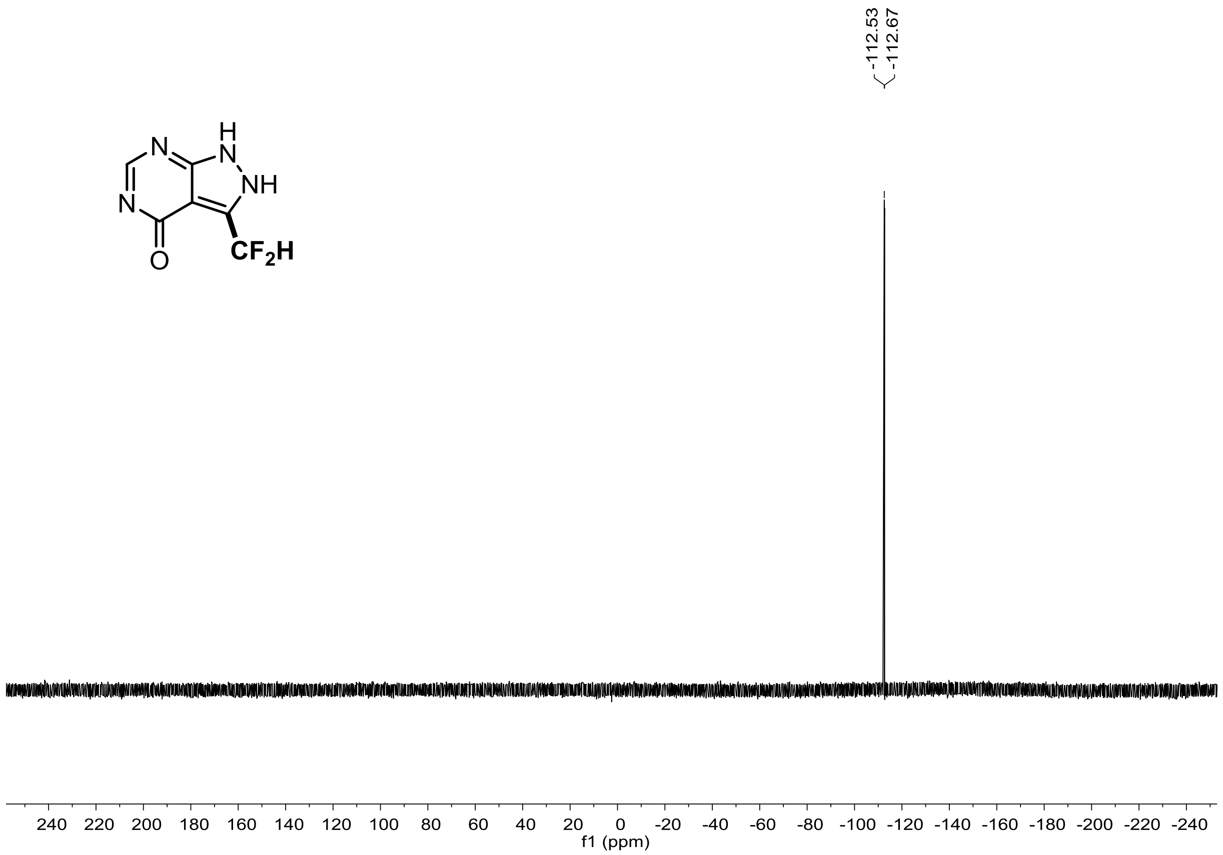

Supplementary Figure 111. ^{19}F NMR Spectrum of **6h**

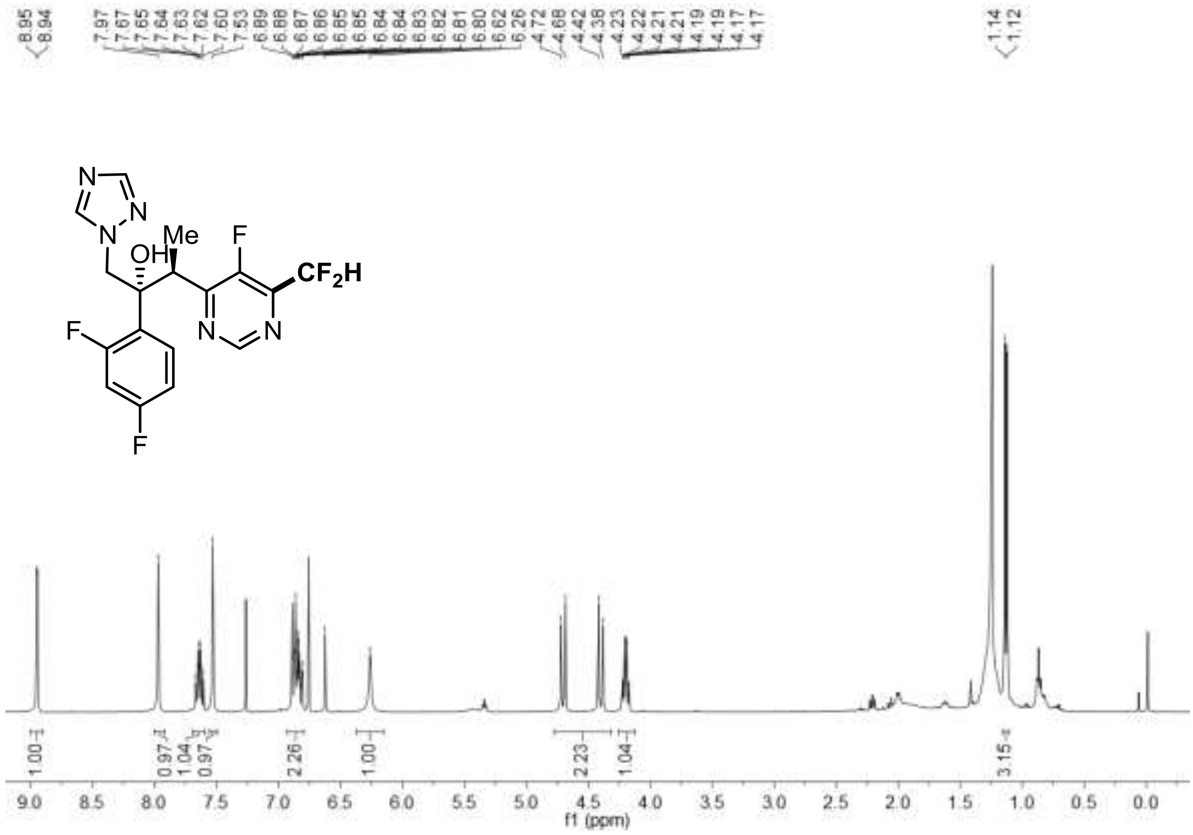

Supplementary Figure 112. ^{1}H NMR Spectrum of **6i**

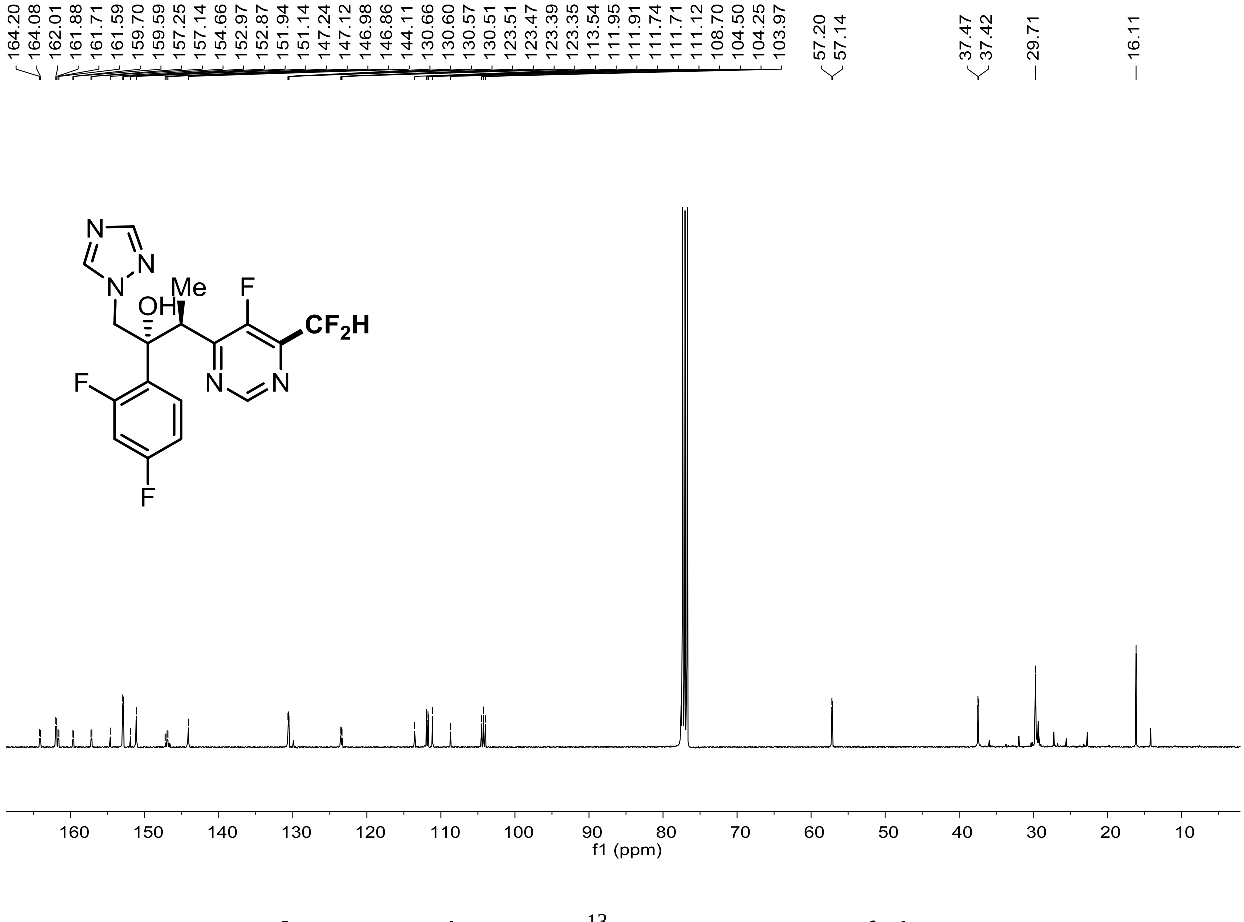

Supplementary Figure 113. ^{13}C NMR Spectrum of **6i**

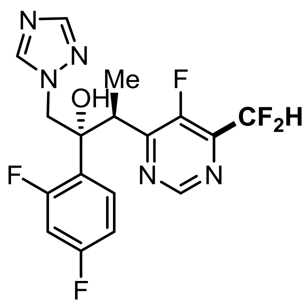

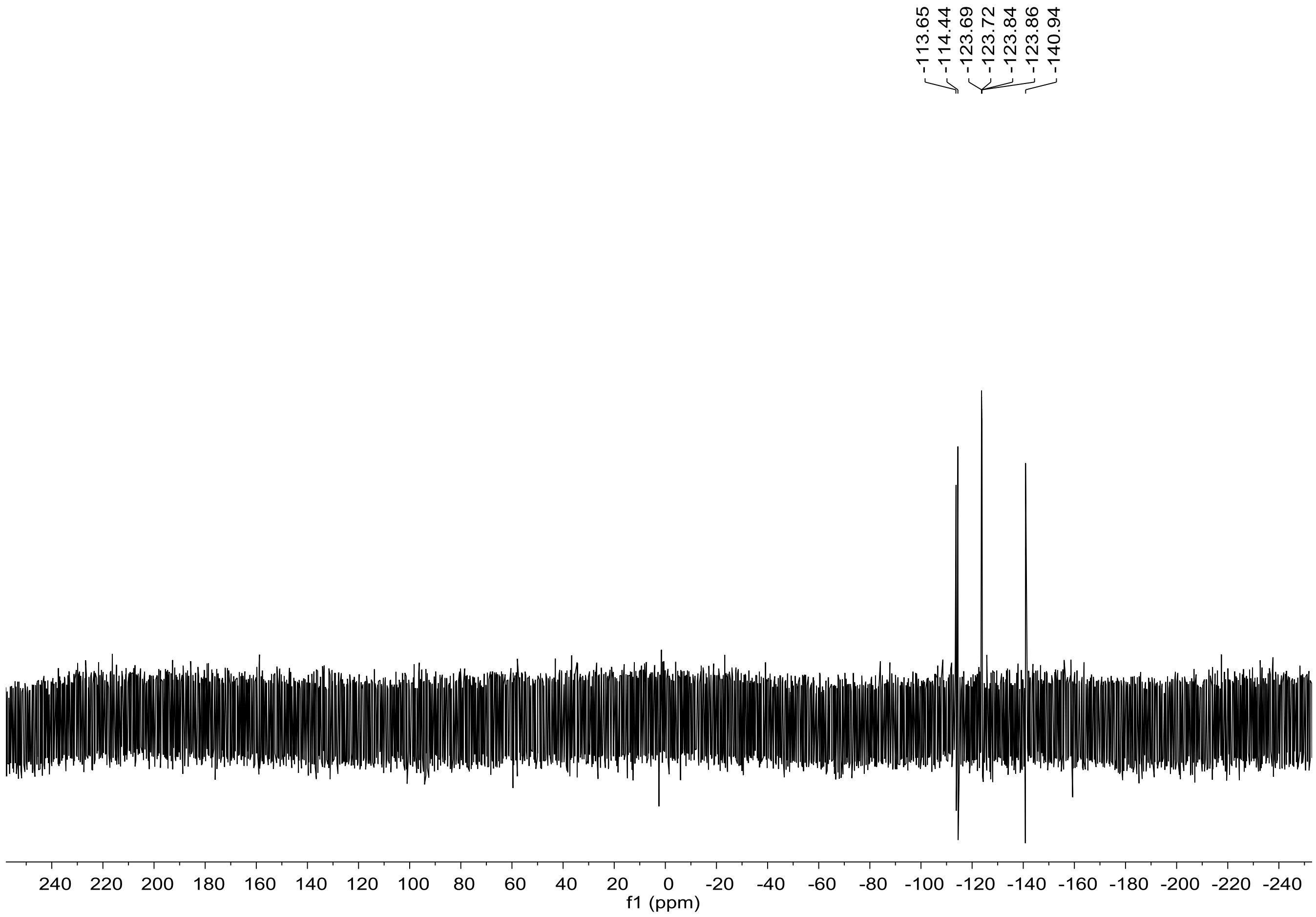

Supplementary Figure 114. ^{13}F NMR Spectrum of **6i**

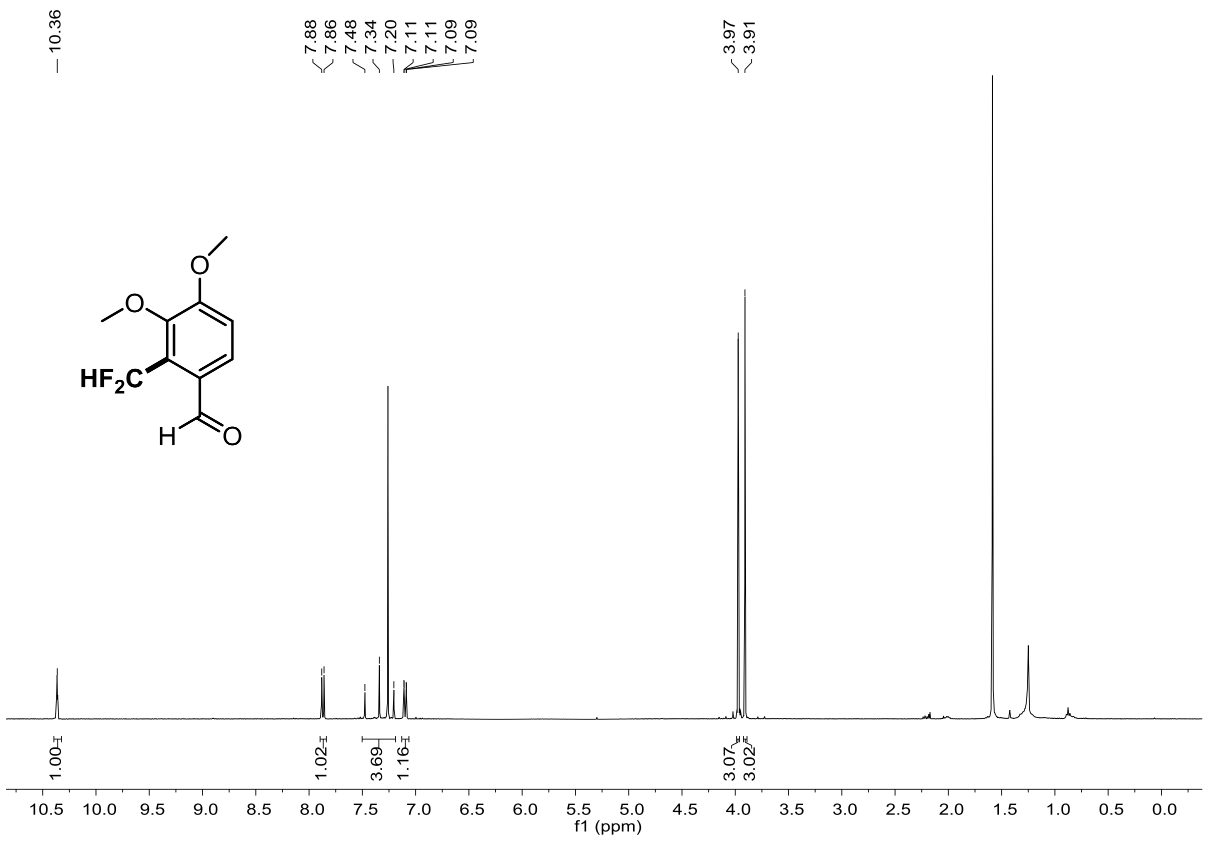

Supplementary Figure 115. ^{1}H NMR Spectrum of **6j**

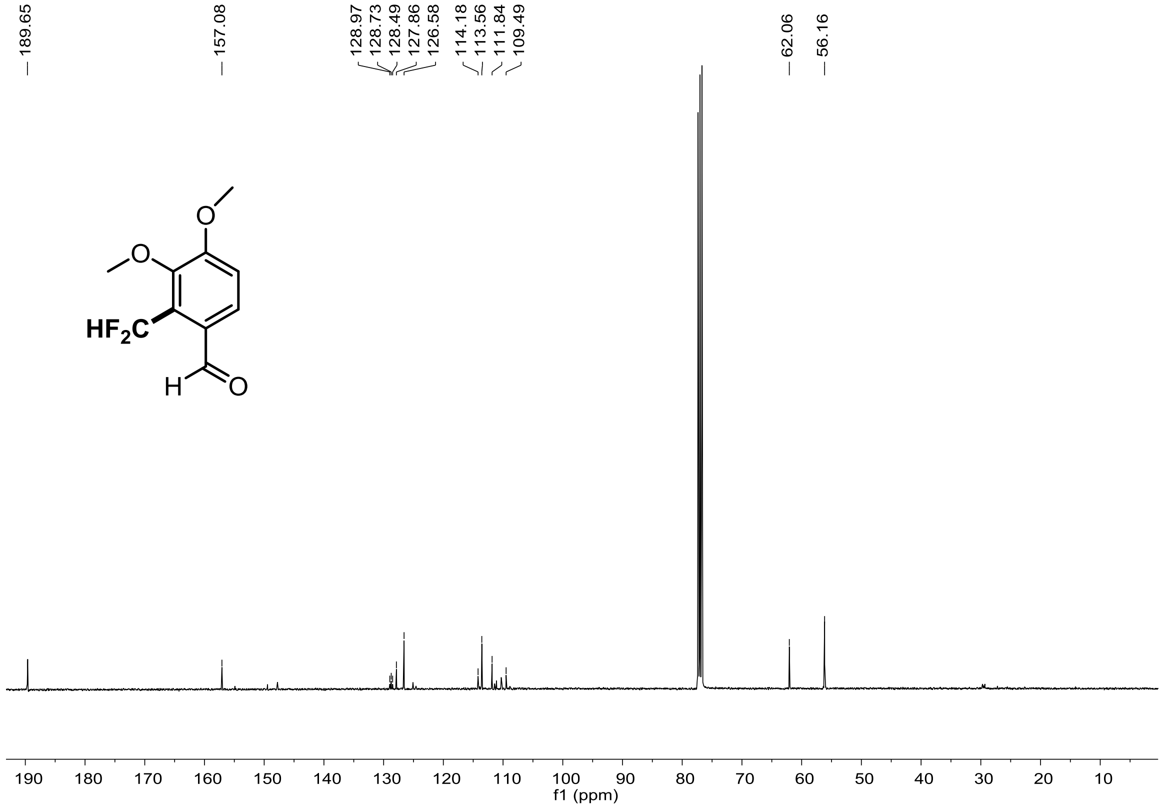

Supplementary Figure 116. ^{13}C NMR Spectrum of **6j**

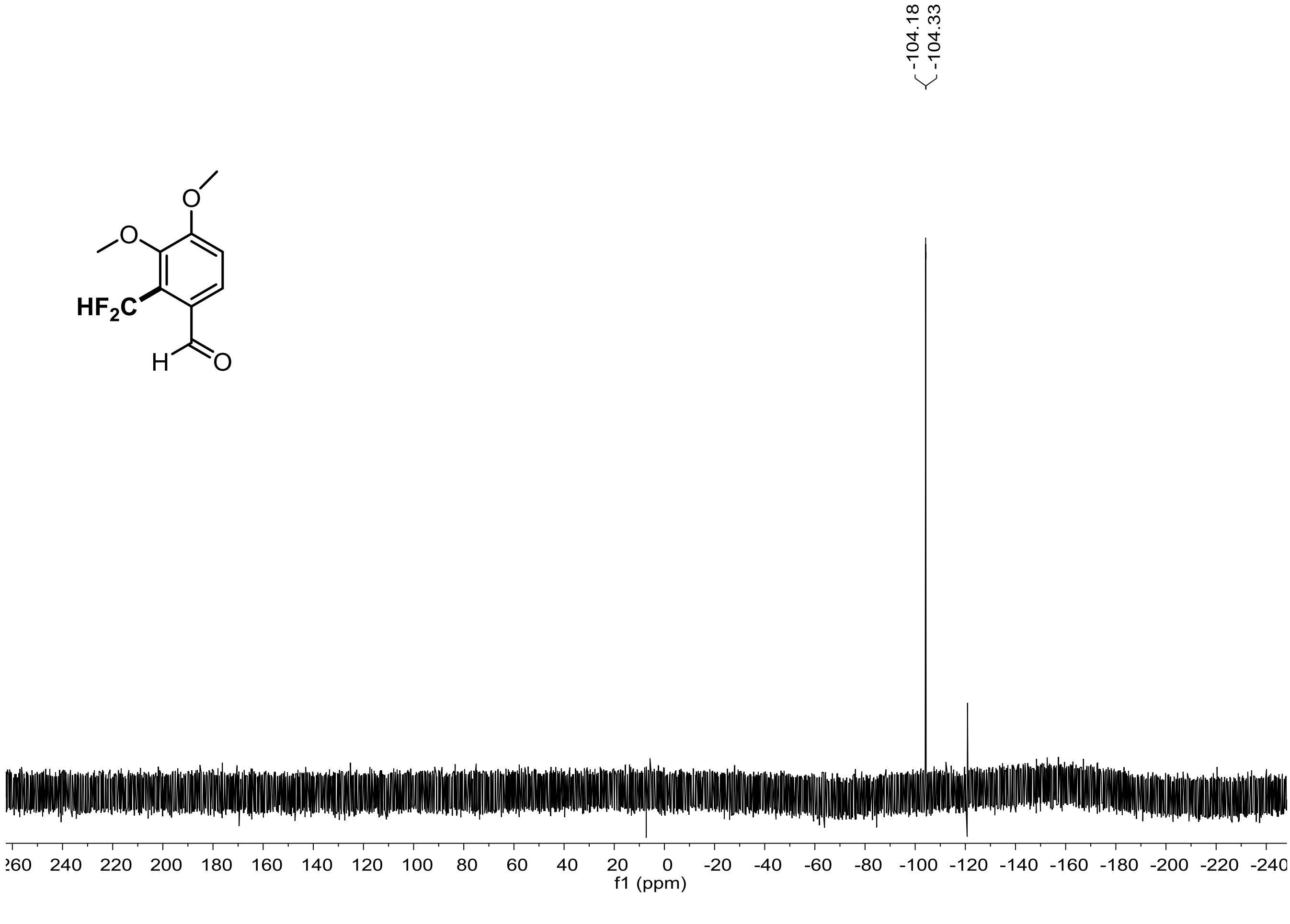

Supplementary Figure 117. ^{19}F NMR Spectrum of **6j**

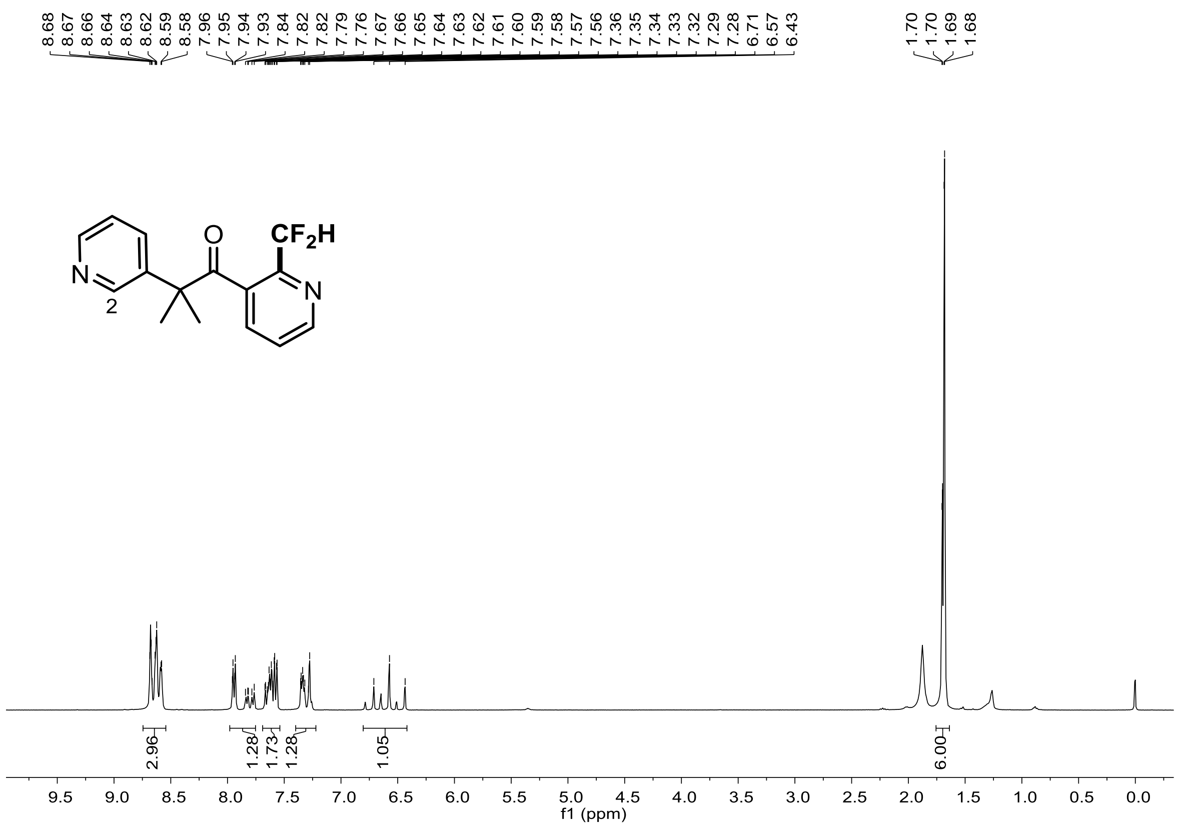

Supplementary Figure 118. ^{1}H NMR Spectrum of **6k**

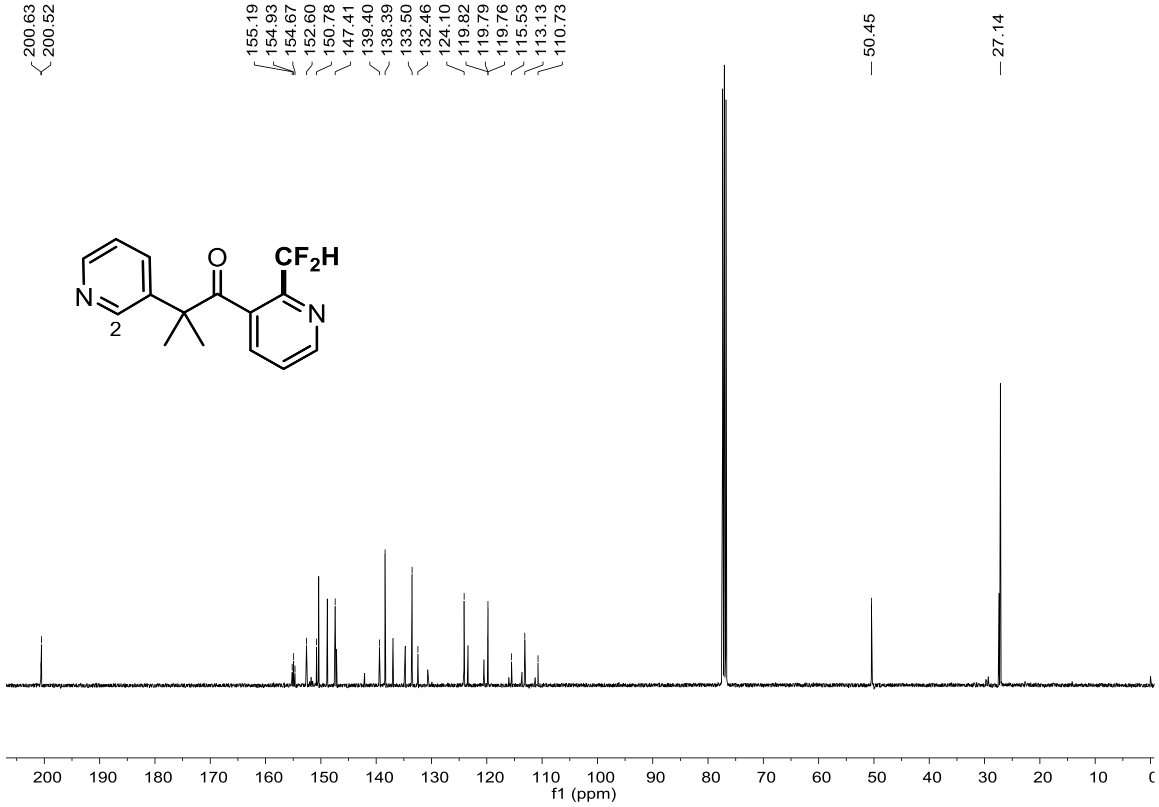

Supplementary Figure 119. ^{13}C NMR Spectrum of **6k**

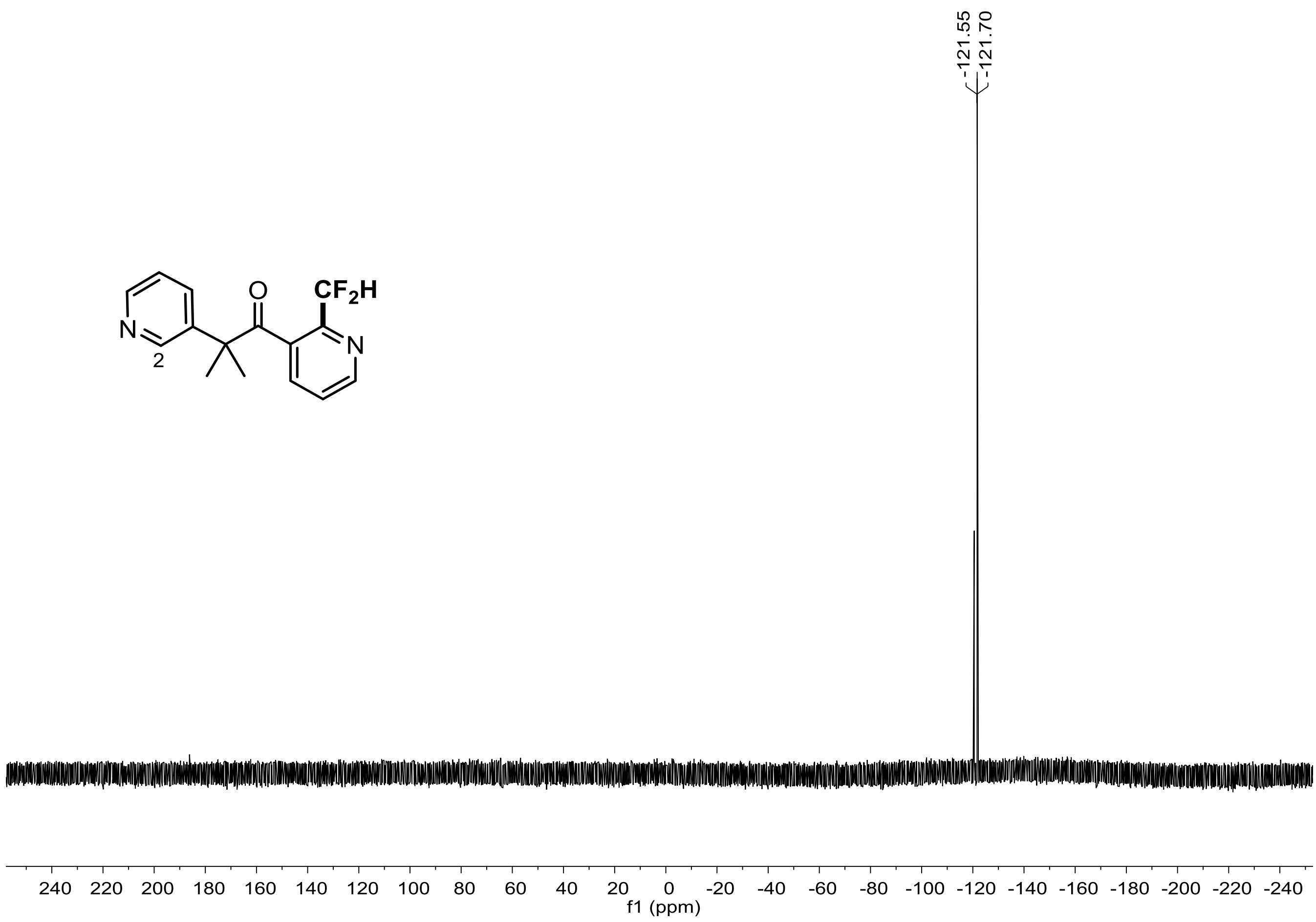

Supplementary Figure 120. ^{19}F NMR Spectrum of **6k**

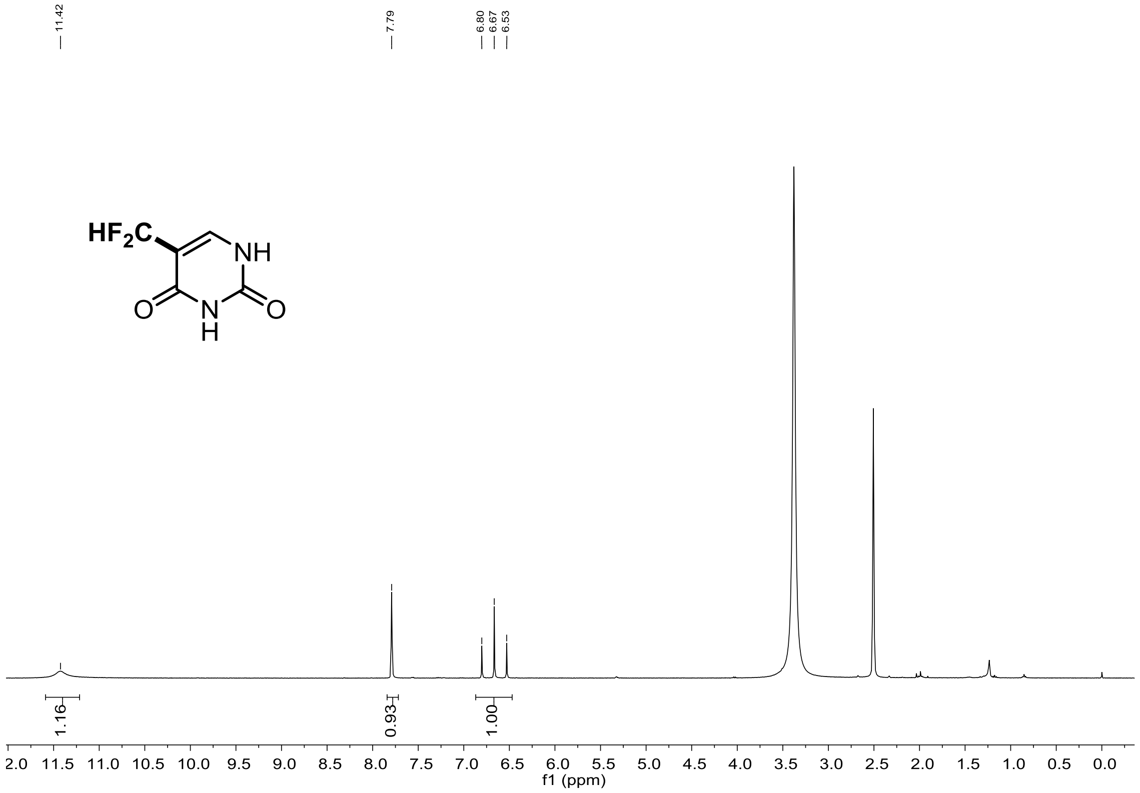

Supplementary Figure 121. ^{1}H NMR Spectrum of **6l**

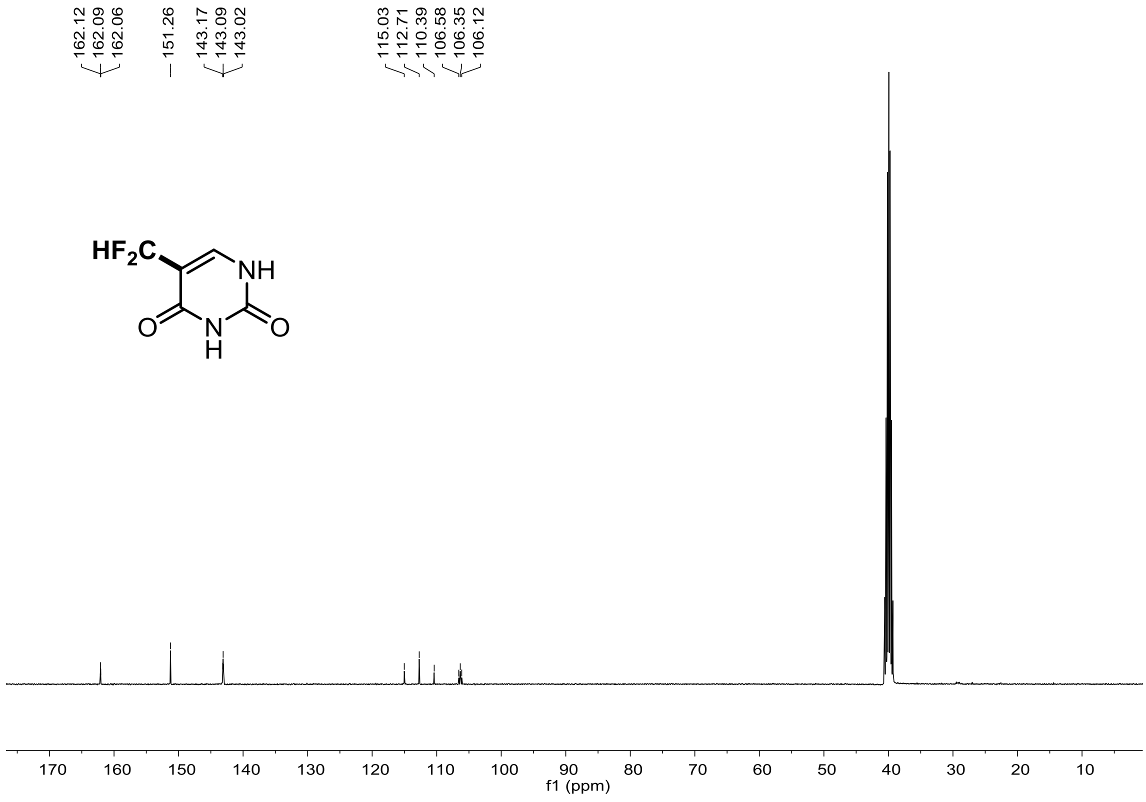

Supplementary Figure 122. ^{13}C NMR Spectrum of **6l**

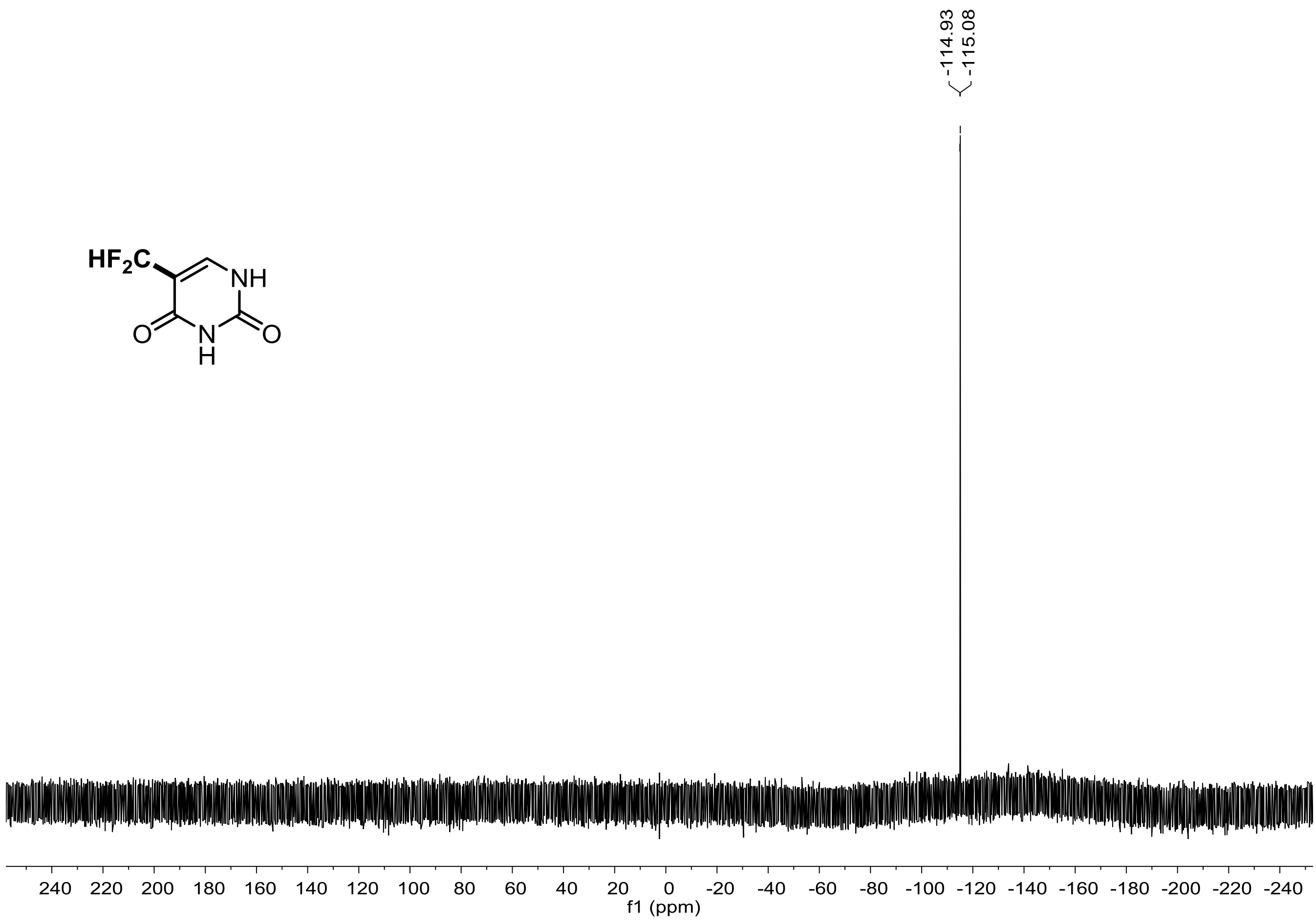

Supplementary Figure 123. ^{19}F NMR Spectrum of **6l**

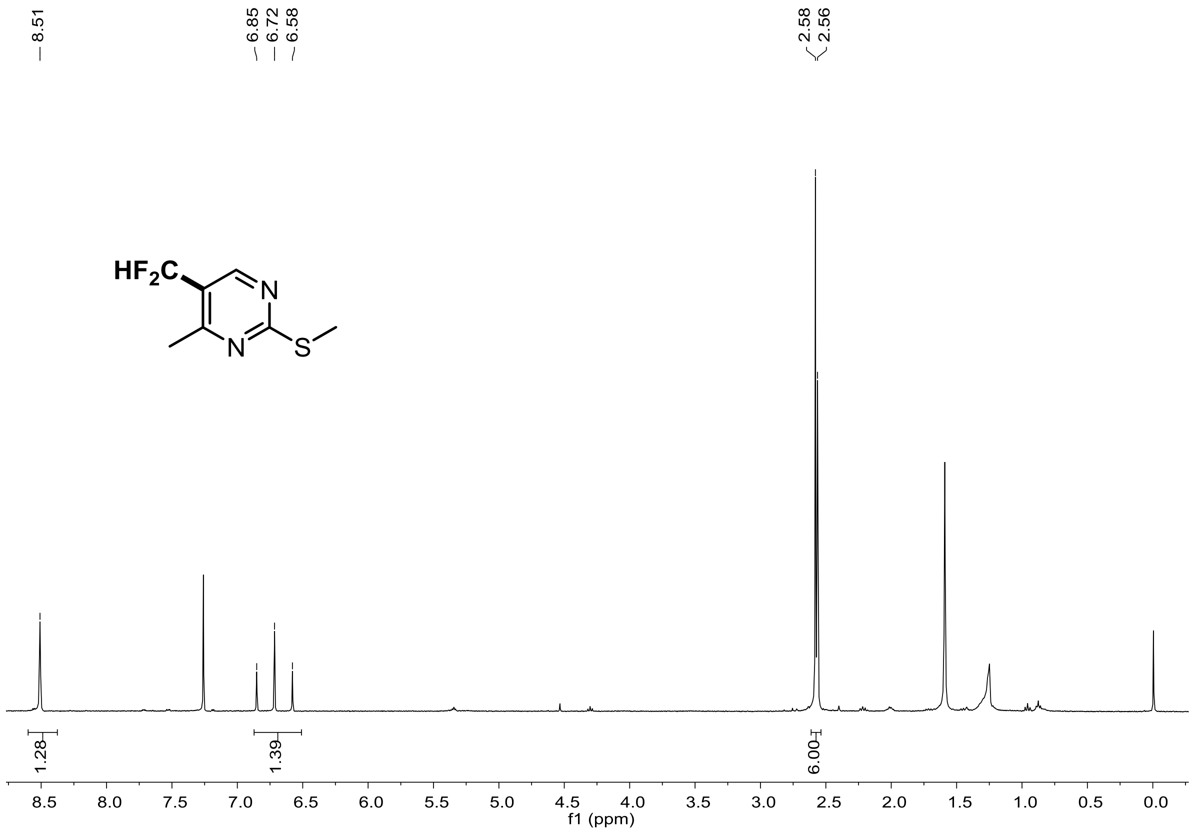

Supplementary Figure 124. ^{1}H NMR Spectrum of **6m**

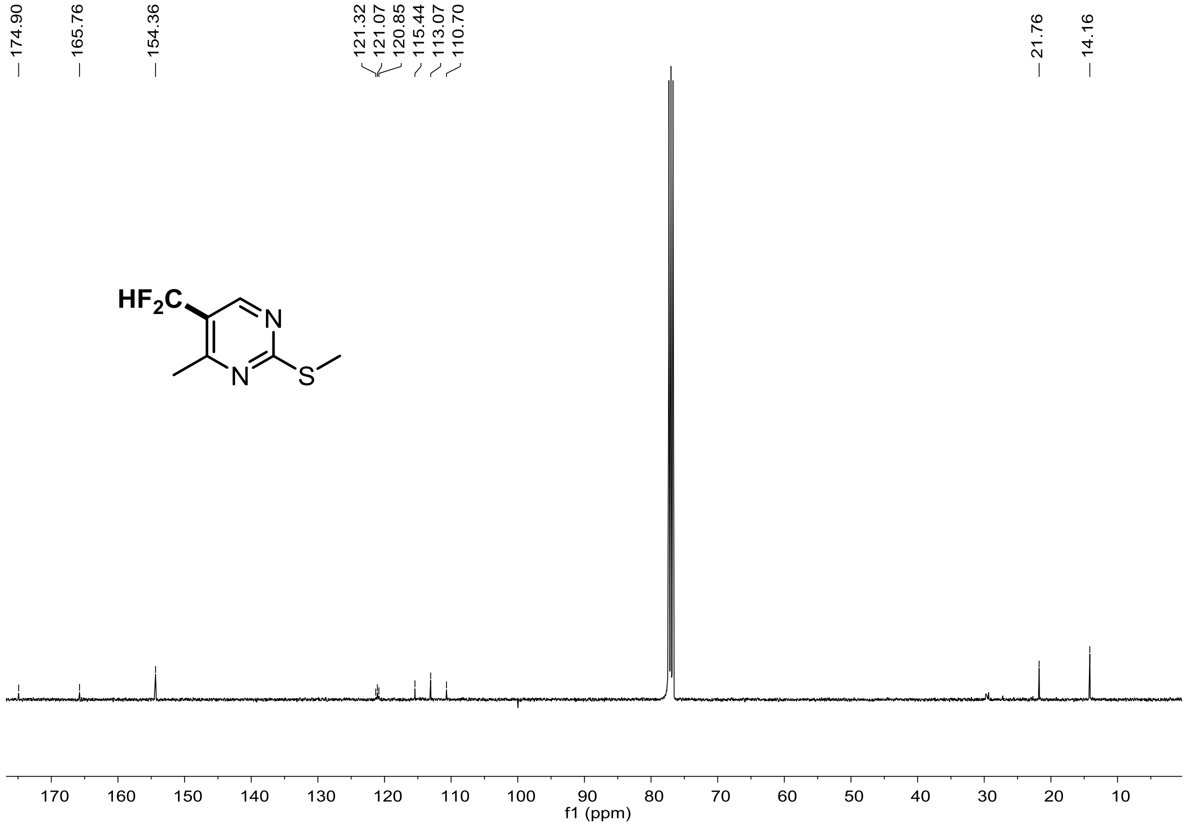

Supplementary Figure 125. ^{13}C NMR Spectrum of **6m**

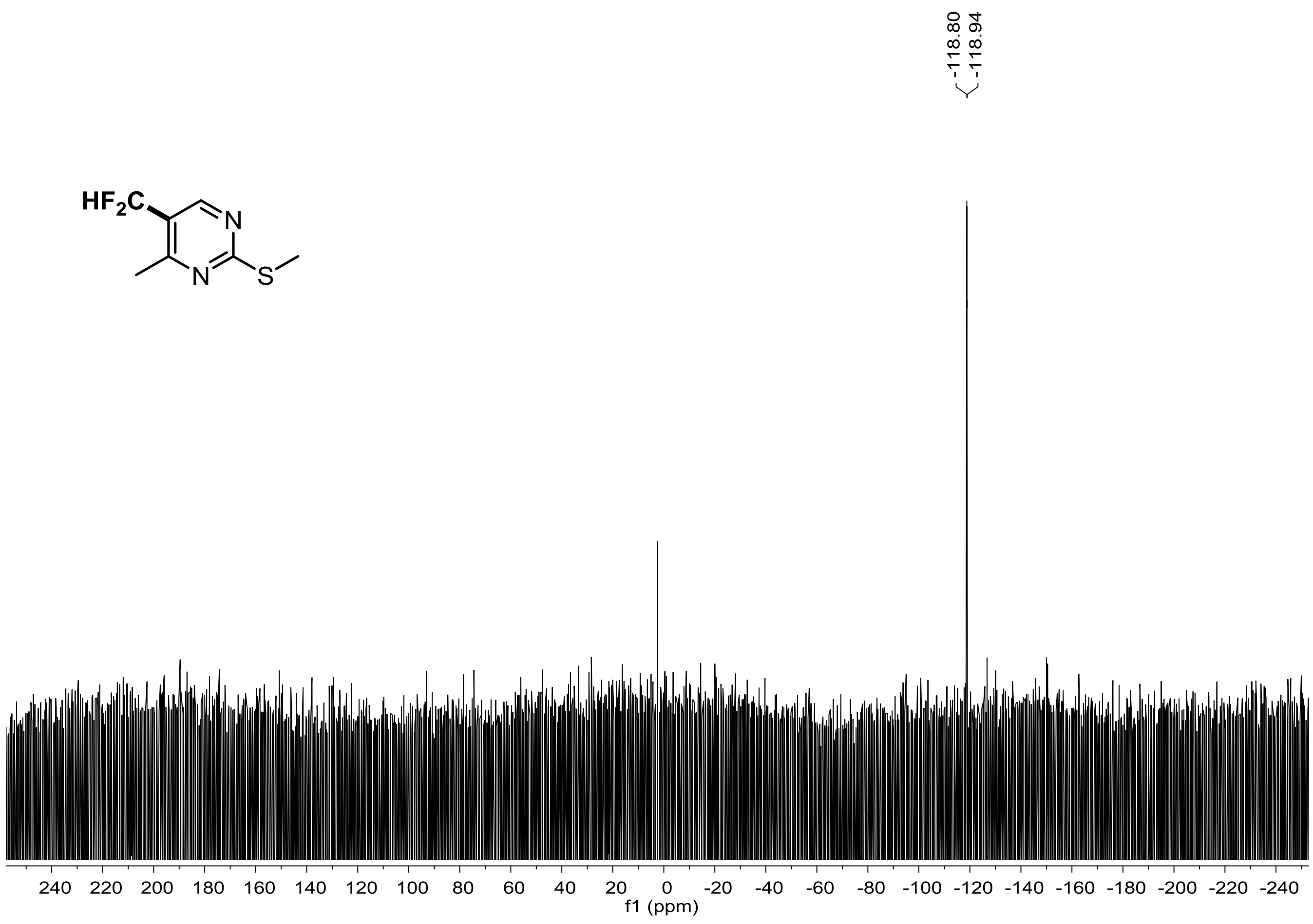

Supplementary Figure 126. ^{19}F NMR Spectrum of **6m**

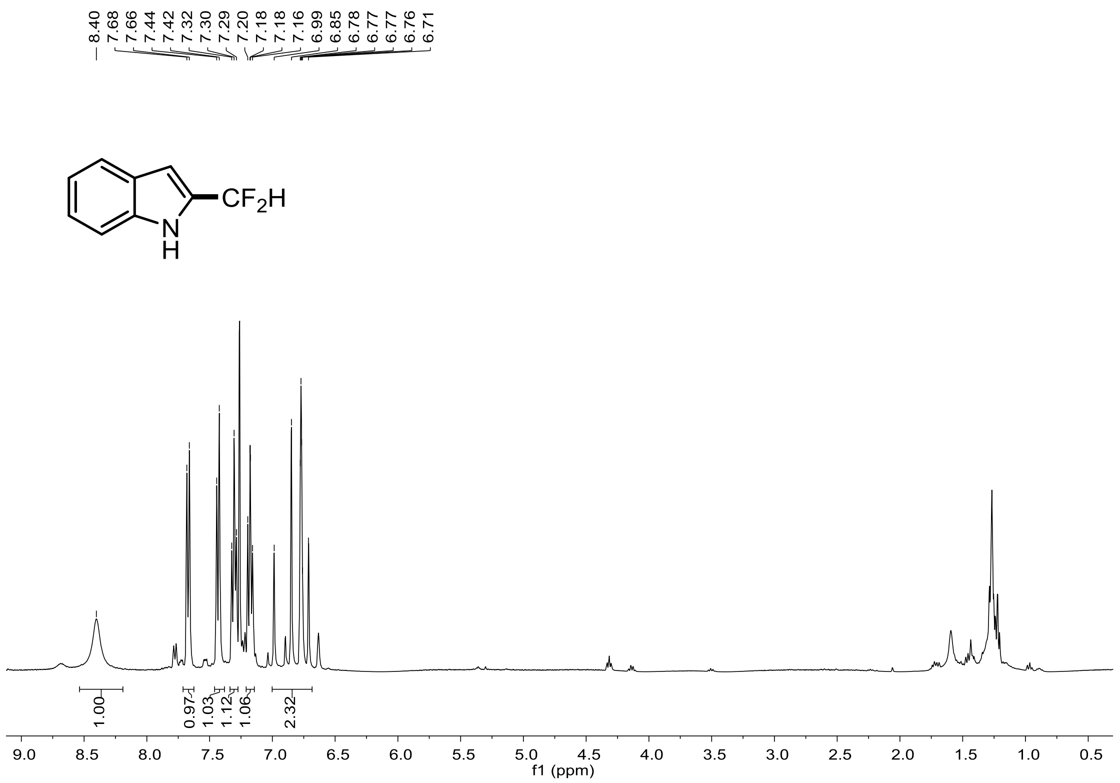

Supplementary Figure 127. ^{1}H NMR Spectrum of **8**

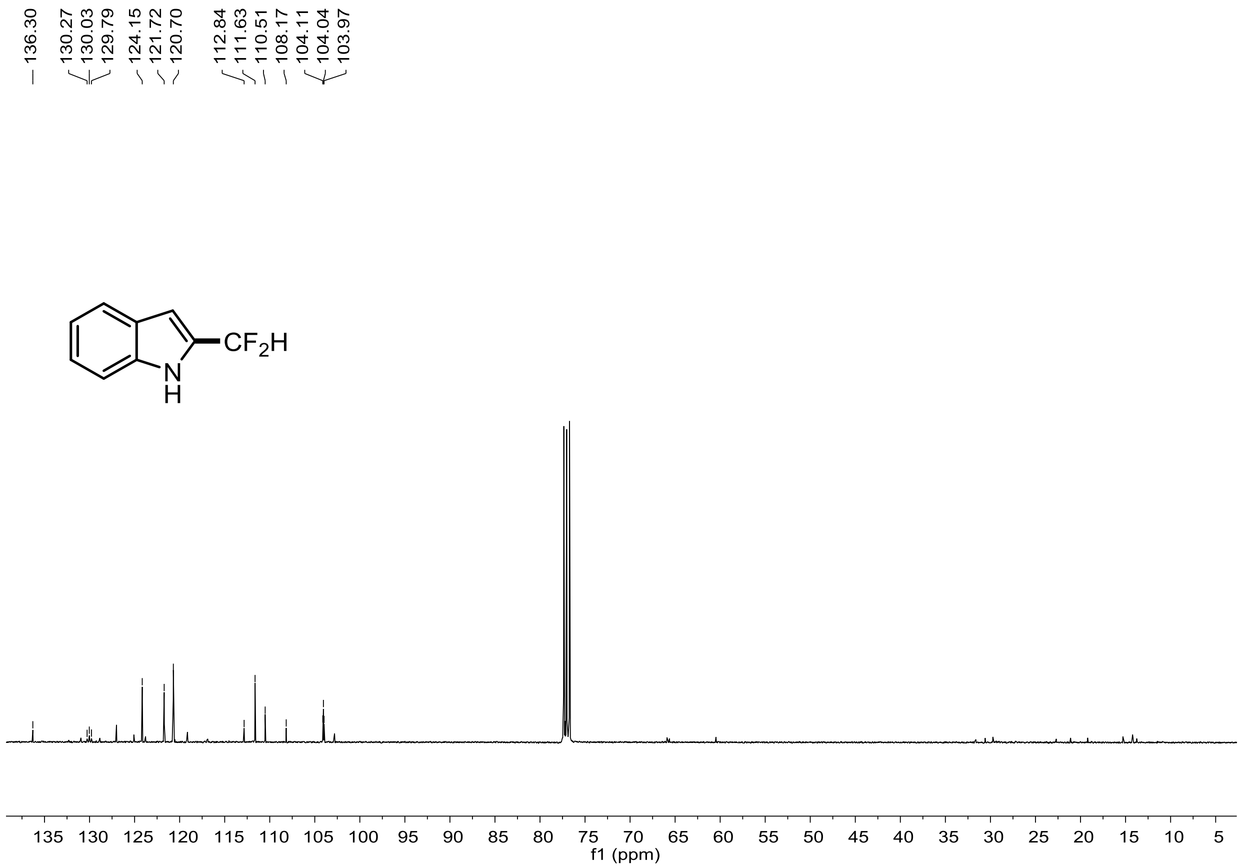

Supplementary Figure 128. ^{13}C NMR Spectrum of **8**

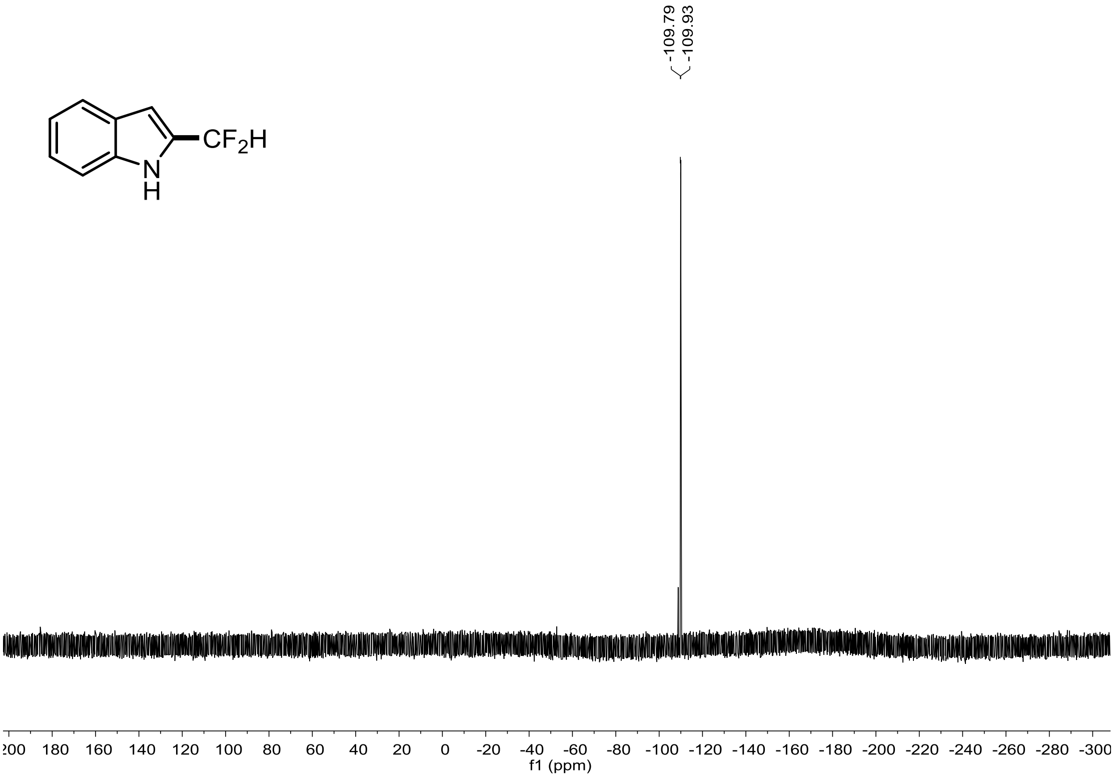

Supplementary Figure 129. ^{19}F NMR Spectrum of **8**

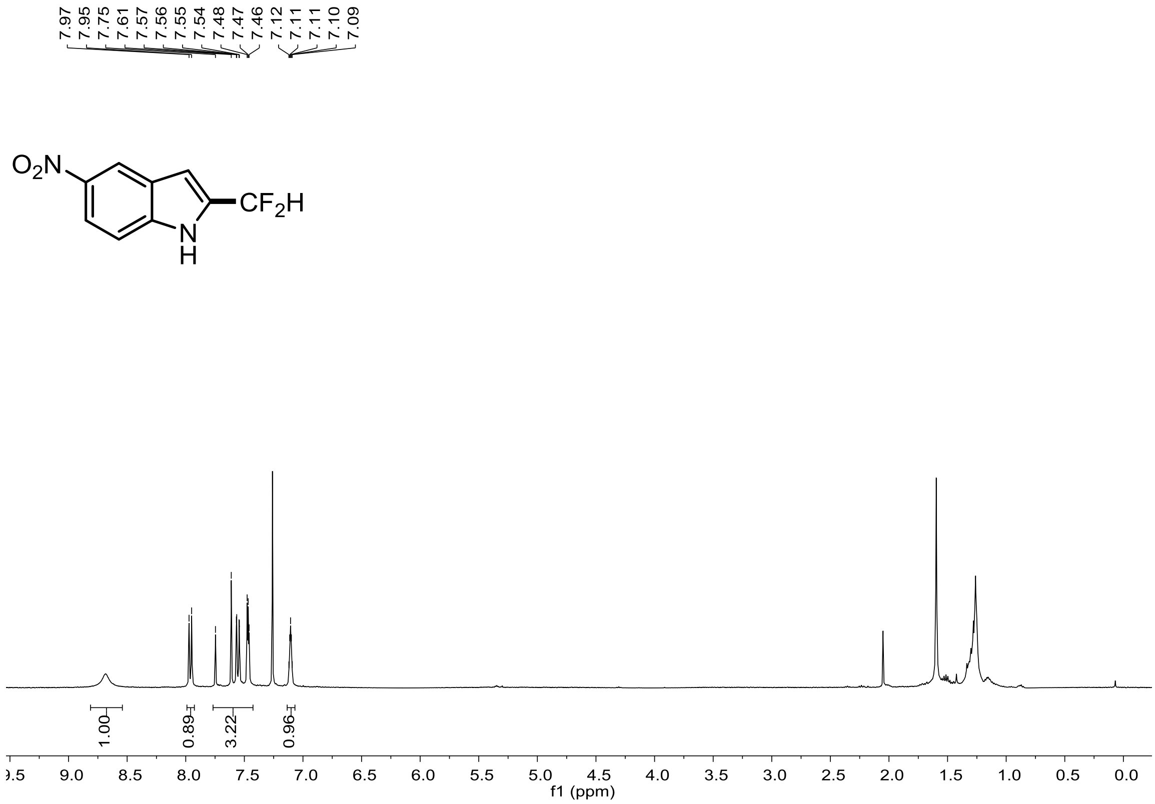

Supplementary Figure 130. ^{1}H NMR Spectrum of **10**

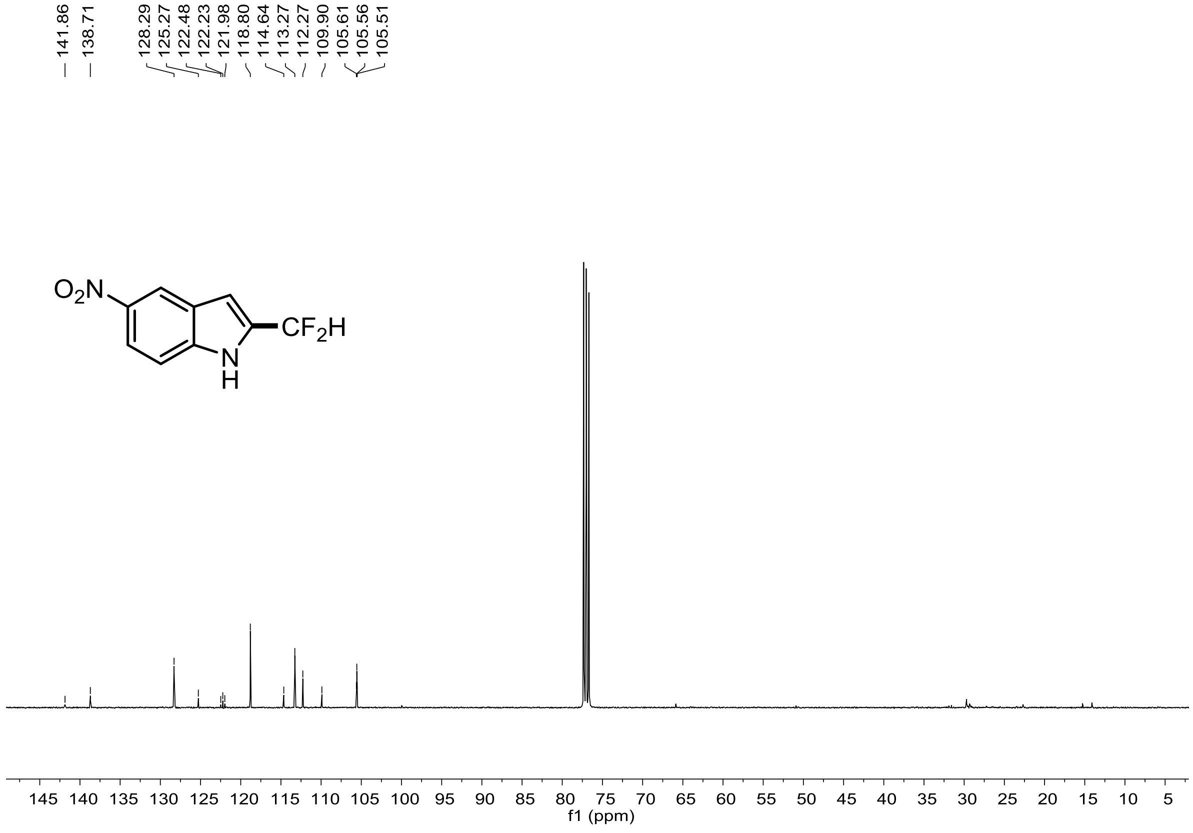

Supplementary Figure 131. ^{13}C NMR Spectrum of **10**

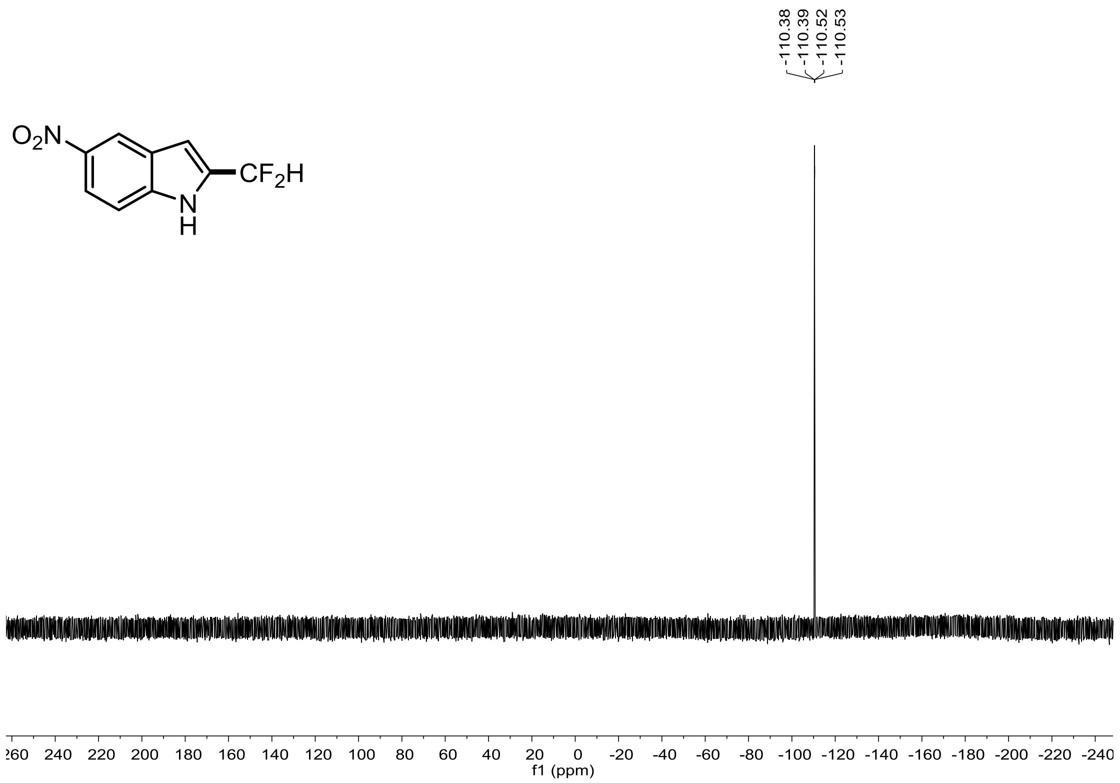

Supplementary Figure 132. ^{19}F NMR Spectrum of **10**

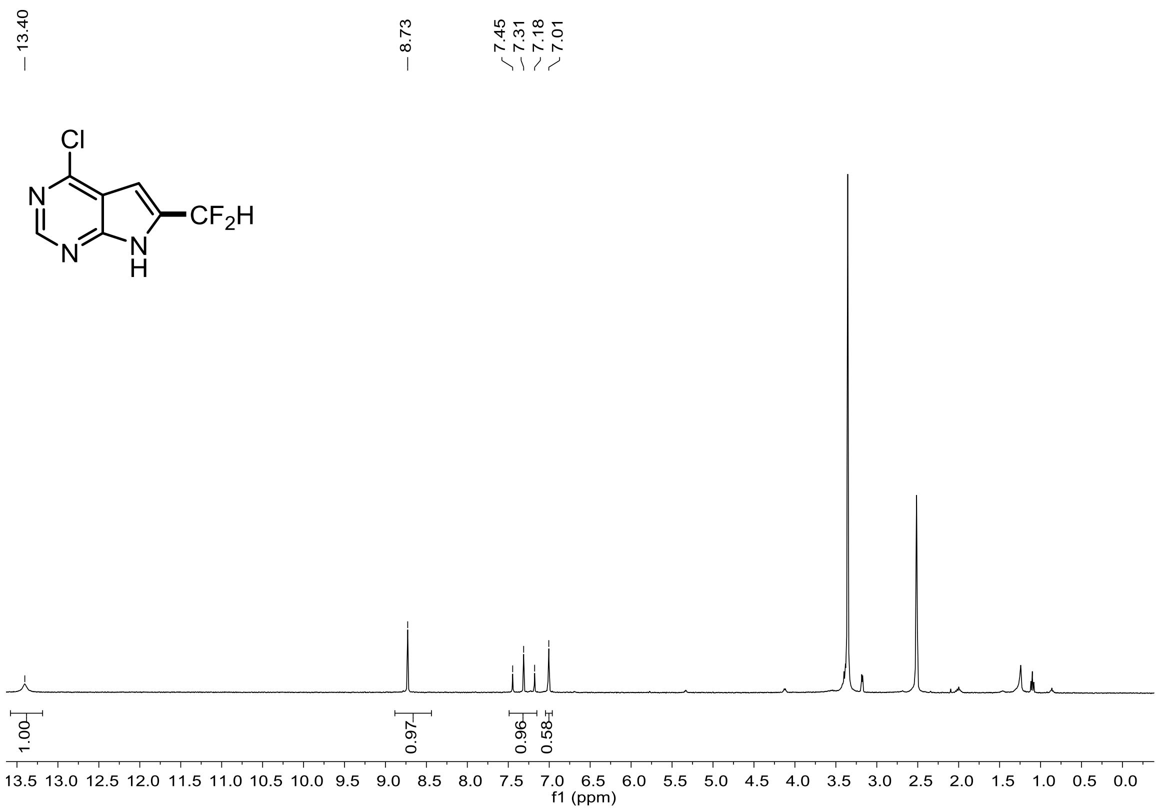

Supplementary Figure 133. ^{1}H NMR Spectrum of **12**

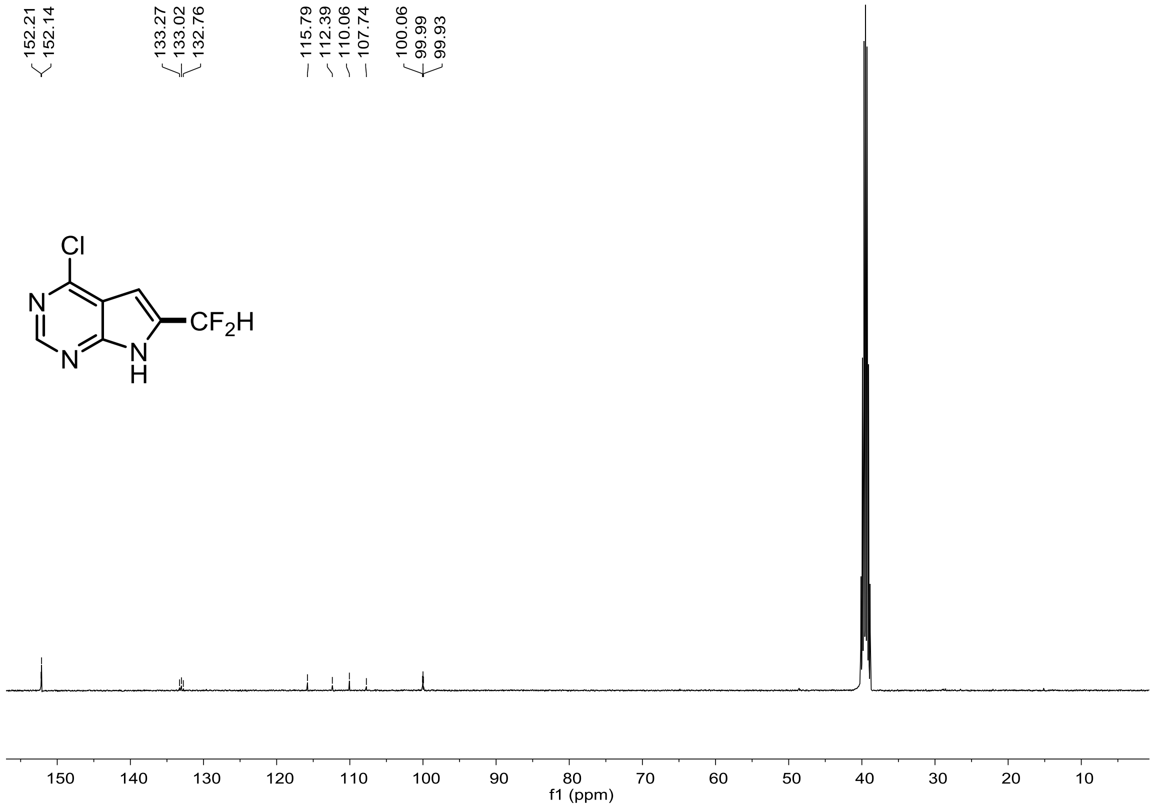

Supplementary Figure 134. ^{13}C NMR Spectrum of **12**

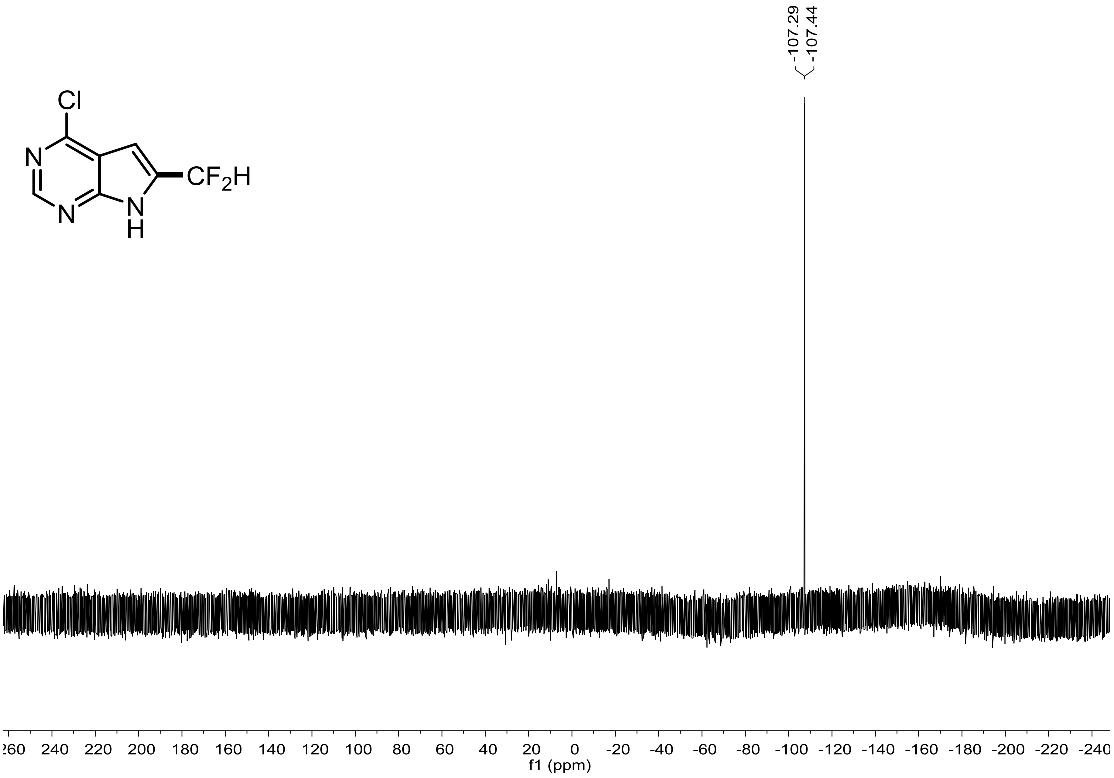

Supplementary Figure 135. ^{19}F NMR Spectrum of **12**

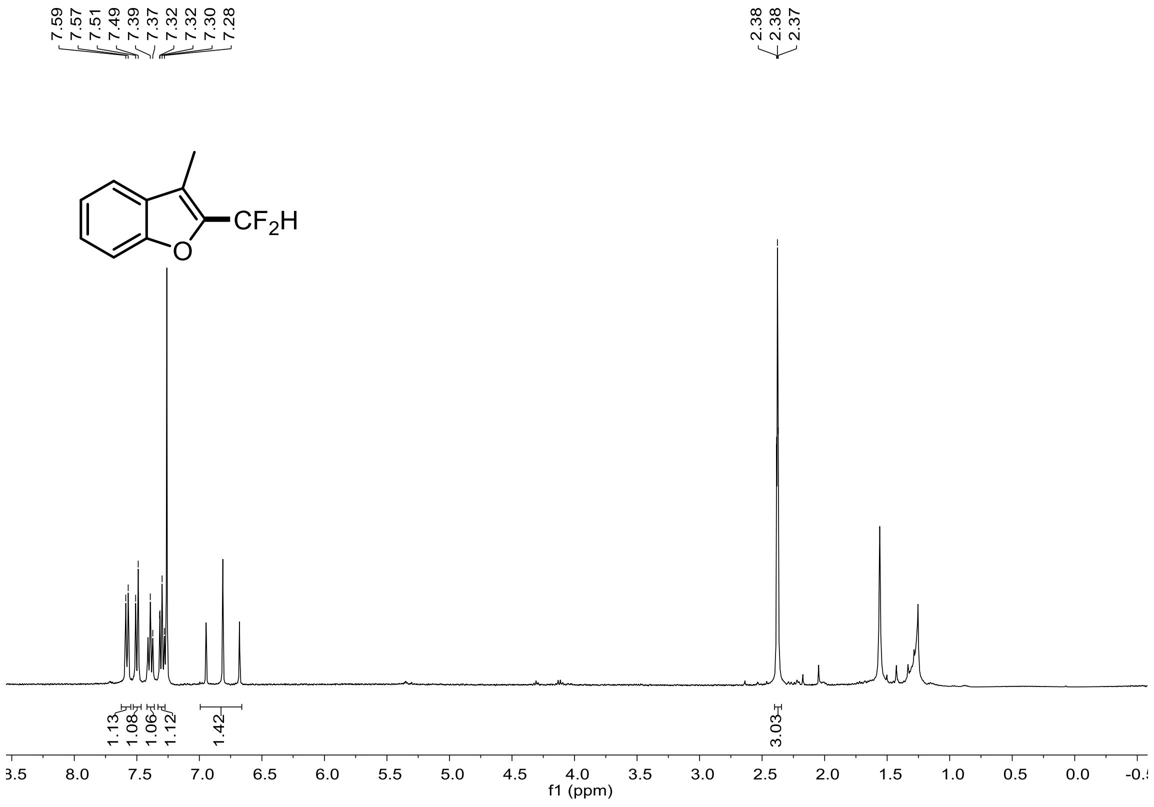

Supplementary Figure 136. ^{1}H NMR Spectrum of **14**

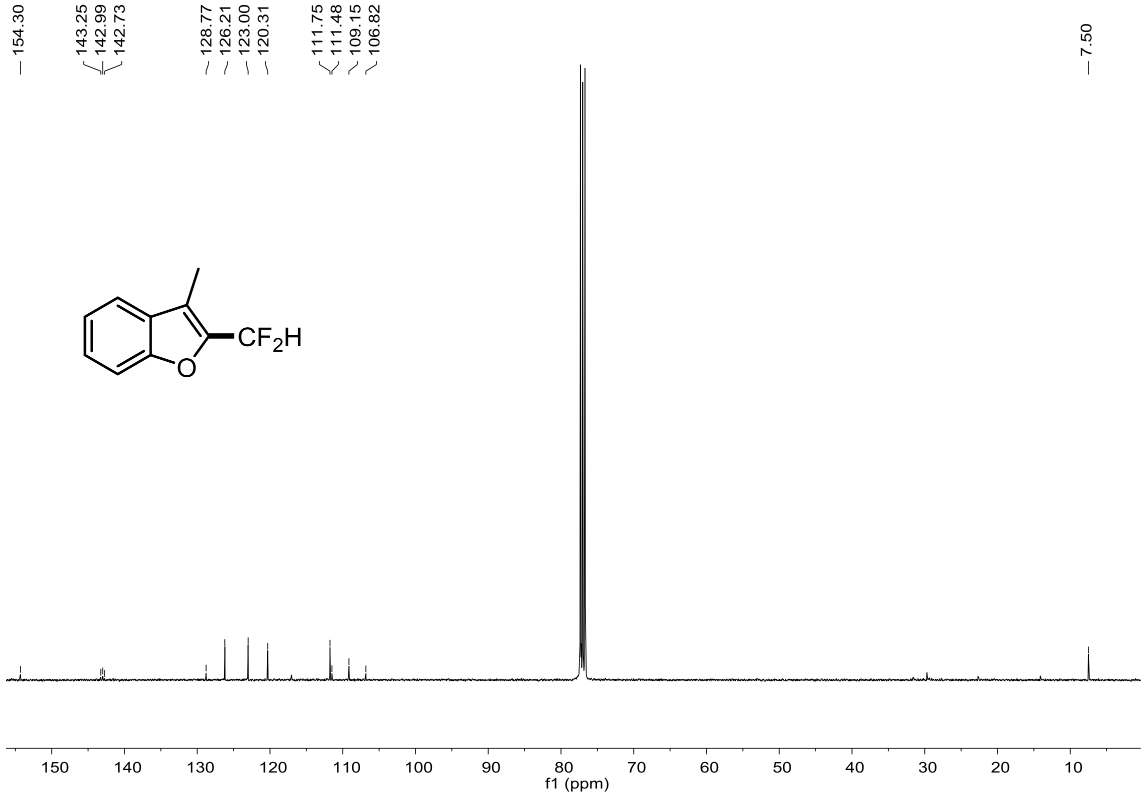

Supplementary Figure 137. ^{13}C NMR Spectrum of **14**

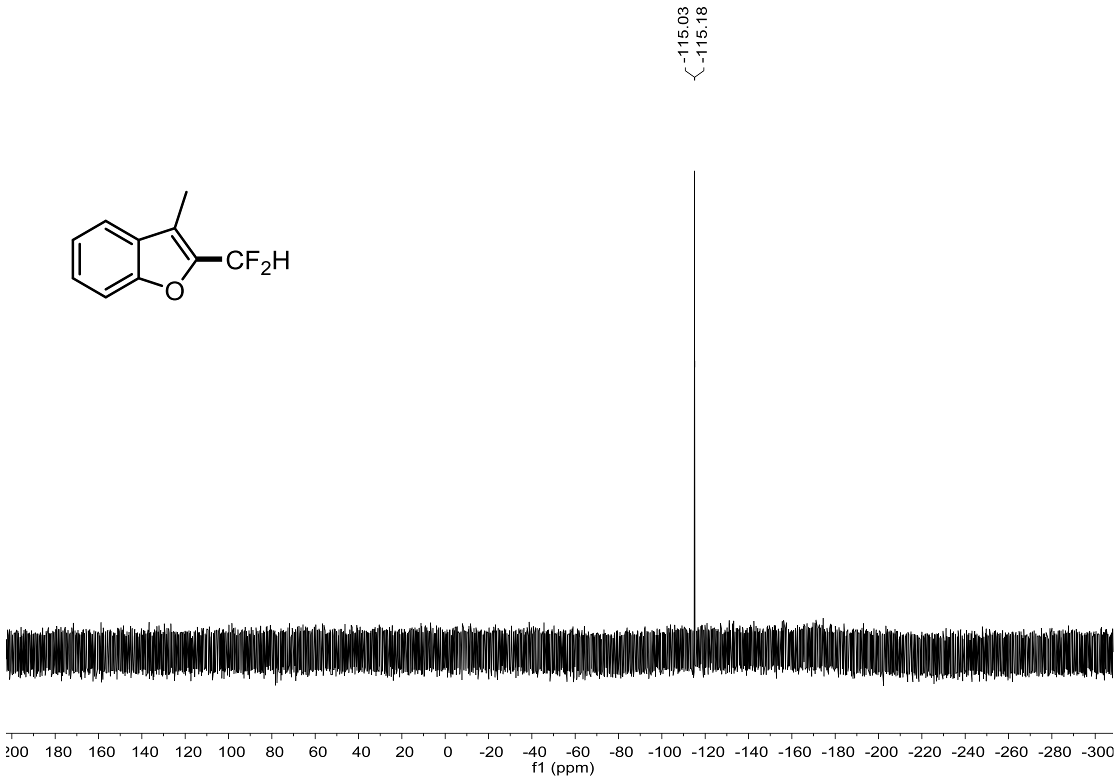

Supplementary Figure 138. ^{19}F NMR Spectrum of **14**

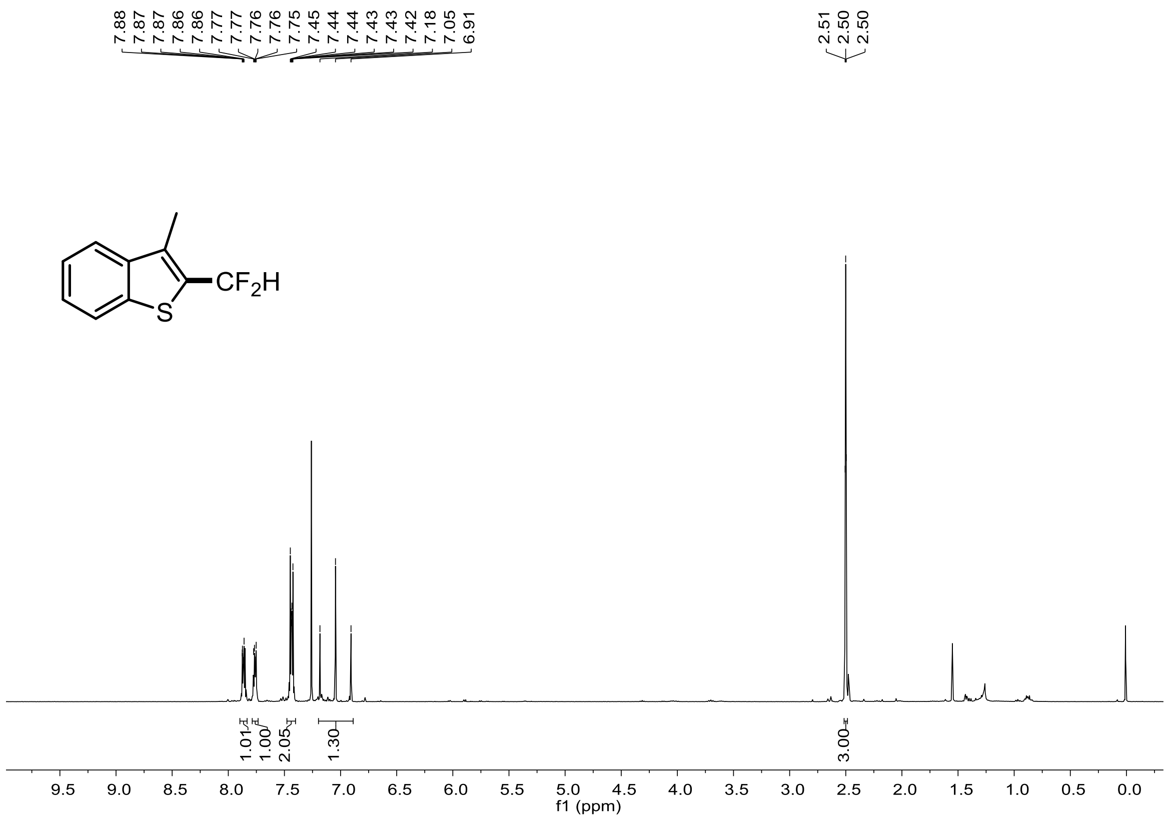

Supplementary Figure 139. ^{1}H NMR Spectrum of **16**

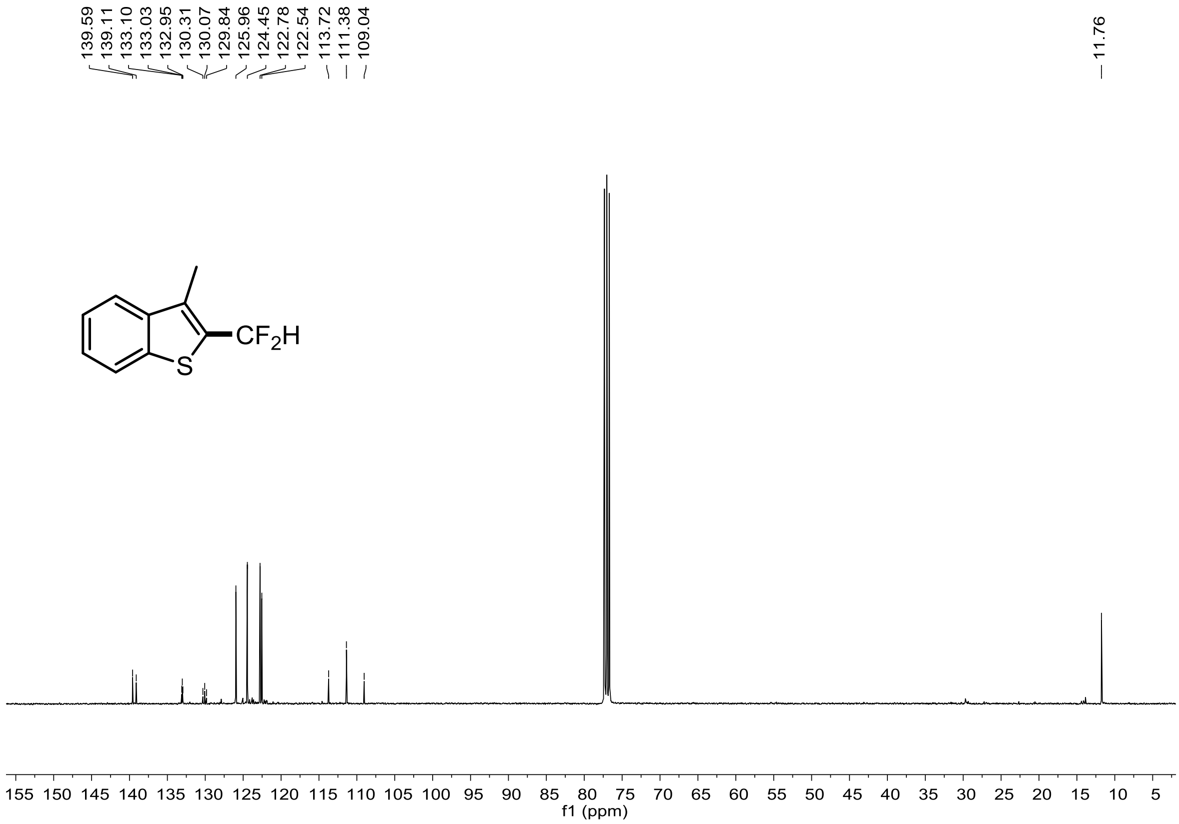

Supplementary Figure 140. ^{13}C NMR Spectrum of **16**

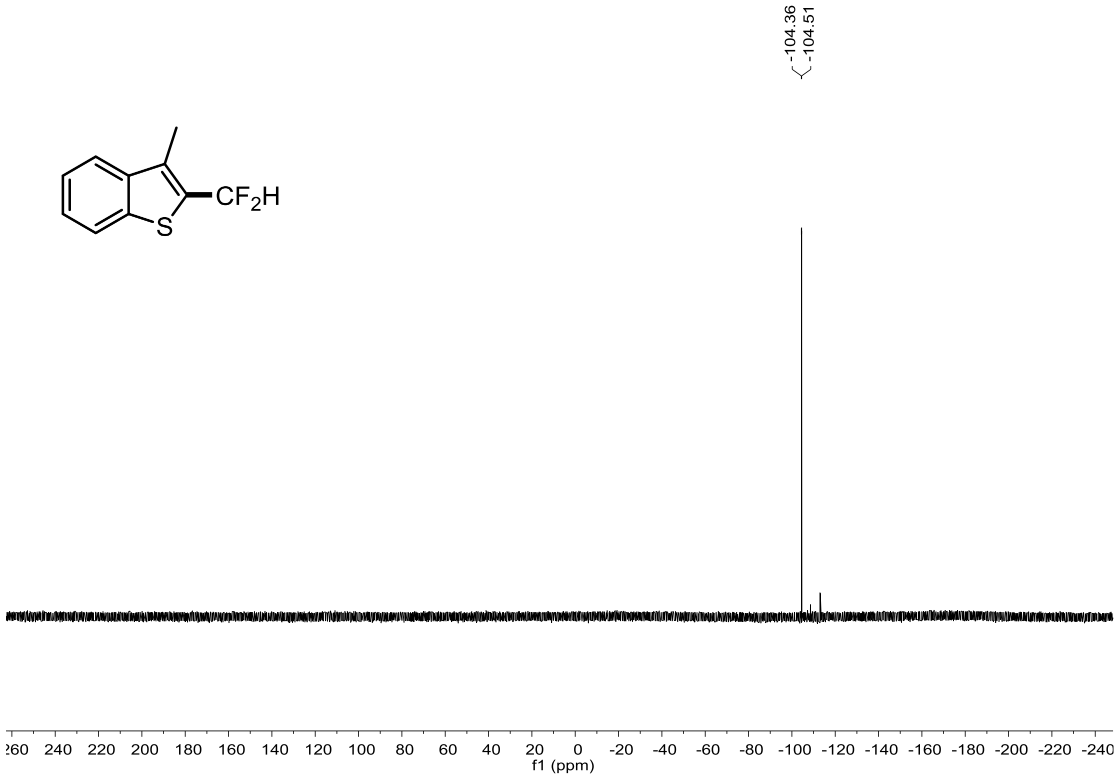

Supplementary Figure 140. ^{19}F NMR Spectrum of **16**

Supplementary References

1. Fujiwara, Y., Dixon, J. A., Rodriguez, R. A., Baxter, R. D., Dixon, D. D., Collins, M. R., Blackmond, D. G. & Baran, P. S. A New Reagent for Direct Difluoromethylation. *J. Am. Chem. Soc.* **134**, 1494-1497 (2012)

2. Sakamoto, R., Kashiwagi, H. & Maruoka, K. The Direct C–H Difluoromethylation of Heteroarenes Based on the Photolysis of Hypervalent Iodine(III) Reagents That Contain Difluoroacetoxy Ligands. *Org. Lett.* **19**, 5126-5129 (2017)

3. Tung, T. T., Christensen, S. B. & Nielsen, J. Difluoroacetic Acid as a New Reagent for Direct C-H Difluoromethylation of Heteroaromatic Compounds. *Chem.- Eur. J.* **23**, 18125-18128 (2017)

4. Zhu, S., Liu, Y., Li, H., Xu, X. & Qing, F. Direct and Regioselective C-H Oxidative Difluoromethylation of Heteroarenes. *J. Am. Chem. Soc.* **140**, 11613-11617 (2018)

5. Zhang,W., Pan,Y.-L., Yang, C., Chen, L., Li , X. & Cheng, J.-P. Metal-Free Direct C–H Cyanoalkylation of Quinoxalin-2(1*H*)-Ones by Organic Photoredox Catalysis. *J. Org. Chem.* **84**, 7786-7795 (2019**)**

6. Liu, W. Q., Lei, T., Song, Z.-Q., Yang, X.-L., Wu, C. J., Jiang, X., Chen, B., Tung, C.-H. & Wu, L.-Z. Visible Light Promoted Synthesis of Indoles by Single Photosensitizer under Aerobic Conditions. *Org. Lett.* **19**, 3251−3254 (2017)
